# Palladium‐Catalyzed Atroposelective Suzuki–Miyaura Coupling to Construct Axially Chiral Tetra‐Substituted α‐Boryl Styrenes

**DOI:** 10.1002/advs.202309706

**Published:** 2024-04-11

**Authors:** Xiaorui Li, Lingyu Kong, Shuxin Yin, Hengrui Zhou, Aijun Lin, Hequan Yao, Shang Gao

**Affiliations:** ^1^ State Key Laboratory of Natural Medicines (SKLNM) and Department of Medicinal Chemistry School of Pharmacy China Pharmaceutical University Nanjing 210009 P. R. China

**Keywords:** atroposelective synthesis, axially chiral tetra‐substituted styrenes, *gem*‐diboryl alkenes, palladium‐catalyzed, Suzuki–Miyaura coupling

## Abstract

Palladium‐catalyzed Suzuki–Miyaura (SM) coupling is a valuable method for forming C─C bonds, including those between aryl moieties. However, achieving atroposelective synthesis of axially chiral styrenes via SM coupling remains challenging. In this study, a palladium‐catalyzed atroposelective Suzuki–Miyaura coupling between *gem*‐diborylalkenes and aryl halides is presented. Using the monophosphine ligand Me‐BI‐DIME (**L2**), a range of axially chiral tetra‐substituted acyclic styrenes with high yields and excellent enantioselectivities are successfully synthesized. Control experiments reveal that the *gem*‐diboryl group significantly influences the product enantioselectivities and the coupling prefers to occur at sites with lower steric hindrance. Additionally, the alkenyl boronate group in the products proves versatile, allowing for various transformations while maintaining high optical purities.

## Graphical abstract

A palladium‐catalyzed chemoselective and atroposelective Suzuki–Miyaura coupling between aryl halides and *gem*‐diboryl alkenes is developed. Various tetra‐substituted axially chiral α‐boryl styrenes are constructed with excellent chemoselectivity and enantioselectivity. Control experiments indicate the coupling prefers to occur at sites with lower steric hindrance. The alkenyl boronate group in the products proves versatile, allowing for various transformations with excellent enantioretention.

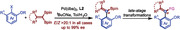

## 1 Introduction

**Scheme 1:**
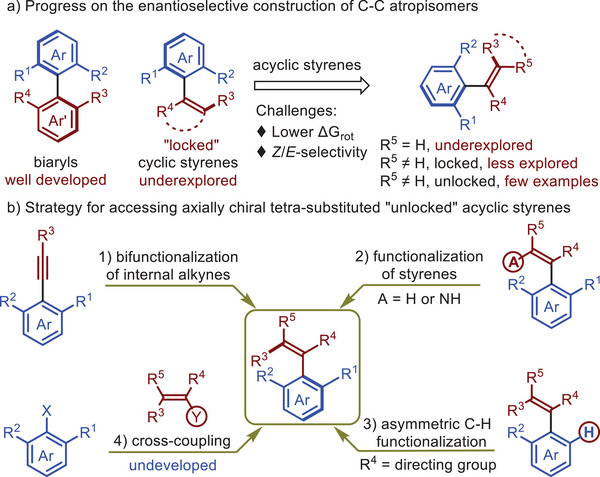
Strategies for the atroposelective syntheses of unlocked tetra‐substituted acyclic styrenes.

Atropisomerism, a type of stereoisomerism resulting from restricted rotation around a single bond, is widely found in natural products, bioactive molecules and chiral ligands.^[^ ^1^ ^]^ In recent decades, tremendous effort has been devoted to the development of novel methods to construct axially chiral molecules, particularly C─C atropisomers.^[^ ^2^ ^]^ While significant advancement has been achieved on atroposelective syntheses of axially chiral biaryl compounds,^[^ ^3^ ^]^ constructing axially chiral tri‐ or tetra‐substituted acyclic alkenes remains a challenging task (**Scheme** **1**a).^[^ ^4^ ^]^ To date, very few examples of atroposelective synthesis have been reported for achieving tetrasubstituted acyclic styrenes, such as enantioselective bifunctionalization of internal alkynes,^[^ ^5^ ^]^ functionalization of preformed tri‐ or tetra‐substituted styrenes,^[^ ^6^ ^]^ and C─H functionalization of the arene of tetra‐substituted styrenes^[^ ^7^ ^]^ (Scheme 1b). Giving the increasing importance of axially chiral styrenes, further development of novel atroposelective synthetic methods is highly desirable.

**Scheme 2:**
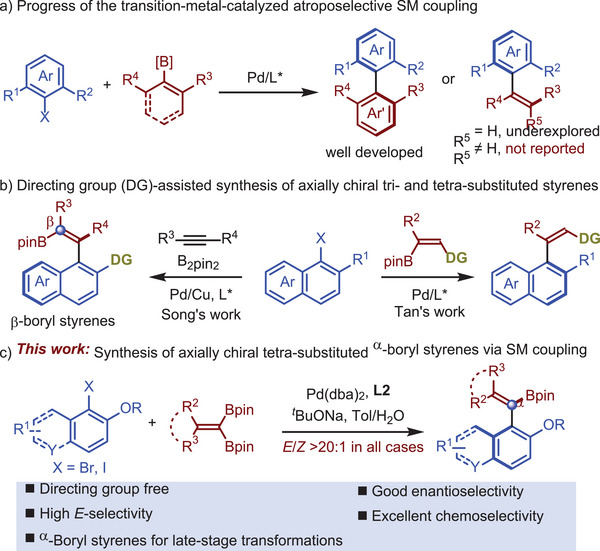
Palladium‐catalyzed atroposelective Suzuki–Miyaura coupling to construct axially chiral styrenes.

Transition metal‐catalyzed Suzuki–Miyaura (SM) coupling is a highly effective method for constructing C─C bonds. However, the construction of C─C atropisomers via Suzuki–Miyaura coupling has not been extensively explored in the synthetic community. In 2000, the Buchwald and Cammidge groups independently reported seminal work on atroposelective Suzuki coupling to synthesize axially chiral biaryl compounds.^[^ ^8^ ^]^ With the rapid development of novel chiral ligands,^[^ ^9^ ^]^ asymmetric Suzuki–Miyaura coupling to generate biaryl atropisomers has been intensively studied.^[^ ^10^ ^]^ In contrast, atroposelective syntheses of axially chiral tri‐ and tetra‐substituted acyclic olefins largely remain dormant (**Scheme** **2**a). In 2022, Tan and co‐workers reported a palladium‐catalyzed atroposelective Suzuki–Miyaura coupling between aryl bromides and vinyl boronates.^[^ ^11^ ^]^ With the assistance of a directing group, trisubstituted axially chiral acyclic olefins were obtained with excellent enantioselectivities and good *Z*‐selectivities. However, attempts to construct tetra‐substituted alkene analogues were unsuccessful (Scheme 2b, right). Song's group recently realized an atroposelective synthesis of axially chiral tetra‐substituted styrenes, where the boryl group resides at the *β*‐position (Scheme 2b, left).^[^ ^5b^ ^]^ *gem*‐Diboryl alkenes are versatile building blocks that can be used to construct substituted alkenes via chemodivergent functionalization of the two boryl groups.^[^ ^12^ ^]^ We were intrigued whether *gem*‐diboryl alkenes could be utilized to synthesize axially chiral tetra‐substituted *α*‐boryl styrenes (Scheme 2c). To achieve this goal, several challenges need to be addressed. First, the control of chemoselectivity is essential for this atroposelective Suzuki–Miyaura coupling. Second, further reactions with the coupling products need to be minimized. Lastly, it is not clear whether derivatization of the *α*‐boryl styrene products will retention the optical purities at the outset of our studies, although Song's work provides an encouraging example on enantioretention during their late‐stage transformations of enantioenriched *β*‐boryl styrenes.

Given our ongoing interest in constructing of axially chiral molecules and enantioselective synthesis of alkenyl‐boronates,^[^ ^13^ ^]^ we report herein a palladium‐catalyzed atroposelective Suzuki–Miyaura coupling between aryl halides and *gem*‐diborylalkenes (Scheme 2c). With a monophosphine, Me‐BI‐DIME (**L2**), as the ligand, a series of axially chiral tetra‐substituted *α*‐boryl styrenes were synthesized in good yields with excellent enantioselectivities and *E*‐selectivities. Notably, the reaction does not require any directing group. Moreover, the alkenyl boronate unit in the products can directly undergo various transformations, allowing for further functionalization of the coupling products.

## 2 Results

### 2.1 Reaction Development

We initiated our studies with 1‐iodo‐2‐methoxynaphthalene (**1a**) and *gem*‐diborylalkene **2a** as the model substrates to establish an effective catalytic system. The representative results are summarized in **Table** **1**. First, a variety of bases were examined in the presence of 10 mol % Pd(OAc)_2_, 15 mol % BINAP (**L1**) and a mixed solvent system (toluene‐H_2_O) at 30 °C for 48 h (Table 1, entries 1–4). It was found that the choice of base significantly influenced the conversion of the starting materials and the yield of **3a**. *^t^* BuONa was identified as the optimal base, affording **3a** in 64% yield. However, the enantiopurity of **3a** is rather poor (16% ee). Further evaluation of the chiral ligands indicated that monophosphine ligands **L2**–**L4** had better enantioinduction, delivering **3a** in 30–82% yields with 38–60% ee (entries 5–7). Inferior enantioselectivities were observed with THF or PhCF_3_ as solvents (entries 8, 9). Screening of palladium catalysts showed that Pd(dba)_2_ was optimal, affording **3a** in 88% yield with 57% ee (entries 10, 11). By reducing the reaction temperature to 20 °C and prolonging reaction time to 72 h, the enantiomeric purity of **3a** was improved to 86% ee without any decrease in yield (entry 12).

**Table 1:** Optimization of the reaction conditions.

Entry^a)^	[Pd]	Ligand	Base	Yield [%]	Ee [%]
1	Pd(OAc)_2_	**L1**	K_3_PO_4_	10	15
2	Pd(OAc)_2_	**L1**	KOH	28	15
3	Pd(OAc)_2_	**L1**	*^t^* BuOK	35	14
4	Pd(OAc)_2_	**L1**	*^t^* BuONa	64	16
5	Pd(OAc)_2_	**L2**	*^t^* BuONa	82	59
6	Pd(OAc)_2_	**L3**	*^t^* BuONa	30	38
7	Pd(OAc)_2_	**L4**	*^t^* BuONa	40	60
8^b)^	Pd(OAc)_2_	**L2**	*^t^* BuONa	85	19
9^c)^	Pd(OAc)_2_	**L2**	*^t^* BuONa	96	49
10	PdCl_2_	**L2**	*^t^* BuONa	72	57
11	Pd(dba)_2_	**L2**	*^t^* BuONa	88	57
12^d)^	Pd(dba)_2_	**L2**	*^t^* BuONa	90	86

^a)^ Reaction conditions: **1a** (0.1 mmol, 1.0 equiv), **2a** (0.12 mmol, 1.2 equiv), [Pd] (10 mol %), **L** (15 mol %) and base (3.0 equiv in 0.2 mL H_2_O) in 1.0 mL toluene at 30 °C under argon atmosphere for 48 h. Yields were determined by ^1^H NMR with 1,3,5‐trimethoxybenzene as the internal standard. The enantiomeric excesses were determined by HPLC;

^b)^ THF was used;

^c)^ PhCF_3_ was used;

^d)^ The reaction was stirred at 20 °C for 72 h and isolated yield was listed.

Next, various substituents at the *β*‐position of *α*‐halogenated naphthalene **1** were tested with Me‐BI‐DIME (**L2**) as the ligand (**Table** **2**). In general, *α*‐halogenated naphthalene containing an alkoxyl group at the *β*‐position performed well in the atroposelective coupling reactions. The size of the alkyl group (R^1^) has a substantial effect on the enantioselectivity of the reaction. Coupling products **3a**‐**f** were obtained in 71–90% yields with 84–94% ee, with isopropyl being the optimal group. The reaction of aryl bromide **1e’** provided **3e** in 90% yield with 92% ee. Product **3** **g**, containing an isopropylthio group at the C2‐position, was obtained in 76% yield with 84% ee, but the enantioselectivities of products **3** **h** and **3i** decreased significantly. In addition, no reaction occurred when a dimethylamine or N‐protected amine group was introduced to the C2‐position. The reaction could be easily scaled up to 1 mmol without any loss of the efficiency, yielding **3e** in 88% yield with 94% ee.

**Table 2:** Evaluation of the β‐substituent of α‐halogenated naphthalene.

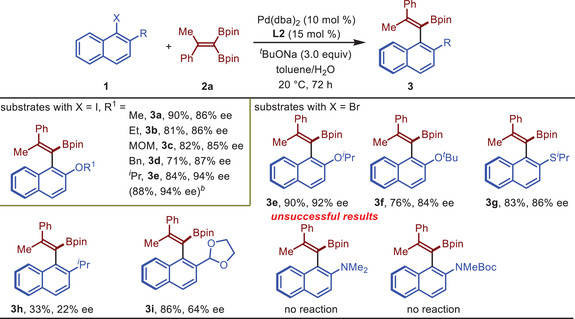 ^a)^

^a)^ Reaction conditions: **1** (0.1 mmol, 1.0 equiv), **2a** (0.12 mmol, 1.2 equiv), Pd(dba)_2_ (10 mol %), **L2** (15 mol %) and *^t^* BuONa (3.0 equiv in 0.2 mL H_2_O) in 1.0 mL toluene at 20 °C under an argon atmosphere for 72 h. Isolated yields were listed. The enantiomeric excesses were determined by HPLC;

^b)^ On 1.0 mmol scale.

### 2.2 Substrate Scope

With the optimized conditions established, we next examined the scope of this atroposelective synthesis of tetra‐substituted styrenes. **Table** **3** summarizes our findings. The reaction proved tolerant to naphthyl iodides **1** containing either electron‐donating or electron‐withdrawing groups at the C4‐, C5‐ or C6‐position of the naphthyl group, giving **4a**–**g** in 59–91% yields with 91–96% ee. This atroposelective cross‐coupling was also applicable to substituted 5‐iodoquinoline, affording product **4** **h** in 80% yield with 93% ee. Then various *gem*‐diboryl alkenes were investigated. Subsequent investigation of various *gem*‐diboryl alkenes revealed excellent discrimination of the two geminal boryl groups for 2‐aryl‐2‐alkyl *gem*‐diborylalkenes, providing **4i**–**p** in 69–90% yields with 91–97% ee. Notably, the formation of the corresponding diastereomer, generated from the coupling with the boryl group cis to the aryl group, was not observed. Next, the reactions with various symmetrical *gem*‐diboryl alkenes were examined. (Diborylmethylene)cyclohexane reacted smoothly with several O‐substituted naphthyl iodides, providing **4q–u** in 64–89% yields with 88–97% ee.

**Table 3:** Substrate scope.

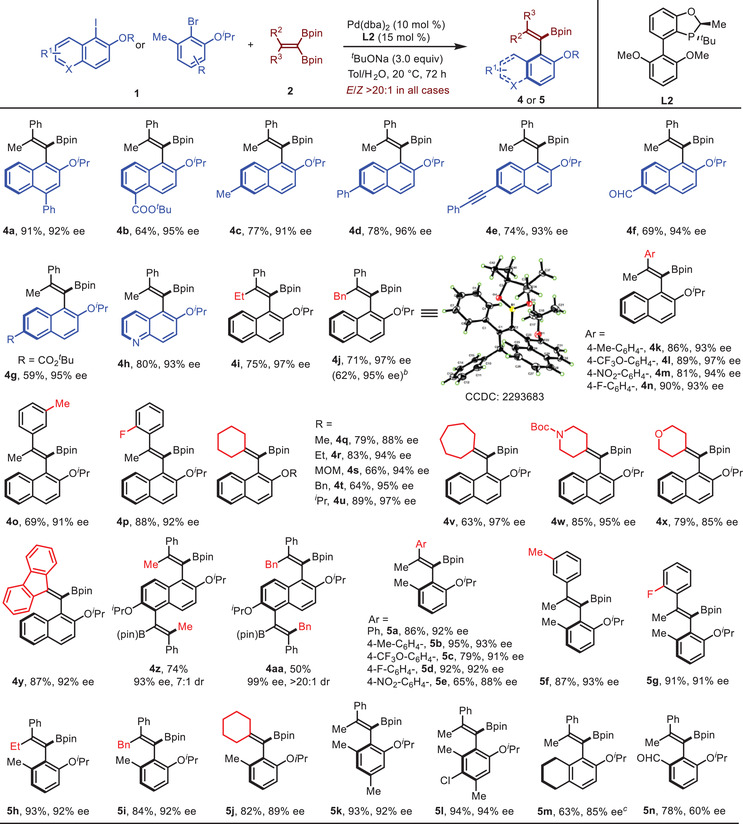 ^a)^

^a)^ Reaction conditions: **1** (0.1 mmol, 1.0 equiv), **2** (0.12 mmol, 1.2 equiv), Pd(dba)_2_ (10.0 mol %), **L2** (15 mol %) and *^t^* BuONa (3.0 equiv in 0.2 mL H_2_O) in 1.0 mL toluene at 20 °C under an argon atmosphere for 72 h. Isolated yields were listed. The enantiomeric excesses were determined by HPLC;

^b)^ In 1.0 mmol scale;

^c)^ Aryl iodide was used.

The enantioselectivity varied from 85% to 97% with different ring systems in the *gem*‐diborylalkene coupling partners. Furthermore, 1,5‐diiodo‐2,6‐diisopropoxy‐naphthalene underwent double cross coupling, producing **4z** in 74% yield with 93% ee (7:1 dr) and **4aa** in 50% yield with 99% ee (>20:1 dr) respectively. The absolute configuration of **4j** was determined to be *R* by X‐ray diffraction. We also explored the product diversity of this atroposelective cross‐coupling with aryl bromides. A variety of aryl bromides and *gem*‐diborylalkenes reacted to give **5a**–**k** in 65–95% yields with 88–93% ee. Notably, the reaction tolerated aryl chloride, delivering **5l** in 94% yield with 94% ee. Iodinated tetrahydronaphthalene participated in the reaction to form product **5m** in 63% yield with 85% ee. For the substrate with a formyl group at the ortho‐position, product **5n** was isolated in 78% yield with 60% ee.

## 3 Discussion

**Scheme 3:**
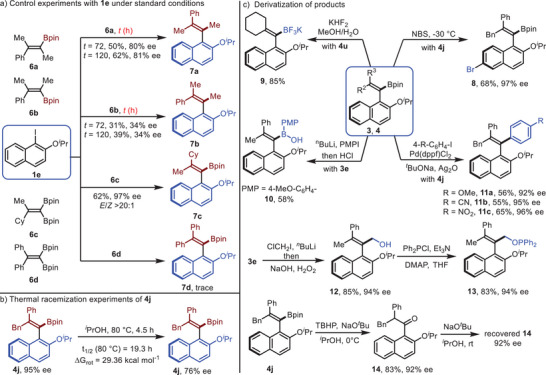
Further studies of the products.

To investigate the role of *gem*‐diboryl group in this atroposelective Suzuki–Miyaura coupling, control experiments were performed. For alkenylboronate **6a** and **6b**, inferior results were observed under the standard conditions, giving coupling products **7a** in 50% yield with 80% ee and **7b** in 31% yield with 34% ee (**Scheme** **3**a). Extending the reaction time to 120 h, slightly improved the yields of **7a** and **7b**. These results suggest that the *gem*‐diboryl group played a vital role in both the yields and the enantioselectivities of the reaction. Additionally, to investigate the origin of the chemoselectivity of this cross‐coupling, *gem*‐diboryl alkene substrates **6c** and **6d** were tested. Under the standard conditions, the corresponding product **7c** was obtained in 62% yield with 97% ee and > 20:1 *E*‐selectivity, while only a trace amount of **7d** was detected. These results indicated that the steric bulk of the alkyl and aryl substituents of diborylalkenes has a substantial effect on the chemoselectivity of the reaction, and the coupling prefers to occur at the boryl group *syn* to the smaller group. To assess the configuration stability of the products, thermal racemization experiments were conducted with compound **4j**. After treating **4j** in isopropanol at 80 °C for 4.5 h, the enantiomeric excess of **4j** decreased to 76% (Scheme 3b). Accordingly, the half‐life of racemization for compound **4j** at 80 °C is 19.3 h. The rotation barrier of **4j** was determined to be 29.36 kcal mol^−1^, which is classified into class II atropisomers (atropisomers possess a barrier to rotation, Δ*G*_rot_, between 20 and 30 kcal mol^−1^).^[^ ^14^ ^]^ The synthetic utility of the atroposelective coupling is summarized in Scheme 3c. Treating **4j** with NBS at −30 °C introduced a bromo group to the C6‐position selectively, and product **8** was obtained in 68% yield with 97% ee.^[^ ^15^ ^]^ The bromo group of **8** could serve as a handle for further transformations. Trifluoroborate salt **9** was prepared in 85% yield from vinyl pinacol boronic ester **4u**.^[^ ^16^ ^]^ Borinic acid **10** was isolated in 58% yield after treating **3e** with aryl lithium. Moreover, tetra‐substituted styrene **4j** participated in the Suzuki–Miyaura coupling with *para*‐substituted phenyl iodides,^[^ ^17^ ^]^ giving **11a**–**c** in 55–65% yields with excellent retention of enantiopurities (92–96% ee). Alkenyl boronate **3e** underwent homologation with in situ generated chloromethyl lithium, delivering allylic alcohol **12** in 85% yield with 94% ee after oxidation.^[^ ^18^ ^]^ Allyl diphenylphosphinite **13** was obtained smoothly from **12** in 83% yield with 94% ee. As an example of axial to point chirality transfer, oxidation of the alkenyl boronate unit of **4j** gave ketone **14** in 83% yield with 92% ee. It is worth noting that no erosion of the enantiopurity of recovered **14** was observed after treating **14** with NaO*^t^* Bu in *^i^* PrOH at room temperature overnight. The observed conservation of enantioselectivity is likely due to the low acidity of the H atom α to the carbonyl group induced by the stereoelectronic effect.^[^ ^19^ ^]^

## 4 Conclusion

In summary, we developed a palladium‐catalyzed atroposelective Suzuki–Miyaura coupling between *gem*‐diboryl alkenes and ArX (X = Br, I). *gem*‐Diboryl alkenes have been used as coupling partners for Suzuki–Miyaura coupling reactions to construct achiral tetra‐substituted alkenes. Our studies expanded its synthetic applications to atroposelective syntheses of tetra‐substituted acyclic styrenes. Both unlocked and locked axially chiral tetra‐substituted acyclic styrenes were synthesized in good yields with excellent enantioselectivities and chemoselectivities. The alkenyl boronate group of the axially chiral styrenes can directly undergo various transformations with excellent enantioretention.

## 5 Experimental Section

### General Procedure for the Palladium‐Catalyzed Suzuki–Miyaura Coupling

An oven‐dried 10 mL Schlenk tube was charged with Pd(dba)_2_ (5.8 mg, 0.01 mmol, 10 mol %), **L2** (5.2 mg, 0.015 mmol, 15 mol %) and toluene (0.3 mL) under argon atmosphere. The reaction mixture was stirred at room temperature for 30 minutes. Then **1** (0.1 mmol, 1.0 equiv), **2** (0.12 mmol, 1.2 equiv), *^t^* BuONa (0.3 mmol, 3.0 equiv in 0.2 mL water) and toluene (0.7 mL) was added under argon atmosphere. The reaction mixture was stirred at 20 °C (water bath) for 72 h. The reaction mixture was diluted with ethyl acetate (10.0 mL) and filtered through a plug of Celite. The filtrate was washed with water and brine, dried over anhydrous Na_2_SO_4_, and concentrated under vacuum to give yellow residue, which was purified by flash chromatography on silica gel with PE/EA to afford product. The ee value was determined by chiral phase HPLC (please see the Supporting Information for more details).

[CCDC 2293683 contains the supplementary crystallographic data for this paper. These data can be obtained free of charge from The Cambridge Crystallographic Data Centre via www.ccdc.cam.ac.uk/data_request/cif.]

## Conflict of Interest

The authors declare no conflict of interest.

## Supporting information

Supporting Information

Supporting Information

## Data Availability

The data that support the findings of this study are available in the Supporting Information of this article.
