# Supplementary material for: Palladium‐Catalyzed Atroposelective Suzuki–Miyaura Coupling to Construct Axially Chiral Tetra‐Substituted α‐Boryl Styrenes
Source: Adv Sci (Weinh). 2024 Apr 11;11(24):2309706. doi: 10.1002/advs.202309706 (PMC11199998; doi:10.1002/advs.202309706)
Supplement: Supplementary file 1 — Supporting Information [file ADVS-11-2309706-s002.pdf]

ADVANCED SCIENCE
Open Access

Supporting Information

for *Adv. Sci.*, DOI 10.1002/advs.202309706

Palladium-Catalyzed Atroposelective Suzuki–Miyaura Coupling to Construct Axially Chiral Tetra-Substituted α-Boryl Styrenes

Xiaorui Li, Lingyu Kong, Shuxin Yin, Hengrui Zhou, Aijun Lin, Hequan Yao and Shang Gao**

Supporting Information

Palladium-Catalyzed Atroposelective Suzuki-Miyaura Coupling to Construct Axially Chiral Tetra-Substituted α-Boryl Styrenes

Xiaorui Li, Lingyu Kong, Shuxin Yin, Hengrui Zhou, Aijun Lin, Hequan Yao* and Shang Gao*

State Key Laboratory of Natural Medicines (SKLNM) and Department of Medicinal Chemistry, School of Pharmacy, China Pharmaceutical University, Nanjing 210009, P. R. China

*E-mail: hyao@cpu.edu.cn; gaoshang1990@cpu.edu.cn

Contents

1. General Information *1*
2. General Procedure for the Preparation of Aryl Halides *2*
3. General Procedure for the Preparation of gem-diborylalkene *6*
4. Unsuccessful and inferior results *11*
5. General Procedure for the Pd-Catalyzed Suzuki–Miyaura coupling *11*
6. Characterization of the Products *12*
7. Scale-up Reaction *63*
8. Derivatization and Characterization of Products *64*
9. Crystal Structures of 4j *77*
10. Enantiomerization Barrier Determination of 4j *78*
11. ^{1}H, ^{13}C ^{19}F and ^{31}P NMR Spectra of Title Compounds *80*
12. References *171*

1. General Information

Reagents and Solvents: PE refers to petroleum ether b. p. 60 - 90 °C, EA refers to ethyl acetate, and DCM refers to dichloromethane. All other starting materials and solvents were commercially available and were used without further purification unless otherwise stated.

Chromatography: Flash column chromatography was carried out using commercially available 200-300 mesh under pressure unless otherwise indicated. Gradient flash chromatography was conducted eluting with PE/EA, they were listed as volume/volume ratios.

Data collection: ^1H, ^{13}C and ^{19}F NMR spectra were collected on BRUKER AV-300 (300 MHz) spectrometer using $CDCl_3$ as solvent. Chemical shifts of ^1H NMR were recorded in parts per million (ppm, δ) relative to tetramethylsilane (δ = 0.00 ppm) with the solvent resonance as an internal standard ($CDCl_3$: δ = 7.26 ppm). Data are reported as follows: chemical shift in ppm (δ), multiplicity (s = singlet, d = doublet, t = triplet, q = quartet, brs = broad singlet, m = multiplet), coupling constant (Hz), and integration. Chemical shifts of ^{13}C NMR were reported in ppm with the solvent as the internal standard ($CDCl_3$: δ = 77.16 ppm). High Resolution Mass measurement was performed on Agilent Q-TOF 6520 mass spectrometer with electron spray ionization (ESI) as the ion source. Melting point (m. p.) was measured on a microscopic melting point apparatus. Optical rotations were measured on an automatic polarimeter with $[\alpha]^{20}_D$ values reported in degrees; concentration (c) is in g/100 mL. The enantiomeric excess values were determined by chiral HPLC using an Agilent 1200 LC instrument or SHIMADZU 20AD instrument with Daicel CHIRALPAK® OD-H, OJ-H, AD-H, IA-3, IC-3 columns and Phenomenex Lux-Cellulose-1 column. X-ray diffraction analyses were carried out on a microcrystalline powder using a Rigaku Oxford Diffraction XtaLAB Synergy-S diffractometer using Cu radiation (λ = 1.54178 Å).

2. General Procedure for the Preparation of Aryl Halides

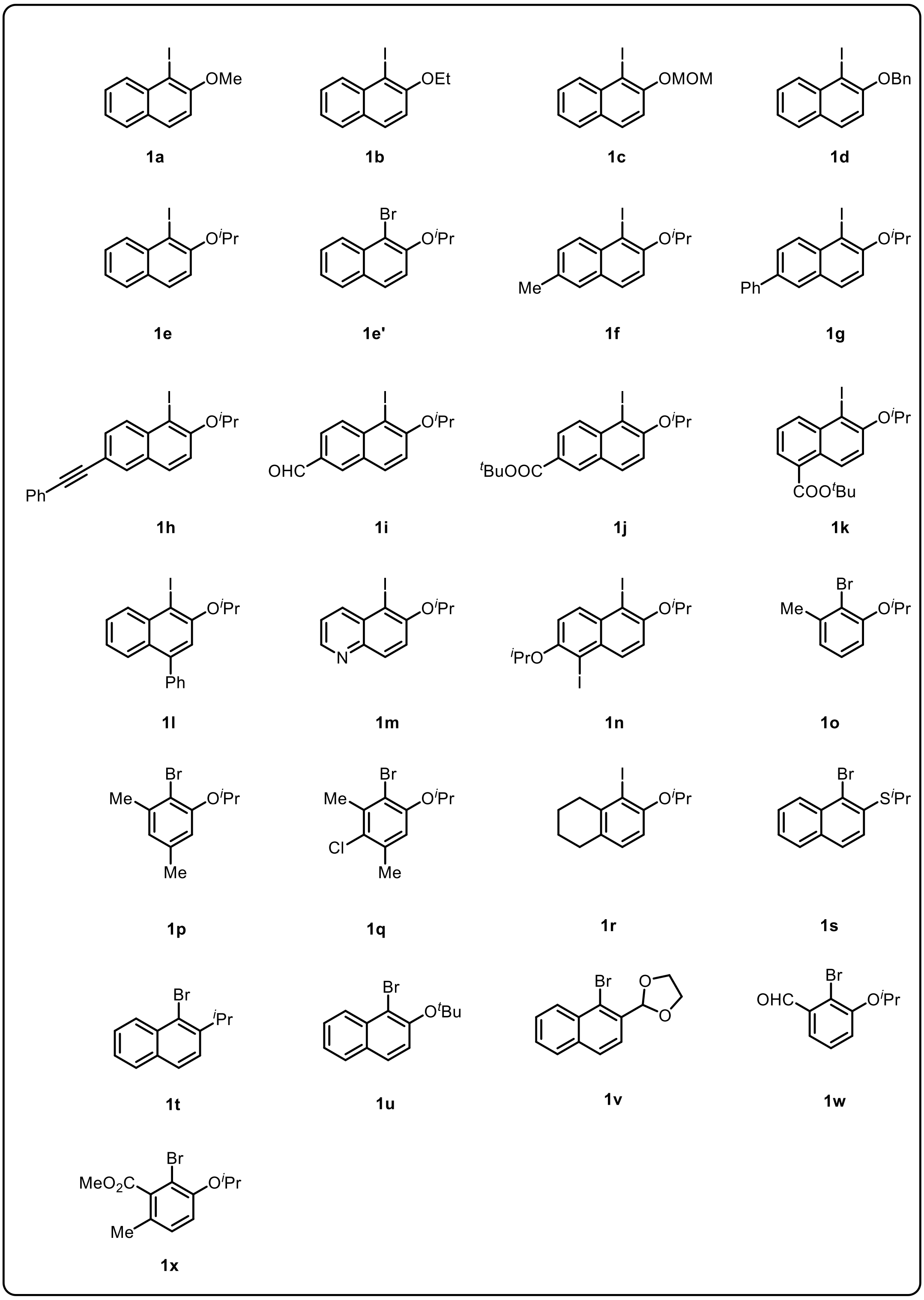

Figure S1. Structures of aryl halides

While substrates **1a** and **1p** are commercially available, substrates **1b – 1e', 1g, 1o and 1s – 1x** are known compounds and prepared according to literature procedures.[1 - 8]

For substrates **1f, 1h – 1n**, and **1r**, they are prepared according to the following procedure.

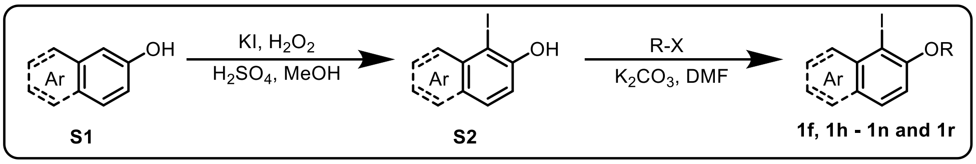

Scheme S1. General procedure for the synthesis of 1f, 1h – 1n and 1r.

Sulfuric acid (15.0 mmol, 1.5 eq.) was added to a solution of **S1** (10.0 mmol, 1.0 eq.) and potassium iodide (11.0 mmol, 1.1 eq.) in methanol (30.0 mL) at 0 °C. When the white precipitate formed, hydrogen peroxide (30% aqueous solution, 3.0 eq) was added. After 1.5 hours later, the mixture was filtered, and the filtrate was concentrated. The residue was dissolved in CH_2Cl_2, washed with $Na_2S_2O_3$ aq. and water, dried over anhydrous Na_2SO_4. Then the mixture was filtrated and the colature was evaporated on a rotary evaporator. This material was purified by flash chromatography (PE/EA = 50/1) to provide **S2**.

To a solution of **S2** (5.0 mmol, 1.0 eq.) in 10.0 mL DMF was charged with K_2CO_3 (10.0 mmol, 2.0 eq.) and alkyl halides (10.0 mmol, 2.0 eq.). The mixture was then agitated at r. t. for 12 h. The reaction was added 30.0 mL water, then extracted with EA (3 × 30.0 mL). The combined organic layers were washed with brine and dried over anhydrous Na_2SO_4. Then the mixture was filtrated and the colature was evaporated on a rotary evaporator. This material was purified by flash chromatography (PE/EA = 100/1) to provide **1f ,1h – 1n and 1r**.

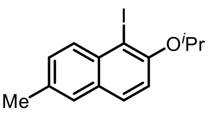

1-iodo-2-isopropoxy-6-methylnaphthalene (1f)

Prepared according to the general procedure. White solid, m. p. 46 – 48 °C, R$_f$ = 0.6 (PE/EA = 20/1). ^{1}H NMR (300 MHz, $CDCl_3$) δ 8.03 (d, J = 8.6 Hz, 1H), 7.67 (d, J = 8.9 Hz, 1H), 7.49 (s, 1H), 7.36 – 7.33 (m, 1H), 7.12 (d, J = 8.9 Hz, 1H), 4.67 (hept, J = 6.1 Hz, 1H), 2.49 (s, 3H), 1.43 (d, J = 6.1 Hz, 6H) ppm. ^{13}C NMR (75 MHz, $CDCl_3$) δ 155.0, 134.2 (two overlapping carbon signals), 131.5, 130.4, 130.2, 129.4, 127.1, 117.0, 91.2, 73.6, 22.6, 21.3 ppm. HRMS (ESI) *m/z* calcd for $[C_{14}H_{15}IO+Na]^+$ 349.0060, found 349.0055. IR (neat, cm^{-1}) 3022, 2974, 1594, 1495, 1375, 1343, 1270, 1011.

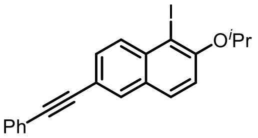

1-iodo-2-isopropoxy-6-(phenylethynyl)naphthalene (1h)

Prepared according to the general procedure. Yellow solid, m. p. 116 – 118 °C, R$_f$ = 0.6 (PE/EA = 20/1). ^{1}H NMR (300 MHz, $CDCl_3$) δ 8.11 (d, J = 8.8 Hz, 1H), 7.94 (s, 1H), 7.76 (d, J = 8.9 Hz, 1H), 7.63 – 7.56 (m, 3H), 7.38 – 7.36 (m, 3H), 7.19 (d, J = 9.0 Hz, 1H), 4.74 (hept, J = 6.0 Hz, 1H), 1.46 (d, J = 6.1 Hz, 6H) ppm. ^{13}C NMR (75 MHz, $CDCl_3$) δ 156.3, 135.5, 131.8, 131.6, 130.5, 130.0, 129.6, 128.7, 128.52, 128.45, 123.3, 119.2, 116.8, 90.7, 90.0, 89.5, 73.4, 22.6 ppm. HRMS (ESI) *m/z* calcd for $[C_{21}H_{17}IO+H]^+$ 413.0397, found 413.0387. IR (neat, cm^{-1}) 3053, 2979. 1590, 1491, 1364, 1344, 1239, 1002.

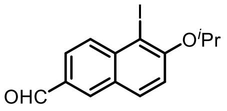

5-iodo-6-isopropoxy-2-naphthaldehyde (1i)

Prepared according to the general procedure. White solid, m. p. 104 – 105 °C, R_f = 0.5 (PE/EA = 5/1). ^{1}H NMR (300 MHz, $CDCl_3$) δ 10.12 (s, 1H), 8.24 – 8.19 (m, 2H), 7.96 – 7.90 (m, 2H), 7.23 (d, J = 8.9 Hz, 1H), 4.79 (hept, J = 6.0 Hz, 1H), 1.47 (d, J = 6.1 Hz, 6H) ppm. ^{13}C NMR (75 MHz, $CDCl_3$) δ 191.7, 158.1, 139.4, 139.2, 134.3, 132.7, 131.9, 129.0, 125.1, 116.5, 90.4, 73.4, 22.5 ppm. HRMS (ESI) *m/z* calcd for $[C_{14}H_{13}IO_2+H]^+$ 341.0033, found 341.0033. IR (neat, cm^{-1}) 3052, 2972, 1684, 1620, 1594, 1375, 1355, 1273, 1014.

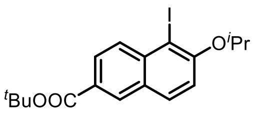

tert-butyl 5-iodo-6-isopropoxy-2-naphthoate (1j)

Prepared according to the general procedure. White solid, m. p. 88 – 90 °C, R_f = 0.5 (PE/EA = 20/1). ^{1}H NMR (300 MHz, $CDCl_3$) δ 8.41 (s, 1H), 8.14 (d, J = 8.9 Hz, 1H), 8.03 (d, J = 8.9 Hz, 1H), 7.86 (d, J = 9.0 Hz, 1H), 7.18 (d, J = 8.9 Hz, 1H), 4.75 (hept, J = 5.8 Hz, 1H), 1.64 (s, 9H), 1.45 (d, J = 6.0 Hz, 6H) ppm. ^{13}C NMR (75 MHz, $CDCl_3$) δ 165.8, 157.2, 138.1, 131.61, 131.55, 130.9, 129.0, 127.8, 127.4, 116.3, 90.2, 81.3, 73.3, 28.4, 22.6 ppm. HRMS (ESI) *m/z* calcd for $[C_{18}H_{21}IO_3+Na]^+$ 435.0428, found 435.0418. IR (neat, cm^{-1}) 3070, 2977, 1710, 1595, 1470, 1392, 1367, 1238, 1107, 1013.

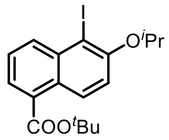

tert-butyl 5-iodo-6-isopropoxy-1-naphthoate (1k)

Prepared according to the general procedure. White solid, m. p. 84 – 86 °C, R_f = 0.5 (PE/EA = 20/1). ^{1}H NMR (300 MHz, $CDCl_3$) δ 8.82 (d, J = 9.4 Hz, 1H), 8.39 – 8.36 (m, 1H), 7.97 – 7.94 (m, 1H), 7.53 – 7.48 (m, 1H), 7.25 (d, J = 9.4 Hz, 1H), 4.75 (hept, J = 6.1 Hz, 1H), 1.66 (s, 9H), 1.45 (d, J = 6.1 Hz, 6H) ppm. ^{13}C NMR (75 MHz, $CDCl_3$) δ 166.9, 155.6, 136.3, 136.2, 129.8, 128.1, 127.9 (two overlapping carbon signals), 126.7, 117.4, 91.3, 81.9, 73.3, 28.4, 22.6 ppm. HRMS (ESI) *m/z* calcd for $[C_{18}H_{21}IO_3+Na]^+$ 435.0428, found 435.0419. IR (neat, cm^{-1}) 3031, 2967, 1700, 1653, 1507, 1340, 1262, 1182.

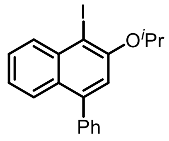

1-iodo-2-isopropoxy-4-phenylnaphthalene (1l)

Prepared according to the general procedure. White solid, m. p. 94 – 96 °C, R_f = 0.6 (PE/EA = 20/1). ^{1}H NMR (300 MHz, $CDCl_3$) δ 8.25 (d, J = 8.4 Hz, 1H), 7.73 (d, J = 8.4 Hz, 1H), 7.55 – 7.42 (m, 6H), 7.34 – 7.28 (m, 1H), 7.12 (s, 1H), 4.73 (hept, J = 6.0 Hz, 1H), 1.45 (d, J = 6.1 Hz, 6H) ppm. ^{13}C NMR (75 MHz, $CDCl_3$) δ 155.1, 142.6, 140.1, 136.1, 132.1, 130.1, 128.7, 128.5, 128.1, 127.8, 126.7, 124.8, 117.8, 90.6, 73.6, 22.7 ppm. HRMS (ESI) *m/z* calcd for $[C_{19}H_{17}IO+Na]^+$ 411.0216, found 411.0226. IR (neat, cm^{-1}) 3057, 2975, 1586, 1488, 1367, 1340, 1217, 1015, 754, 702.

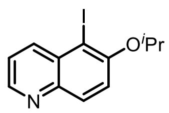

5-iodo-6-isopropoxyquinoline (1m)

Prepared according to the general procedure. Brown solid, m. p. 70 – 72 °C, R_f = 0.5 (PE/EA = 10/1). ^{1}H NMR (300 MHz, DMSO-d_6) δ 8.75 – 8.74 (m, 1H), 8.34 (d, J = 8.6 Hz, 1H), 8.02 (d, J = 9.2 Hz, 1H), 7.65 (d, J = 9.2 Hz, 1H), 7.56 (dd, J = 8.6, 4.2 Hz, 1H), 4.85 (hept, J = 6.0 Hz, 1H), 1.34 (d,

J = 6.0 Hz, 6H) ppm. ^{13}C NMR (75 MHz, DMSO-d_6) δ 155.4, 149.0, 144.4, 138.7, 131.0, 130.8, 123.3, 119.5, 89.0, 72.5, 22.2 ppm. HRMS (ESI) *m/z* calcd for $[C_{12}H_{12}INO+H]^+$ 314.0036, found 314.0043. IR (neat, cm^{-1}) 3064, 2976, 1606, 1496, 1373, 1256, 1007, 945.

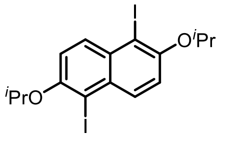

1,5-diiodo-2,6-diisopropoxynaphthalene (1n)

Prepared according to the general procedure. White solid, m. p. 164 – 166 °C, R_f = 0.6 (PE/EA = 20/1). ^{1}H NMR (300 MHz, $CDCl_3$) δ 8.16 (d, J = 9.1 Hz, 2H), 7.19 (d, J = 9.3 Hz, 2H), 4.70 (hept, J = 6.1 Hz, 2H), 1.44 (d, J = 6.1 Hz, 12H) ppm. ^{13}C NMR (75 MHz, $CDCl_3$) δ 154.8, 134.1, 131.8, 118.1, 90.8, 73.7, 22.6 ppm. HRMS (ESI) *m/z* calcd for $[C_{16}H_{18}I_2O_2+Na]^+$ 518.9288, found 518.9284. IR (neat, cm^{-1}) 3066, 2978, 1587, 1480, 1375, 1357, 1255, 1005.

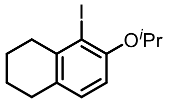

5-iodo-6-isopropoxy-1,2,3,4-tetrahydronaphthalene (1r)

Prepared according to the general procedure. White solid, m. p. 44 – 46 °C, R_f = 0.6 (PE/EA = 20/1). ^{1}H NMR (300 MHz, $CDCl_3$) δ 6.97 (d, J = 8.3 Hz, 1H), 6.63 (d, J = 8.3 Hz, 1H), 4.51 (hept, J = 6.1 Hz, 1H), 2.70 (t, J = 6.6 Hz, 4H), 1.84 – 1.67 (m, 4H), 1.38 (d, J = 6.1 Hz, 6H) ppm. ^{13}C NMR (75 MHz, $CDCl_3$) δ 154.8, 141.1, 131.9, 129.7, 112.4, 98.0, 36.9, 29.8, 24.2, 23.1, 22.4 ppm. HRMS (ESI) *m/z* calcd for $[C_{13}H_{17}IO+Na]^+$ 339.0216, found 339.0225. IR (neat, cm^{-1}) 3057, 2975, 1591, 1468, 1382, 1372, 1269, 1013.

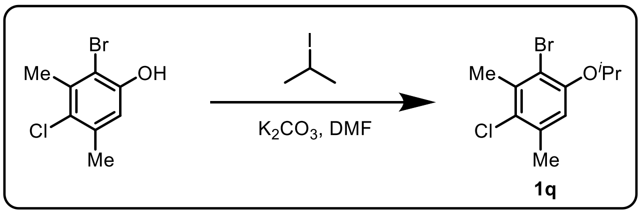

2-bromo-4-chloro-1-isopropoxy-3,5-dimethylbenzene (1q) To a solution of 2-bromo-4-chloro-3,5-dimethylphenol (1.18 g, 5.0 mmol) in 10.0 mL DMF was charged with K_2CO_3 (1.38 g, 10.0 mmol) and 2-iodopropane (1.7 g, 10.0 mmol). The mixture was then agitated at r. t. for 12 h. The reaction was added 30.0 mL water, then extracted with EA (3 × 30.0 mL). The combined organic layers were washed with brine and dried over anhydrous Na_2SO_4. Then the mixture was filtrated and the colature was evaporated on a rotary evaporator. This material was purified by flash chromatography (PE) to provide 1q. Colorless oil, R_f = 0.6 (PE). ^{1}H NMR (300 MHz, $CDCl_3$) δ 6.69 (s, 1H), 4.50 (hept, J = 6.1 Hz, 1H), 2.54 (s, 3H), 2.33 (s, 3H), 1.36 (d, J = 6.1 Hz, 6H) ppm. ^{13}C NMR (75 MHz, $CDCl_3$) δ 152.9, 137.1, 135.7, 127.1, 115.5, 114.7, 72.6, 22.2, 21.7, 21.4 ppm. HRMS (ESI) *m/z* calcd for $[C_{11}H_{14}BrClO+Na]^+$ 298.9809, found 298.9805. IR (neat, cm^{-1}) 3050, 2977, 1574, 1457, 1389, 1218, 1063, 450.

3. General Procedure for the Preparation of gem-diborylalkene

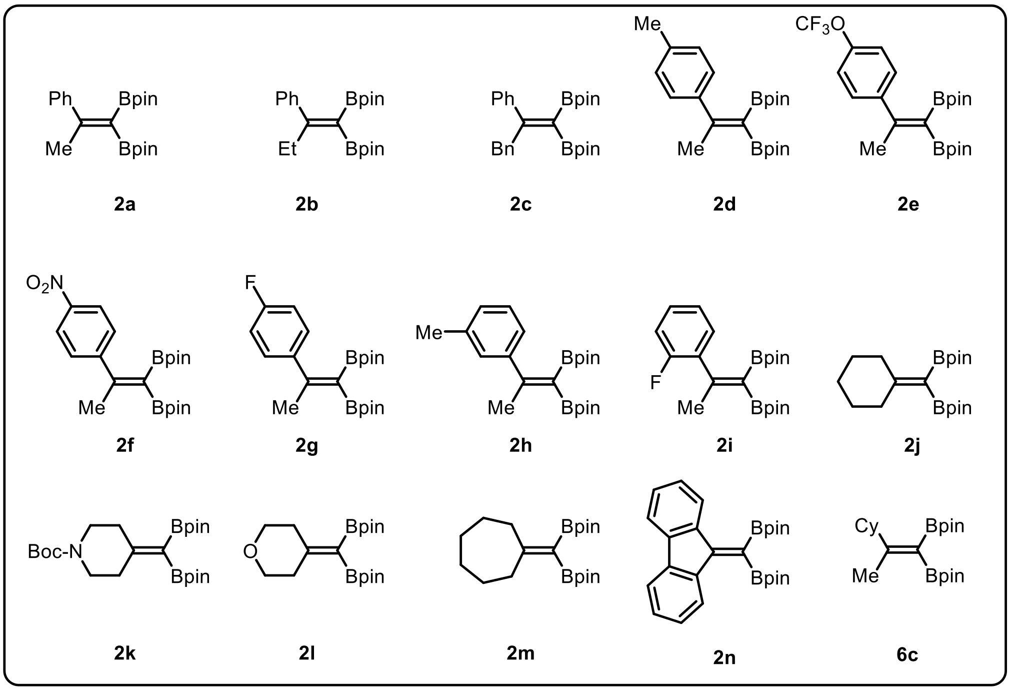

Figure S2. Structures of gem-diborylalkene

While substrates **2a, 2b and 2j** are known compounds and prepared according to literature procedures.[9, 10]

For substrates **2d – 2i, 2k – 2n and 6c**, they are prepared according to the following procedure.

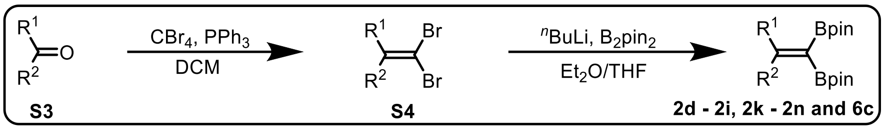

Scheme S3. General procedure for the synthesis of **2d** – **2i**, **2k** – **2n and 6c**

To a solution of PPh_3 (4.0 eq.) in CH_2Cl_2 (20.0 mL) was slowly dropped CBr_4 (2.0 eq.) in CH_2Cl_2 (6.0 mL) solution at 0 °C under argon atmosphere. After being stirred for 1 h, the ketone (6.0 mmol) in CH_2Cl_2 (6.0 mL) solution was dropped at 0 °C and the reaction mixture was stirred overnight. To the reaction mixture was added PE and then the precipitated solid was filtered. The eluent was concentrated under reduced pressure and the residue was purified by flash chromatography to give the corresponding gem-dibromoalkene derivative **S4**.

n-Butyllithium in THF (2.2 mmol) was added dropwise to a solution of **S4** (2.0 mmol) in a mixture of THF (10.0 mL) and diethyl ether (5.0 mL) at –110 °C, and the resulting solution was stirred at –110 °C for 10 min. To the resulting solution was added dropwise a solution of bis(pinacolato)diboron (2.2 mmol) in THF (10.0 mL). The whole was gradually warmed to room temperature and stirred for 12 h. The reaction mixture was quenched with NH_4Cl aq., diluted with diethyl ether (20.0 mL), and treated with water (10.0 mL). The organic layer was separated, dried over anhydrous Na_2SO_4, and concentrated to give a white solid, which was purified by flash chromatography (PE/EA = 50/1) to give **2d – 2i, 2k – 2n and 6c**.

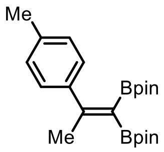

2,2'-(2-(p-tolyl)prop-1-ene-1,1-diyl)bis(4,4,5,5-tetramethyl-1,3,2-dioxaborolane) (2d) Prepared according to the general procedure. White solid, m. p. 98 – 100 °C, R_f = 0.4 (PE/EA = 10/1). ^{1}H NMR (300 MHz, $CDCl_3$) δ 7.21 (d, J = 8.0 Hz, 2H), 7.06 (d, J = 7.8 Hz, 2H), 2.33 (s, 3H), 2.31 (s, 3H), 1.28 (s, 12H), 1.09 (s, 12H) ppm. ^{13}C NMR (75 MHz, $CDCl_3$) δ 164.6, 143.9, 137.1, 128.5, 127.1, 83.1, 83.0, 25.4, 25.0, 24.6, 21.3 ppm. HRMS (ESI) *m/z* calcd for $[C_{22}H_{34}B_2O_4+H]^+$ 385.2716, found 385.2712. IR (neat, cm^{-1}) 3086, 2978, 1604, 1511, 1371, 1352, 1293, 1143, 855, 669.

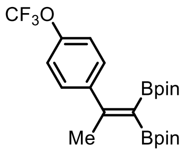

2,2'-(2-(4-(trifluoromethoxy)phenyl)prop-1-ene-1,1-diyl)bis(4,4,5,5-tetramethyl-1,3,2-dioxaborolane) (2e) Prepared according to the general procedure. White solid, m. p. 103 – 104 °C, R_f = 0.4 (PE/EA = 10/1). ^{1}H NMR (300 MHz, $CDCl_3$) δ 7.34 – 7.29 (m, 2H), 7.13 – 7.10 (m, 2H), 2.32 (s, 3H), 1.29 (s, 12H), 1.06 (s, 12H) ppm. ^{13}C NMR (75 MHz, $CDCl_3$) δ 163.1, 148.4, 145.7, 128.5, 120.6 (q, J = 256.7 Hz), 120.5, 83.20, 83.18, 25.6, 24.9, 24.5 ppm. ^{19}F NMR (282 MHz, $CDCl_3$) δ -57.99 ppm. HRMS (ESI) *m/z* calcd for $[C_{22}H_{31}B_2F_3O_5+H]^+$ 455.2382, found 455.2392. IR (neat, cm^{-1}) 3069, 2931, 1605, 1498, 1380, 1372, 1260, 1147, 855, 669.

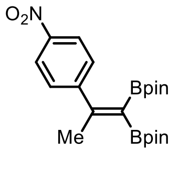

2,2'-(2-(4-nitrophenyl)prop-1-ene-1,1-diyl)bis(4,4,5,5-tetramethyl-1,3,2-dioxaborolane) (2f) Prepared according to the general procedure. Yellow solid, m. p. 123 – 125 °C, R_f = 0.4 (PE/EA = 10/1). ^{1}H NMR (300 MHz, $CDCl_3$) δ 8.14 (d, J = 8.7 Hz, 2H), 7.44 (d, J = 8.7 Hz, 2H), 2.33 (s, 3H), 1.31 (s, 12H), 1.06 (s, 12H) ppm. ^{13}C NMR (75 MHz, $CDCl_3$) δ 161.5, 153.3, 147.0, 128.1, 123.3, 83.49, 83.46, 25.4, 25.0, 24.6 ppm. HRMS (ESI) *m/z* calcd for $[C_{21}H_{31}B_2NO_6+H]^+$ 416.2410, found 416.2415. IR (neat, cm^{-1}) 3078, 2978, 1592, 1519, 1346, 1142, 855, 702.

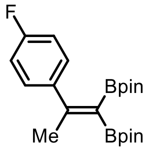

2,2'-(2-(4-fluorophenyl)prop-1-ene-1,1-diyl)bis(4,4,5,5-tetramethyl-1,3,2-dioxaborolane) (2g) Prepared according to the general procedure. White solid, m. p. 103 – 105 °C, R_f = 0.4 (PE/EA = 10/1). ^{1}H NMR (300 MHz, $CDCl_3$) δ 7.30 – 7.25 (m, 2H), 6.97 – 6.91 (m, 2H), 2.31 (s, 3H), 1.29 (s, 12H), 1.08 (s, 12H) ppm. ^{13}C NMR (75 MHz, $CDCl_3$) δ 163.4, 162.2 (d, $^{1}J_{C\text{-}F}$ = 245.2 Hz), 142.8 (d, $^{4}J_{C\text{-}F}$ = 2.6 Hz), 128.8 (d, $^{3}J_{C\text{-}F}$ = 7.9 Hz), 114.6 (d, $^{2}J_{C\text{-}F}$ = 21.3 Hz), 83.2, 83.1, 25.6, 25.0, 24.6 ppm. ^{19}F NMR (282 MHz, $CDCl_3$) δ -115.53 ppm. HRMS (ESI) *m/z* calcd for $[C_{21}H_{31}B_2FO_4+H]^+$ 389.2465, found 389.2468. IR (neat, cm^{-1}) 3072, 2978, 1610, 1507, 1372, 1353, 1143, 855, 716.

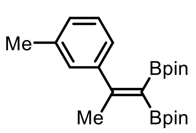

2,2'-(2-(m-tolyl)prop-1-ene-1,1-diyl)bis(4,4,5,5-tetramethyl-1,3,2-dioxaborolane) (2h) Prepared according to the general procedure. White solid, m. p. 54 – 56 °C, R_f = 0.4 (PE/EA = 10/1). ^{1}H NMR (300 MHz, $CDCl_3$) δ 7.18 – 7.09 (m, 3H), 7.05 – 7.02 (m, 1H), 2.33 (s, 3H), 2.31 (s, 3H), 1.28 (s, 12H), 1.07 (s, 12H) ppm. ^{13}C NMR (75 MHz, $CDCl_3$) δ 165.0, 146.9, 137.2, 128.1, 127.9, 127.8, 124.1, 83.1, 83.0, 25.4, 25.0, 24.5, 21.5 ppm. HRMS (ESI) *m/z* calcd for $[C_{22}H_{34}B_2O_4+H]^+$ 385.2716, found 385.2713. IR (neat, cm^{-1}) 3059, 2978, 1600, 1482, 1379, 1371, 1143, 856, 705.

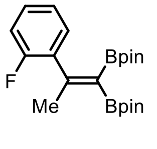

2,2'-(2-(2-fluorophenyl)prop-1-ene-1,1-diyl)bis(4,4,5,5-tetramethyl-1,3,2-dioxaborolane) (2i) Prepared according to the general procedure. White solid, m. p. 82 – 84 °C, R_f = 0.4 (PE/EA = 10/1). ^{1}H NMR (300 MHz, $CDCl_3$) δ 7.22 – 7.16 (m, 2H), 7.04 – 6.94 (m, 2H), 2.30 (s, 3H), 1.29 (s, 12H), 1.00 (s, 12H) ppm. ^{13}C NMR (75 MHz, $CDCl_3$) δ 159.7, 159.1 (d, $^{1}J_{C\text{-}F}$ = 246.4 Hz), 134.3 (d, $^{2}J_{C\text{-}F}$ = 16.7 Hz), 130.1 (d, $^{3}J_{C\text{-}F}$ = 4.5 Hz), 128.6 (d, $^{3}J_{C\text{-}F}$ = 8.1 Hz), 123.5 (d, $^{4}J_{C\text{-}F}$ = 3.5 Hz), 115.3 (d, $^{2}J_{C\text{-}F}$ = 22.2 Hz), 83.2, 83.0, 77.6, 77.2, 76.7, 25.6, 25.0, 24.4 ppm. ^{19}F NMR (282 MHz, $CDCl_3$) δ -115.08 ppm. HRMS (ESI) *m/z* calcd for $[C_{21}H_{31}B_2FO_4+H]^+$ 389.2465, found 389.2466. IR (neat, cm^{-1}) 3071, 2980, 2930, 1606, 1487, 1371, 1360, 1141.

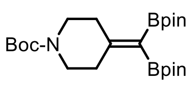

tert-butyl-4-(bis(4,4,5,5-tetramethyl-1,3,2-dioxaborolan-2-yl)methylene)piperidine-1-carboxylate (2k) Prepared according to the general procedure. White solid, m. p. 140 – 142 °C, R_f = 0.3 (PE/EA = 10/1). ^{1}H NMR (300 MHz, $CDCl_3$) δ 3.47 – 3.43 (m, 4H), 2.49 – 2.45 (m, 4H), 1.46 (s, 9H), 1.26 (s, 24H) ppm. ^{13}C NMR (75 MHz, $CDCl_3$) δ 165.7, 154.6, 82.9, 79.2, 45.0,

36.1, 28.3, 24.6 ppm. HRMS (ESI) *m/z* calcd for $[C_{23}H_{41}B_2NO_6+H]^+$ 450.3193, found 450.3196. IR (neat, cm^{-1}) 3071, 2980, 2930, 1606, 1487, 1371, 1360, 1141.

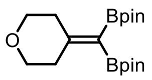

2,2'-((tetrahydro-4H-pyran-4-ylidene)methylene)bis(4,4,5,5-tetramethyl-1,3,2-dioxaborolane) (2l) Prepared according to the general procedure. White solid, m. p. 110 – 114 °C, R_f = 0.4 (PE/EA = 10/1). ^1H NMR (300 MHz, $CDCl_3$) δ 3.73 (t, *J* = 5.5 Hz, 4H), 2.54 (t, *J* = 5.5 Hz, 4H), 1.26 (s, 24H) ppm. ^{13}C NMR (75 MHz, $CDCl_3$) δ 165.4, 83.1, 69.5, 37.8, 24.9 ppm. HRMS (ESI) *m/z* calcd for $[C_{18}H_{32}B_2O_5+H]^+$ 351.2509, found 351.2508. IR (neat, cm^{-1}) 2976, 1620, 1378, 1359, 1286, 1142.

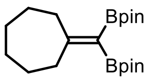

2,2'-(cycloheptylidenemethylene)bis(4,4,5,5-tetramethyl-1,3,2-dioxaborolane) (2m) Prepared according to the general procedure. White solid, m. p. 48 – 50 °C, R_f = 0.4 (PE/EA = 10/1). ^1H NMR (300 MHz, $CDCl_3$) δ 2.53 – 2.49 (m, 4H), 1.60 – 1.50 (m, 8H), 1.25 (s, 24H) ppm. ^{13}C NMR (75 MHz, $CDCl_3$) δ 173.9, 82.7, 38.1, 29.2, 28.0, 24.8 ppm. HRMS (ESI) *m/z* calcd for $[C_{20}H_{36}B_2O_4+H]^+$ 363.2872, found 363.2879. IR (neat, cm^{-1}) 2926, 2854, 1403, 1397, 1156, 668.

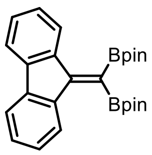

2,2'-((9H-fluoren-9-ylidene)methylene)bis(4,4,5,5-tetramethyl-1,3,2-dioxaborolane) (2n) Prepared according to the general procedure. Yellow solid, m. p. 164 – 166 °C, R_f = 0.4 (PE/EA = 10/1). ^1H NMR (300 MHz, $CDCl_3$) δ 8.04 (d, *J* = 7.7 Hz, 2H), 7.58 (d, *J* = 7.4 Hz, 2H), 7.29 (t, *J* = 7.4 Hz, 2H), 7.19 (t, *J* = 7.5 Hz, 2H), 1.41 (s, 24H) ppm. ^{13}C NMR (75 MHz, $CDCl_3$) δ 154.4, 141.1, 139.6, 129.0, 126.7, 123.9, 119.3, 84.1, 25.1 ppm. HRMS (ESI) *m/z* calcd for $[C_{26}H_{32}B_2O_4+H]^+$ 431.2559, found 431.2568. IR (neat, cm^{-1}) 3062, 2979, 1600, 1478, 1379, 1331, 1143.

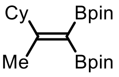

2,2'-(2-cyclohexylprop-1-ene-1,1-diyl)bis(4,4,5,5-tetramethyl-1,3,2-dioxaborolane) (6c) Prepared according to the general procedure. White solid, m. p. 94 – 96 °C, R_f = 0.4 (PE/EA = 10/1). ^1H NMR (300 MHz, $CDCl_3$) δ 2.48 – 2.40 (m, 1H), 1.88 (s, 3H), 1.75 – 1.71 (m, 2H), 1.65 – 1.61 (m, 2H), 1.31 – 1.17 (m, 30H) ppm. ^{13}C NMR (75 MHz, $CDCl_3$) δ 172.3, 82.9, 82.8, 49.8, 31.6, 26.6, 26.3, 24.9, 24.8, 19.5 ppm. HRMS (ESI) *m/z* calcd for $[C_{21}H_{38}B_2O_4+H]^+$ 377.3029, found 377.3032. IR (neat, cm^{-1}) 2979, 2853, 1608, 1380, 1370, 1352, 1139.

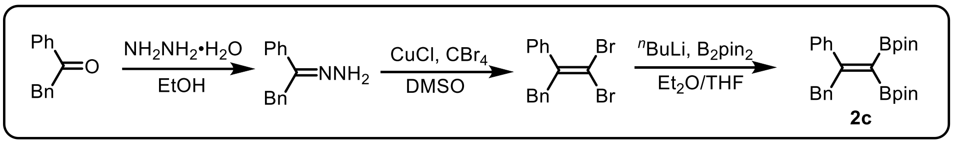

A 100 mL three-necked flask was charged with 1,2-diphenylethan-1-one (3.92 g, 20.0 mmol), hydrazine monohydrate (60.0 mmol), ethanol (40.0 mL). The reaction mixture was then refluxed for 4 h. After cooled to room temperature, the reaction mixture was extracted with DCM (50.0 mL×3), and dried over anhydrous Na_2SO_4 and

concentrated under vacuum. The hydrazone was obtained in quantitative yield, and used directly for the next step without further purification.

A 25% aqueous solution of ammonia (6.6 mL) and CuCl (200 mg, 2.0 mmol) were added to a solution of freshly prepared hydrazine in DMSO (20.0 mL). CBr_4 (19.9 g, 60.0 mmol) in DMSO (50.0 mL) was added into the flask under the ice bath after 10 min. The reaction mixture was stirred for 24 h at room temperature and then quenched with water (20.0 mL). The reaction mixture was extracted with DCM (20.0 mL × 3). The extracts were dried over anhydrous Na_2SO_4 and filtered off, and the solvent was removed by evaporation under vacuum. The residue was purified by flash chromatography to give colorless transparent liquid (4.2 g, 60%).

n-Butyllithium in THF (2.2 mmol) was added dropwise to a solution of **S4** (2.0 mmol) in a mixture of THF (10.0 mL) and diethyl ether (5.0 mL) at –110 °C, and the resulting solution was stirred at –110 °C for 10 min. To the resulting solution was added dropwise a solution of bis(pinacolato)diboron (2.2 mmol) in THF (10.0 mL). The whole was gradually warmed to room temperature and stirred for 12 h. The reaction mixture was quenched with NH_4Cl aq., diluted with diethyl ether (20.0 mL), and treated with water (10.0 mL). The organic layer was separated, dried over anhydrous Na_2SO_4, and concentrated to give a white solid, which was purified by flash chromatography (PE/EA = 50/1) to give **2c**.

2,2'-(2,3-diphenylprop-1-ene-1,1-diyl)bis(4,4,5,5-tetramethyl-1,3,2-dioxaborolane) (2c) White solid, m. p. 74 – 76 °C, R_f = 0.4 (PE/EA = 10/1). ^{1}H NMR (300 MHz, $CDCl_3$) δ 7.16 – 7.05 (m, 10H), 4.08 (s, 2H), 1.29 (s, 12H), 1.03 (s, 12H) ppm. ^{13}C NMR (75 MHz, $CDCl_3$) δ 166.8, 145.0, 139.5, 129.3, 128.0, 127.9, 127.6, 127.1, 125.7, 83.2, 44.8, 25.0, 24.5 ppm. HRMS (ESI) *m/z* calcd for $[C_{27}H_{36}B_2O_4+H]^+$ 447.2872, found 447.2870. IR (neat, cm^{-1}) 3054, 2975, 1594, 1494, 1370, 1353, 1292, 1141, 1063, 759, 701.

4. Unsuccessful and inferior results

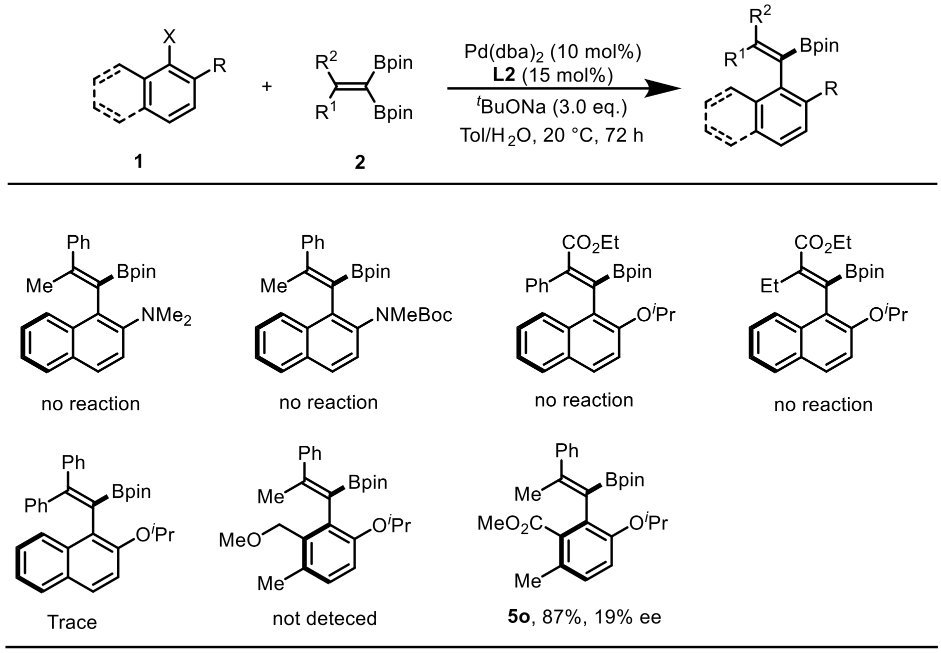

Scheme S4. Unsuccessful and inferior results

Reaction conditions: **1** (0.1 mmol, 1.0 equiv), **2** (0.12 mmol, 1.2 equiv), Pd(dba)$_2$ (10 mol %), **L2** (15 mol %) and ^{t}BuONa (3.0 eq. in 0.2 mL H_2O) in 1.0 mL toluene at 20 ℃ under an argon atmosphere for 72 h.

5. General Procedure for the Pd-Catalyzed Suzuki–Miyaura coupling

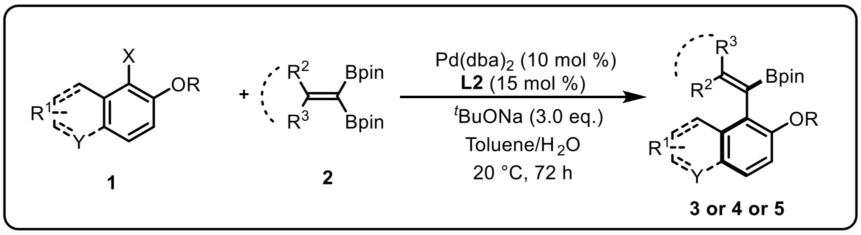

An oven-dried 10 mL Schlenk tube was charged with Pd(dba)$_2$ (5.8 mg, 0.01 mmol, 10 mol %), **L2** (5.2 mg, 0.015 mmol, 15 mol %) and toluene (0.3 mL) under argon atmosphere. The reaction mixture was stirred at r. t. for 30 minutes. Then **1** (0.1 mmol, 1.0 eq.), **2** (0.12 mmol, 1.2 eq.), ^{t}BuONa (0.3 mmol, 3.0 eq. in 0.2 mL water) and toluene (0.7 mL) was added under argon atmosphere. The reaction mixture was stirred at 20 °C (water temperature) for 72 h. The reaction mixture was diluted with EA (10.0 mL) and filtered through a plug of Celite. The filtrate was washed with water and brine, dried over anhydrous Na_2SO_4, and concentrated under vacuum to give yellow residue, which was purified by flash chromatography on silica gel with PE/EA to afford product. The ee value was determined by chiral phase HPLC.

6. Characterization of the Products

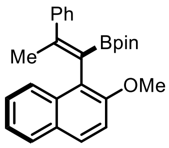

(R,E)-2-(1-(2-methoxynaphthalen-1-yl)-2-phenylprop-1-en-1-yl)-4,4,5,5-tetramethyl-1,3,2-dioxaborolane (3a)

Prepared according to the general procedure. 36.1 mg, 90% yield, 86% ee; R_f = 0.4 (PE/EA = 20/1); colorless oil, $[\alpha]^{20}_D$ = +157.1 (c = 0.028, $CHCl_3$). ^1H NMR (300 MHz, $CDCl_3$) δ 7.91 (d, J = 8.5 Hz, 1H), 7.80 – 7.76 (m, 2H), 7.54 – 7.51 (m, 2H), 7.43 – 7.28 (m, 6H), 3.91 (s, 3H), 1.79 (s, 3H), 1.04 (s, 6H), 0.99 (s, 6H) ppm. ^{13}C NMR (75 MHz, $CDCl_3$) δ 153.9, 152.4, 145.3, 132.7, 129.5, 128.4, 128.2, 128.1, 128.0, 127.4, 126.2, 125.6, 125.5, 123.3, 113.6, 83.0, 56.6, 24.7, 24.4, 22.9 ppm. HRMS (ESI) *m/z* calcd for $[C_{26}H_{29}BO_3+H]^+$ 401.2283, found 401.2286. IR (neat, cm^{-1}) 3059, 2977, 1591, 1508, 1328, 1267, 1146, 1092, 748, 700. HPLC: Daicel Chiralcel OJ-H, n-hexane/isopropanol 95/5, flow rate = 0.5 mL/min, uv-vis λ = 230 nm, t_{R1} = 8.9 (minor), t_{R2} = 13.5 (major).

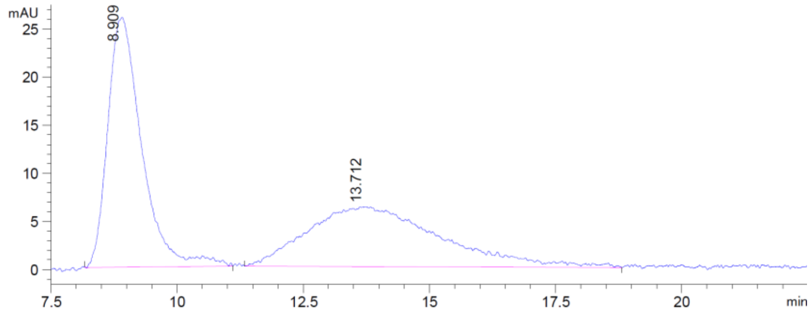

Signal 2: MWD1 B, Sig=230,4 Ref=off

Peak #	RT [min]	Type	Height	Width [min]	Area %	Area
1	8.909	MM	25.942	0.781	51.665	1.215e3
2	13.712	MM	6.199	3.057	48.335	1.137e3

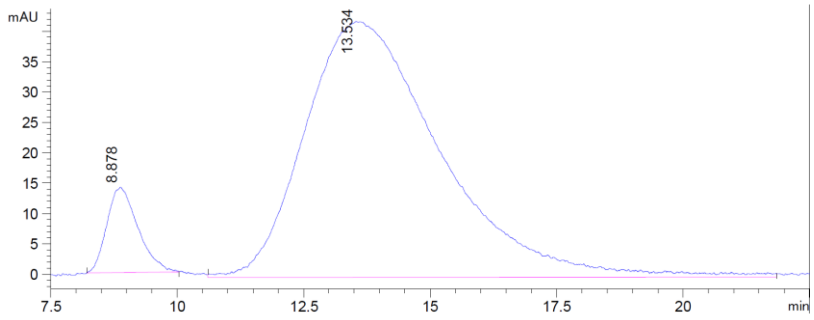

Signal 2: MWD1 B, Sig=230,4 Ref=off

Peak #	RT [min]	Type	Height	Width [min]	Area %	Area
1	8.878	MM	14.102	0.712	7.148	602.462
2	13.534	MM	42.117	3.097	92.852	7.826e3

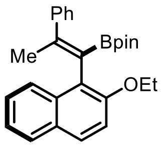

(R,E)-2-(1-(2-ethoxynaphthalen-1-yl)-2-phenylprop-1-en-1-yl)-4,4,5,5-tetramethyl-1,3,2-dioxaborolane (3b)

Prepared according to the general procedure. 33.7 mg, 81% yield, 86% ee; R_f = 0.4 (PE/EA = 20/1); colorless oil, $[\alpha]^{20}_{D}$ = +38.9 (c = 0.036, $CHCl_3$). ^{1}H NMR (300 MHz, $CDCl_3$) δ 7.92 (d, J = 8.5 Hz, 1H), 7.80 – 7.77 (m, 1H), 7.74 (d, J = 8.9 Hz, 1H), 7.51 – 7.47 (m, 2H), 7.43 – 7.27 (m, 6H), 4.17 (q, J = 7.0 Hz, 2H), 1.80 (s, 3H), 1.41 (t, J = 7.0 Hz, 3H), 1.00 (s, 6H), 0.98 (s, 6H) ppm. ^{13}C NMR (75 MHz, $CDCl_3$) δ 153.1, 152.2, 145.5, 133.1, 129.6, 128.3, 128.1, 128.0, 127.9, 127.3, 126.8, 126.0, 125.5, 123.5, 116.3, 83.0, 65.6, 24.6, 24.5, 22.8, 15.4 ppm. HRMS (ESI) *m/z* calcd for $[C_{27}H_{31}BO_3+Na]^+$ 437.2258, found 437.2258. IR (neat, cm^{-1}) 3056, 2929, 1591, 1508, 1371, 1342, 1265, 1145, 747, 700. HPLC: Daicel Chiralcel IC-3, n-hexane/isopropanol 98/2, flow rate = 0.5 mL/min, uv-vis λ = 230 nm, t_{R1} = 7.2 min (minor), t_{R2} = 7.8 min (major).

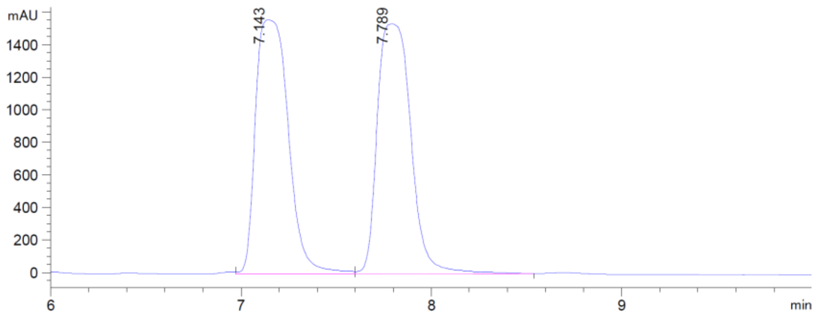

Signal 2: MWD1 B, Sig=230,4 Ref=off

Peak #	RT [min]	Type	Height	Width [min]	Area %	Area
1	7.143	VV	1.562e3	0.192	49.902	1.876e4
2	7.789	VB	1.537e3	0.197	50.098	1.883e4

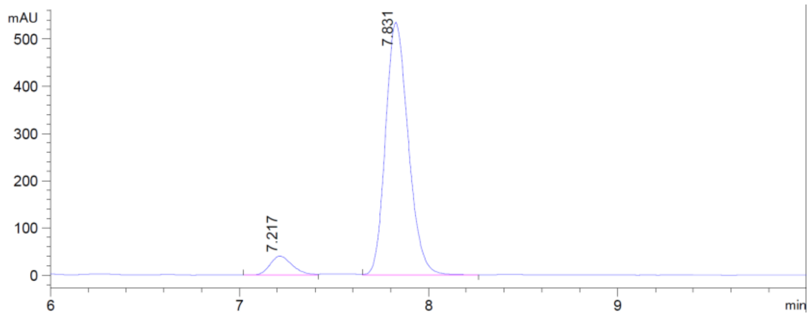

Signal 2: MWD1 B, Sig=230,4 Ref=off

Peak #	RT [min]	Type	Height	Width [min]	Area %	Area
1	7.217	BV	39.626	0.128	6.804	325.540
2	7.831	VB	533.913	0.129	93.196	4.459e3

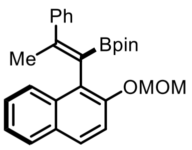

(R,E)-2-(1-(2-(methoxymethoxy)naphthalen-1-yl)-2-phenylprop-1-en-1-yl)-4,4,5,5-tetramethyl-1,3,2-dioxaborolane (3c)

Prepared according to the general procedure. 35.2 mg, 82% yield, 85% ee; R_f = 0.4 (PE/EA = 10/1); colorless oil, $[\alpha]^{20}_{D}$ = +93.8 (c = 0.048, $CHCl_3$). ^1H NMR (300 MHz, $CDCl_3$) δ 7.94 (d, J = 8.3 Hz, 1H), 7.81 – 7.78 (m, 1H), 7.74 (d, J = 9.0 Hz, 1H), 7.52 – 7.29 (m, 8H), 5.30 (d, J = 6.6 Hz, 1H), 5.18 (d, J = 6.6 Hz, 1H), 3.55 (s, 3H), 1.81 (s, 3H), 0.99 (s, 12H) ppm. ^{13}C NMR (75 MHz, $CDCl_3$) δ 152.3, 151.6, 145.2, 132.9, 130.3, 128.20, 128.17, 128.1, 128.0, 127.6, 127.4, 126.1, 125.7, 124.0, 118.1, 96.1, 83.1, 56.3, 24.6, 24.5, 22.6 ppm. HRMS (ESI) *m/z* calcd for $[C_{27}H_{31}BO_4+H]^+$ 431.2388, found 431.2386. IR (neat, cm^{-1}) 3056, 2978, 1592, 1507, 1379, 1343, 1240, 1146, 1036, 749, 701. HPLC: Daicel Chiralcel IC-3, n-hexane/isopropanol 98/2, flow rate = 0.5 mL/min, uv-vis λ = 230 nm, t_{R1} = 7.8 min (minor), t_{R2} = 8.9 min (major).

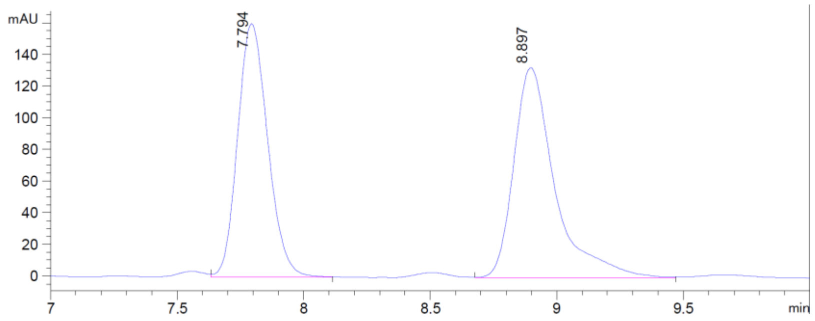

Signal 2: MWD1 B, Sig=230,4 Ref=off

Peak #	RT [min]	Type	Height	Width [min]	Area %	Area
1	7.794	VV	160.253	0.129	46.991	1.326e3
2	8.897	VV	132.775	0.167	53.009	1.496e3

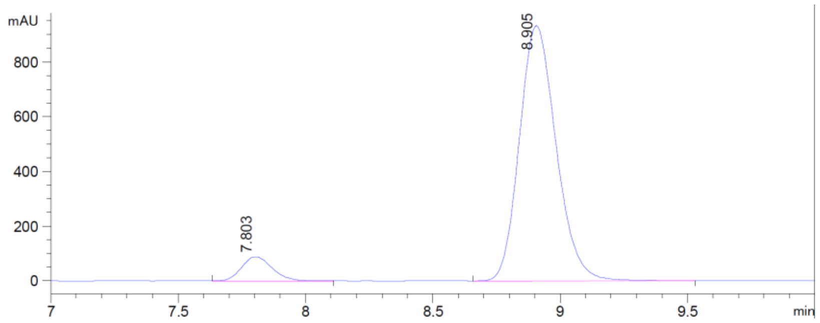

Signal 2: MWD1 B, Sig=230,4 Ref=off

Peak #	RT [min]	Type	Height	Width [min]	Area %	Area
1	7.803	VV	88.637	0.129	7.280	734.944
2	8.905	BV	933.637	0.155	92.720	9.360e3

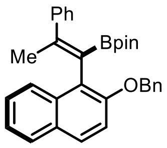

(R,E)-2-(1-(2-(benzyloxy)naphthalen-1-yl)-2-phenylprop-1-en-1-yl)-4,4,5,5-tetramethyl-1,3,2-dioxaborolane (3d)

Prepared according to the general procedure. 33.7 mg, 71% yield, 87% ee; R_f = 0.4 (PE/EA = 20/1); colorless oil, $[\alpha]^{20}_{D}$ = +83.3 (c = 0.018, $CHCl_3$). ^{1}H NMR (300 MHz, $CDCl_3$) δ 7.96 (d, J = 8.4 Hz, 1H), 7.78 (d, J = 7.9 Hz, 1H), 7.71 (d, J = 8.9 Hz, 1H), 7.50 – 7.44 (m, 4H), 7.42 – 7.23 (m, 9H), 5.23 (s, 2H), 1.81 (s, 3H), 0.98 (s, 6H), 0.94 (s, 6H) ppm. ^{13}C NMR (75 MHz, $CDCl_3$) δ 152.7, 152.6, 145.5, 138.2, 133.1, 129.8, 128.4, 128.2, 128.1, 127.95, 127.86, 127.5, 127.3 (two overlapping carbon signals), 127.1, 126.1, 125.6, 123.7, 116.6, 83.0, 71.8, 24.6, 24.5, 22.8 ppm. IR (neat, cm^{-1}) 3058, 2977, 1591, 1507, 1378, 1343, 1266, 1145, 1010, 746, 699. HRMS (ESI) m/z calcd for $[C_{32}H_{33}BO_3+H]^+$ 477.2596, found 477.2597. HPLC: Daicel Chiralcel IA-3, n-hexane/isopropanol 99/1, flow rate = 0.5 mL/min, uv-vis λ = 230 nm, t_{R1} = 8.8 min (minor), t_{R2} = 9.3 min (major).

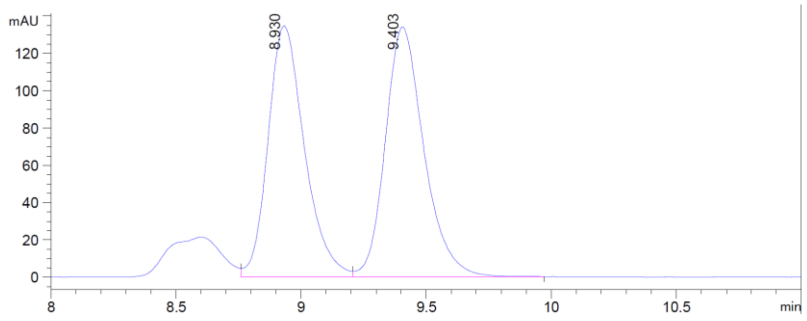

Signal 2: MWD1 B, Sig=230,4 Ref=off

Peak #	RT [min]	Type	Height	Width [min]	Area %	Area
1	8.930	VV	134.623	0.154	48.632	1.360e3
2	9.403	VB	133.931	0.163	51.368	1.436e3

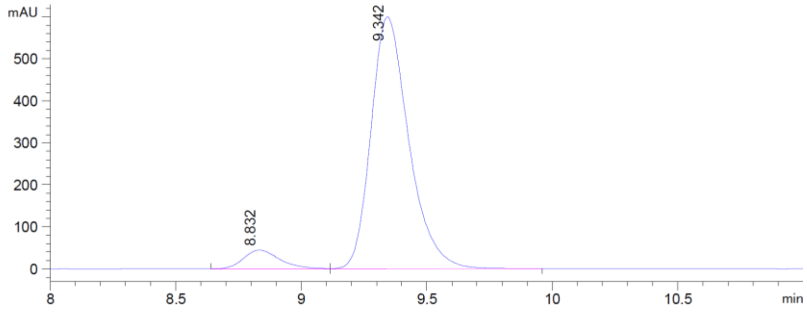

Signal 2: MWD1 B, Sig=230,4 Ref=off

Peak #	RT [min]	Type	Height	Width [min]	Area %	Area
1	8.832	VV	44.742	0.153	6.661	449.682
2	9.342	VV	600.436	0.158	93.339	6.301e3

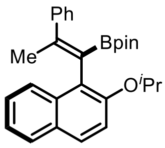

(R,E)-2-(1-(2-isopropoxynaphthalen-1-yl)-2-phenylprop-1-en-1-yl)-4,4,5,5-tetramethyl-1,3,2-dioxaborolane (3e)

Prepared according to the general procedure. 36.1 mg, 84% yield, 94% ee; R_f = 0.4 (PE/EA = 20/1); colorless oil, $[\alpha]^{20}_{D}$ = +47.7 (c = 0.044, $CHCl_3$). ^{1}H NMR (300 MHz, $CDCl_3$) δ 7.93 – 7.90 (m, 1H), 7.80 – 7.77 (m, 1H), 7.72 (d, J = 8.9 Hz, 1H), 7.48 – 7.44 (m, 2H), 7.43 – 7.29 (m, 5H), 7.25 – 7.24 (m, 1H), 4.58 (hept, J = 6.0 Hz, 1H), 1.80 (s, 3H), 1.36 (d, J = 6.1 Hz, 3H), 1.31 (d, J = 6.0 Hz, 3H), 0.99 (s, 6H), 0.97 (s, 6H) ppm. ^{13}C NMR (75 MHz, $CDCl_3$) δ 152.22, 152.16, 145.7, 133.2, 130.0, 128.9, 128.2, 128.1, 128.0, 127.6, 127.2, 125.9, 125.7, 123.7, 119.4, 82.9, 73.0, 24.7, 24.5, 23.0, 22.8, 22.5 ppm. HRMS (ESI) *m/z* calcd for $[C_{28}H_{33}BO_3+Na]^+$ 451.2415, found 451.2421. IR (neat, cm^{-1}) 3058, 2929, 1591, 1506, 1379, 1340, 1264, 1145, 1112, 749, 700. HPLC: Daicel Chiralcel IC-3, n-hexane/isopropanol 98/2, flow rate = 0.5 mL/min, uv-vis λ = 230 nm, t_{R1} = 6.9 min (minor), t_{R2} = 7.5 min (major).

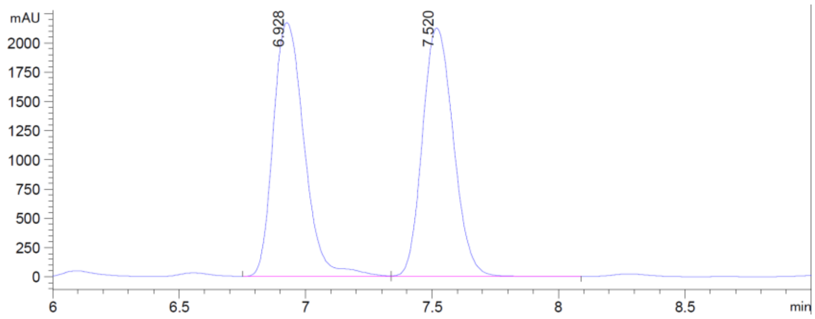

Signal 2: MWD1 B, Sig=230,4 Ref=off

Peak #	RT [min]	Type	Height	Width [min]	Area %	Area
1	6.928	VV	2.172e3	0.134	50.605	1.856e4
2	7.520	VB	2.128e3	0.133	49.395	1.811e4

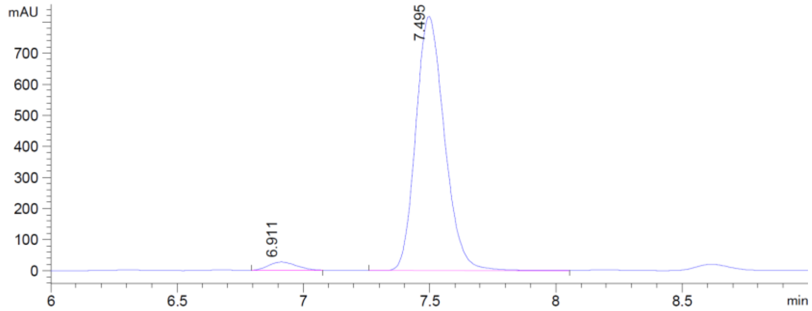

Signal 2: MWD1 B, Sig=230,4 Ref=off

Peak #	RT [min]	Type	Height	Width [min]	Area %	Area
1	6.911	MM	27.023	0.126	3.073	204.934
2	7.495	BV	817.584	0.122	96.927	6.463e3

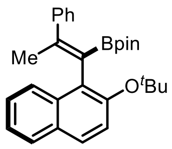

(R,E)-2-(1-(2-(tert-butoxy)naphthalen-1-yl)-2-phenylprop-1-en-1-yl)-4,4,5,5-tetramethyl-1,3,2-dioxaborolane (3f)

Prepared according to the general procedure. 33.4 mg, 76% yield, 84% ee; R_f = 0.4 (PE/EA = 20/1); white solid, m.p.132 – 134 °C , $[\alpha]^{20}_{D}$ = +100.9 (c = 0.038, $CHCl_3$). ^{1}H NMR (300 MHz, $CDCl_3$) δ 7.92 (d, J = 8.2 Hz, 1H), 7.79 (d, J = 8.0 Hz, 1H), 7.67 (d, J = 8.7 Hz, 1H), 7.46 – 7.40 (m, 3H), 7.38 – 7.24 (m, 5H), 1.80 (s, 3H), 1.43 (s, 9H), 1.02 (s, 6H), 0.98 (s, 6H) ppm. ^{13}C NMR (75 MHz, $CDCl_3$) δ 151.9, 151.0, 145.7, 132.8, 132.7, 130.7, 128.14, 128.08, 128.0, 127.1, 127.0, 126.3, 125.8, 124.1, 124.0, 82.9, 80.2, 29.6, 25.0, 24.3, 23.8 ppm. HRMS (ESI) *m/z* calcd for $[C_{29}H_{35}BO_3+H]^+$ 443.2752, found 443.2752. IR (neat, cm^{-1}) 3055, 2977, 1650, 1591, 1389, 1379, 1165, 1145, 1102, 765, 699. HPLC: Daicel Chiralcel IC-3, n-hexane/isopropanol 99.7/0.3, flow rate = 0.5 mL/min, uv-vis λ = 230 nm, t_{R1} = 9.4 min (minor), t_{R2} = 12.0 min (major).

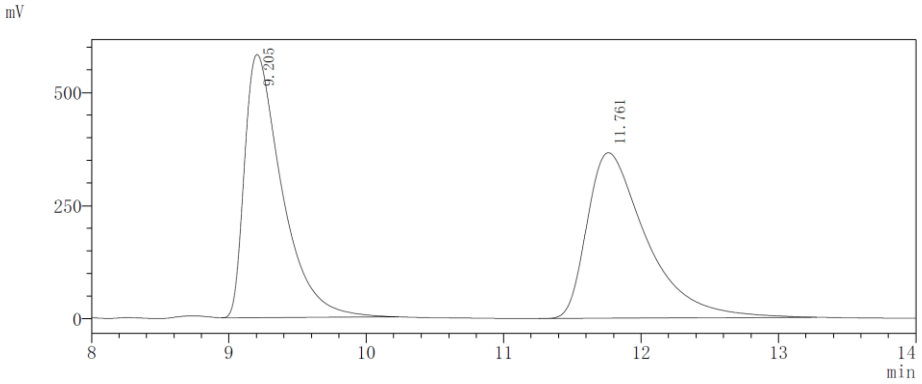

:A Ch2 230nm

RetTime[min]	Area	Hight	Area%
9.205	10958458	581311	49.947
11.761	10981699	365610	50.053

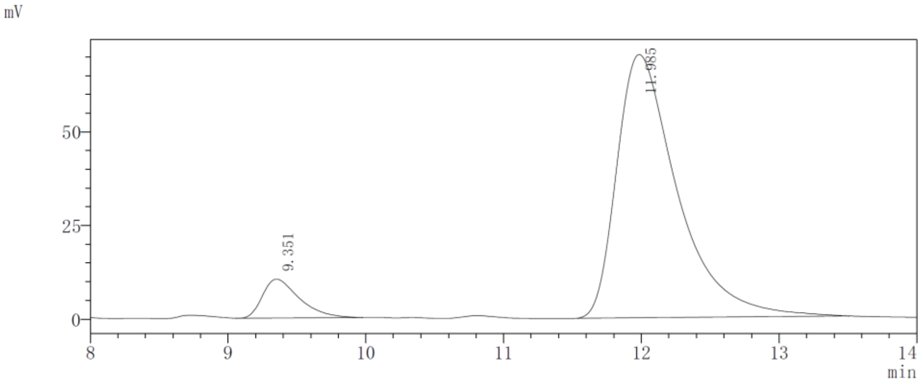

:A Ch2 230nm

RetTime[min]	Area	Hight	Area%
9.351	192620	10369	8.234
11.985	2146659	70194	91.766

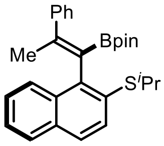

(R,E)-2-(1-(2-((isopropylthio)oxy)naphthalen-1-yl)-2-phenylprop-1-en-1-yl)-4,4,5,5-tetramethyl-1,3,2-dioxaborolane(3g)

Prepared according to the general procedure. 37.0 mg, 83% yield, 86% ee; R_f = 0.4 (PE/EA = 20/1); colorless oil, $[\alpha]^{20}_D$ = -1.3 (c = 0.074, $CHCl_3$). ^1H NMR (300 MHz, $CDCl_3$) δ 7.98 – 7.95 (m, 1H), 7.81 – 7.78 (m, 1H), 7.71 (d, J = 8.6 Hz, 1H), 7.56 – 7.51 (m, 3H), 7.47 – 7.29 (m, 5H), 3.63 (hept, J = 6.8 Hz, 1H), 1.73 (s, 3H), 1.35 (d, J = 4.1 Hz, 3H), 1.33 (d, J = 4.0 Hz, 3H), 1.00 (s, 6H), 0.98 (s, 6H) ppm. ^{13}C NMR (75 MHz, $CDCl_3$) δ 153.0, 145.2, 140.4, 132.4, 132.2, 132.1, 128.2, 128.1, 128.0, 127.4(two overlapping carbon signals), 126.8, 126.3, 125.9, 125.3, 83.1, 36.9, 24.6, 24.6, 23.5, 23.2, 22.7 ppm. HRMS (ESI) *m/z* calcd for $[C_{28}H_{33}BO_2S+H]^+$ 445.2367, found 445.2363. IR (neat, cm^{-1}) 3053, 2976, 2864, 1596, 1503, 1377, 1340, 1145, 1010, 745, 700. HPLC: Daicel Chiralcel IC-3, n-hexane/isopropanol 99.7/0.3, flow rate = 0.5 mL/min, uv-vis λ = 230 nm, t_{R1} = 8.6 min (minor), t_{R2} = 10.1 min (major).

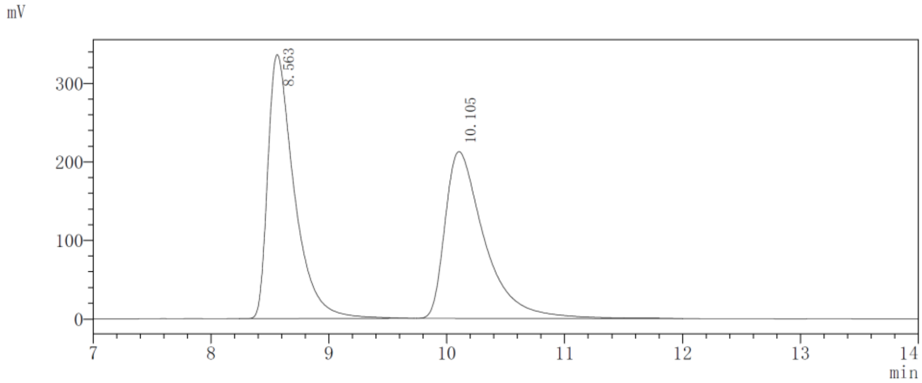

:A Ch2 230nm

RetTime[min]	Area	Hight	Area%
8.563	5240611	335816	51.417
10.105	4951719	212246	48.583

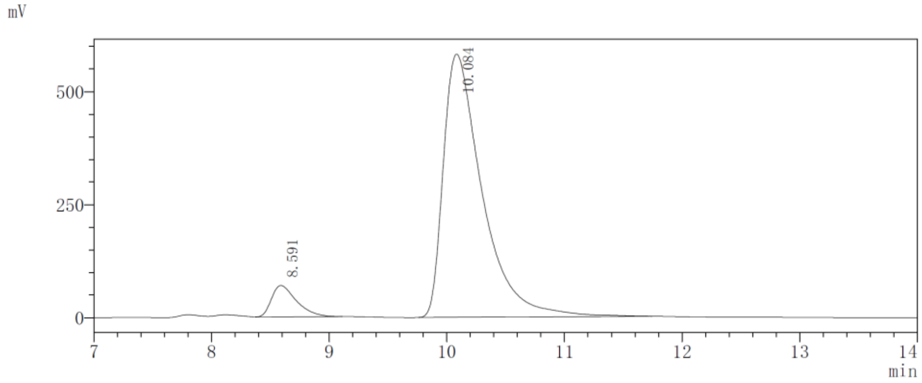

:A Ch2 230nm

RetTime[min]	Area	Hight	Area%
8.591	1022210	69357	7.105
10.084	13365963	582100	92.895

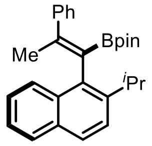

(R,E)-2-(1-(2-isopropoxynaphthalen-1-yl)-2-phenylprop-1-en-1-yl)-4,4,5,5-tetramethyl-1,3,2-dioxaborolane(3h)

Prepared according to the general procedure. 13.8 mg, 33% yield, 22% ee; R_f = 0.5 (PE/EA = 20/1); colorless oil, $[\alpha]^{20}_D$ = -2.6 (c = 0.076, $CHCl_3$). ^1H NMR (300 MHz, $CDCl_3$) δ 8.04 – 8.00 (m, 1H), 7.81 – 7.74 (m, 2H), 7.56 – 7.48 (m, 3H), 7.47 – 7.29 (m, 5H), 3.49 (hept, J = 6.8 Hz, 1H), 1.71 (s, 3H), 1.31 (d, J = 6.8 Hz, 3H), 1.26 (d, J = 6.9 Hz, 3H), 0.96 (s, 6H), 0.95 (s, 6H) ppm. ^{13}C NMR (75 MHz, $CDCl_3$) δ 150.0, 145.1, 143.1, 135.8, 132.3, 132.1, 128.2, 127.9(two overlapping carbon signals), 127.4, 126.8, 126.6, 125.7, 124.8, 124.1, 83.2, 30.6, 24.6, 24.5, 24.1, 23.8, 21.8 ppm. HRMS (ESI) *m/z* calcd for $[C_{28}H_{33}BO_2+H]^+$ 413.2646, found 413.2649. IR (neat, cm^{-1}) 3077, 2976, 1490, 1471, 1371, 1347, 1263, 1146, 1117, 766, 699. HPLC: Daicel Chiralcel IC-3, n-hexane/isopropanol 99.7/0.3, flow rate = 0.5 mL/min, uv-vis λ = 230 nm, t_{R1} = 7.1 min (minor), t_{R2} = 8.4 min (major).

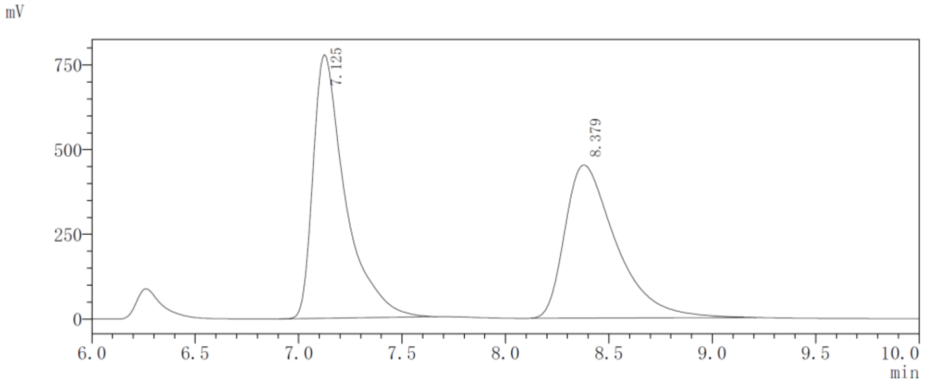

A Ch2 230nm

RetTime[min]	Area	Hight	Area%
7.125	8441935	778812	52.040
8.379	7780195	451635	47.960

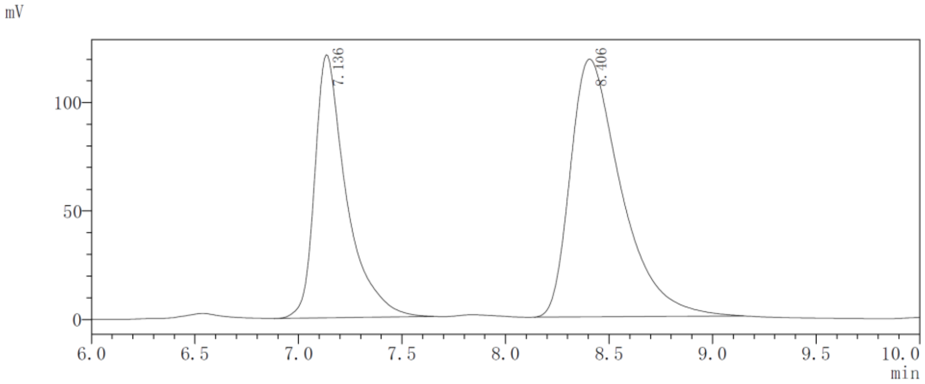

A Ch2 230nm

RetTime[min]	Area	Hight	Area%
7.136	1294494	121362	38.974
8.406	2026967	118846	61.026

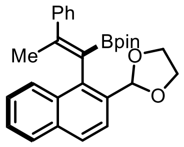

(R,E)-2-(1-(2-(1,3-dioxolan-2-yl)naphthalen-1-yl)-2-phenylprop-1-en-1-yl)-4,4,5,5-tetramethyl-1,3,2-dioxaborolane(3i)

Prepared according to the general procedure. 38.2 mg, 86% yield, 64% ee; R_f = 0.4 (PE/EA = 5/1); colorless oil, $[\alpha]^{20}_{D}$ = +11.4 (c = 0.044, $CHCl_3$). ^1H NMR (300 MHz, $CDCl_3$) δ 8.12 – 8.09 (m, 1H), 7.85 – 7.79 (m, 2H), 7.71 – 7.69 (m, 1H), 7.54 – 7.47 (m, 4H), 7.40 – 7.30 (m, 3H), 6.18 (s, 1H), 4.25 – 3.99 (m, 4H), 1.74 (s, 3H), 0.97 (s, 12H) ppm. ^{13}C NMR (75 MHz, $CDCl_3$) δ 152.0, 144.9, 139.6, 134.3, 131.8, 131.7, 128.1, 127.9, 127.5, 127.0, 126.7, 126.3, 125.9, 123.7, 102.1, 83.4, 77.6, 77.2, 76.7, 65.63, 65.57, 24.4, 22.1 ppm. HRMS (ESI) *m/z* calcd for $[C_{28}H_{31}BO_4+H]^+$ 443.2392, found 443.2388. IR (neat, cm^{-1}) 3091, 2977, 1596, 1472, 1372, 1265, 1144, 1090, 748, 700. HPLC: Daicel Chiralcel IC-3, n-hexane/isopropanol 99.5/0.5, flow rate = 0.5 mL/min, uv-vis λ = 230 nm, t_{R1} = 13.7 min (minor), t_{R2} = 15.2 min (major).

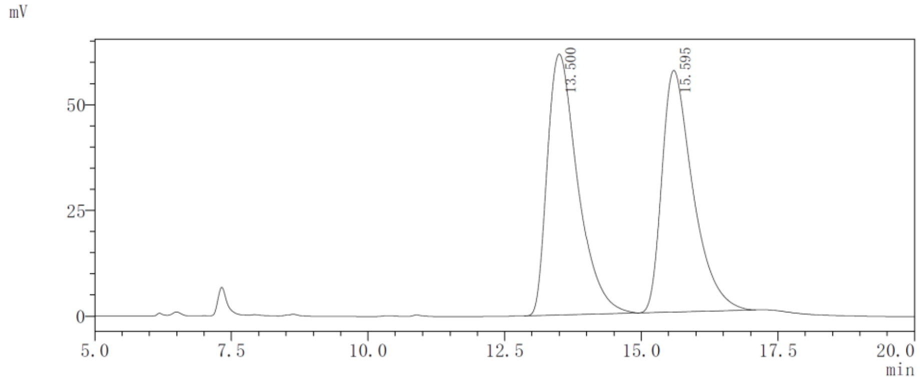

A Ch2 230nm

RetTime[min]	Area	Hight	Area%
13.500	2394638	61721	51.447
15.595	2259898	57140	48.553

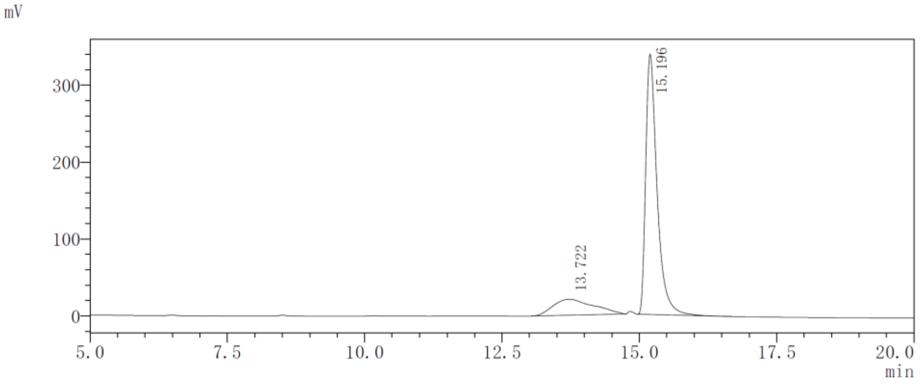

A Ch2 230nm

RetTime[min]	Area	Hight	Area%
13.722	1061795	20823	17.900
15.196	4869989	338398	82.100

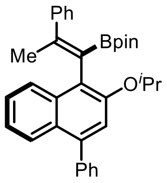

(R,E)-2-(1-(2-isopropoxy-4-phenylnaphthalen-1-yl)-2-phenylprop-1-en-1-yl)-4,4,5,5-tetramethyl-1,3,2-dioxaborolane (4a)

Prepared according to the general procedure. 45.8 mg, 91% yield, 92% ee; R_f = 0.4 (PE/EA = 20/1); colorless oil, $[\alpha]^{20}_D$ = +107.7 (c = 0.026, $CHCl_3$). ^1H NMR (300 MHz, $CDCl_3$) δ 7.99 (d, *J* = 8.3 Hz, 1H), 7.84 (d, *J* = 8.4 Hz, 1H), 7.56 – 7.47 (m, 6H), 7.44 – 7.31 (m, 5H), 7.29 – 7.22 (m, 2H), 4.61 (hept, *J* = 6.0 Hz, 1H), 1.85 (s, 3H), 1.36 (d, *J* = 6.1 Hz, 3H), 1.32 (d, *J* = 6.0 Hz, 3H), 1.02 (s, 6H), 1.00 (s, 6H) ppm. ^{13}C NMR (75 MHz, $CDCl_3$) δ 152.5, 151.6, 145.8, 141.1, 139.9, 133.7, 130.4, 128.32, 128.28, 128.2 (two overlapping carbon signals), 128.0, 127.25, 127.22, 126.3, 126.1, 125.8, 123.8, 120.3, 83.0, 72.9, 24.7, 24.5, 23.2, 22.9, 22.6 ppm. HRMS (ESI) *m/z* calcd for $[C_{34}H_{37}BO_3+H]^+$ 505.2909, found 505.2908. IR (neat, cm^{-1}) 3062, 2994, 1609, 1489, 1379, 1370, 1208, 1148, 1109, 752, 690. HPLC: Daicel Chiralcel IC-3, n-hexane/isopropanol 98/2, flow rate = 0.3 mL/min, uv-vis λ = 230 nm, t_{R1} = 11.2 min (minor), t_{R2} = 12.0 min (major).

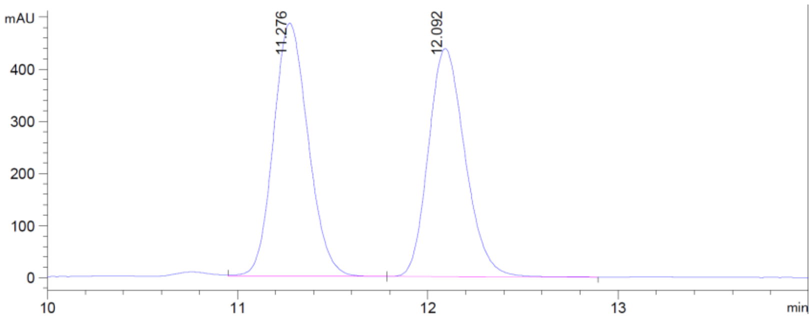

Signal 2: MWD1 B, Sig=230,4 Ref=off

Peak #	RT [min]	Type	Height	Width [min]	Area %	Area
1	11.276	VB	485.938	0.198	51.350	6.176e3
2	12.092	BB	437.487	0.208	48.650	5.851e3

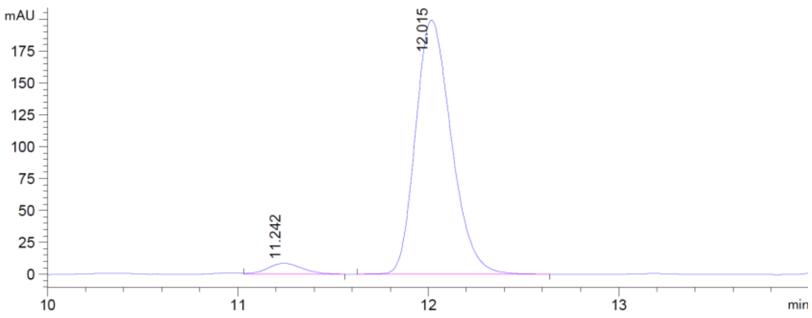

Signal 2: MWD1 B, Sig=230,4 Ref=off

Peak #	RT [min]	Type	Height	Width [min]	Area %	Area
1	11.242	VV	8.524	0.191	4.017	108.931
2	12.015	BV	199.134	0.204	95.983	2.603e3

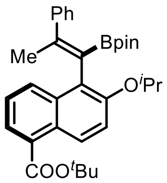

***tert*-butyl(*R*,*E*)-6-isopropoxy-5-(2-phenyl-1-(4,4,5,5-tetramethyl-1,3,2-dioxaborolan-2-yl)prop-1-en-1-yl)-1-naphthoate (4b)**

Prepared according to the general procedure. 33.9 mg, 64% yield, 95% ee; R_f = 0.2 (PE/EA = 20/1); colorless oil, $[\alpha]^{20}_D$ = +42.5 (c = 0.04, $CHCl_3$). ^1H NMR (300 MHz, $CDCl_3$) δ 8.71 (d, J = 9.4 Hz, 1H), 8.10 (d, J = 8.5 Hz, 1H), 7.91 – 7.88 (m, 1H), 7.47 – 7.44 (m, 2H), 7.42 – 7.31 (m, 5H), 4.63 (hept, J = 6.1 Hz, 1H), 1.76 (s, 3H), 1.68 (s, 9H), 1.36 (d, J = 6.1 Hz, 3H), 1.31 (d, J = 6.0 Hz, 3H), 0.98 (s, 6H), 0.95 (s, 6H) ppm. ^{13}C NMR (75 MHz, $CDCl_3$) δ 167.8, 152.3, 152.1, 145.5, 134.0, 130.1, 129.7, 128.8, 128.1, 128.0, 127.4, 127.30, 127.26, 125.4, 124.6, 120.1, 83.0, 81.4, 72.4, 28.5, 24.7, 24.5, 22.8, 22.7, 22.5 ppm. HRMS (ESI) *m/z* calcd for $[C_{33}H_{41}BO_5+H]^+$ 529.3120, found 529.3115. IR (neat, cm^{-1}) 3056, 2928, 1709, 1599, 1509, 1370, 1342, 1253, 1118, 764, 700. HPLC: Daicel Chiralcel IC-3, n-hexane/isopropanol 98/2, flow rate = 0.5 mL/min, uv-vis λ = 230 nm, t_{R1} = 8.3 min (minor), t_{R2} = 9.6 min (major).

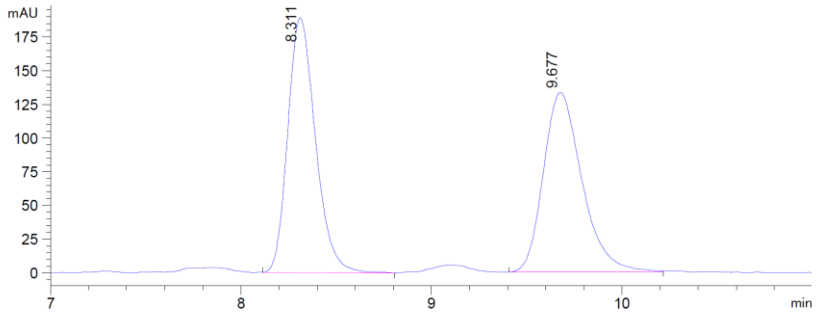

Signal 2: MWD1 B, Sig=230,4 Ref=off

Peak #	RT [min]	Type	Height	Width [min]	Area %	Area
1	8.311	VB	189.153	0.156	50.437	1.911e3
2	9.677	BV	133.260	0.216	49.563	1.878e3

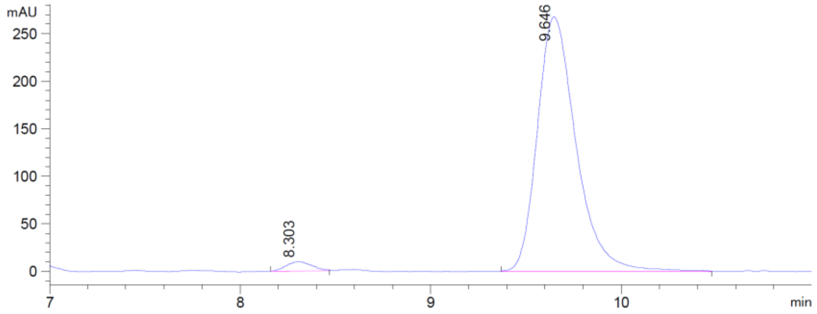

Signal 2: MWD1 B, Sig=230,4 Ref=off

Peak #	RT [min]	Type	Height	Width [min]	Area %	Area
1	8.303	MM	9.658	0.155	2.305	89.705
2	9.646	MM	268.088	0.236	97.695	3.802e3

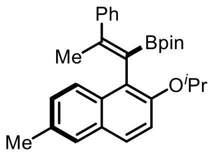

(R,E)-2-(1-(2-isopropoxy-6-methylnaphthalen-1-yl)-2-phenylprop-1-en-1-yl)-4,4,5,5-tetramethyl-1,3,2-dioxaborolane (4c)

Prepared according to the general procedure. 34.2 mg, 77% yield, 91% ee; R_f = 0.4 (PE/EA = 20/1); colorless oil, $[\alpha]^{20}_{D}$ = +86.7 (c = 0.03, $CHCl_3$). ^{1}H NMR (300 MHz, $CDCl_3$) δ 7.81 (d, *J* = 8.6 Hz, 1H), 7.62 (d, *J* = 8.8 Hz, 1H), 7.55 (s, 1H), 7.48 – 7.44 (m, 2H), 7.39 – 7.30 (m, 3H), 7.25 – 7.20 (m, 2H), 4.54 (hept, *J* = 6.1 Hz, 1H), 2.47 (s, 3H), 1.80 (s, 3H), 1.34 (d, *J* = 6.1 Hz, 3H), 1.29 (d, *J* = 6.0 Hz, 3H), 0.99 (s, 6H), 0.97 (s, 6H) ppm. ^{13}C NMR (75 MHz, $CDCl_3$) δ 151.9, 151.6, 145.7, 133.1, 131.4, 130.3, 129.0, 128.2 (two overlapping carbon signals), 128.0, 127.2, 127.1, 126.9, 125.6, 119.8, 82.9, 73.1, 24.7, 24.5, 22.9, 22.8, 22.5, 21.5 ppm. HRMS (ESI) *m/z* calcd for $[C_{29}H_{35}BO_3+H]^+$ 443.2752, found 443.2753. IR (neat, cm^{-1}) 3057, 2976, 1596, 1491, 1371, 1339, 1266, 1145, 1112, 765. 700. HPLC: Daicel Chiralcel IC-3, n-hexane/isopropanol 99/1, flow rate = 0.5 mL/min, uv-vis λ = 230 nm, t_{R1} = 7.1 min (minor), t_{R2} = 9.0 min (major).

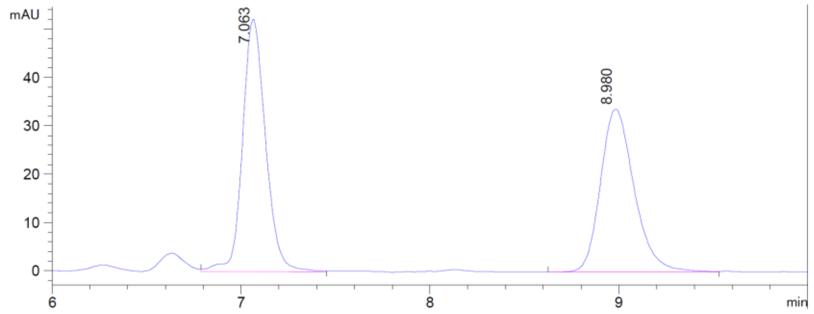

Signal 2: MWD1 B, Sig=230,4 Ref=off

Peak #	RT [min]	Type	Height	Width [min]	Area %	Area
1	7.063	VV	52.290	0.131	51.617	445.735
2	8.980	VV	33.730	0.191	48.383	417.806

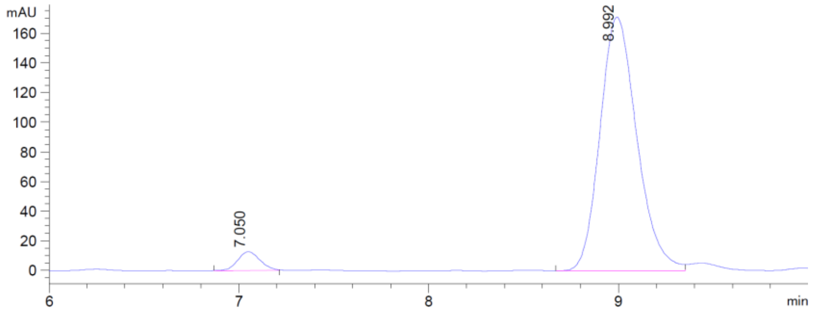

Signal 2: MWD1 B, Sig=230,4 Ref=off

Peak #	RT [min]	Type	Height	Width [min]	Area %	Area
1	7.050	MM	12.740	0.136	4.298	104.029
2	8.992	BV	171.291	0.210	95.702	2.316e3

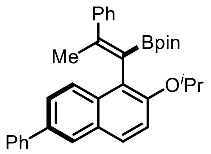

(R,E)-2-(1-(2-isopropoxy-6-phenylnaphthalen-1-yl)-2-phenylprop-1-en-1-yl)-4,4,5,5-tetramethyl-1,3,2-dioxaborolane (4d)

Prepared according to the general procedure. 39.3 mg, 78% yield, 96% ee; R_f = 0.4 (PE/EA = 20/1); colorless oil, $[\alpha]^{20}_D$ = +92.5 (c = 0.04, $CHCl_3$). ^1H NMR (300 MHz, $CDCl_3$) δ 8.00 – 7.98 (m, 2H), 7.77 (d, J = 8.9 Hz, 1H), 7.72 – 7.66 (m, 3H), 7.50 – 7.43 (m, 4H), 7.40 – 7.27 (m, 5H), 4.59 (hept, J = 6.0 Hz, 1H), 1.83 (s, 3H), 1.37 (d, J = 6.1 Hz, 3H), 1.32 (d, J = 6.0 Hz, 3H), 1.00 (s, 6H), 0.98 (s, 6H) ppm. ^{13}C NMR (75 MHz, $CDCl_3$) δ 152.4, 152.2, 145.6, 141.5, 136.3, 132.4, 130.2, 128.91, 128.86, 128.2, 128.0 (two overlapping carbon signals), 127.4, 127.3, 127.1, 126.3, 126.1, 125.6, 119.8, 83.0, 73.0, 24.7, 24.5, 23.0, 22.8, 22.5 ppm. HRMS (ESI) *m/z* calcd for $[C_{34}H_{37}BO_3+H]^+$ 505.2909, found 505.2903. IR (neat, cm^{-1}) 3056, 2976, 1591, 1490, 1371, 1338, 1242, 1144, 1111, 760, 699. HPLC: Daicel Chiralcel IC-3, n-hexane/isopropanol 99/1, flow rate = 0.5 mL/min, uv-vis λ = 230 nm, t_{R1} = 7.9 min (minor), t_{R2} = 9.6 min (major).

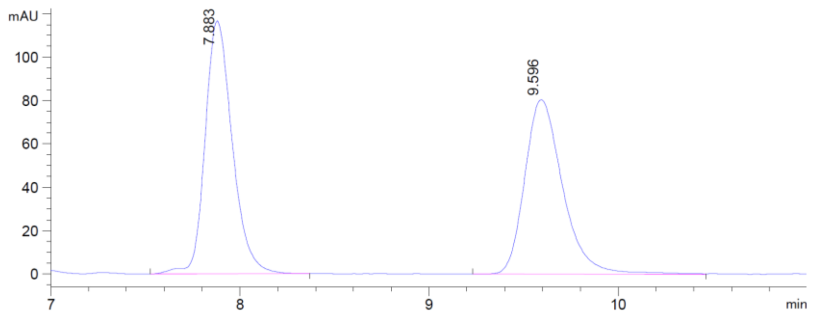

Signal 2: MWD1 B, Sig=230,4 Ref=off

Peak #	RT [min]	Type	Height	Width [min]	Area %	Area
1	7.883	BB	116.649	0.155	51.034	1.170e3
2	9.596	VV	80.465	0.213	48.966	1.123e3

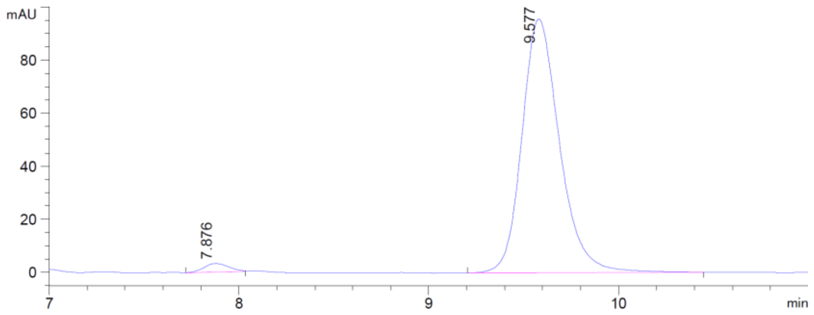

Signal 2: MWD1 B, Sig=230,4 Ref=off

Peak #	RT [min]	Type	Height	Width [min]	Area %	Area
1	7.876	MM	3.262	0.148	2.153	29.053
2	9.577	BB	95.674	0.211	97.847	1.320e3

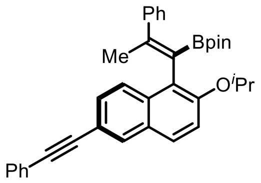

(R,E)-2-(1-(2-isopropoxy-6-(phenylethynyl)naphthalen-1-yl)-2-phenylprop-1-en-1-yl)-4,4,5,5-tetramethyl-1,3,2-dioxa borolane (4e)

Prepared according to the general procedure. 39.0 mg, 74% yield, 93% ee; R_f = 0.4 (PE/EA = 20/1); colorless oil, $[\alpha]^{20}_{D}$ = +62.5 (c = 0.032, $CHCl_3$). ^{1}H NMR (300 MHz, $CDCl_3$) δ 8.01 (s, 1H), 7.88 (d, J = 8.8 Hz, 1H), 7.70 (d, J = 8.9 Hz, 1H), 7.59 – 7.55 (m, 2H), 7.51 – 7.46 (m, 3H), 7.40 – 7.27 (m, 7H), 4.61 (hept, J = 6.1 Hz, 1H), 1.80 (s, 3H), 1.37 (d, J = 6.0 Hz, 3H), 1.31 (d, J = 6.0 Hz, 3H), 1.01 (s, 6H), 0.97 (s, 6H) ppm. ^{13}C NMR (75 MHz, $CDCl_3$) δ 153.0, 152.5, 145.5, 132.8, 131.8, 131.7, 129.4, 128.7, 128.5 (two overlapping carbon signals), 128.2, 128.1, 128.0, 127.6, 127.3, 125.8, 123.7, 119.4, 118.1, 90.4, 89.1, 83.0, 72.7, 24.7, 24.5, 22.9, 22.8, 22.5 ppm. HRMS (ESI) m/z calcd for $[C_{36}H_{37}BO_3+H]^+$ 529.2909, found 529.2907. IR (neat, cm^{-1}) 3053, 2976, 1598, 1469, 1372, 1336, 1264, 1144, 1111, 756, 691. HPLC: Daicel Chiralcel IC-3, n-hexane/isopropanol 95/5, flow rate = 0.5 mL/min, uv-vis λ = 230 nm, t_{R1} = 7.0 min (minor), t_{R2} = 7.6 min (major).

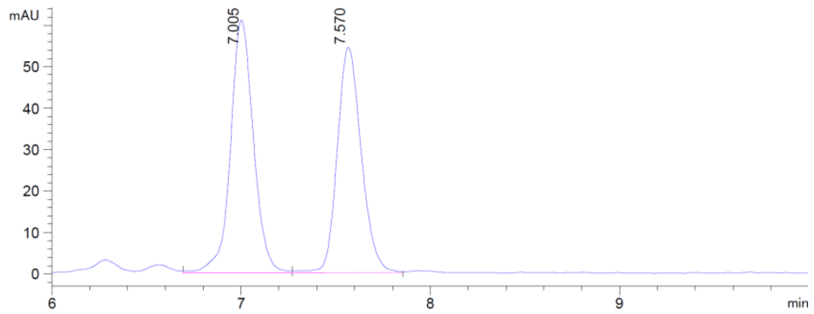

Signal 2: MWD1 B, Sig=230,4 Ref=off

Peak #	RT [min]	Type	Height	Width [min]	Area %	Area
1	7.005	VV	61.023	0.131	51.699	515.549
2	7.570	VV	54.422	0.137	48.301	481.662

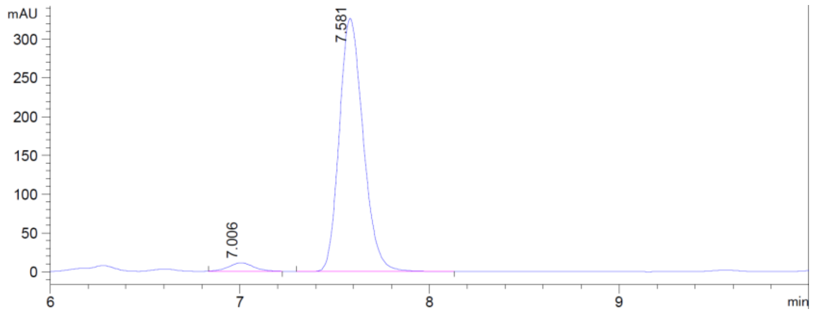

Signal 2: MWD1 B, Sig=230,4 Ref=off

Peak #	RT [min]	Type	Height	Width [min]	Area %	Area
1	7.006	MM	11.121	0.153	3.446	102.132
2	7.581	BV	326.402	0.136	96.554	2.862e3

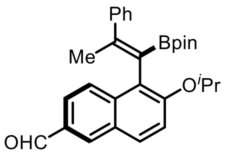

(R,E)-6-isopropoxy-5-(2-phenyl-1-(4,4,5,5-tetramethyl-1,3,2-dioxaborolan-2-yl)prop-1-en-1-yl)-2-naphthaldehyde (4f) Prepared according to the general procedure. 31.4 mg, 69% yield, 94% ee; R_f = 0.4 (PE/EA = 5/1); colorless oil, $[\alpha]^{20}_D$ = +12.1 (c = 0.058, $CHCl_3$). ^{1}H NMR (300 MHz, $CDCl_3$) δ 10.10 (s, 1H), 8.27 (s, 1H), 8.00 (d, J = 8.8 Hz, 1H), 7.91 – 7.85 (m, 2H), 7.47 – 7.45 (m, 2H), 7.39 – 7.30 (m, 4H), 4.69 (hept, J = 5.7 Hz, 1H), 1.78 (s, 3H), 1.39 (d, J = 6.0 Hz, 3H), 1.35 (d, J = 6.0 Hz, 3H), 0.99 (s, 6H), 0.96 (s, 6H) ppm. ^{13}C NMR (75 MHz, $CDCl_3$) δ 192.5, 155.1, 152.9, 145.3, 136.7, 135.3, 132.2, 129.6, 128.52, 128.51, 128.1, 128.0, 127.4, 126.6, 122.8, 118.6, 83.0, 72.2, 24.6, 24.5, 22.9, 22.6, 22.5 ppm. HRMS (ESI) *m/z* calcd for $[C_{29}H_{33}BO_4+H]^+$ 457.2545, found 457.2546. IR (neat, cm^{-1}) 3055, 2977, 1693, 1619, 1471, 1372, 1233, 1145, 772, 700. HPLC: Daicel Chiralcel IC-3, n-hexane/isopropanol 95/5, flow rate = 0.5 mL/min, uv-vis λ = 230 nm, t_{R1} = 22.7 min (minor), t_{R2} = 28.8 min (major).

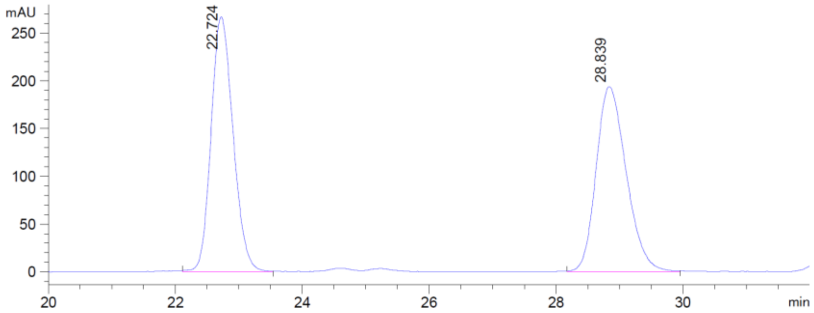

Signal 2: MWD1 B, Sig=230,4 Ref=off

Peak #	RT [min]	Type	Height	Width [min]	Area %	Area
1	22.724	VV	266.365	0.373	50.107	6.412e3
2	28.839	VV	193.613	0.513	49.893	6.385e3

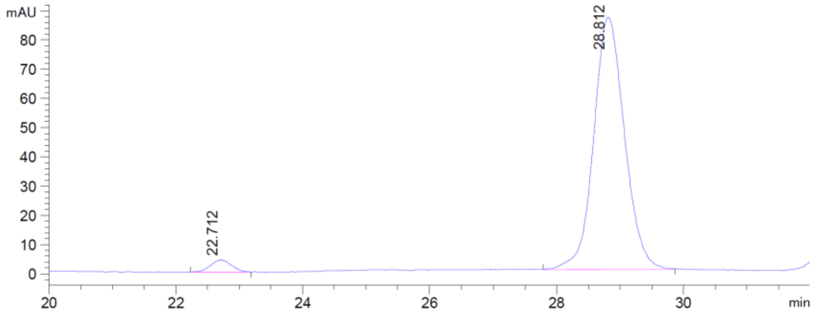

Signal 2: MWD1 B, Sig=230,4 Ref=off

Peak #	RT [min]	Type	Height	Width [min]	Area %	Area
1	22.712	BV	4.173	0.279	3.169	95.626
2	28.812	VV	86.384	0.515	96.831	2.922e3

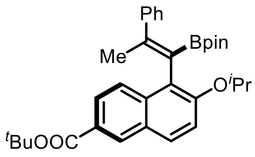

tert-butyl(R,E)-6-isopropoxy-5-(2-phenyl-1-(4,4,5,5-tetramethyl-1,3,2-dioxaborolan-2-yl)prop-1-en-1-yl)-2-naphthoate (4g)

Prepared according to the general procedure. 31.0 mg, 59% yield, 95% ee; R_f = 0.2 (PE/EA = 20/1); colorless oil, $[\alpha]^{20}_D$ = +46.9 (c = 0.016, $CHCl_3$). ^{1}H NMR (300 MHz, $CDCl_3$) δ 8.47 (s, 1H), 7.96 – 7.89 (m, 2H), 7.82 (d, *J* = 8.9 Hz, 1H), 7.47 – 7.43 (m, 2H), 7.39 – 7.29 (m, 4H), 4.65 (hept, *J* = 6.2 Hz, 1H), 1.76 (s, 3H), 1.63 (s, 9H), 1.37 (d, *J* = 6.1 Hz, 3H), 1.33 (d, *J* = 6.0 Hz, 3H), 0.99 (s, 6H), 0.95 (s, 6H) ppm. ^{13}C NMR (75 MHz, $CDCl_3$) δ 166.5, 154.0, 152.4, 145.5, 135.5, 131.0, 129.2, 128.7, 128.3, 128.2, 128.0, 127.3, 127.0, 125.6, 125.4, 118.8, 83.0, 80.8, 72.4, 28.4, 24.6, 24.5, 22.8, 22.7, 22.6 ppm. HRMS (ESI) *m/z* calcd for $[C_{33}H_{41}BO_5+Na]^+$ 551.2939, found 551.2931. IR (neat, cm^{-1}) 3055, 2930, 1711, 1590, 1471, 1370, 1341, 1285, 1146, 1109, 762, 700. HPLC: Daicel Chiralcel IA-3, n-hexane/isopropanol 99/1, flow rate = 0.2 mL/min, uv-vis λ = 230 nm, t_{R1} = 6.8 min (minor), t_{R2} = 7.4 min (major).

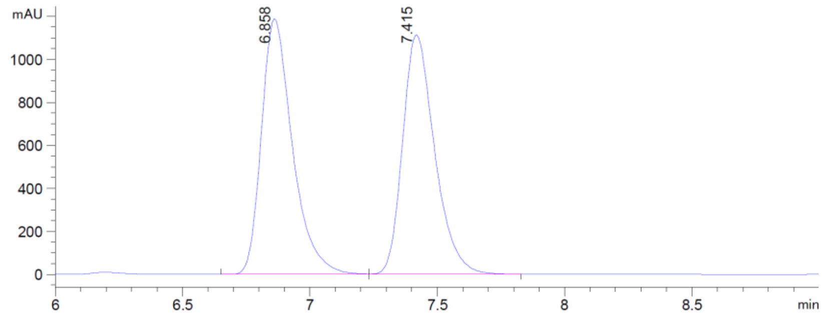

Signal 2: MWD1 B, Sig=230,4 Ref=off

Peak #	RT [min]	Type	Height	Width [min]	Area %	Area
1	6.858	BB	1.187e3	0.129	50.818	1.003e4
2	7.415	BV	1.113e3	0.134	49.182	9.712e3

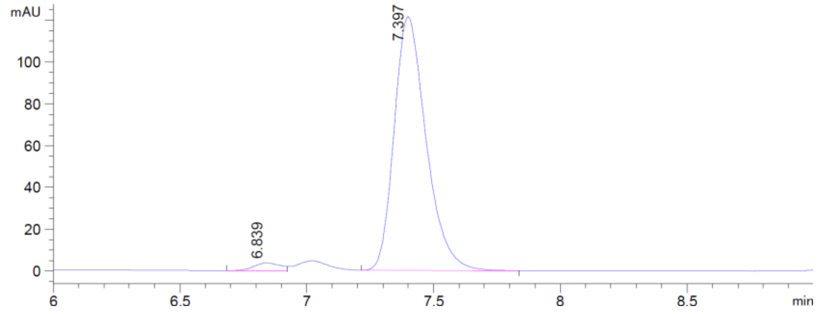

Signal 2: MWD1 B, Sig=230,4 Ref=off

Peak #	RT [min]	Type	Height	Width [min]	Area %	Area
1	6.839	BV	3.621	0.110	2.450	26.772
2	7.397	BB	121.420	0.134	97.550	1.066e3

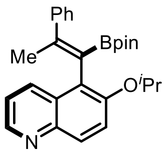

(R,E)-6-isopropoxy-5-(2-phenyl-1-(4,4,5,5-tetramethyl-1,3,2-dioxaborolan-2-yl)prop-1-en-1-yl)quinoline (4h)

Prepared according to the general procedure. 34.3 mg, 80% yield, 93% ee; R_f = 0.3 (PE/EA = 5/1); colorless oil, $[\alpha]^{20}_{D}$ = +71.4 (c = 0.028, $CHCl_3$). ^1H NMR (300 MHz, $CDCl_3$) δ 8.78 (dd, J = 4.1, 1.6 Hz, 1H), 8.27 – 8.23 (m, 1H), 8.00 (d, J = 9.2 Hz, 1H), 7.51 – 7.44 (m, 3H), 7.40 – 7.30 (m, 4H), 4.62 (hept, J = 6.1 Hz, 1H), 1.78 (s, 3H), 1.37 (d, J = 6.1 Hz, 3H), 1.32 (d, J = 6.0 Hz, 3H), 0.99 (s, 12H) ppm. ^{13}C NMR (75 MHz, $CDCl_3$) δ 152.8, 152.4, 148.2, 145.2, 144.9, 134.0, 128.9, 128.5, 128.2, 128.1 (two overlapping carbon signals), 127.4, 122.3, 120.9, 83.1, 73.0, 24.7, 24.5, 22.9, 22.7, 22.4 ppm. IR (neat, cm^{-1}) 3061, 2931, 1588, 1496, 1379, 1372, 1255, 1145, 1112, 771, 700. HRMS (ESI) *m/z* calcd for $[C_{27}H_{32}BNO_3+H]^+$ 430.2548, found 430.2544. HPLC: Daicel Chiralcel IC-3, n-hexane/isopropanol 95/5, flow rate = 0.5 mL/min, uv-vis λ = 230 nm, t_{R1} = 23.3 min (minor), t_{R2} = 24.7 min (major).

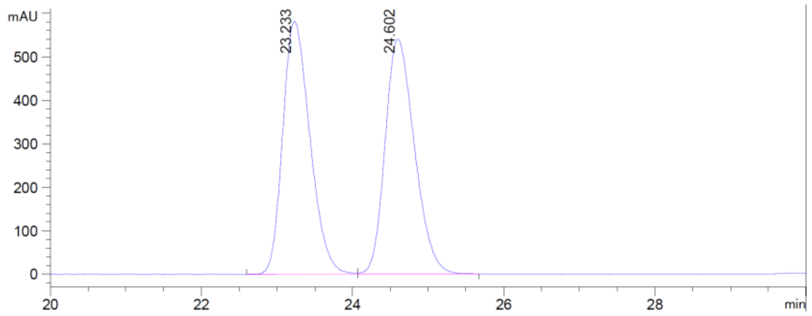

Signal 2: MWD1 B, Sig=230,4 Ref=off

Peak #	RT [min]	Type	Height	Width [min]	Area %	Area
1	23.233	BV	581.840	0.388	49.925	1.458e4
2	24.602	VV	540.751	0.421	50.075	1.462e4

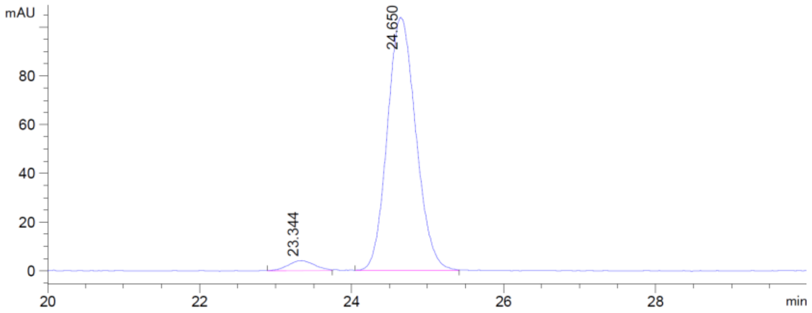

Signal 2: MWD1 B, Sig=230,4 Ref=off

Peak #	RT [min]	Type	Height	Width [min]	Area %	Area
1	23.344	VV	4.137	0.312	3.602	101.547
2	24.650	VV	103.802	0.411	96.398	2.718e3

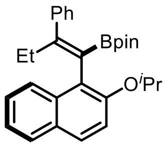

(R,E)-2-(1-(2-isopropoxynaphthalen-1-yl)-2-phenylbut-1-en-1-yl)-4,4,5,5-tetramethyl-1,3,2-dioxaborolane (4i)

Prepared according to the general procedure. 33.1 mg, 75% yield, 97% ee; R_f = 0.4 (PE/EA = 20/1); colorless oil, $[\alpha]^{20}_{D}$ = -22.2 (c = 0.036, $CHCl_3$). ^{1}H NMR (300 MHz, $CDCl_3$) δ 7.98 (d, J = 8.4 Hz, 1H), 7.78 – 7.76 (m, 1H), 7.71 (d, J = 8.9 Hz, 1H), 7.44 – 7.29 (m, 7H), 7.28 – 7.25 (m, 1H), 4.64 (hept, J = 6.1 Hz, 1H), 2.15 (q, J = 7.5 Hz, 2H), 1.37 (d, J = 3.9 Hz, 3H), 1.35 (d, J = 3.8 Hz, 3H), 0.94 (s, 6H), 0.90 (s, 6H), 0.72 (t, J = 7.5 Hz, 3H) ppm. ^{13}C NMR (75 MHz, $CDCl_3$) δ 157.4, 152.0, 144.1, 133.7, 129.7, 128.7, 128.2, 127.9, 127.8, 127.4, 126.9, 125.9, 125.7, 123.6, 118.9, 82.8, 72.3, 28.7, 24.6, 24.4, 22.71, 22.69, 12.0 ppm. HRMS (ESI) *m/z* calcd for $[C_{29}H_{35}BO_3H]^+$ 443.2752, found 443.2761. IR (neat, cm^{-1}) 3057, 2975, 1621, 1591, 1379, 1371, 1264, 1146, 1112, 750, 701. HPLC: Daicel Chiralcel OD-H, n-hexane/isopropanol 99.9/0.1, flow rate = 0.5 mL/min, uv-vis λ = 230 nm, t_{R1} = 15.0 min (major), t_{R2} = 24.0 min (minor).

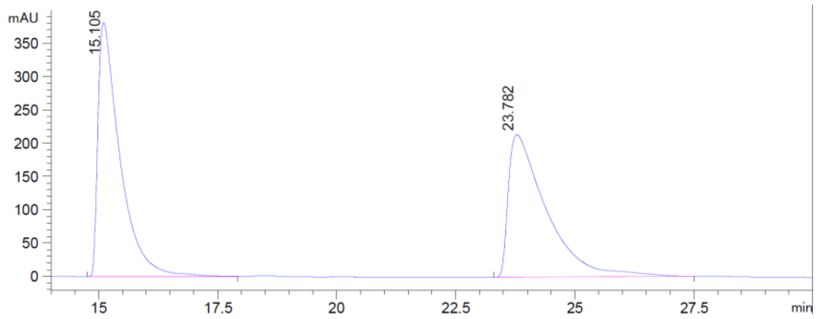

Signal 2: MWD1 B, Sig=230,4 Ref=off

Peak #	RT [min]	Type	Height	Width [min]	Area %	Area
1	15.105	BV	381.315	0.466	50.694	1.228e4
2	23.782	BB	213.958	0.780	49.306	1.195e4

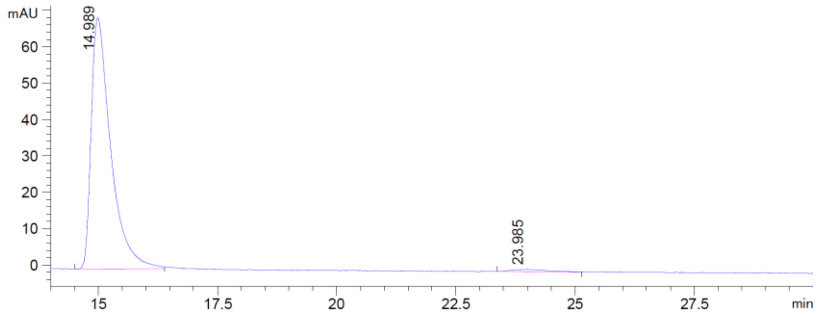

Signal 2: MWD1 B, Sig=230,4 Ref=off

Peak #	RT [min]	Type	Height	Width [min]	Area %	Area
1	14.989	BV	68.947	0.419	98.413	1.966e3
2	23.985	MM	0.677	0.781	1.587	31.701

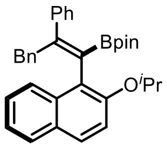

(R,E)-2-(1-(2-isopropoxynaphthalen-1-yl)-2,3-diphenylprop-1-en-1-yl)-4,4,5,5-tetramethyl-1,3,2-dioxaborolane (4j)

Prepared according to the general procedure. 36.0 mg, 71% yield, 97% ee; R_f = 0.4 (PE/EA = 20/1); white solid, m. p. 120 – 124 °C, $[\alpha]^{20}_{D}$ = -47.1 (c = 0.034, $CHCl_3$). ^1H NMR (300 MHz, $CDCl_3$) δ 8.02 (d, J = 8.4 Hz, 1H), 7.78 (d, J = 8.0 Hz, 1H), 7.73 (d, J = 9.0 Hz, 1H), 7.46 – 7.41 (m, 1H), 7.36 – 7.19 (m, 7H), 7.05 – 6.98 (m, 3H), 6.93 – 6.90 (m, 2H), 4.72 (hept, J = 6.0 Hz, 1H), 3.48 (s, 2H), 1.44 – 1.41 (m, 6H), 0.94 (s, 6H), 0.89 (s, 6H) ppm. ^{13}C NMR (75 MHz, $CDCl_3$) δ 153.5, 152.1, 144.0, 139.4, 133.8, 129.7, 129.3, 129.0, 128.0, 127.75, 127.72, 127.5, 126.8, 126.2, 125.9, 125.7, 125.5, 123.4, 117.0, 82.9, 71.1, 41.9, 24.54, 24.48, 22.9, 22.6 ppm. HRMS (ESI) *m/z* calcd for $[C_{34}H_{37}BO_3+H]^+$ 505.2909, found 505.2909. IR (neat, cm^{-1}) 3063, 2928, 1591, 1506, 1379, 1371, 1265, 1144, 744, 699. HPLC: Daicel Chiralcel IC-3, n-hexane/isopropanol 99/1, flow rate = 0.2 mL/min, uv-vis λ = 230 nm, t_{R1} = 19.3 min (minor), t_{R2} = 20.4 min (major).

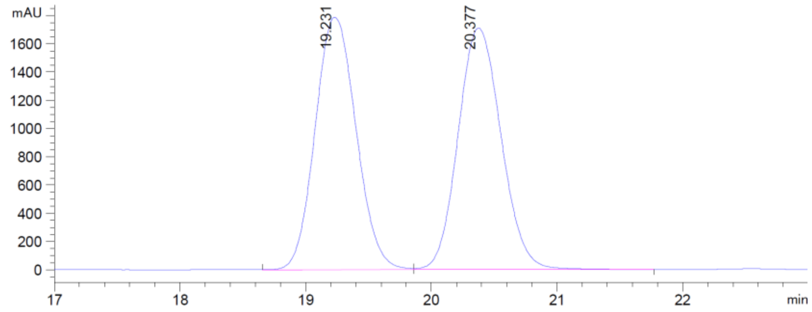

Signal 2: MWD1 B, Sig=230,4 Ref=off

Peak #	RT [min]	Type	Height	Width [min]	Area %	Area
1	19.231	VV	1.788e3	0.355	49.822	4.057e4
2	20.377	VV	1.712e3	0.372	50.178	4.086e4

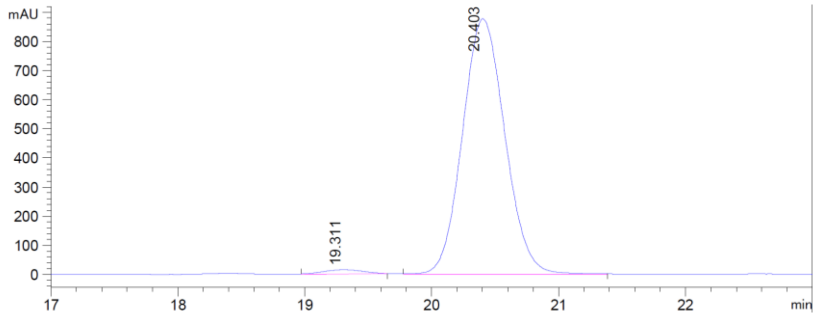

Signal 2: MWD1 B, Sig=230,4 Ref=off

Peak #	RT [min]	Type	Height	Width [min]	Area %	Area
1	19.311	MM	14.948	0.365	1.579	327.388
2	20.403	VV	876.583	0.364	98.421	2.040e4

(R,E)-2-(1-(2-isopropoxynaphthalen-1-yl)-2-(p-tolyl)prop-1-en-1-yl)-4,4,5,5-tetramethyl-1,3,2-dioxaborolane (4k)

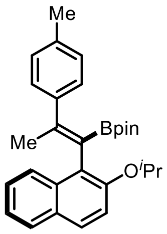

Prepared according to the general procedure. 37.9 mg, 86% yield, 93% ee; R_f = 0.4 (PE/EA = 20/1); white solid, m. p. 80 – 82 °C, $[\alpha]^{20}_D$ = +64.7 (c = 0.034, $CHCl_3$). ^1H NMR (300 MHz, $CDCl_3$) δ 7.91 (d, J = 8.3 Hz, 1H), 7.80 – 7.76 (m, 1H), 7.70 (d, J = 8.9 Hz, 1H), 7.41 – 7.29 (m, 4H), 7.25 (d, J = 8.9 Hz, 1H), 7.18 – 7.15(m, 2H), 4.56 (hept, J = 6.0 Hz, 1H), 2.38 (s, 3H), 1.78 (s, 2H), 1.34 (d, J = 6.1 Hz, 3H), 1.29 (d, J = 6.0 Hz, 3H), 1.01 (s, 6H), 0.99 (s, 6H) ppm. ^{13}C NMR (75 MHz, $CDCl_3$) δ 152.3, 151.9, 142.6, 136.9, 133.3, 130.0, 129.3, 128.6, 128.1 (two overlapping carbon signals), 127.5, 125.9, 125.8, 123.7, 119.6, 82.9, 73.0, 24.7, 24.5, 22.9, 22.8, 22.5, 21.4 ppm. HRMS (ESI) *m/z* calcd for $[C_{29}H_{35}BO_3+H]^+$ 444.2786, found 444.2782. IR (neat, cm^{-1}) 3064, 2974, 1588, 1508, 1379, 1371, 1260, 1146, 1106, 818. HPLC: Daicel Chiralcel IC-3, n-hexane/isopropanol 98/2, flow rate = 0.5 mL/min, uv-vis λ = 230 nm, t_{R1} = 6.8 min (minor), t_{R2} = 8.3 min (major).

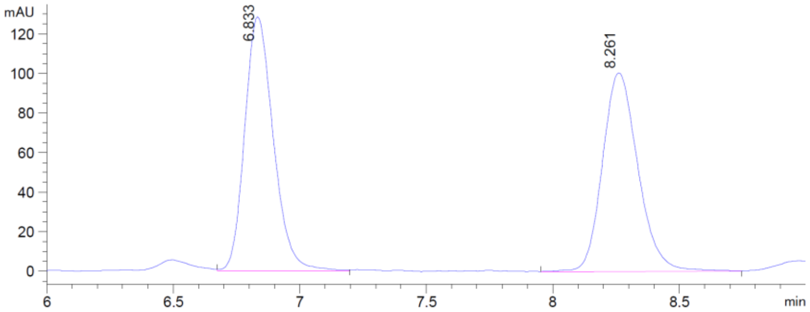

Signal 2: MWD1 B, Sig=230,4 Ref=off

Peak #	RT [min]	Type	Height	Width [min]	Area %	Area
1	6.833	VV	128.318	0.122	50.841	1.011e3
2	8.261	BB	100.316	0.152	49.159	977.203

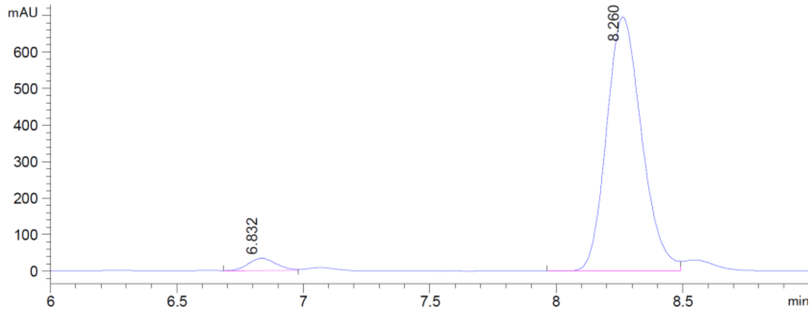

Signal 2: MWD1 B, Sig=230,4 Ref=off

Peak #	RT [min]	Type	Height	Width [min]	Area %	Area
1	6.832	MM	33.315	0.128	3.665	255.341
2	8.260	BV	695.926	0.149	96.335	6.711e3

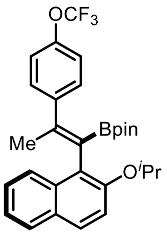

(R,E)-2-(1-(2-isopropoxynaphthalen-1-yl)-2-(4-(trifluoromethoxy) phenyl)prop-1-en-1-yl)-4,4,5,5-tetramethyl-1,3,2-dioxaborolane (4l)

Prepared according to the general procedure. 45.6 mg, 89% yield, 97% ee; R_f = 0.4 (PE/EA = 20/1); white solid, m. p. 68 – 70 °C, $[\alpha]^{20}_D$ = +53.0 (c = 0.066, $CHCl_3$). ^1H NMR (300 MHz, $CDCl_3$) δ 7.86 (d, *J* = 8.4 Hz, 1H), 7.78 (d, *J* = 8.1 Hz, 1H), 7.73 (d, *J* = 8.9 Hz, 1H), 7.48 – 7.43 (m, 2H), 7.41 – 7.30 (m, 2H), 7.25 – 7.20 (m, 3H), 4.57 (hept, *J* = 6.1 Hz, 1H), 1.78 (s, 3H), 1.35 (d, *J* = 6.1 Hz, 3H), 1.31 (d, *J* = 6.0 Hz, 3H), 0.98 (s, 6H), 0.96 (s, 6H) ppm. ^{13}C NMR (75 MHz, $CDCl_3$) δ 152.2, 151.0, 148.5, 144.7, 133.1, 129.9, 129.5, 128.3, 128.2, 127.8, 126.0, 125.5, 123.7, 120.7 (q, *J* = 255.2 Hz), 120.6, 119.0, 83.1, 72.8, 24.6, 24.5, 23.1, 22.8, 22.6 ppm. ^{19}F NMR (282 MHz, $CDCl_3$) δ -57.88 ppm. HRMS (ESI) *m/z* calcd for $[C_{29}H_{32}BF_3O_4+H]^+$ 513.2419, found 513.2412. IR (neat, cm^{-1}) 3063, 2973, 1505, 1460, 1380, 1372, 1261, 1145, 1106, 839. HPLC: Daicel Chiralcel OD-H, n-hexane/isopropanol 99.9/0.1, flow rate = 0.65 mL/min, uv-vis λ = 230 nm, t_{R1} = 12.0 min (major), t_{R2} = 20.5 min (minor).

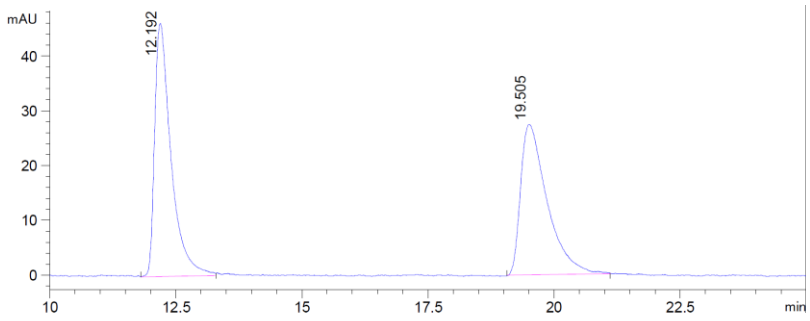

Signal 2: MWD1 B, Sig=230,4 Ref=off

Peak #	RT [min]	Type	Height	Width [min]	Area %	Area
1	12.192	BV	46.134	0.329	50.861	1.040e3
2	19.505	MM	27.468	0.610	49.139	1.005e3

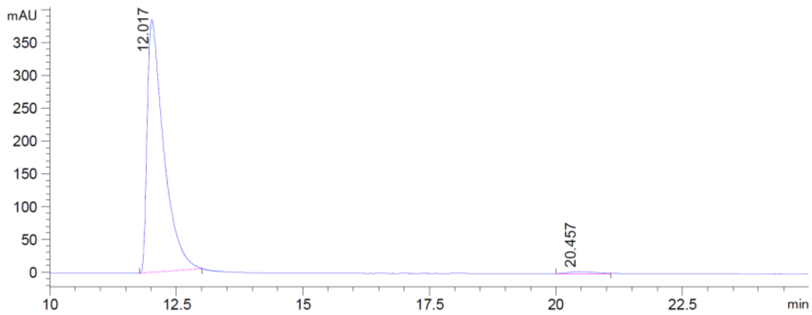

Signal 2: MWD1 B, Sig=230,4 Ref=off

Peak #	RT [min]	Type	Height	Width [min]	Area %	Area
1	12.017	MM	384.753	0.378	98.706	8.721e3
2	20.457	MM	3.150	0.605	1.294	114.328

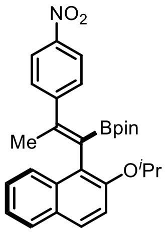

(R,E)-2-(1-(2-isopropoxynaphthalen-1-yl)-2-(4-nitrophenyl)prop-1-en-1-yl)-4,4,5,5-tetramethyl-1,3,2-dioxaborolane (4m)

Prepared according to the general procedure. 38.4 mg, 81% yield, 94% ee; R_f = 0.4 (PE/EA = 10/1); yellow solid, m. p. 132 – 134 °C, $[\alpha]^{20}_{D}$ = +61.1 (c = 0.036, $CHCl_3$). ^{1}H NMR (300 MHz, $CDCl_3$) δ 8.26 – 8.21 (m, 2H), 7.83 – 7.79 (m, 2H), 7.75 (d, *J* = 8.9 Hz, 1H), 7.60 – 7.56 (m, 2H), 7.45 – 7.32 (m, 2H), 7.28 (d, *J* = 8.9 Hz, 1H), 4.59 (hept, *J* = 6.0 Hz, 1H), 1.79 (s, 3H), 1.36 (d, *J* = 6.1 Hz, 3H), 1.32 (d, *J* = 6.0 Hz, 3H), 0.98 (s, 6H), 0.96 (s, 6H) ppm. ^{13}C NMR (75 MHz, $CDCl_3$) δ 152.7, 152.1, 150.4, 146.9, 132.8, 129.8, 129.1, 128.3, 128.1, 127.5, 126.2, 125.1, 123.8, 123.3, 118.6, 83.3, 72.7, 24.6, 24.4, 23.0, 22.8, 22.5 ppm. HRMS (ESI) *m/z* calcd for $[C_{28}H_{32}BNO_5+H]^+$ 474.2446, found 474.2451. IR (neat, cm^{-1}) 3061, 2928, 1594, 1519, 1373, 1344, 1225, 1142, 1106, 851. HPLC: Lux Cellulose-1, n-hexane/isopropanol 99.7/0.3, flow rate = 0.5 mL/min, uv-vis λ = 230 nm, t_{R1} = 19.4 min (major), t_{R2} = 22.6 min (minor).

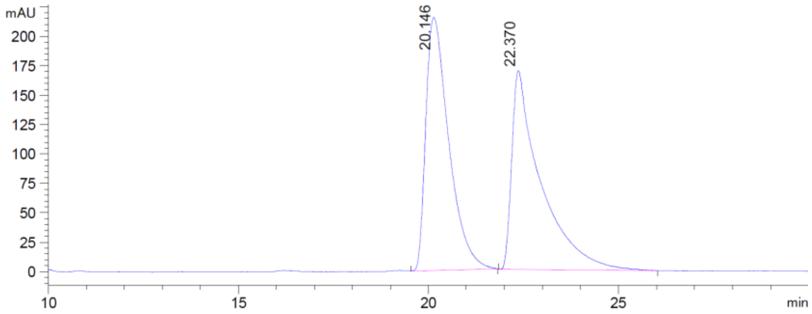

Signal 2: MWD1 B, Sig=230,4 Ref=off

Peak #	RT [min]	Type	Height	Width [min]	Area %	Area
1	20.146	MM	215.148	0.702	50.338	9.062e3
2	22.370	MM	168.819	0.883	49.662	8.941e3

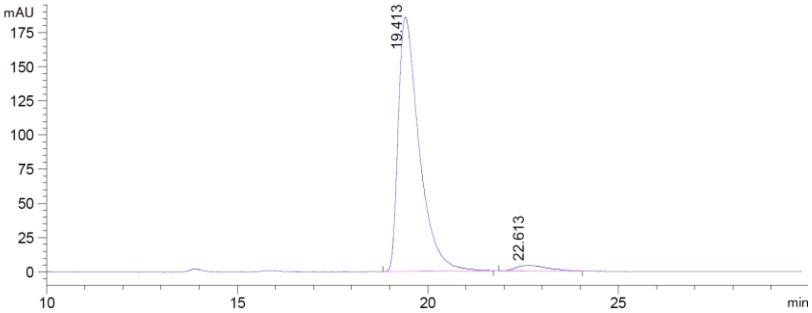

Signal 2: MWD1 B, Sig=230,4 Ref=off

Peak #	RT [min]	Type	Height	Width [min]	Area %	Area
1	19.413	MM	185.414	0.635	96.902	7.069e3
2	22.613	MM	4.146	0.908	3.098	225.975

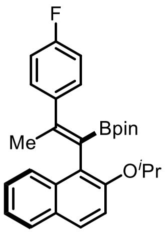

(R,E)-2-(2-(4-fluorophenyl)-1-(2-isopropoxynaphthalen-1-yl)prop-1-en-1-yl)-4,4,5,5-tetramethyl-1,3,2-dioxaborolane (4n)

Prepared according to the general procedure. 40.0 mg, 90% yield, 93% ee; R_f = 0.4 (PE/EA = 20/1); white solid, m. p. 66 – 68 °C, $[\alpha]^{20}_{D}$ = +35.0 (c = 0.04, $CHCl_3$). ^1H NMR (300 MHz, $CDCl_3$) δ 7.87 (d, J = 8.2 Hz, 1H), 7.79 (d, J = 7.7 Hz, 1H), 7.72 (d, J = 8.9 Hz, 1H), 7.44 – 7.38 (m, 3H), 7.36 – 7.31 (m, 1H), 7.253 – 7.245 (m, 1H), 7.08 – 7.02 (m, 2H), 4.57 (hept, J = 6.3 Hz, 1H), 1.76 (s, 3H), 1.35 (d, J = 6.1 Hz, 3H), 1.30 (d, J = 6.0 Hz, 3H), 1.00 (s, 6H), 0.98 (s, 6H) ppm. ^{13}C NMR (75 MHz, $CDCl_3$) δ 162.4 (d, $^1J_{C\text{-}F}$ = 243.6 Hz), 152.2, 151.2, 141.7 (d, $^4J_{C\text{-}F}$ = 3.2 Hz), 133.2, 129.9, 129.7 (d, $^3J_{C\text{-}F}$ = 8.0 Hz), 128.6, 128.2, 127.7, 126.0, 125.6, 123.7, 119.2, 114.7 (d, $^2J_{C\text{-}F}$ = 21.1 Hz), 83.0, 72.9, 24.7, 24.5, 23.2, 22.8, 22.5 ppm. ^{19}F NMR (282 MHz, $CDCl_3$) δ -116.00 ppm. HRMS (ESI) *m/z* calcd for $[C_{28}H_{32}BFO_3+H]^+$ 447.2501, found 447.2497. IR (neat, cm^{-1}) 3063, 2977, 1601, 1507, 1379, 1371, 1264, 1145, 1012, 836. HPLC: Daicel Chiralcel IC-3, n-hexane/isopropanol 98/2, flow rate = 0.5 mL/min, uv-vis λ = 230 nm, t_{R1} = 6.8 min (minor), t_{R2} = 7.0 min (major).

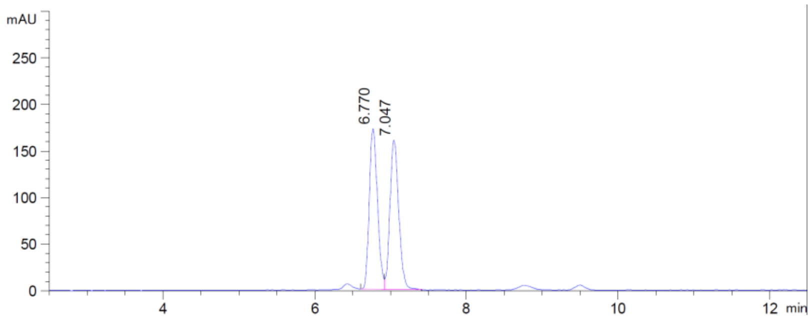

Signal 2: MWD1 B, Sig=230,4 Ref=off

Peak #	RT [min]	Type	Height	Width [min]	Area %	Area
1	6.770	VV	173.366	0.116	50.033	1.315e3
2	7.047	VB	161.067	0.125	49.967	1.313e3

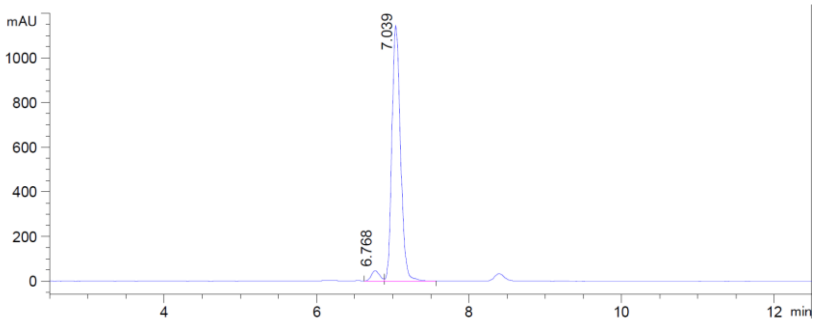

Signal 2: MWD1 B, Sig=230,4 Ref=off

Peak #	RT [min]	Type	Height	Width [min]	Area %	Area
1	6.768	BV	45.432	0.116	3.604	334.673
2	7.039	VB	1.143e3	0.121	96.396	8.952e3

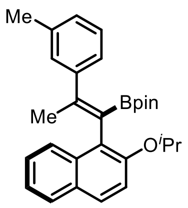

(R,E)-2-(1-(2-isopropoxynaphthalen-1-yl)-2-(m-tolyl)prop-1-en-1-yl)-4,4,5,5-tetramethyl-1,3,2-dioxaborolane (4o)

Prepared according to the general procedure. 30.6 mg, 69% yield, 91% ee; R_f = 0.4 (PE/EA = 20/1); colorless oil, $[\alpha]^{20}_D$ = +44.7 (c = 0.038, $CHCl_3$). ^{1}H NMR (300 MHz, $CDCl_3$) δ 7.93 – 7.90 (m, 1H), 7.79 – 7.77 (m, 1H), 7.71 (d, J = 8.9 Hz, 1H), 7.42 – 7.37 (m, 1H), 7.35 – 7.30 (m, 1H), 7.27 – 7.22 (m, 4H), 7.14 – 7.11 (m, 1H), 4.58 (hept, J = 5.8 Hz, 1H), 2.40 (s, 3H), 1.79 (s, 3H), 1.36 (d, J = 6.1 Hz, 3H), 1.30 (d, J = 6.0 Hz, 3H), 1.00 (s, 6H), 0.98 (s, 6H) ppm. ^{13}C NMR (75 MHz, $CDCl_3$) δ 152.23, 152.20, 145.5, 137.2, 133.3, 130.0, 129.1, 129.0, 128.1, 128.02, 127.96, 127.6, 125.9, 125.8, 125.3, 123.7, 119.5, 82.9, 73.0, 24.7, 24.5, 22.9, 22.8, 22.5, 21.7 ppm. HRMS (ESI) m/z calcd for $[C_{29}H_{35}BO_3+Na]^+$ 465.2571, found 465.2574. IR (neat, cm^{-1}) 3057, 2964, 1591, 1507, 1379, 1371, 1264, 1145, 1112, 786, 705. HPLC: Daicel Chiralcel IC-3, n-hexane/isopropanol 99/1, flow rate = 0.5 mL/min, uv-vis λ = 230 nm, t_{R1} = 6.9 min (minor), t_{R2} = 7.3 min (major).

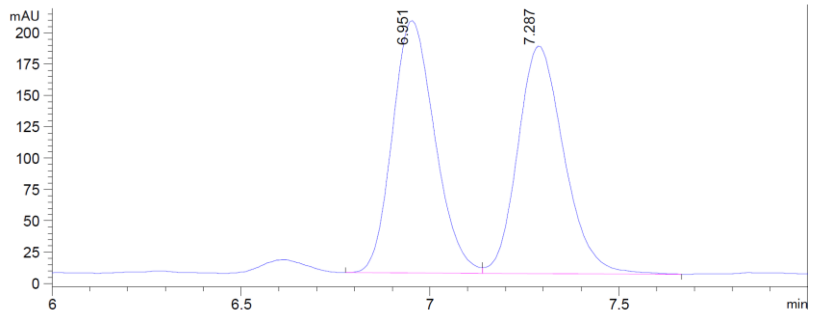

Signal 2: MWD1 B, Sig=230,4 Ref=off

Peak #	RT [min]	Type	Height	Width [min]	Area %	Area
1	6.951	BV	201.282	0.121	50.471	1.570e3
2	7.287	VB	181.647	0.131	49.529	1.540e3

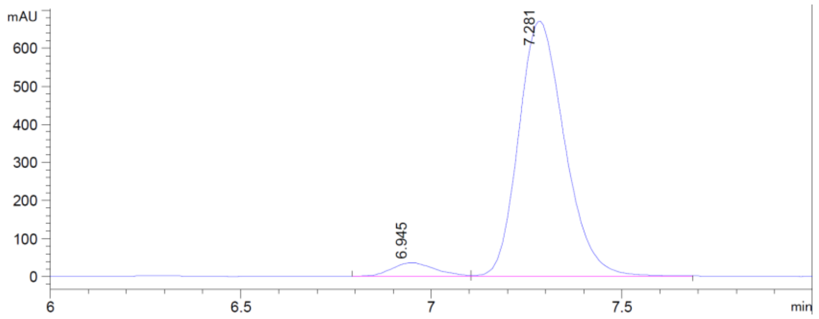

Signal 2: MWD1 B, Sig=230,4 Ref=off

Peak #	RT [min]	Type	Height	Width [min]	Area %	Area
1	6.945	MF	35.310	0.132	4.743	279.124
2	7.281	FM	670.776	0.139	95.257	5.605e3

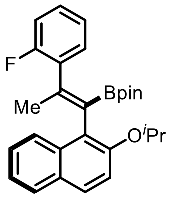

(R,E)-2-(2-(2-fluorophenyl)-1-(2-isopropoxynaphthalen-1-yl)prop-1-en-1-yl)-4,4,5,5-tetramethyl-1,3,2-dioxaborolane (4p)

Prepared according to the general procedure. 39.2 mg, 88% yield, 92% ee; R_f = 0.4 (PE/EA = 20/1); colorless oil, $[\alpha]^{20}_{D}$ = +91.1 (c = 0.056, $CHCl_3$). ^{1}H NMR (300 MHz, $CDCl_3$) δ 7.93 (d, J = 8.5 Hz, 1H), 7.79 (d, J = 8.0 Hz, 1H), 7.73 (d, J = 8.8 Hz, 1H), 7.46 – 7.41 (m, 1H), 7.39 – 7.30 (m, 2H), 7.27 – 7.23 (m, 2H), 7.15 – 7.03 (m, 2H), 4.55 (hept, J = 6.0 Hz, 1H), 1.75 (s, 3H), 1.35 (d, J = 6.1 Hz, 3H), 1.32 (d, J = 6.1 Hz, 3H), 0.96 (s, 6H), 0.94 (s, 6H) ppm. ^{13}C NMR (75 MHz, $CDCl_3$) δ 160.0 (d, $^1J_{C-F}$ = 243.0Hz), 152.2, 147.0, 133.4 (d, $^2J_{C-F}$ = 16.2 Hz), 133.1, 130.7 (d, $^3J_{C-F}$ = 4.3 Hz), 130.0, 128.5, 128.4 (d, $^3J_{C-F}$ = 7.9 Hz), 128.1, 127.7, 126.0, 125.7, 123.8, 123.5 (d, $^4J_{C-F}$ = 3.5 Hz), 119.6, 115.2 (d, $^2J_{C-F}$ = 22.3 Hz), 82.8, 73.2, 24.6, 24.4, 23.1, 22.7, 22.5 ppm. ^{19}F NMR (282 MHz, $CDCl_3$) δ -115.84 ppm. HRMS (ESI) *m/z* calcd for $[C_{28}H_{32}BFO_3+H]^+$ 447.2501, found 447.2512. IR (neat, cm^{-1}) 3061, 2977, 1591, 1507, 1380, 1371, 1265, 1146, 1112, 752. HPLC: Daicel Chiralcel IC-3, n-hexane/isopropanol 99/1, flow rate = 0.5 mL/min, uv-vis λ = 254 nm, t_{R1} = 6.9 min (minor), t_{R2} = 7.7 min (major).

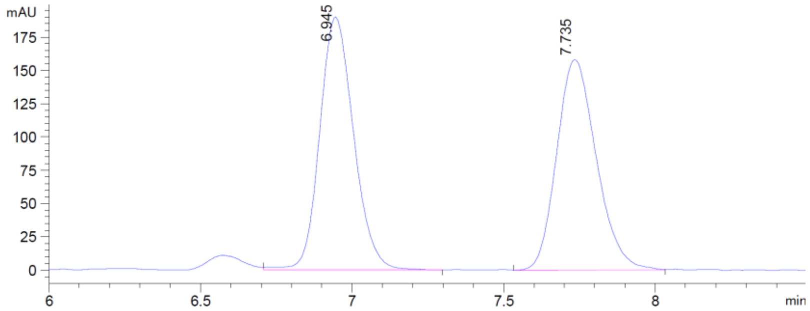

Signal 2: MWD1 B, Sig=230,4 Ref=off

Peak #	RT [min]	Type	Height	Width [min]	Area %	Area
1	6.945	VB	190.000	0.121	50.758	1.481e3
2	7.735	BV	158.125	0.140	49.242	1.437e3

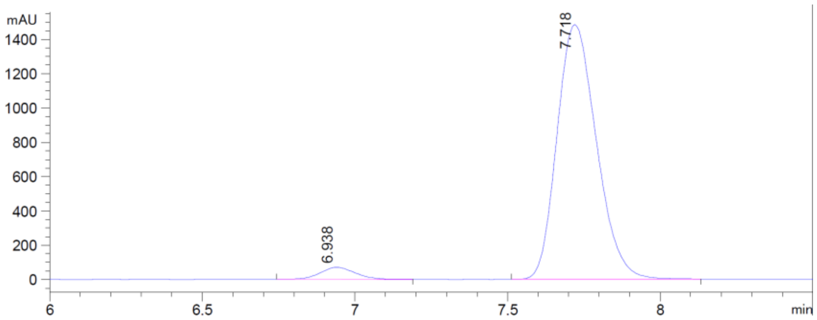

Signal 2: MWD1 B, Sig=230,4 Ref=off

Peak #	RT [min]	Type	Height	Width [min]	Area %	Area
1	6.938	VV	70.071	0.125	4.083	568.782
2	7.718	VV	1.485e3	0.141	95.917	1.336e4

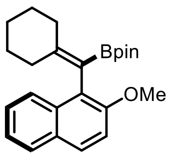

(R)-2-(cyclohexylidene(2-methoxynaphthalen-1-yl)methyl)-4,4,5,5-tetramethyl-1,3,2-dioxaborolane (4q)

Prepared according to the general procedure. 30.0 mg, 79% yield, 88% ee; R_f = 0.4 (PE/EA = 20/1); colorless oil, $[\alpha]^{20}_{D}$ = -26.0 (c = 0.05, $CHCl_3$). ^1H NMR (300 MHz, $CDCl_3$) δ 7.83 (d, J = 8.4 Hz, 1H), 7.76 – 7.70 (m, 2H), 7.40 – 7.34 (m, 1H), 7.31 – 7.24 (m, 2H), 3.87 (s, 3H), 2.90 – 2.82 (m, 1H), 2.77 – 2.68 (m, 1H), 1.86 – 1.65 (m, 4H), 1.61 – 1.50 (m, 2H), 1.43 – 1.35 (m, 2H), 1.18 (s, 6H), 1.17 (s, 6H) ppm. ^{13}C NMR (75 MHz, $CDCl_3$) δ 158.8, 153.7, 133.6, 129.3, 128.0, 127.2, 126.5, 125.9, 125.6, 123.2, 114.1, 82.8, 56.9, 34.7, 33.5, 29.0, 27.7, 26.9, 24.9, 24.6 ppm. HRMS (ESI) *m/z* calcd for $[C_{24}H_{31}BO_3+H]^+$ 379.2439, found 379.2445. IR (neat, cm^{-1}) 3054, 2927, 1591, 1508, 1350, 1267, 1147, 1092. HPLC: Daicel Chiralcel OJ-H, n-hexane/isopropanol 95/5, flow rate = 0.5 mL/min, uv-vis λ = 230 nm, t_{R1} = 24.5 min (minor), t_{R2} = 35.6 min (major).

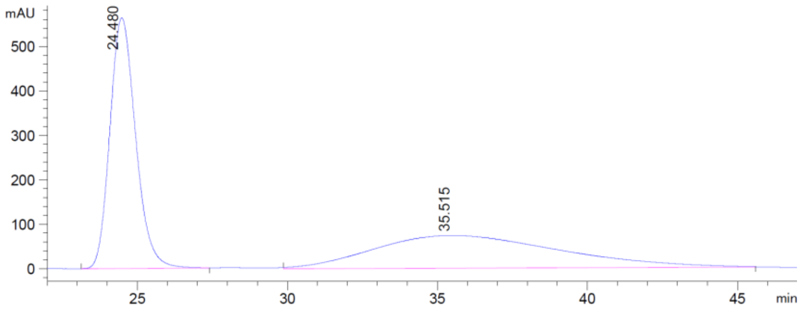

Signal 2: MWD1 B, Sig=230,4 Ref=off

Peak #	RT [min]	Type	Height	Width [min]	Area %	Area
1	24.480	BV	563.208	0.925	51.425	3.357e4
2	35.515	MM	73.564	7.184	48.575	3.171e4

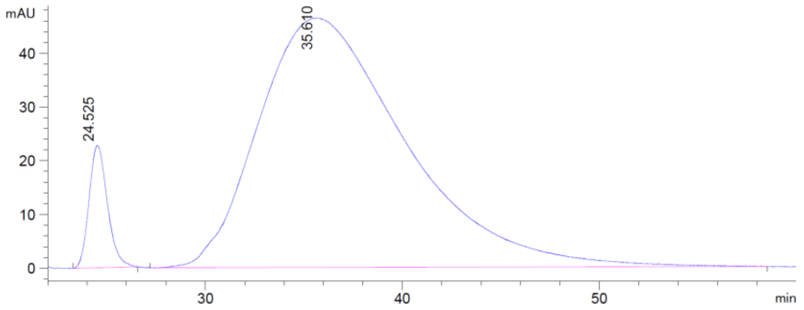

Signal 2: MWD1 B, Sig=230,4 Ref=off

Peak #	RT [min]	Type	Height	Width [min]	Area %	Area
1	24.525	BB	22.772	0.792	5.804	1.479e3
2	35.610	MM	46.397	8.622	94.196	2.400e4

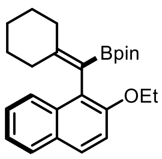

(R)-2-(cyclohexylidene(2-ethoxynaphthalen-1-yl)methyl)-4,4,5,5-tetramethyl-1,3,2-dioxaborolane (4r)

Prepared according to the general procedure. 32.5 mg, 83% yield, 94% ee; R_f = 0.4 (PE/EA = 20/1); colorless oil, $[\alpha]^{20}_D$ = -30.4 (c = 0.046, $CHCl_3$). ^{1}H NMR (300 MHz, $CDCl_3$) δ 7.80 (d, J = 8.4 Hz, 1H), 7.76 – 7.73 (m, 1H), 7.68 (d, J = 8.9 Hz, 1H), 7.39 – 7.33 (m, 1H), 7.31 – 7.22 (m, 2H), 4.08 (q, J = 7.0 Hz, 2H), 2.87 – 2.71 (m, 2H), 1.79 – 1.71 (m, 4H), 1.59 – 1.52 (m, 2H), 1.43 – 1.34 (m, 5H), 1.18 (s, 6H),1.15 (s, 6H) ppm. ^{13}C NMR (75 MHz, $CDCl_3$) δ 158.8, 153.1, 133.8, 129.6, 128.1, 127.9, 127.1, 125.9, 125.4, 123.3, 116.8, 82.7, 65.8, 34.6, 33.6, 29.1, 27.9, 26.9, 24.9, 24.7, 15.5 ppm. HRMS (ESI) *m/z* calcd for $[C_{25}H_{33}BO_3+H]^+$ 393.2596, found 393.2600. IR (neat, cm^{-1}) 3061, 2977, 1591, 1508, 1371, 1348, 1267, 1146, 1053, 748. HPLC: Daicel Chiralcel OD-H, n-hexane/isopropanol 99.9/0.1, flow rate = 0.5 mL/min, uv-vis λ = 230 nm, t_{R1} = 18.4 min (major), t_{R2} = 24.4 min (minor).

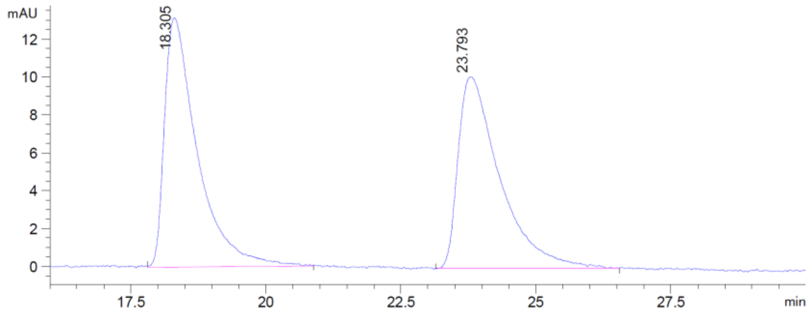

Signal 2: MWD1 B, Sig=230,4 Ref=off

Peak #	RT [min]	Type	Height	Width [min]	Area %	Area
1	18.305	MM	13.154	0.706	50.639	557.503
2	23.793	MM	10.105	0.896	49.361	543.424

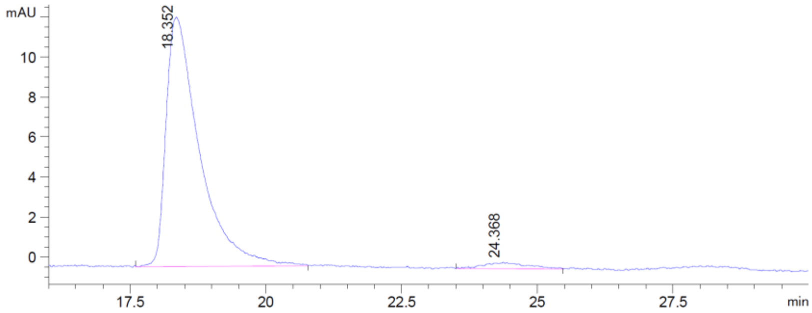

Signal 2: MWD1 B, Sig=230,4 Ref=off

Peak #	RT [min]	Type	Height	Width [min]	Area %	Area
1	18.352	MM	12.457	0.715	96.888	534.754
2	24.368	MM	0.314	0.910	3.112	17.173

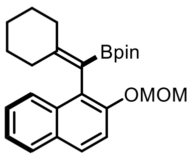

(R)-2-(cyclohexylidene(2-(methoxymethoxy)naphthalen-1-yl)methyl)-4,4,5,5-tetramethyl-1,3,2-dioxaborolane (4s)

Prepared according to the general procedure. 27.1 mg, 66% yield, 94% ee; R_f = 0.4 (PE/EA=10/1); colorless oil, $[\alpha]^{20}_D$ = +5.6 (c = 0.036, $CHCl_3$). ^1H NMR (300 MHz, $CDCl_3$) δ 7.84 – 7.81 (m, 1H), 7.77 – 7.74 (m, 1H), 7.68 (d, *J* = 8.9 Hz, 1H), 7.40 – 7.29 (m, 3H), 5.20 (d, *J* = 6.5 Hz, 1H), 5.12 (d, *J* = 6.6 Hz, 1H), 3.52 (s, 3H), 2.88 – 2.70 (m, 2H), 1.80 –1.71 (m, 4H), 1.60 – 1.51 (m, 2H), 1.43 – 1.35 (m, 2H), 1.17 (s, 6H), 1.15 (s, 6H) ppm. ^{13}C NMR (75 MHz, $CDCl_3$) δ 159.2, 151.4, 133.7, 130.1, 128.3, 128.0, 127.2, 126.0, 125.5, 123.8, 118.1, 96.0, 82.8, 56.2, 34.6, 33.6, 29.1, 27.9, 26.9, 24.8, 24.7 ppm. HRMS (ESI) *m/z* calcd for $[C_{25}H_{33}BO_4+Na]^+$ 431.2364, found 431.2375. IR (neat, cm^{-1}) 3059, 2977, 1592, 1508, 1379, 1371, 1239, 1147, 1035, 749. HPLC: Daicel Chiralcel OD-H, n-hexane/isopropanol 99.9/0.1, flow rate = 0.5 mL/min, uv-vis λ = 230 nm, t_{R1} = 18.3 min (major), t_{R2} = 19.9 min (minor).

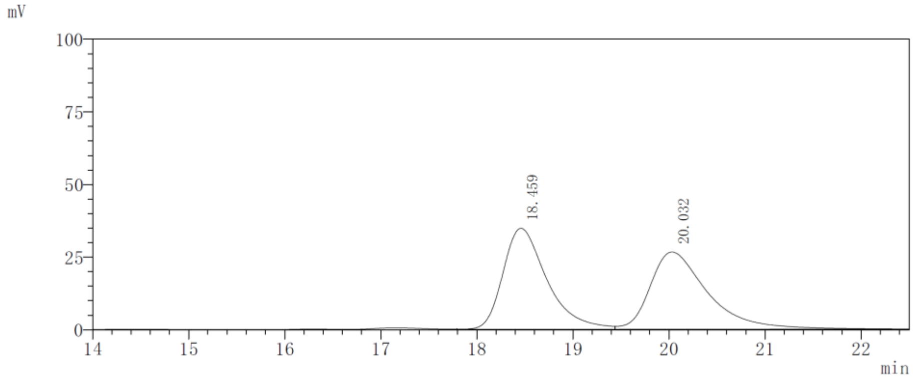

A Ch2 230nm

RetTime[min]	Area	Hight	Area%
18.459	1108356	34777	49.964
20.032	1109954	26596	50.036

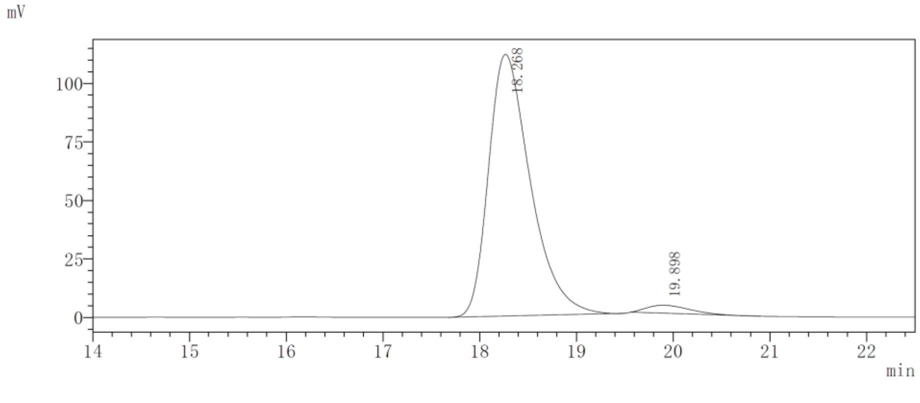

A Ch2 230nm

RetTime[min]	Area	Hight	Area%
18.268	3354842	111818	96.965
19.898	105018	3394	3.035

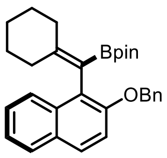

(R)-2-((2-(benzyloxy)naphthalen-1-yl)(cyclohexylidene)methyl)-4,4,5,5-tetramethyl-1,3,2-dioxaborolane (4t)

Prepared according to the general procedure. 29.1 mg, 64% yield, 95% ee; R_f = 0.4 (PE/EA = 20/1); colorless oil, $[\alpha]^{20}_{D}$ = -2.3 (c = 0.064, $CHCl_3$). ^1H NMR (300 MHz, $CDCl_3$) δ 7.83 (d, J = 8.4 Hz, 1H), 7.76 – 7.73 (m, 1H), 7.66 (d, J = 8.9 Hz, 1H), 7.51 – 7.47 (m, 2H), 7.41 – 7.21 (m, 6H), 5.13 (s, 2H), 2.95 – 2.87 (m, 1H), 2.80 –2.71 (m, 1H), 1.87 – 1.68 (m, 4H), 1.58 – 1.50 (m, 2H), 1.45 – 1.31 (m, 2H), 1.14 (s, 6H), 1.10 (s, 6H) ppm. ^{13}C NMR (75 MHz, $CDCl_3$) δ 159.5, 152.8, 138.4, 133.8, 129.7, 128.35, 128.28, 128.0, 127.5, 127.2, 127.1, 125.9, 125.6, 123.5, 116.9, 82.8, 71.8, 34.6, 33.7, 29.1, 28.0, 26.9, 24.9, 24.7 ppm. HRMS (ESI) *m/z* calcd for $[C_{30}H_{35}BO_3+Na]^+$ 477.2571, found 477.2586. IR (neat, cm^{-1}) 3061, 2977, 1591, 1508, 1349, 1268, 1146, 1091, 749. HPLC: Daicel Chiralcel OD-H, n-hexane/isopropanol 99.9/0.1, flow rate = 0.5 mL/min, uv-vis λ = 230 nm, t_{R1} = 27.2 min (major), t_{R2} = 35.7 min (minor).

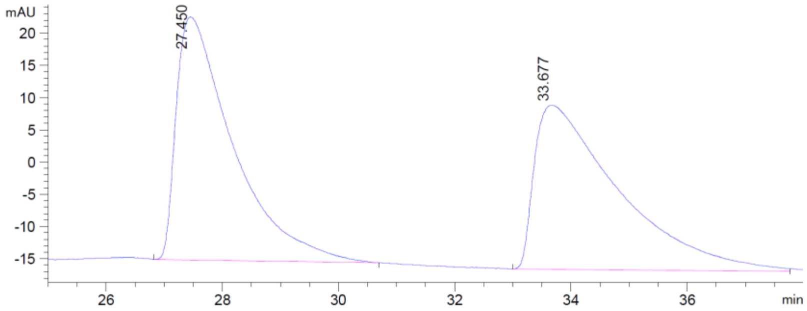

Signal 2: MWD1 B, Sig=230,4 Ref=off

Peak #	RT [min]	Type	Height	Width [min]	Area %	Area
1	27.450	BB	37.712	0.917	49.972	2.563e3
2	33.677	BV	25.440	1.191	50.028	2.566e3

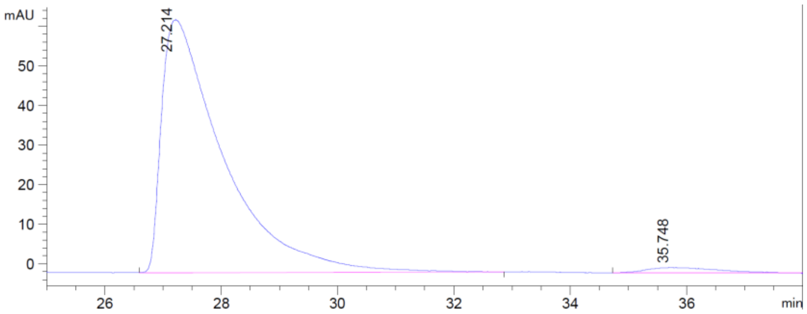

Signal 2: MWD1 B, Sig=230,4 Ref=off

Peak #	RT [min]	Type	Height	Width [min]	Area %	Area
1	27.214	MM	63.883	1.234	97.370	4.729e3
2	35.748	MM	1.402	1.518	2.630	127.752

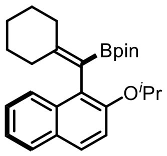

(R)-2-(cyclohexylidene(2-isopropoxynaphthalen-1-yl)methyl)-4,4,5,5-tetramethyl-1,3,2-dioxaborolane (4u)

Prepared according to the general procedure. 36.0 mg, 89% yield, 97% ee; R_f = 0.4 (PE/EA = 20/1); colorless oil, $[\alpha]^{20}_D$ = -25.9 (c = 0.054, $CHCl_3$). ^{1}H NMR (300 MHz, $CDCl_3$) δ 7.80 – 7.73 (m, 2H), 7.66 (d, J = 8.8 Hz, 1H), 7.39 – 7.27 (m, 2H), 7.20 (d, J = 8.8 Hz, 1H), 4.43 (hept, J = 6.1 Hz, 1H), 2.93 – 2.85 (m, 1H), 2.74 – 2.65 (m, 1H), 1.85 – 1.65 (m, 4H), 1.60 – 1.51 (m, 2H), 1.45 – 1.35 (m, 2H), 1.29 (d, J = 5.3 Hz, 3H), 1.27 (d, J = 5.3 Hz, 3H), 1.18 (s, 6H), 1.15 (s, 6H) ppm. ^{13}C NMR (75 MHz, $CDCl_3$) δ 158.8, 152.3, 133.9, 130.0, 129.9, 127.9, 126.8, 126.0, 125.4, 123.5, 119.9, 82.7, 72.9, 34.5, 33.7, 29.0, 27.7, 26.9, 24.9, 24.7, 22.72, 22.67 ppm. HRMS (ESI) *m/z* calcd for $[C_{26}H_{35}BO_3+Na]^+$ 429.2571, found 429.2580. IR (neat, cm^{-1}) 3061, 2976, 1620, 1591, 1507, 1379, 1370, 1226, 1147, 1116, 750. HPLC: Daicel Chiralcel OD-H, n-hexane/isopropanol 99.9/0.1, flow rate = 0.5 mL/min, uv-vis λ = 230 nm, t_{R1} = 12.6 min (major), t_{R2} = 16.2 min (minor).

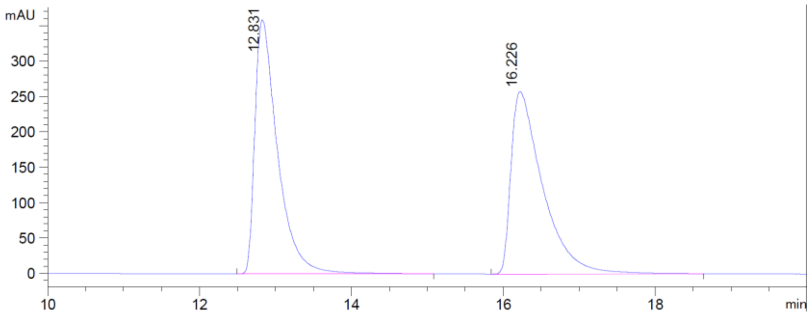

Signal 2: MWD1 B, Sig=230,4 Ref=off

Peak #	RT [min]	Type	Height	Width [min]	Area %	Area
1	12.831	BV	359.152	0.309	49.996	7.490e3
2	16.226	BB	257.681	0.423	50.004	7.491e3

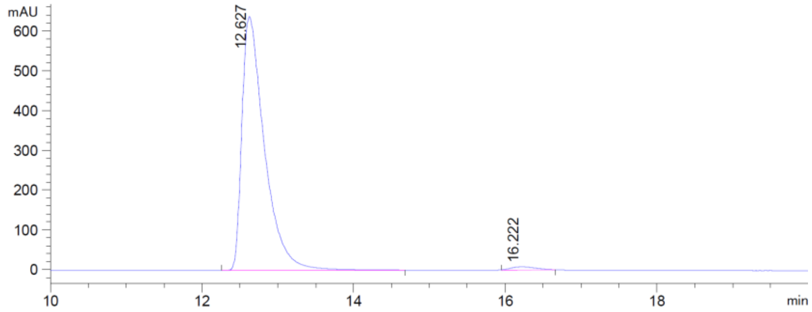

Signal 2: MWD1 B, Sig=230,4 Ref=off

Peak #	RT [min]	Type	Height	Width [min]	Area %	Area
1	12.627	BV	637.419	0.309	98.480	1.319e4
2	16.222	MM	8.673	0.391	1.520	203.463

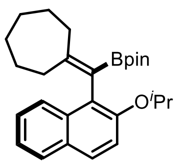

(R)-2-(cycloheptylidene(2-isopropoxynaphthalen-1-yl)methyl)-4,4,5,5-tetramethyl-1,3,2-dioxaborolane (4v)

Prepared according to the general procedure. 26.3 mg, 63% yield, 97% ee; R_f = 0.4 (PE/EA = 20/1); white solid, m. p. 58 – 60 °C, $[\alpha]^{20}_{D}$ = -23.7 (c = 0.038, $CHCl_3$). ^1H NMR (300 MHz, $CDCl_3$) δ 7.78 – 7.73 (m, 2H), 7.66 (d, J = 8.8 Hz, 1H), 7.37 – 7.26 (m, 2H), 7.20 (d, J = 8.9 Hz, 1H), 4.51 (hept, J = 6.0 Hz, 1H), 3.03 – 2.95 (m, 1H), 2.76 – 2.68 (m, 1H), 2.04 – 1.86 (m, 2H), 1.83 – 1.71 (m, 2H), 1.68 – 1.54 (m, 2H), 1.47 – 1.33 (m, 4H), 1.30 (d, J = 1.5 Hz, 3H), 1.28 (d, J = 1.5 Hz, 3H), 1.16 (s, 6H), 1.13 (s, 6H) ppm. ^{13}C NMR (75 MHz, $CDCl_3$) δ 161.7, 151.7, 133.6, 129.7, 129.6, 127.9, 126.8, 125.7, 125.4, 123.4, 118.8, 82.6, 71.8, 35.1, 34.6, 30.3, 29.6, 28.8, 26.2, 24.8 (two overlapping carbon signals), 22.8, 22.7 ppm. HRMS (ESI) m/z calcd for $[C_{27}H_{37}BO_3+H]^+$ 421.2909, found 421.2906. IR (neat, cm^{-1}) 3061, 2976, 1591, 1507, 1379, 1370, 1263, 1145, 1115, 748. HPLC: Daicel Chiralcel OD-H, n-hexane/isopropanol 99.9/0.1, flow rate = 0.5 mL/min, uv-vis λ = 230 nm, t_{R1} = 12.4 min (major), t_{R2} = 15.1 min (minor).

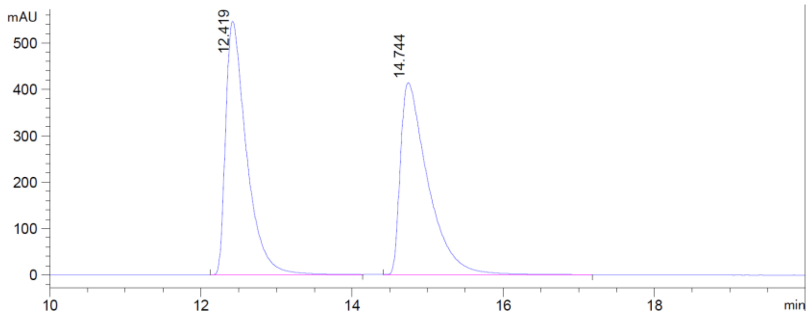

Signal 2: MWD1 B, Sig=230,4 Ref=off

Peak #	RT [min]	Type	Height	Width [min]	Area %	Area
1	12.419	BV	545.573	0.287	49.950	1.050e4
2	14.744	VB	413.843	0.371	50.050	1.052e4

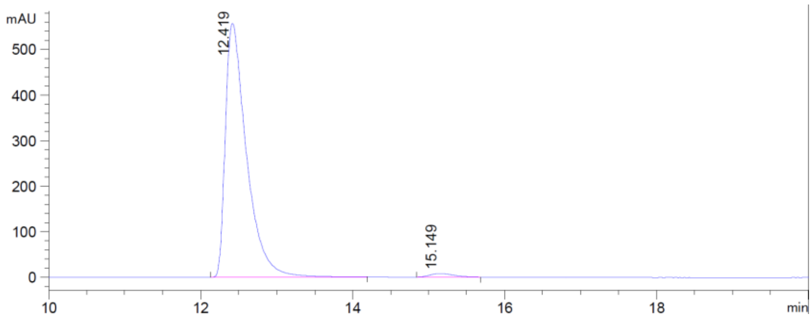

Signal 2: MWD1 B, Sig=230,4 Ref=off

Peak #	RT [min]	Type	Height	Width [min]	Area %	Area
1	12.419	BV	557.414	0.288	98.306	1.080e4
2	15.149	MM	8.200	0.378	1.694	186.058

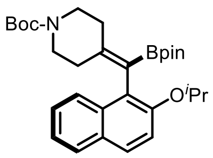

***tert*-butyl-(*R*)-4-((2-isopropoxynaphthalen-1-yl)(4,4,5,5-tetramethyl-1,3,2-dioxaborolan-2-yl)methylene)piperidine-1-carboxylate (4w)**

Prepared according to the general procedure. 43.1 mg, 85% yield, 95% ee; R_f = 0.4 (PE/EA = 10/1); white solid, m. p. 68 – 70 °C, $[\alpha]^{20}_D$ = +15.0 (c = 0.02, $CHCl_3$). ^{1}H NMR (300 MHz, $CDCl_3$) δ 7.75 (d, *J* = 8.0 Hz, 1H), 7.71 – 7.66 (m, 2H), 7.38 – 7.27 (m, 2H), 7.21 (d, *J* = 8.9 Hz, 1H), 4.47 (hept, *J* = 5.9 Hz, 1H), 3.77 – 3.69 (m, 1H), 3.50 – 3.37 (m, 2H), 3.24 – 3.16 (m, 1H), 3.07 – 2.99 (m, 1H), 2.83 – 2.75 (m, 1H), 1.94 – 1.79 (m, 2H), 1.44 (s, 9H), 1.28 (d, *J* = 4.6 Hz, 3H), 1.26 (d, *J* = 4.7 Hz, 3H), 1.18 (s, 6H), 1.15 (s, 6H) ppm. ^{13}C NMR (75 MHz, $CDCl_3$) δ 155.0, 154.0, 152.0, 133.6, 129.7, 128.5, 128.0, 127.3, 125.7, 125.5, 123.6, 119.0, 83.0, 79.4, 72.6, 33.5, 32.8, 28.6, 24.8, 24.7, 22.75, 22.71 ppm. HRMS (ESI) *m/z* calcd for $[C_{30}H_{42}BNO_5+Na]^+$ 530.3048, found 530.3050. IR (neat, cm^{-1}) 3059, 2976, 1698, 1591, 1507, 1380, 1366, 1222, 1169, 1145, 1000. HPLC: Daicel Chiralcel IC-3, n-hexane/isopropanol 99.5/0.5, flow rate = 0.5 mL/min, uv-vis λ = 230 nm, t_{R1} = 29.9 min (minor), t_{R2} = 31.6 min (major).

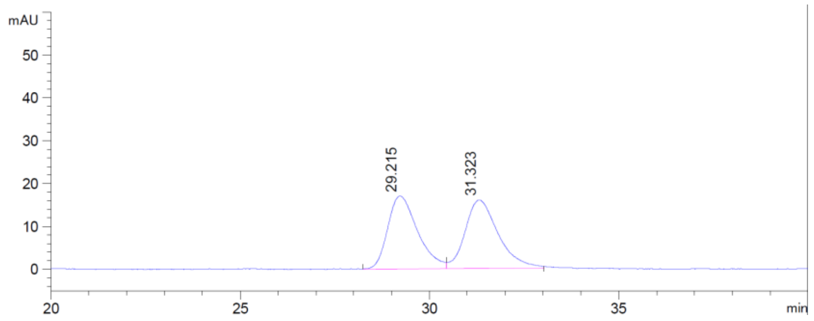

Signal 2: MWD1 B, Sig=230,4 Ref=off

Peak #	RT [min]	Type	Height	Width [min]	Area %	Area
1	29.215	BV	16.987	0.711	48.956	929.892
2	31.323	VV	15.952	0.739	51.044	969.557

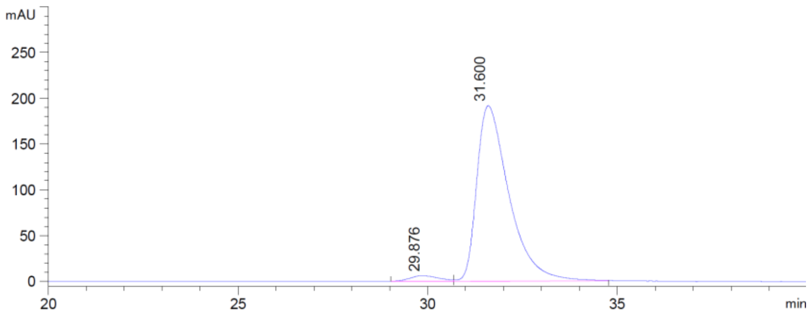

Signal 2: MWD1 B, Sig=230,4 Ref=off

Peak #	RT [min]	Type	Height	Width [min]	Area %	Area
1	29.876	BV	6.029	0.626	2.671	310.030
2	31.600	VV	191.310	0.873	97.329	1.130e4

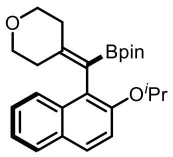

(R)-2-((2-isopropoxynaphthalen-1-yl)(tetrahydro-4H-pyran-4-ylidene)methyl)-4,4,5,5-tetramethyl-1,3,2-dioxaborolane (4x)

Prepared according to the general procedure. 32.1 mg, 79% yield, 85% ee; R_f = 0.3 (PE/EA = 10/1); colorless oil, $[\alpha]^{20}_{D}$ = -11.8 (c = 0.034, $CHCl_3$). ^1H NMR (300 MHz, $CDCl_3$) δ 7.77 – 7.72 (m, 2H), 7.68 (d, *J* = 8.9 Hz, 1 H), 7.40 – 7.28 (m, 2H), 7.21 (d, *J* = 8.9 Hz, 1H), 4.48 (hept, *J* = 6.0 Hz, 1H), 3.97 – 3.90 (m, 1H), 3.84 – 3.77 (m, 1H), 3.65 – 3.52 (m, 2H), 3.13 – 3.06 (m, 1H), 2.93 – 2.85 (m, 1H), 1.93 (t, *J* = 5.3 Hz, 2H), 1.30 (d, *J* = 4.2 Hz, 3H), 1.28 (d, *J* = 4.1 Hz, 3H), 1.18 (s, 6H), 1.15 (s, 6H) ppm. ^{13}C NMR (75 MHz, $CDCl_3$) δ 153.5, 152.1, 133.7, 129.7, 128.5, 128.0, 127.3, 125.64, 123.59, 119.1, 82.9, 72.6, 69.8, 68.8, 35.0, 34.3, 24.8, 24.7, 22.8, 22.7 ppm. HRMS (ESI) *m/z* calcd for $[C_{25}H_{33}BO_4+H]^+$ 409.2545, found 409.2536. IR (neat, cm^{-1}) 3061, 2930, 1591, 1506, 1379, 1371, 1243, 1146, 1098. HPLC: Daicel Chiralcel IC-3, n-hexane/isopropanol 97/3, flow rate = 0.5 mL/min, uv-vis λ = 230 nm, t_{R1} = 17.2 min (minor), t_{R2} = 18.5 min (major).

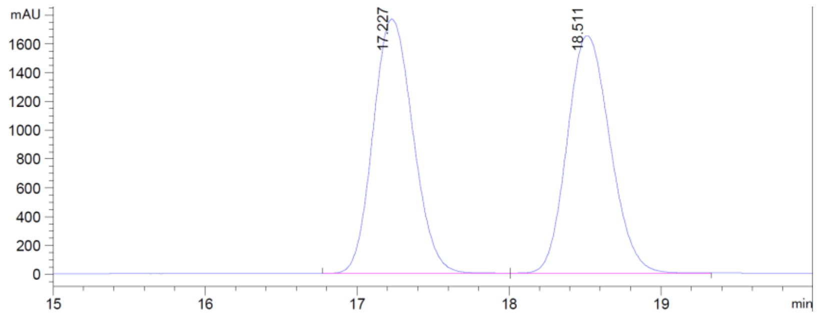

Signal 2: MWD1 B, Sig=230,4 Ref=off

Peak #	RT [min]	Type	Height	Width [min]	Area %	Area
1	17.227	BB	1.765e3	0.287	49.854	3.218e4
2	18.511	BV	1.649e3	0.309	50.146	3.237e4

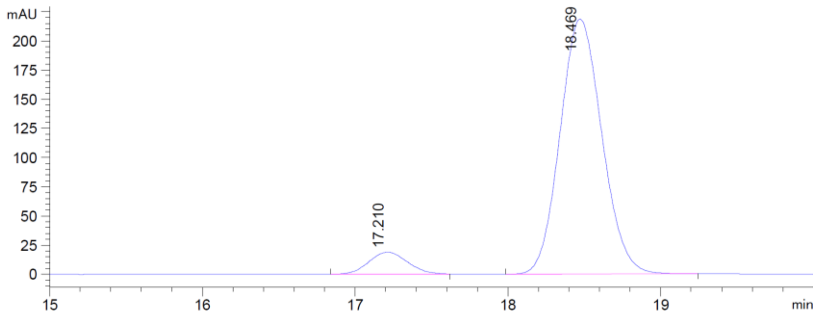

Signal 2: MWD1 B, Sig=230,4 Ref=off

Peak #	RT [min]	Type	Height	Width [min]	Area %	Area
1	17.210	VV	19.006	0.268	7.469	335.786
2	18.469	BB	218.346	0.298	92.531	4.160e3

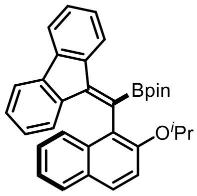

(R)-2-((9H-fluoren-9-ylidene)(2-isopropoxynaphthalen-1-yl)methyl)-4,4,5,5-tetramethyl-1,3,2-dioxaborolane (4y)

Prepared according to the general procedure. 42.5 mg, 87% yield, 92% ee; R_f = 0.4 (PE/EA = 20/1); yellow oil, $[\alpha]^{20}_{D}$ = +75.0 (c = 0.052, $CHCl_3$). ^1H NMR (300 MHz, $CDCl_3$) δ 8.35 – 8.33 (m, 1H), 7.85 – 7.77 (m, 3H), 7.69 – 7.66 (m, 1H), 7.57 – 7.54 (m, 1H), 7.39 – 7.25 (m, 4H), 7.22 – 7.17 (m, 1H), 7.09 – 7.04 (m, 1H), 6.62 – 6.57 (m, 1H), 6.05 – 6.02 (m, 1H), 4.50 (hept, *J* = 6.0 Hz, 1H), 1.30 (s, 6H), 1.28 (s, 6H), 1.26 (d, *J* = 6.1 Hz, 3H), 1.08 (d, *J* = 6.1 Hz, 3H) ppm. ^{13}C NMR (75 MHz, $CDCl_3$) δ 152.1, 146.0, 140.91, 140.87, 139.0, 138.6, 133.0, 130.0, 128.6, 128.4, 128.1, 127.9, 127.8, 126.9, 126.6, 126.3, 125.7, 125.6, 124.3, 124.0, 119.3, 119.0, 118.8, 84.0, 73.0, 25.0, 24.9, 22.64, 22.56 ppm. HRMS (ESI) *m/z* calcd for $[C_{33}H_{33}BO_3+Na]^+$ 511.2415, found 511.2407. IR (neat, cm^{-1}) 3057, 2977, 1590, 1506, 1371, 1267, 1141, 730. HPLC: Daicel Chiralcel OD-H, n-hexane/isopropanol 99.9/0.1, flow rate = 0.5 mL/min, uv-vis λ = 254 nm, t_{R1} = 25.3 min (major), t_{R2} = 33.7 min (minor).

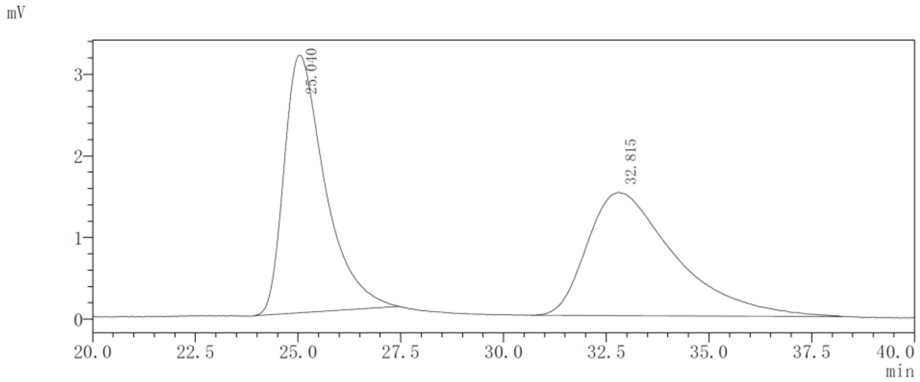

A Ch1 254nm

RetTime[min]	Area	Hight	Area%
25.040	219275	3159	49.418
32.815	224435	1509	50.582

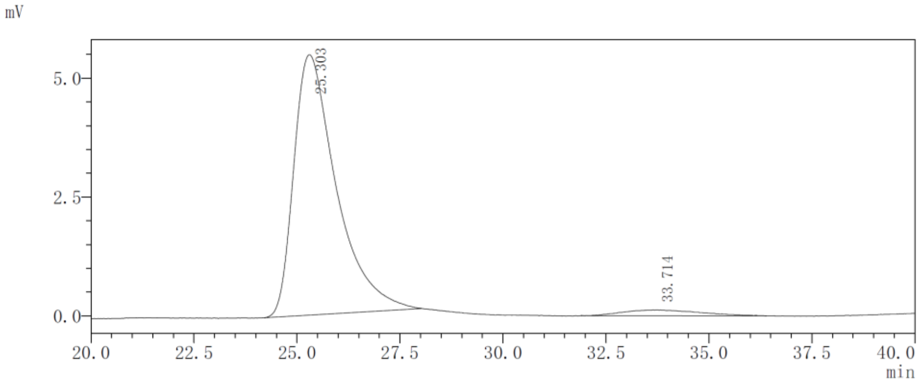

A Ch1 254nm

RetTime[min]	Area	Hight	Area%
25.303	392740	5475	96.097
33.714	15950	122	3.903

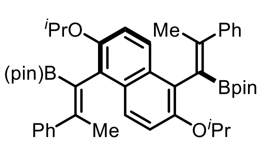

(R,R)-2,2'-((1E,1'E)-(2,6-diisopropoxynaphthalene-1,5-diyl)bis(2-phenylprop-1-ene-1,1-diyl))bis(4,4,5,5-tetramethyl-1,3,2-dioxaborolane) (4z)

Prepared according to the general procedure. 54.1 mg, 74% yield, 93% ee, 7:1 dr; R_f = 0.4 (PE/EA = 10/1); colorless oil, $[\alpha]^{20}_D$ = +142.3 (c = 0.054, $CHCl_3$). ^1H NMR (300 MHz, $CDCl_3$) δ 7.79 (d, J = 9.3 Hz, 2H), 7.47 – 7.44 (m, 4H), 7.38 – 7.28 (m, 6H), 7.16 (d, J = 9.2 Hz, 2H), 4.50 (hept, J = 5.8 Hz, 2H), 1.83 (s, 6H), 1.33 (d, J = 6.0 Hz, 6H), 1.28 (d, J = 5.9 Hz, 6H), 1.004 (s, 12H), 0.996 (s, 12H) ppm. ^{13}C NMR (75 MHz, $CDCl_3$) δ 151.8, 150.8, 145.9, 129.7, 129.1, 128.2, 128.0, 127.1, 125.4, 119.7, 82.9, 73.2, 24.7, 24.5, 23.2, 22.9, 22.5 ppm. HRMS (ESI) *m/z* calcd for $[C_{46}H_{58}B_2O_6+Na]^+$ 751.4312, found 751.4309. IR (neat, cm^{-1}) 3075, 2929, 1598, 1491, 1379, 1371, 1146, 1112, 764, 699. HPLC: Daicel Chiralcel IA-3, n-hexane/isopropanol 99.5/0.5, flow rate = 0.1 mL/min, uv-vis λ = 230 nm, t_{R1} = 34.2 min (minor), t_{R2} = 35.9 min (major).

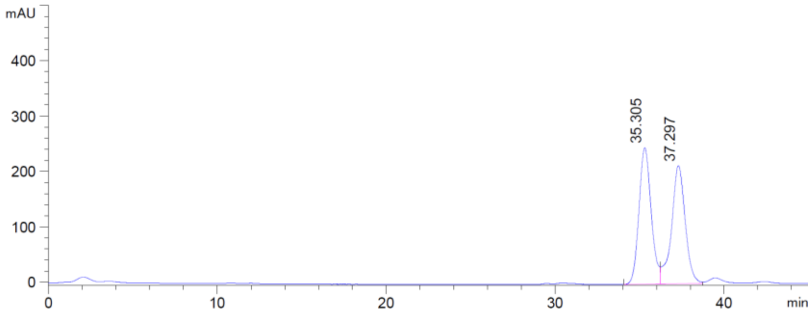

Signal 2: MWD1 B, Sig=230,4 Ref=off

Peak #	RT [min]	Type	Height	Width [min]	Area %	Area
1	35.305	BV	246.097	0.768	50.848	1.240e4
2	37.297	VV	212.961	0.848	49.152	1.198e4

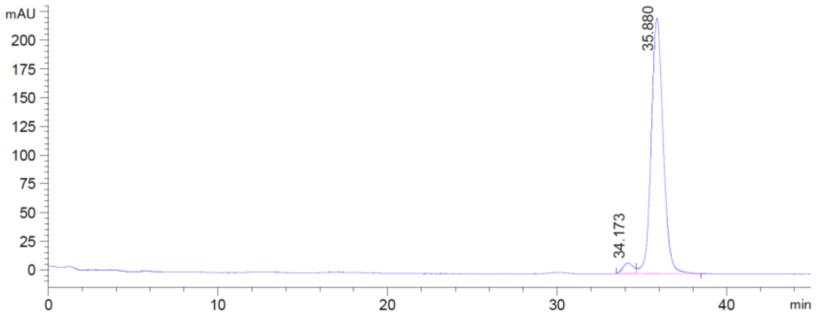

Signal 2: MWD1 B, Sig=230,4 Ref=off

Peak #	RT [min]	Type	Height	Width [min]	Area %	Area
1	34.173	MF	9.042	0.701	3.385	380.319
2	35.880	FM	222.163	0.814	96.615	1.085e4

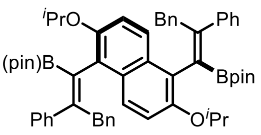

(R,R)-2,2'-((1E,1'E)-(2,6-diisopropoxynaphthalene-1,5-diyl)bis(2,3-diphenylprop-1-ene-1,1-diyl))bis(4,4,5,5-tetramethyl-1,3,2-dioxaborolane) (4aa)

Prepared according to the general procedure. 44.1 mg, 50% yield, 99% ee, >20:1 dr; R_f = 0.4 (PE/EA = 10/1); white solid, m. p. 118 – 120 °C, $[\alpha]^{20}_D$ = +5.6 (c = 0.054, $CHCl_3$). ^1H NMR (300 MHz, $CDCl_3$) δ 7.86 (d, J = 9.1 Hz, 2H), 7.31 – 7.27 (m, 4H), 7.25 – 7.16 (m, 8H), 6.99 – 6.88 (m, 10H), 4.65 (hept, J = 6.0 Hz, 2H), 3.59 – 3.47 (m, 4H), 1.41 (d, J = 4.3 Hz, 6H), 1.39 (d, J = 4.3 Hz, 6H), 0.95 (s, 12H), 0.91 (s, 12H) ppm. ^{13}C NMR (75 MHz, $CDCl_3$) δ 153.3, 150.4, 144.4, 139.6, 129.6, 129.4, 128.9, 127.7, 127.6, 126.8, 126.2, 125.42, 125.37, 117.3, 82.9, 71.1, 42.0, 24.50, 24.45, 22.9, 22.7. HRMS (ESI) *m/z* calcd for $[C_{58}H_{66}B_2O_6+Na]^+$ 903.4938, found 903.4921. IR (neat, cm^{-1}) 3063, 2929, 1598, 1495, 1379, 1371, 1245, 1145, 1114, 762, 698. HPLC: Lux-Cellulose-1, n-hexane/isopropanol 99.7/0.3, flow rate = 0.5 mL/min, uv-vis λ = 254 nm, t_{R1} = 11.9 min (minor), t_{R2} = 12.4 min (major).

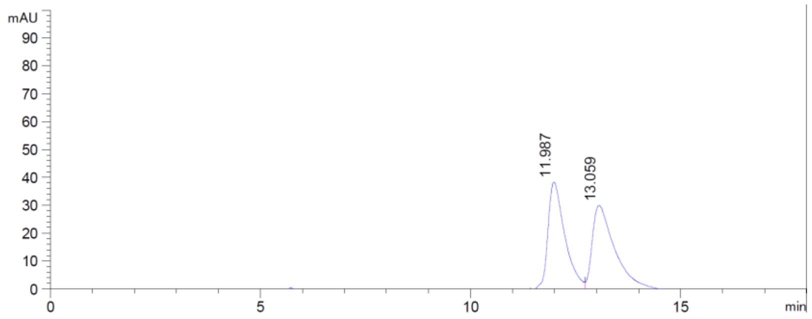

Signal 1: MWD1 A, Sig=254,4 Ref=off

Peak #	RT [min]	Type	Height	Width [min]	Area %	Area
1	11.987	BV	39.509	0.415	49.070	1.148e3
2	13.059	VV	30.874	0.534	50.930	1.192e3

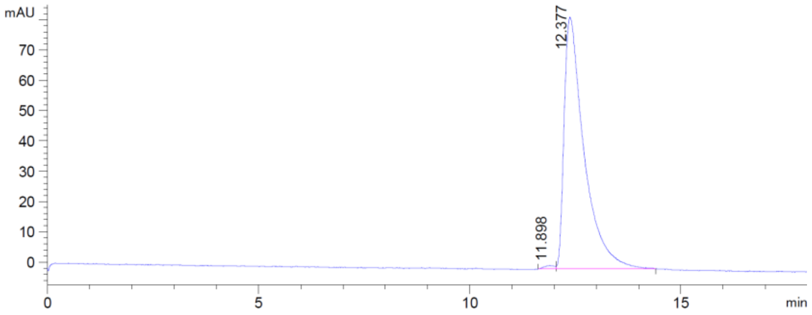

Signal 1: MWD1 A, Sig=254,4 Ref=off

Peak #	RT [min]	Type	Height	Width [min]	Area %	Area
1	11.898	MF	1.165	0.279	0.703	19.472
2	12.377	FM	83.162	0.551	99.297	2.749e3

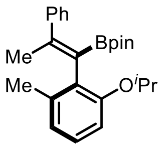

(R,E)-2-(1-(2-isopropoxy-6-methylphenyl)-2-phenylprop-1-en-1-yl)-4,4,5,5-tetramethyl-1,3,2-dioxaborolane (5a)

Prepared according to the general procedure. 33.6 mg, 86% yield, 92% ee; R_f = 0.4 (PE/EA = 20/1); colorless oil, $[\alpha]^{20}_{D}$ = +68.4 (c = 0.038, $CHCl_3$). ^{1}H NMR (300 MHz, $CDCl_3$) δ 7.39 – 7.25 (m, 5H), 7.08 (t, *J* = 7.8 Hz, 1H), 6.84 (d, *J* = 7.4 Hz, 1H), 6.76 (d, *J* = 8.1 Hz, 1H), 4.42 (hept, *J* = 6.0 Hz, 1H), 2.24 (s, 3H), 1.81 (s, 3H), 1.29 (d, *J* = 6.1 Hz, 3H), 1.26 (d, *J* = 6.1 Hz, 3H), 1.03 (s, 6H), 1.00 (s, 6H) ppm. ^{13}C NMR (75 MHz, $CDCl_3$) δ 155.1, 150.6, 145.6, 137.6, 133.3, 128.1, 127.8, 127.0, 126.5, 122.8, 113.2, 82.7, 71.1, 24.7, 24.5, 22.4, 22.3, 22.2, 20.2 ppm. HRMS (ESI) *m/z* calcd for $[C_{25}H_{33}BO_3+H]^+$ 393.2596, found 393.2602. IR (neat, cm^{-1}) 3061, 2930, 1596, 1574, 1371, 1346, 1251, 1145, 1029, 761, 700. HPLC: Daicel Chiralcel IC-3, n-hexane/isopropanol 99.5/0.5, flow rate = 0.5 mL/min, uv-vis λ = 254 nm, t_{R1} = 7.1 min (minor), t_{R2} = 7.8 min (major).

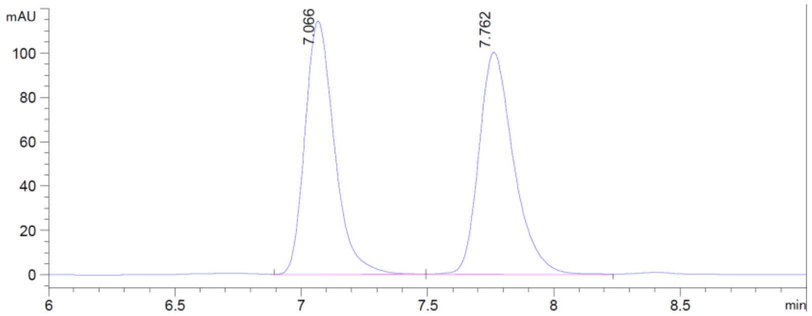

Signal 1: MWD1 A, Sig=254,4 Ref=off

Peak #	RT [min]	Type	Height	Width [min]	Area %	Area
1	7.066	VB	114.276	0.125	49.119	927.321
2	7.762	BV	100.200	0.146	50.881	960.603

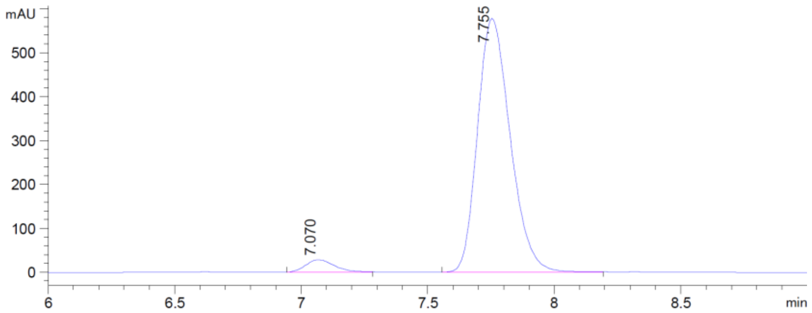

Signal 1: MWD1 A, Sig=254,4 Ref=off

Peak #	RT [min]	Type	Height	Width [min]	Area %	Area
1	7.070	MM	27.750	0.128	3.958	213.948
2	7.755	MM	578.292	0.150	96.042	5.191e3

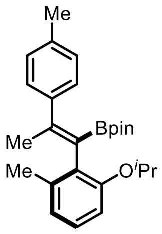

(R,E)-2-(1-(2-isopropoxy-6-methylphenyl)-2-(p-tolyl)prop-1-en-1-yl)-4,4,5,5-tetramethyl-1,3,2-dioxaborolane (5b)

Prepared according to the general procedure. 38.6 mg, 95% yield, 93% ee; R_f = 0.4 (PE/EA = 20/1); colorless oil, $[\alpha]^{20}_D$ = +98.6 (c = 0.074, $CHCl_3$). ^1H NMR (300 MHz, $CDCl_3$) δ 7.27 – 7.25 (m, 2H), 7.13 – 7.10 (m, 2H), 7.06 (d, J = 7.8 Hz, 1H), 6.84 (d, J = 7.5 Hz, 1H), 6.75 (d, J = 8.1 Hz, 1H), 4.41 (hept, J = 6.0 Hz, 1H), 2.36 (s, 3H), 2.23 (s, 3H), 1.79 (s, 3H), 1.28 (d, J = 6.1 Hz, 3H), 1.25 (d, J = 6.1 Hz, 3H), 1.04 (s, 6H), 1.01 (s, 6H) ppm. ^{13}C NMR (75 MHz, $CDCl_3$) δ 155.2, 150.3, 142.6, 137.6, 136.6, 133.6, 128.5, 128.0, 126.4, 122.9, 113.4, 82.7, 71.2, 24.7, 24.5, 22.4, 22.3, 22.1, 21.3, 20.1 ppm. HRMS (ESI) *m/z* calcd for $[C_{26}H_{35}BO_3+H]^+$ 407.2752, found 407.2766. IR (neat, cm^{-1}) 3053, 2927, 1577, 1510, 1371, 1346, 1251, 1146, 1114, 818. HPLC: Daicel Chiralcel IC-3, n-hexane/isopropanol 99.5/0.5, flow rate = 0.5 mL/min, uv-vis λ = 230 nm, t_{R1} = 6.9 min (minor), t_{R2} = 8.8 min (major).

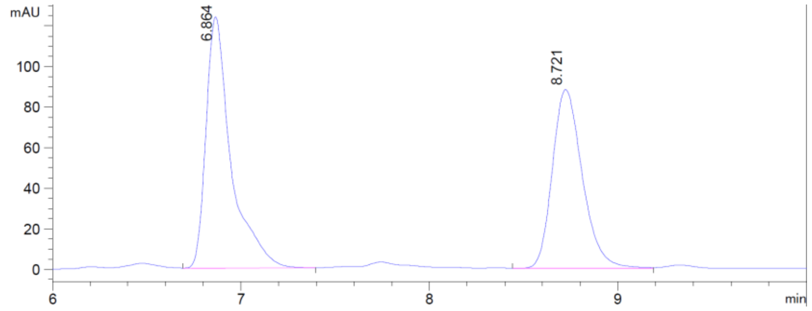

Signal 2: MWD1 B, Sig=230,4 Ref=off

Peak #	RT [min]	Type	Height	Width [min]	Area %	Area
1	6.864	BB	123.743	0.135	53.960	1.140e3
2	8.721	BV	88.127	0.171	46.040	972.865

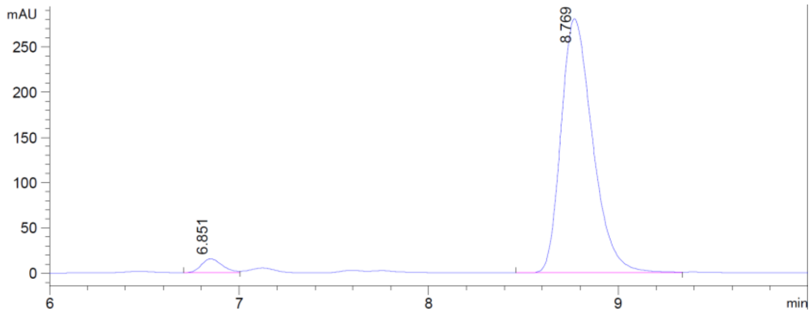

Signal 2: MWD1 B, Sig=230,4 Ref=off

Peak #	RT [min]	Type	Height	Width [min]	Area %	Area
1	6.851	VV	15.553	0.119	3.600	121.848
2	8.769	BV	280.852	0.180	96.400	3.263e3

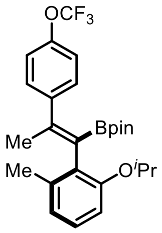

(R,E)-2-(1-(2-isopropoxy-6-methylphenyl)-2-(4-(trifluoromethoxy)phenyl)prop-1-en-1-yl)-4,4,5,5-tetramethyl-1,3,2-dioxaborolane (5c)

Prepared according to the general procedure. 37.7 mg, 79% yield, 91% ee; R_f = 0.4 (PE/EA = 20/1); colorless oil, $[\alpha]^{20}_D$ = +54.5 (c = 0.044, $CHCl_3$). ^1H NMR (300 MHz, $CDCl_3$) δ 7.36 – 7.33 (m, 2H), 7.16 (d, J = 8.5 Hz, 2H), 7.09 (t, J = 7.8 Hz, 1H), 6.83 (d, J = 7.4 Hz, 1H), 6.75 (d, J = 8.1 Hz, 1H), 4.43 (hept, J = 6.1 Hz, 1H), 2.22 (s, 3H), 1.79 (s, 3H), 1.30 – 1.26 (m, 6H), 1.01 (s, 6H), 0.98 (s, 6H) ppm. ^{13}C NMR (75 MHz, $CDCl_3$) δ 155.1, 149.6, 148.29, 148.28, 144.7, 137.4, 132.8, 129.4, 126.7, 122.7, 120.7 (q, J = 254.8 Hz), 120.5, 112.8, 82.9, 70.9, 24.6, 24.5, 22.41, 22.36, 20.1 ppm. ^{19}F NMR (282 MHz, $CDCl_3$) δ -57.92 ppm. HRMS (ESI) *m/z* calcd for $[C_{26}H_{32}BF_3O_4+H]^+$ 477.2419, found 477.2421. IR (neat, cm^{-1}) 3062, 2976, 1609, 1489, 1379, 1370, 1208, 1148, 1109, 752, 690. HPLC: Daicel Chiralcel OD-H, n-hexane/isopropanol 99.9/0.1, flow rate = 0.5 mL/min, uv-vis λ = 230 nm, t_{R1} = 10.6 min (major), t_{R2} = 11.8 min (minor).

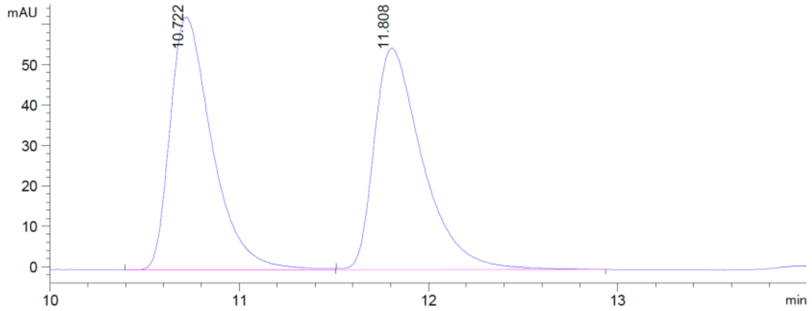

Signal 2: MWD1 B, Sig=230,4 Ref=off

Peak #	RT [min]	Type	Height	Width [min]	Area %	Area
1	10.722	BV	62.535	0.234	49.778	965.397
2	11.808	VB	54.767	0.267	50.222	973.991

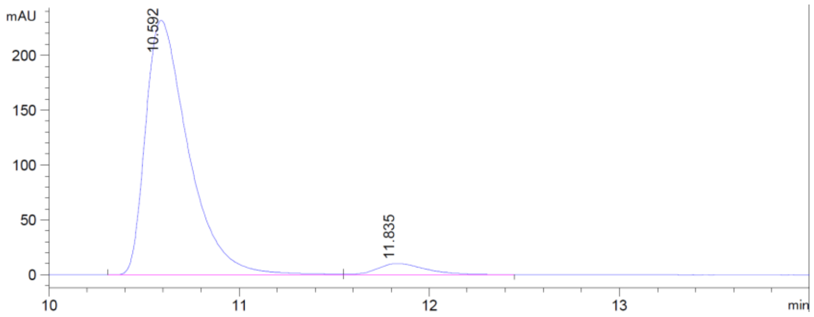

Signal 2: MWD1 B, Sig=230,4 Ref=off

Peak #	RT [min]	Type	Height	Width [min]	Area %	Area
1	10.592	BV	231.848	0.236	95.260	3.625e3
2	11.835	VV	10.238	0.260	4.740	180.371

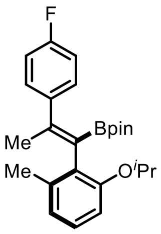

(R,E)-2-(2-(4-fluorophenyl)-1-(2-isopropoxy-6-methylphenyl)prop-1-en-1-yl)-4,4,5,5-tetramethyl-1,3,2-dioxaborolane (5d)

Prepared according to the general procedure. 37.6 mg, 92% yield, 92% ee; R_f = 0.4 (PE/EA = 20/1); colorless oil, $[\alpha]^{20}_D$ = +78.9 (c = 0.038, $CHCl_3$). ^{1}H NMR (300 MHz, $CDCl_3$) δ 7.33 – 7.28 (m, 2H), 7.08 (t, J = 7.8 Hz, 1H), 7.02 – 6.97 (m, 2H), 6.83 (d, J = 7.4 Hz, 1H), 6.75 (d, J = 8.1 Hz, 1H), 4.42 (hept, J = 5.9 Hz, 1H), 2.22 (s, 3H), 1.78 (s, 3H), 1.30 – 1.25 (m, 6H), 1.03 (s, 6H), 1.00 (s, 6H) ppm. ^{13}C NMR (75 MHz, $CDCl_3$) δ 162.2 (d, $^1J_{C\text{-}F}$ = 242.8 Hz), 155.1, 149.7, 141.7 (d, $^4J_{C\text{-}F}$ = 2.8 Hz), 137.5, 133.1, 129.5 (d, $^3J_{C\text{-}F}$ = 7.9 Hz), 126.6, 122.7, 114.5 (d, $^2J_{C\text{-}F}$ = 21.1 Hz), 113.0, 82.8, 71.0, 24.7, 24.5, 22.44, 22.43, 22.3, 20.1 ppm.^{19}F NMR (282 MHz, $CDCl_3$) δ -116.39 ppm. HRMS (ESI) *m/z* calcd for $[C_{25}H_{32}BFO_3+H]^+$ 411.2501, found 411.2496. IR (neat, cm^{-1}) 3065, 2929, 1601, 1508, 1371, 1251, 1145, 1116, 835. HPLC: Daicel Chiralcel OD-H, n-hexane/isopropanol 99.9/0.1, flow rate = 0.65 mL/min, uv-vis λ = 230 nm, t_{R1} = 9.1 min (major), t_{R2} = 10.2 min (minor).

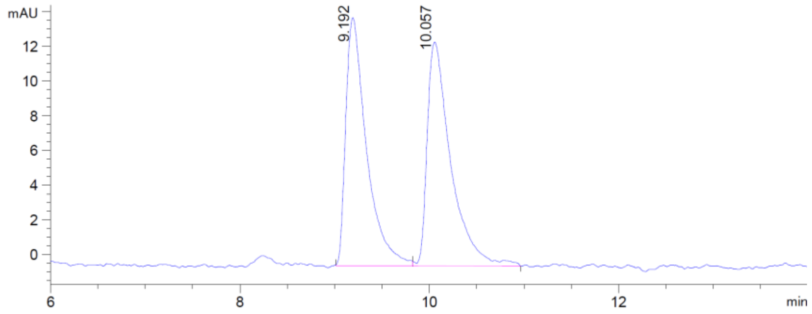

Signal 2: MWD1 B, Sig=230,4 Ref=off

Peak #	RT [min]	Type	Height	Width [min]	Area %	Area
1	9.192	MF	14.292	0.254	49.332	217.628
2	10.057	FM	12.906	0.289	50.668	223.519

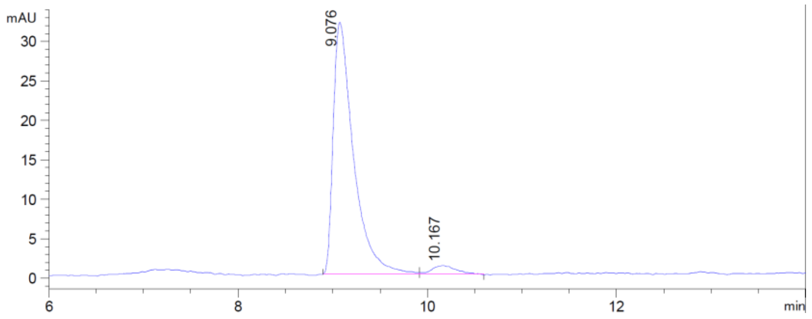

Signal 2: MWD1 B, Sig=230,4 Ref=off

Peak #	RT [min]	Type	Height	Width [min]	Area %	Area
1	9.076	MF	31.922	0.249	96.161	477.021
2	10.167	FM	1.074	0.295	3.839	19.043

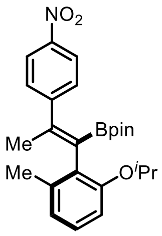

(R,E)-2-(1-(2-isopropoxy-6-methylphenyl)-2-(4-nitrophenyl)prop-1-en-1-yl)-4,4,5,5-tetramethyl-1,3,2-dioxaborolane (5e)

Prepared according to the general procedure. 28.3 mg, 65% yield, 88% ee; R_f = 0.4 (PE/EA = 20/1); yellow solid, m. p. 72 – 74 °C, $[\alpha]^{20}_D$ = +75.8 (c = 0.066, $CHCl_3$). ^1H NMR (300 MHz, $CDCl_3$) δ 8.19 (d, J = 8.7 Hz, 2H), 7.47 (d, J = 8.7 Hz, 2H), 7.11 (t, J = 7.8 Hz, 1H), 6.84 (d, J = 7.5 Hz, 1H), 6.76 (d, J = 8.1 Hz, 1H), 4.44 (hept, J = 6.0 Hz, 1H), 2.23 (s, 3H), 1.81 (s, 3H), 1.30 – 1.27 (m, 6H), 1.02 (s, 6H), 0.98 (s, 6H) ppm. ^{13}C NMR (75 MHz, $CDCl_3$) δ 154.9, 152.8, 149.1, 146.8, 137.2, 132.1, 129.0, 127.0, 123.1, 122.7, 112.5, 83.1, 70.8, 24.6, 24.5, 22.38, 22.37, 22.3, 20.0 ppm. HRMS (ESI) *m/z* calcd for $[C_{25}H_{32}BNO_5+H]^+$ 438.2446, found 438.2452. IR (neat, cm^{-1}) 3061, 2977, 1594, 1518, 1371, 1346, 1252, 1144, 1014, 855. HPLC: Daicel Chiralcel OD-H, n-hexane/isopropanol 99.6/0.4, flow rate = 0.5 mL/min, uv-vis λ = 254 nm, t_{R1} = 10.5 min (minor), t_{R2} = 11.1 min (major).

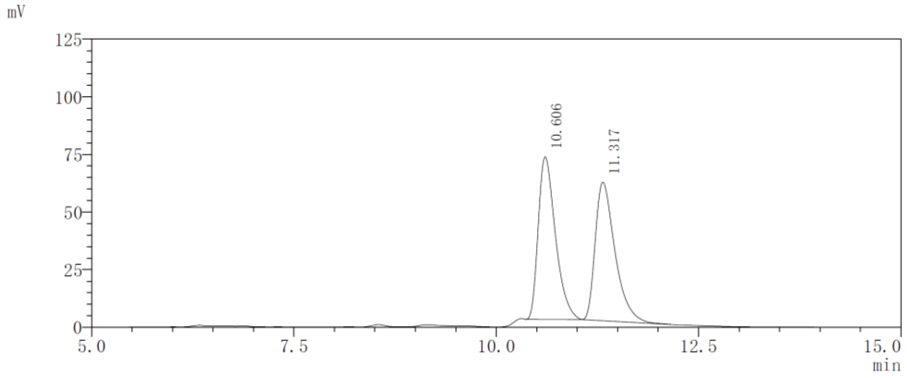

A Ch1 254nm

RetTime[min]	Area	Hight	Area%
10.606	1042517	70549	50.493
11.317	1022153	60141	49.507

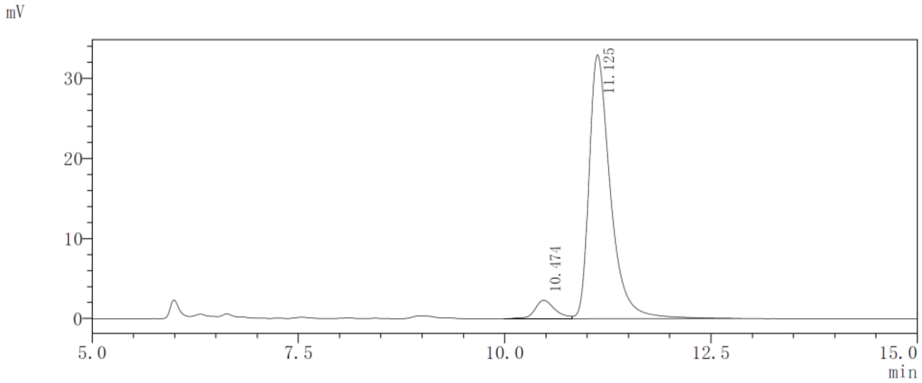

:A Ch1 254nm

RetTime[min]	Area	Hight	Area%
10.474	38596	2299	6.143
11.125	589663	32930	93.857

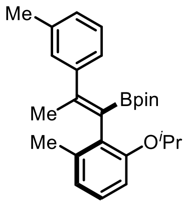

(R,E)-2-(1-(2-isopropoxy-6-methylphenyl)-2-(m-tolyl)prop-1-en-1-yl)-4,4,5,5-tetramethyl-1,3,2-dioxaborolane (5f)

Prepared according to the general procedure. 35.5 mg, 87% yield, 93% ee; R_f = 0.4 (PE/EA = 20/1); colorless oil, $[\alpha]^{20}_{D}$ = +77.6 (c = 0.076, $CHCl_3$). ^{1}H NMR (300 MHz, $CDCl_3$) δ 7.25 – 7.16 (m, 3H), 7.09 – 7.05 (m, 2H), 6.83 (d, *J* = 7.4 Hz, 1H), 6.75 (d, *J* = 8.1 Hz, 1H), 4.43 (hept, *J* = 6.0 Hz, 1H), 2.36 (s, 3H), 2.24 (s, 3H), 1.80 (s, 3H), 1.29 (d, *J* = 6.1 Hz, 3H), 1.26 (d, *J* = 6.1 Hz, 3H), 1.03 (s, 6H), 1.01 (s, 6H) ppm. ^{13}C NMR (75 MHz, $CDCl_3$) δ 155.2, 150.6, 145.5, 137.6, 137.1, 133.4, 128.9, 127.81, 127.78, 126.5, 125.2, 122.8, 113.2, 82.7, 71.1, 24.7, 24.5, 22.4, 22.3, 22.2, 21.7, 20.2 ppm. HRMS (ESI) *m/z* calcd for $[C_{26}H_{35}BO_3+H]^+$ 407.2752, found 407.2762. IR (neat, cm^{-1}) 3057, 2929, 1599, 1460, 1370, 1346, 1252, 1146, 1115, 781, 704. HPLC: Daicel Chiralcel IC-3, n-hexane/isopropanol 99.7/0.3, flow rate = 0.5 mL/min, uv-vis λ = 254 nm, t_{R1} = 7.4 min (minor), t_{R2} = 8.3 min (major).

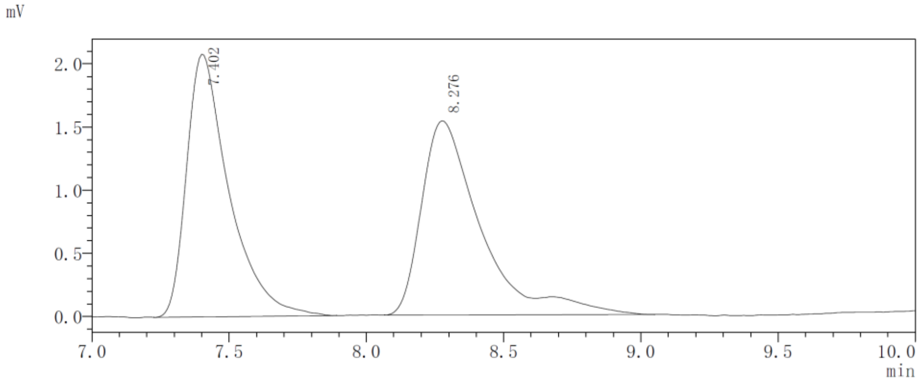

A Ch1 254nm

RetTime[min]	Area	Hight	Area%
7.402	22417	2081	49.480
8.276	22888	1538	50.520

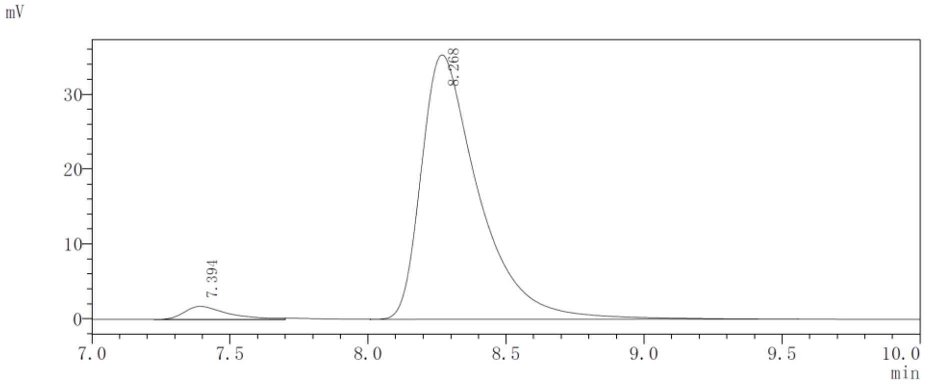

A Ch1 254nm

RetTime[min]	Area	Hight	Area%
7.394	19033	1749	3.585
8.268	511919	35358	96.415

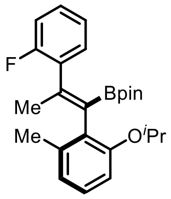

(R,E)-2-(2-(2-fluorophenyl)-1-(2-isopropoxy-6-methylphenyl)prop-1-en-1-yl)-4,4,5,5-tetramethyl-1,3,2-dioxaborolane (5g)

Prepared according to the general procedure. 37.4 mg, 91% yield, 91% ee; R_f = 0.4 (PE/EA = 20/1); colorless oil, $[\alpha]^{20}_D$ = +82.8 (c = 0.058, $CHCl_3$). ^{1}H NMR (300 MHz, $CDCl_3$) δ 7.26 – 7.19 (m, 2H), 7.11 – 6.98 (m, 3H), 6.86 (d, J = 7.5 Hz, 1H), 6.76 (d, J = 8.1 Hz, 1H), 4.40 (hept, J = 6.0 Hz, 1H), 2.25 (s, 3H), 1.78 (s, 3H), 1.29 (d, J = 1.7 Hz, 3H), 1.27 (d, J = 1.7 Hz, 3H), 0.99 (s, 6H), 0.95 (s, 6H) ppm. ^{13}C NMR (75 MHz, $CDCl_3$) δ 159.9 (d, $^1J_{C\text{-}F}$ = 242.9 Hz), 155.1, 145.7, 137.5, 133.4 (d, $^2J_{C\text{-}F}$ = 16.6 Hz), 133.0, 130.7 (d, $^3J_{C\text{-}F}$ = 4.5 Hz), 128.2 (d, $^3J_{C\text{-}F}$ = 8.0 Hz), 126.6, 123.3 (d, $^4J_{C\text{-}F}$ = 3.3 Hz), 123.0, 115.1 (d, $^2J_{C\text{-}F}$ = 22.3 Hz), 113.6, 82.7, 71.4, 24.6, 24.5, 22.4, 22.3 (two overlapping carbon signals), 20.0 ppm. ^{19}F NMR (282 MHz, $CDCl_3$) δ -116.16 ppm. HRMS (ESI) *m/z* calcd for $[C_{25}H_{32}BFO_3+H]^+$ 411.2501, found 411.2502. IR (neat, cm^{-1}) 3061, 2977, 1607, 1579, 1489, 1371, 1349, 1254, 1146, 1112, 756. HPLC: Daicel Chiralcel IC-3, n-hexane/isopropanol 99.5/0.5, flow rate = 0.5 mL/min, uv-vis λ = 230 nm, t_{R1} = 6.9 min (minor), t_{R2} = 7.6 min (major).

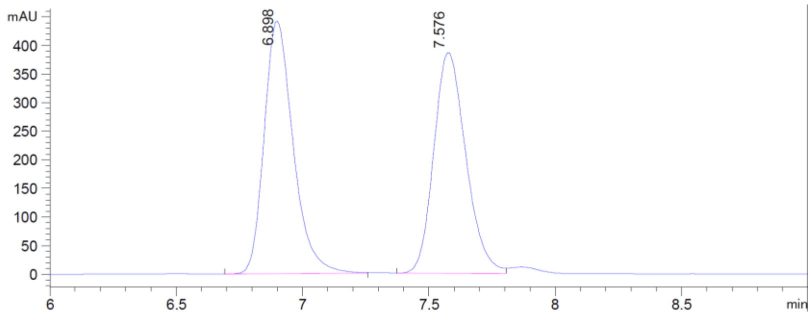

Signal 2: MWD1 B, Sig=230,4 Ref=off

Peak #	RT [min]	Type	Height	Width [min]	Area %	Area
1	6.898	BV	441.730	0.123	50.878	3.530e3
2	7.576	BV	386.365	0.135	49.122	3.408e3

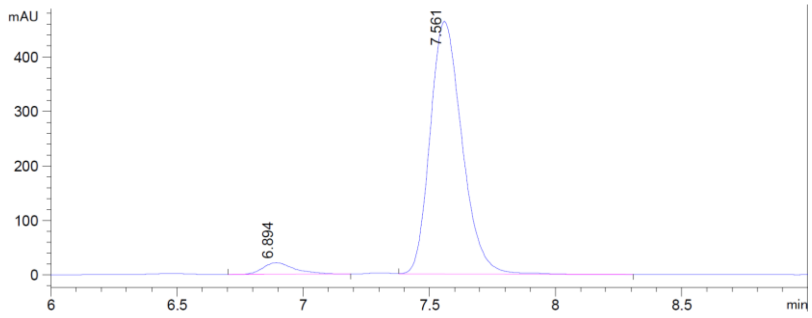

Signal 2: MWD1 B, Sig=230,4 Ref=off

Peak #	RT [min]	Type	Height	Width [min]	Area %	Area
1	6.894	BB	21.229	0.131	4.377	187.341
2	7.561	VB	464.804	0.137	95.623	4.092e3

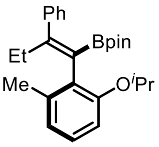

(R,E)-2-(1-(2-isopropoxy-6-methylphenyl)-2-phenylbut-1-en-1-yl)-4,4,5,5-tetramethyl-1,3,2-dioxaborolane (5h)

Prepared according to the general procedure. 37.8 mg, 93% yield, 92% ee; R_f = 0.4 (PE/EA = 20/1); colorless oil, $[\alpha]^{20}_D$ = -2.6 (c = 0.038, $CHCl_3$). ^{1}H NMR (300 MHz, $CDCl_3$) δ 7.33 – 7.23 (m, 5H), 7.08 (t, *J* = 7.8 Hz, 1H), 6.81 (d, *J* = 7.5 Hz, 1H), 6.74 (d, *J* = 8.1 Hz, 1H), 4.47 (hept, *J* = 6.0 Hz, 1H), 2.25 (s, 3H), 2.15 (q, *J* = 7.5 Hz, 2H), 1.31 (d, *J* = 2.7 Hz, 3H), 1.29 (d, *J* = 2.7 Hz, 3H), 0.96 (s, 12H), 0.75 (t, *J* = 7.5 Hz, 3H) ppm. ^{13}C NMR (75 MHz, $CDCl_3$) δ 155.7, 155.1, 144.2, 137.6, 132.5, 128.7, 127.6, 126.7, 126.3, 122.2, 112.1, 82.6, 70.3, 28.3, 24.5 (two overlapping carbon signals), 22.5, 22.3, 20.4, 12.1 ppm. HRMS (ESI) *m/z* calcd for $[C_{26}H_{35}BO_3+H]^+$ 407.2752, found 407.2750. IR (neat, cm^{-1}) 3059, 2932, 1595, 1460, 1371, 1352, 1252, 1146, 1120, 758, 700. HPLC: Daicel Chiralcel OD-H, n-hexane/isopropanol 99.9/0.1, flow rate = 0.2 mL/min, uv-vis λ = 230 nm, t_{R1} = 24.2 min (major), t_{R2} = 26.6 min (minor).

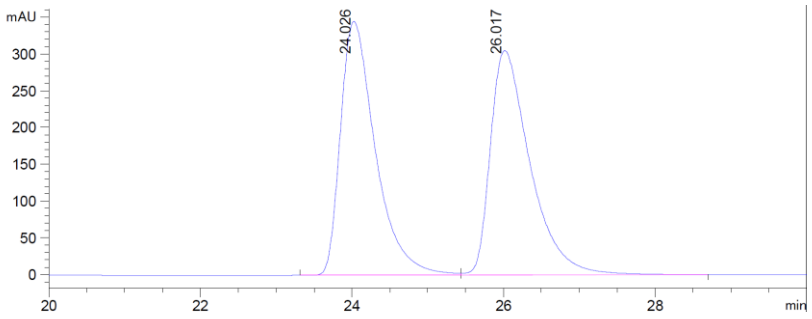

Signal 2: MWD1 B, Sig=230,4 Ref=off

Peak #	RT [min]	Type	Height	Width [min]	Area %	Area
1	24.026	BV	345.185	0.484	49.802	1.107e4
2	26.017	VB	305.364	0.550	50.198	1.115e4

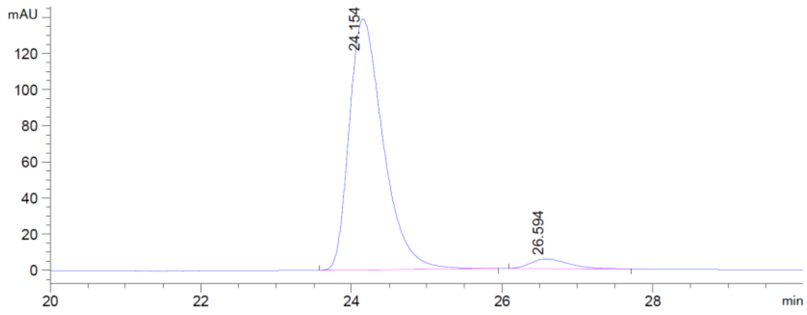

Signal 2: MWD1 B, Sig=230,4 Ref=off

Peak #	RT [min]	Type	Height	Width [min]	Area %	Area
1	24.154	BB	138.969	0.482	95.836	4.363e3
2	26.594	MM	5.378	0.587	4.164	189.572

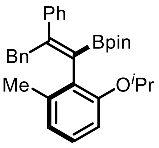

(R,E)-2-(1-(2-isopropoxy-6-methylphenyl)-2,3-diphenylprop-1-en-1-yl)-4,4,5,5-tetramethyl-1,3,2-dioxaborolane (5i)

Prepared according to the general procedure. 39.3 mg, 84% yield, 92% ee; R_f = 0.4 (PE/EA = 20/1); colorless oil, $[\alpha]^{20}_D$ = +11.3 (c = 0.062, $CHCl_3$). ^1H NMR (300 MHz, $CDCl_3$) δ 7.23 – 7.17 (m, 5H), 7.11 – 7.02 (m, 4H), 6.98 – 6.96 (m, 2H), 6.80 (d, *J* = 7.5 Hz, 1H), 6.73 (d, *J* = 8.2 Hz, 1H), 4.55 (hept, *J* = 5.9 Hz, 1H), 3.60 (d, *J* = 14.2 Hz, 1H), 3.41 (d, *J* = 14.2 Hz, 1H), 2.27 (s, 3H), 1.38 (d, *J* = 6.1 Hz, 3H), 1.35 (d, *J* = 6.1 Hz, 3H), 0.96 (s, 6H), 0.94 (s, 6H) ppm. ^{13}C NMR (75 MHz, $CDCl_3$) δ 155.1, 151.7, 144.0, 139.5, 137.9, 131.2, 129.6, 128.9, 127.8, 127.5, 126.73, 126.69, 125.5, 122.0, 110.7, 82.7, 69.6, 41.3, 24.54, 24.50, 22.6, 22.2, 20.4 ppm. HRMS (ESI) *m/z* calcd for $[C_{31}H_{37}BO_3+H]^+$ 469.2909, found 469.2915. IR (neat, cm^{-1}) 3059, 2927, 1596, 1495, 1379, 1371, 1251, 1144, 980, 761, 699. HPLC: Daicel Chiralcel OD-H, n-hexane/isopropanol 99.9/0.1, flow rate = 0.5 mL/min, uv-vis λ = 230 nm, t_{R1} = 13.8 min (major), t_{R2} = 18.8 min (minor).

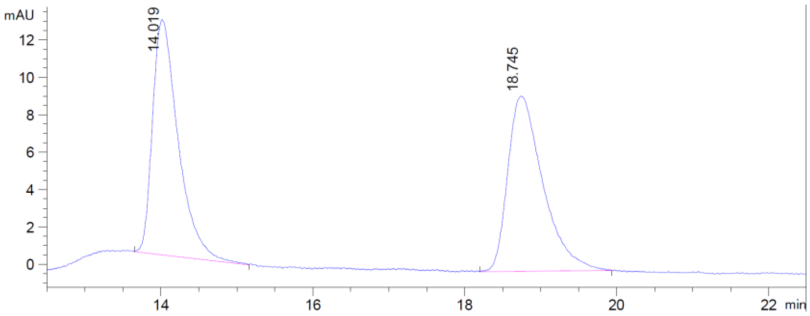

Signal 2: MWD1 B, Sig=230,4 Ref=off

Peak #	RT [min]	Type	Height	Width [min]	Area %	Area
1	14.019	BV	12.566	0.346	49.491	294.880
2	18.745	VB	9.366	0.451	50.509	300.944

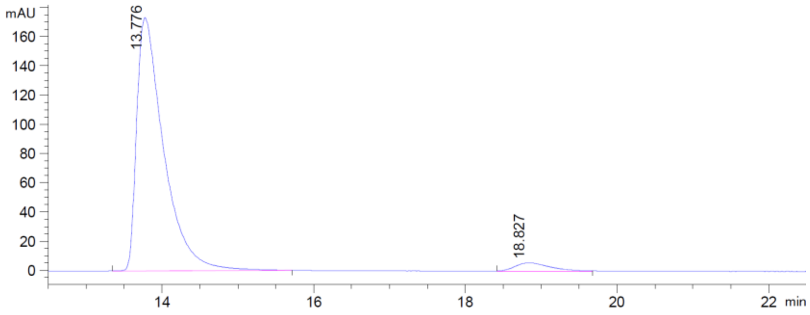

Signal 2: MWD1 B, Sig=230,4 Ref=off

Peak #	RT [min]	Type	Height	Width [min]	Area %	Area
1	13.776	MM	173.475	0.413	95.935	4.304e3
2	18.827	MM	5.756	0.528	4.065	182.352

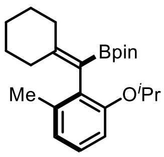

(R)-2-(cyclohexylidene(2-isopropoxy-6-methylphenyl)methyl)-4,4,5,5-tetramethyl-1,3,2-dioxaborolane (5j)

Prepared according to the general procedure. 30.5 mg, 82% yield, 89% ee; R_f = 0.4 (PE/EA = 20/1); white solid, m. p. 72 – 74 °C, $[\alpha]^{20}_D$ = +39.1 (c = 0.046, $CHCl_3$). ^{1}H NMR (300 MHz, $CDCl_3$) δ 7.02 (t, *J* = 7.8 Hz, 1H), 6.79 (d, *J* = 7.5 Hz, 1H), 6.70 (d, *J* = 8.1 Hz, 1H), 4.30 (hept, *J* = 6.0 Hz, 1H), 2.89 – 2.81 (m, 1H), 2.59 – 2.50 (m, 1H), 2.10 (s, 3H), 1.91 – 1.73 (m, 2H), 1.71 – 1.60 (m, 2H), 1.57 – 1.39 (m, 4H), 1.23 (d, *J* = 6.1 Hz, 6H), 1.19 (s, 6H), 1.18 (s, 6H) ppm. ^{13}C NMR (75 MHz, $CDCl_3$) δ 157.8, 155.3, 137.8, 134.4, 125.8, 122.6, 113.5, 82.5, 71.0, 34.1, 33.1, 29.0, 28.0, 27.0, 24.8 (two overlapping carbon signals), 22.4 (two overlapping carbon signals), 20.4 ppm. HRMS (ESI) *m/z* calcd for $[C_{23}H_{35}BO_3+H]^+$ 371.2752, found 371.2755. IR (neat, cm^{-1}) 3057, 2977, 1616, 1577, 1381, 1254, 1146, 1115, 766. HPLC: Daicel Chiralcel OD-H, n-hexane/isopropanol 99.9/0.1, flow rate = 0.5 mL/min, uv-vis λ = 230 nm, t_{R1} = 8.8 min (major), t_{R2} = 9.4 min (minor).

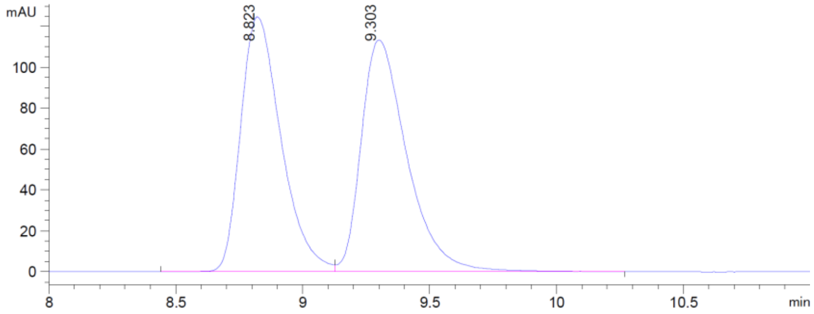

Signal 2: MWD1 B, Sig=230,4 Ref=off

Peak #	RT [min]	Type	Height	Width [min]	Area %	Area
1	8.823	BV	124.741	0.171	49.233	1.380e3
2	9.303	VV	113.515	0.190	50.767	1.423e3

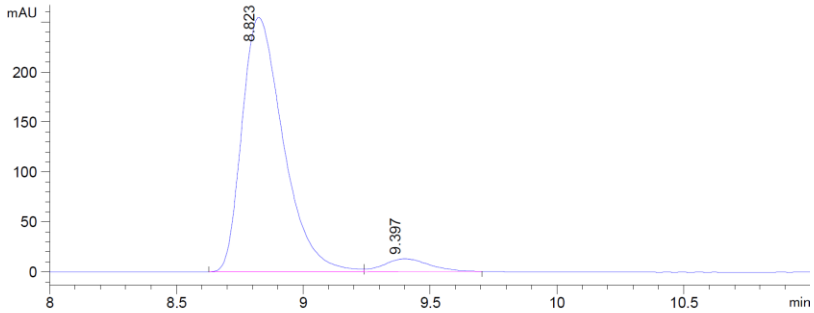

Signal 2: MWD1 B, Sig=230,4 Ref=off

Peak #	RT [min]	Type	Height	Width [min]	Area %	Area
1	8.823	MF	254.527	0.188	94.658	2.876e3
2	9.397	FM	12.948	0.209	5.342	162.319

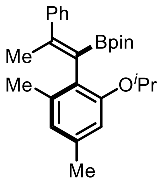

(R,E)-2-(1-(2-isopropoxy-4,6-dimethylphenyl)-2-phenylprop-1-en-1-yl)-4,4,5,5-tetramethyl-1,3,2-dioxaborolane (5k)

Prepared according to the general procedure. 37.9 mg, 93% yield, 92% ee; R_f = 0.4 (PE/EA = 20/1); colorless oil, $[\alpha]^{20}_{D}$ = +100.8 (c = 0.066, $CHCl_3$). ^{1}H NMR (300 MHz, $CDCl_3$) δ 7.36 – 7.22 (m, 5H), 6.67 (s, 1H), 6.58 (s, 1H), 4.40 (hept, *J* = 6.0 Hz, 1H), 2.30 (s, 3H), 2.20 (s, 3H), 1.81 (s, 3H), 1.28 (d, *J* = 6.1 Hz, 3H), 1.25 (d, *J* = 6.1 Hz, 3H), 1.02 (s, 6H), 0.99 (s, 6H) ppm. ^{13}C NMR (75 MHz, $CDCl_3$) δ 155.1, 150.6, 145.8, 137.1, 136.1, 130.4, 128.1, 127.8, 126.9, 123.8, 114.5, 82.7, 71.2, 24.7, 24.5, 22.5, 22.4, 22.3, 21.6, 20.1 ppm. HRMS (ESI) *m/z* calcd for $[C_{26}H_{35}BO_3+H]^+$ 407.2752, found 407.2760. IR (neat, cm^{-1}) 3059, 2977, 1620, 1590, 1506, 1379, 1371, 1267, 1141, 1111, 786, 670. HPLC: Daicel Chiralcel IC-3, n-hexane/isopropanol 99.5/0.5, flow rate = 0.5 mL/min, uv-vis λ = 230 nm, t_{R1} = 6.8 min (minor), t_{R2} = 8.3 min (major).

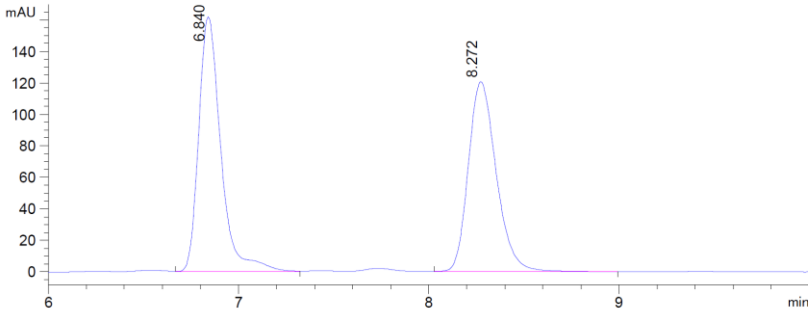

Signal 2: MWD1 B, Sig=230,4 Ref=off

Peak #	RT [min]	Type	Height	Width [min]	Area %	Area
1	6.840	VV	161.842	0.125	51.611	1.313e3
2	8.272	VB	120.648	0.157	48.389	1.231e3

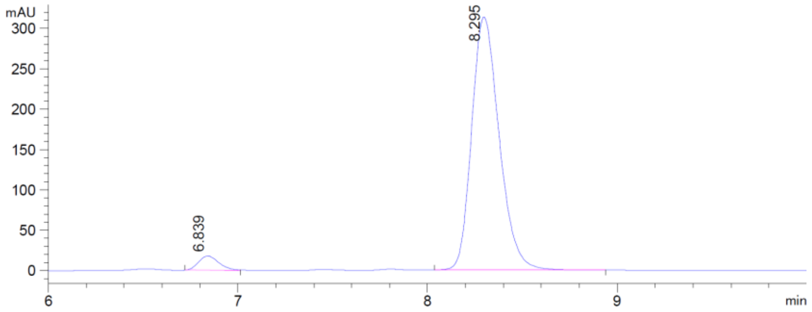

Signal 2: MWD1 B, Sig=230,4 Ref=off

Peak #	RT [min]	Type	Height	Width [min]	Area %	Area
1	6.839	MM	17.387	0.123	3.911	128.779
2	8.295	MM	313.889	0.168	96.089	3.164e3

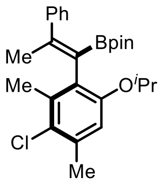

(R,E)-2-(1-(3-chloro-6-isopropoxy-2,4-dimethylphenyl)-2-phenylprop-1-en-1-yl)-4,4,5,5-tetramethyl-1,3,2-dioxaborolane (5l)

Prepared according to the general procedure. 41.2 mg, 94% yield, 94% ee; R_f = 0.4 (PE/EA = 20/1); colorless oil, $[\alpha]^{20}_D$ = +63.8 (c = 0.08, $CHCl_3$). ^{1}H NMR (300 MHz, $CDCl_3$) δ 7.35 – 7.25 (m, 5H), 6.67 (s, 1H), 4.38 (hept, J = 6.1 Hz, 1H), 2.36 (s, 3H), 2.30 (s, 3H), 1.79 (s, 3H), 1.28 (d, J = 6.1 Hz, 3H), 1.25 (d, J = 6.0 Hz, 3H), 1.02 (s, 6H), 0.99 (s, 6H) ppm. ^{13}C NMR (75 MHz, $CDCl_3$) δ 153.2, 151.5, 145.5, 135.2, 134.2, 132.6, 128.1, 127.8, 127.4, 127.1, 116.1, 82.8, 71.6, 24.7, 24.5, 22.5, 22.4, 22.3, 21.3, 18.0 ppm. HRMS (ESI) *m/z* calcd for $[C_{26}H_{34}BClO_3+H]^+$ 441.2362, found 441.2360. IR (neat, cm^{-1}) 3077, 2977, 1596, 1491, 1370, 1348, 1186, 1145, 1115, 761, 699. HPLC: Daicel Chiralcel IC-3, n-hexane/isopropanol 99/1, flow rate = 0.5 mL/min, uv-vis λ = 230 nm, t_{R1} = 6.7 min (minor), t_{R2} = 7.3 min (major).

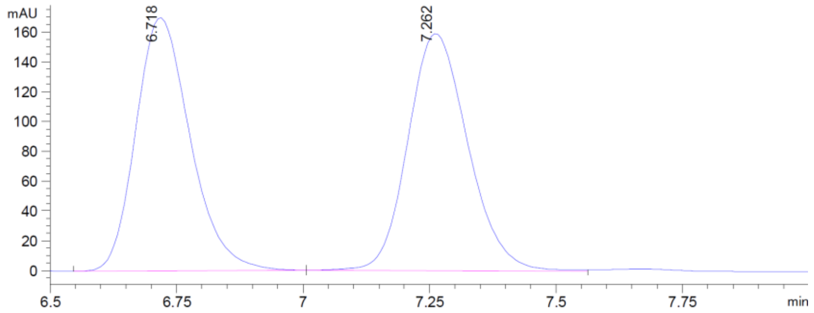

Signal 2: MWD1 B, Sig=230,4 Ref=off

Peak #	RT [min]	Type	Height	Width [min]	Area %	Area
1	6.718	BB	169.725	0.115	49.238	1.269e3
2	7.262	BV	159.037	0.128	50.762	1.308e3

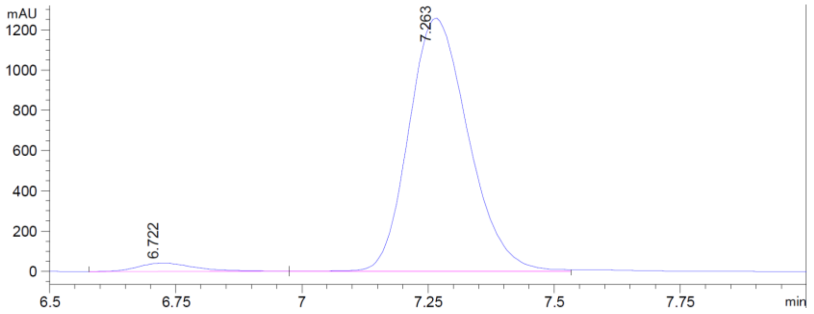

Signal 2: MWD1 B, Sig=230,4 Ref=off

Peak #	RT [min]	Type	Height	Width [min]	Area %	Area
1	6.722	BB	41.226	0.118	2.987	317.222
2	7.263	BV	1.257e3	0.128	97.013	1.030e4

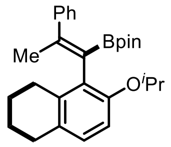

(R,E)-2-(1-(2-isopropoxy-5,6,7,8-tetrahydronaphthalen-1-yl)-2-phenylprop-1-en-1-yl)-4,4,5,5-tetramethyl-1,3,2-dioxaborolane (5m)

Prepared according to the general procedure. 27.3 mg, 63% yield, 85% ee; R_f = 0.4 (PE/EA = 20/1); colorless oil, $[\alpha]^{20}_{D}$ = +8.1 (c = 0.062, $CHCl_3$). ^{1}H NMR (300 MHz, $CDCl_3$) δ 7.37 – 7.24 (m, 5H), 6.91 (d, *J* = 8.3 Hz, 1H), 6.71 (d, *J* = 8.3 Hz, 1H), 4.38 (hept, *J* = 6.0 Hz, 1H), 2.75 – 2.49 (m, 4H), 1.80 (s, 3H), 1.78 – 1.69 (m, 4H), 1.28 (d, *J* = 6.3 Hz, 3H), 1.26 (d, *J* = 6.2 Hz, 3H), 1.01 (s, 12H) ppm. ^{13}C NMR (75 MHz, $CDCl_3$) δ 152.7, 150.7, 145.8, 136.4, 133.0, 130.0, 128.1, 127.8, 127.4, 126.9, 114.0, 82.7, 71.2, 29.7, 27.7, 24.6(two overlapping carbon signals), 23.5, 23.3, 22.5, 22.4(two overlapping carbon signals)ppm. HRMS (ESI) *m/z* calcd for $[C_{28}H_{37}BO_3+H]^+$ 433.2909, found 433.2920. IR (neat, cm^{-1}) 3053, 2976, 1618, 1597, 1371, 1347, 1263, 1146, 1115, 766, 699. HPLC: Daicel Chiralcel IA-3, n-hexane/isopropanol 99/1, flow rate = 0.5 mL/min, uv-vis λ = 230 nm, t_{R1} = 21.1 min (major), t_{R2} = 22.0 min (minor).

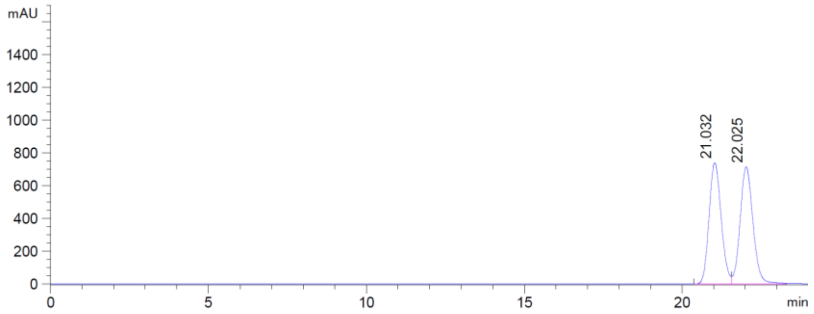

Signal 2: MWD1 B, Sig=230,4 Ref=off

Peak #	RT [min]	Type	Height	Width [min]	Area %	Area
1	21.032	BV	739.730	0.413	49.401	1.971e4
2	22.025	VV	714.112	0.432	50.599	2.019e4

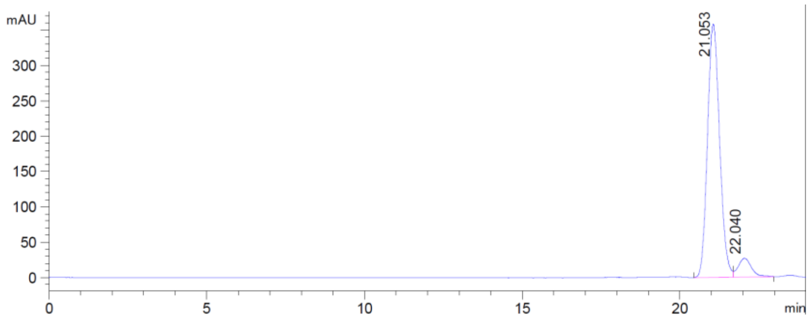

Signal 2: MWD1 B, Sig=230,4 Ref=off

Peak #	RT [min]	Type	Height	Width [min]	Area %	Area
1	21.053	BV	357.768	0.413	92.516	9.551e3
2	22.040	VV	26.444	0.405	7.484	772.559

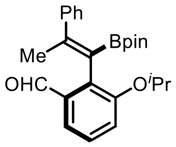

(R,E)-3-isopropoxy-2-(2-phenyl-1-(4,4,5,5-tetramethyl-1,3,2-dioxaborolan-2-yl)prop-1-en-1-yl)benzaldehyde (5n)

Prepared according to the general procedure. 31.8 mg, 78% yield, 60% ee; R_f = 0.4 (PE/EA = 10/1); colorless oil, $[\alpha]^{20}_{D}$ = +101.6 (c = 0.062, $CHCl_3$). ^1H NMR (300 MHz, $CDCl_3$) δ 10.27 (s, 1H), 7.58 (dd, J = 7.7, 1.0 Hz, 1H), 7.36 – 7.28 (m, 6H), 7.15 (dd, J = 8.1, 0.9 Hz, 1H), 4.49 (hept, J = 6.0 Hz, 1H), 1.83 (s, 3H), 1.33 (d, J = 6.1 Hz, 3H), 1.30 (d, J = 6.1 Hz, 3H), 1.03 (s, 6H), 0.97 (s, 6H) ppm. ^{13}C NMR (75 MHz, $CDCl_3$) δ 193.3, 155.7, 153.5, 144.9, 138.2, 135.4, 128.0, 127.9, 127.5, 127.4, 120.8, 119.8, 83.2, 71.9, 24.7, 24.5, 23.1, 22.3, 22.1 ppm. HRMS (ESI) m/z calcd for $[C_{25}H_{31}BO_4+H]^+$ 407.2388, found 407.2386. IR (neat, cm^{-1}) 3051, 2977, 1689, 1590, 1570, 1389, 1373, 1251, 1145, 764, 700. HPLC: Daicel Chiralcel IC-3, n-hexane/isopropanol 98/2, flow rate = 0.5 mL/min, uv-vis λ = 230 nm, t_{R1} = 15.1 min (minor), t_{R2} = 21.5 min (major).

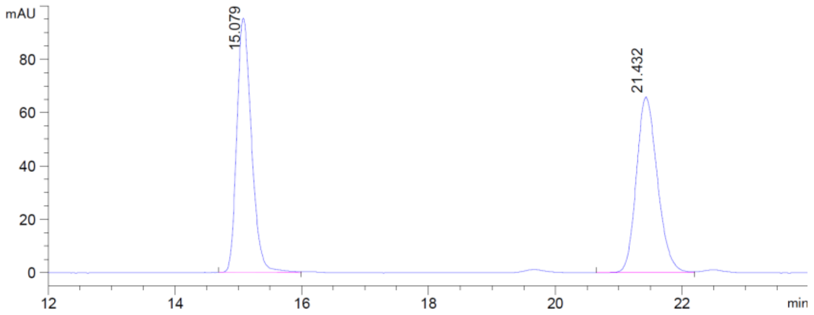

Signal 2: MWD1 B, Sig=230,4 Ref=off

Peak #	RT [min]	Type	Height	Width [min]	Area %	Area
1	15.079	BV	95.304	0.248	50.184	1.535e3
2	21.432	BV	65.814	0.356	49.816	1.523e3

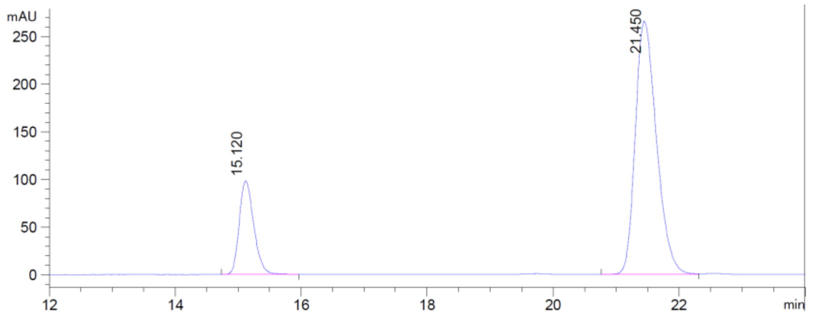

Signal 2: MWD1 B, Sig=230,4 Ref=off

Peak #	RT [min]	Type	Height	Width [min]	Area %	Area
1	15.120	BB	98.529	0.249	20.232	1.580e3
2	21.450	VV	265.709	0.362	79.768	6.231e3

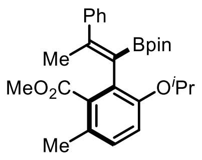

methyl (R,E)-3-isopropoxy-6-methyl-2-(2-phenyl-1-(4,4,5,5-tetramethyl-1,3,2-dioxaborolan-2-yl)prop-1-en-1-yl)benzoate (5o)
Prepared according to the general procedure. 39.1 mg, 87% yield, 19% ee; R_f = 0.4 (PE/EA = 10/1); colorless oil, $[\alpha]^{20}_{D}$ = 19.0 (c = 0.058, $CHCl_3$). ^1H NMR (300 MHz, $CDCl_3$) δ 7.32 – 7.23 (m, 5H), 7.03 (d, *J* = 8.3 Hz, 1H), 6.87 (d, *J* = 8.3 Hz, 1H), 4.42 (hept, *J* = 6.1 Hz, 1H), 3.80 (s, 3H), 2.29 (s, 3H), 1.82 (s, 3H), 1.30 (d, *J* = 6.1 Hz, 3H), 1.25 (d, *J* = 6.0 Hz, 3H), 1.04 (s, 6H), 1.00 (s, 6H) ppm. ^{13}C NMR (75 MHz, $CDCl_3$) δ 170.1, 153.2, 152.1, 145.6, 134.5, 132.5, 129.0, 128.0, 127.8, 127.7, 127.0, 117.4, 82.7, 71.7, 51.8, 24.61, 24.56, 23.1, 22.3, 19.4 ppm. HRMS (ESI) *m/z* calcd for $[C_{27}H_{35}BO_5+H]^+$ 451.2650, found 451.2656. IR (neat, cm^{-1}) 3075, 2930, 1731, 1597, 1470, 1371, 1348, 1277, 1146, 1010, 760, 700. HPLC: Daicel Chiralcel IC-3, n-hexane/isopropanol 99.5/0.5, flow rate = 0.5 mL/min, uv-vis λ = 230 nm, t_{R1} = 13.9 min (major), t_{R2} = 18,7 min (minor).

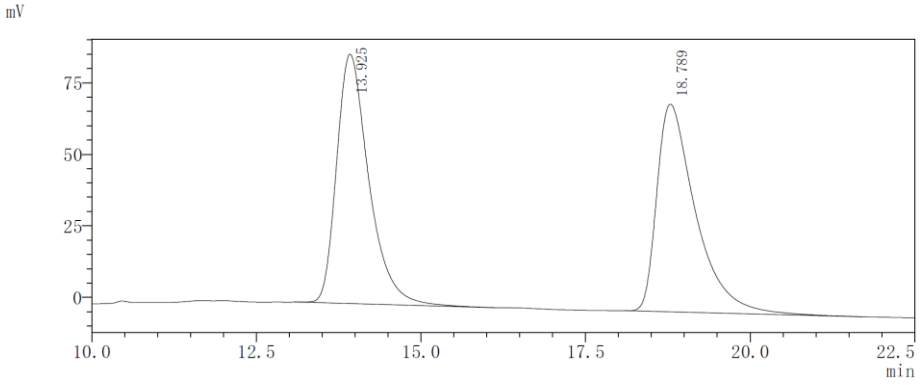

A Ch2 230nm

RetTime[min]	Area	Hight	Area%
13.925	2975531	87209	49.991
18.789	2976639	72579	50.009

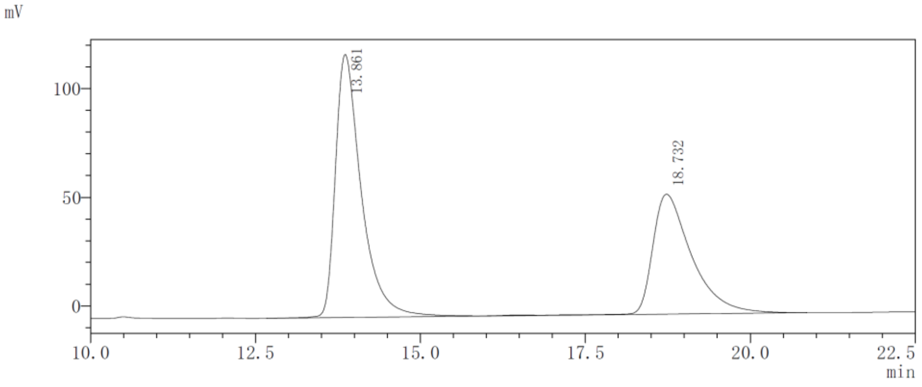

A Ch2 230nm

RetTime[min]	Area	Hight	Area%
13.861	3330096	121002	59.617
18.732	2255703	55162	40.383

7. Scale-up Reaction.

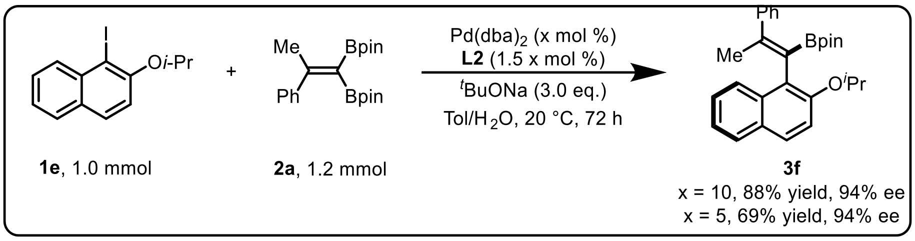

An oven-dried 100 mL two-neck bottle was charged with Pd(dba)$_2$ (58.0 mg, 0.1 mmol, 10 mol %), **L2** (52.0 mg, 0.15 mmol, 15 mol %) and toluene (3.0 mL) under argon atmosphere. The reaction mixture was stirred at r. t. for 30 minutes. Then **1e** (1.0 mmol, 314 mg, 1.0 eq.), **2a** (1.2 mmol, 444 mg 1.2 eq.), ^{t}BuONa (3.0 mmol, 288 mg, 3.0 eq. in 2.0 mL water) and toluene (7.0 mL) was added under argon atmosphere. The reaction mixture was stirred at 20 °C (water temperature) for 72 h. The reaction mixture was diluted with EA (30.0 mL) and filtered through a plug of Celite. The filtrate was washed with water and brine, dried over anhydrous Na_2SO_4, and concentrated under vacuum to give yellow residue, which was purified by flash chromatography on silica gel with PE/EA to afford product **3f** (377.0 mg, 88% yield, 94% ee). While the reaction was run with 5 mol% Pd(dba)$_2$ and 7.5 mol% **L2**, the product **3f** was isolated in 69% yield (296.4mg) with 94% ee.

8. Derivatization and Characterization of Products

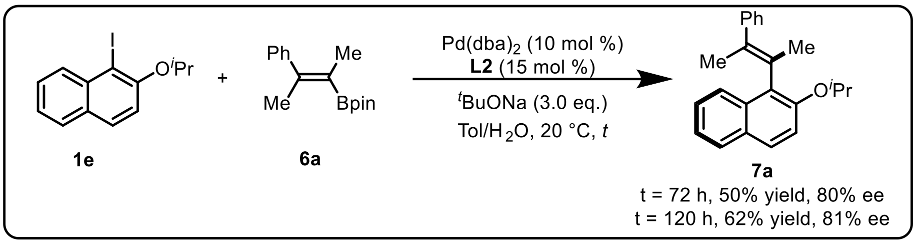

(R,E)-2-isopropoxy-1-(3-phenylbut-2-en-2-yl)naphthalene(7a)

Prepared according to the general procedure for 120 h. 19.5 mg, 62% yield, 81% ee; R_f = 0.4 (PE/EA = 50/1); colorless oil, $[\alpha]^{20}_D$ = -26.7 (c = 0.03, $CHCl_3$). ^1H NMR (300 MHz, $CDCl_3$) δ 7.88 (d, J = 8.4 Hz, 1H), 7.81 (d, J = 8.0 Hz, 1H), 7.75 (d, J = 8.9 Hz, 1H), 7.49 – 7.33 (m, 6H), 7.32 – 7.26 (m, 2H), 4.66 (hept, J = 5.9 Hz, 1H), 1.903 – 1.899 (m, 3H), 1.661 – 1.657 (m, 3H), 1.38 (d, J = 2.5 Hz, 3H), 1.36 (d, J = 2.5 Hz, 3H) ppm. ^{13}C NMR (75 MHz, $CDCl_3$) δ 151.7, 144.5, 134.8, 132.4, 129.5, 129.1, 128.5, 128.3, 128.2, 128.1, 127.9, 126.41, 126.38, 124.8, 123.7, 117.7, 71.5, 22.9, 22.8, 22.5, 21.0 ppm. HRMS (ESI) *m/z* calcd for $[C_{23}H_{24}O+H]^+$ 317.1900, found 317.1893. IR (neat, cm^{-1}) 3057, 2974, 1621, 1591, 1505, 1370, 1329, 1245, 1092, 748, 702. HPLC: Daicel Chiralcel IA-3, n-hexane/isopropanol 99.9/0.1, flow rate = 0.5 mL/min, uv-vis λ = 230 nm, t_{R1} = 9.9 min (minor), t_{R2} = 10.3 min (major).

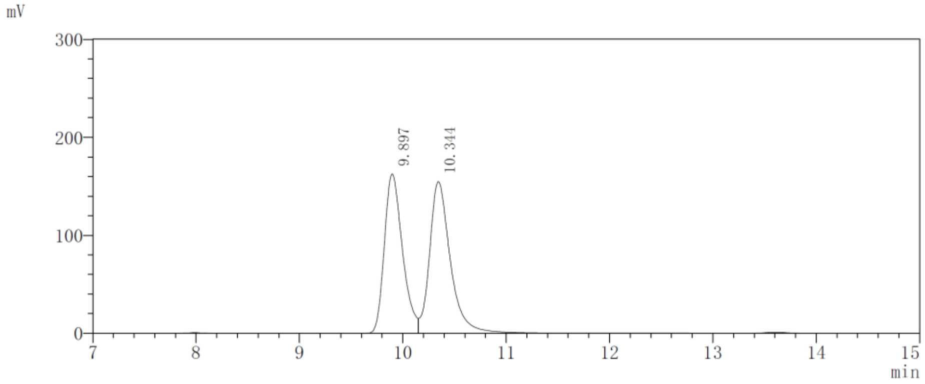

:A Ch2 230nm

RetTime[min]	Area	Hight	Area%
9.897	1987122	162933	47.705
10.344	2178301	155176	52.295

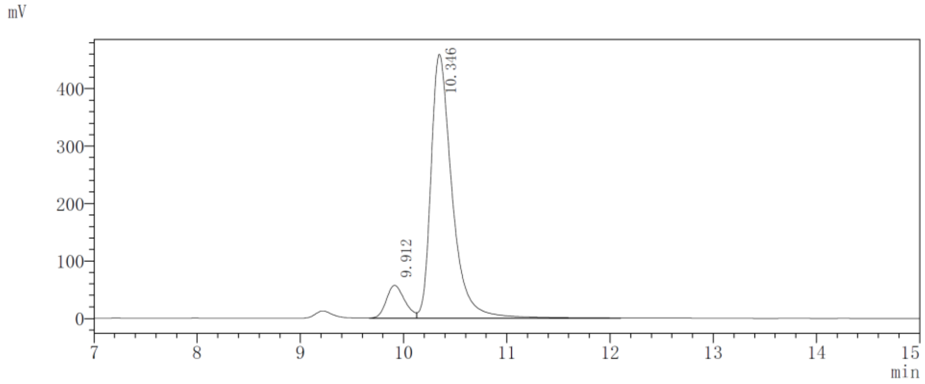

A Ch2 230nm

RetTime[min]	Area	Hight	Area%
9.912	700916	56837	9.710
10.346	6517300	459104	90.290

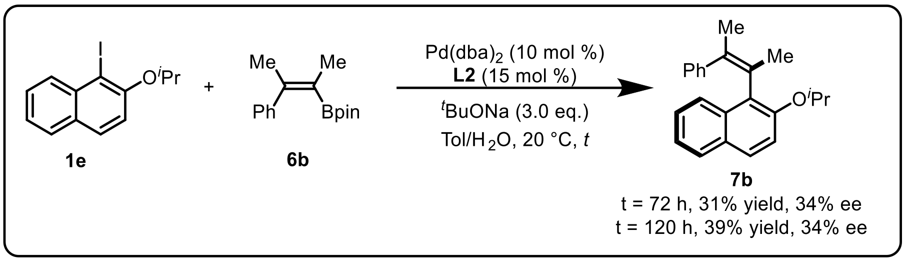

(R,Z)-2-isopropoxy-1-(3-phenylbut-2-en-2-yl)naphthalene(7b)

Prepared according to the general procedure for 120 h. 12.2mg, 39% yield, 34% ee; R_f = 0.3 (PE/EA = 50/1); colorless oil, $[\alpha]^{20}_D$ = -13.6 (c = 0.022, $CHCl_3$). ^1H NMR (300 MHz, $CDCl_3$) δ 7.74 (d, J = 8.5 Hz, 1H), 7.62 (d, J = 7.9 Hz, 1H), 7.56 (d, J = 9.0 Hz, 1H), 7.35 – 7.30 (m, 1H), 7.22 – 7.17 (m, 1H), 7.07 (d, J = 9.0 Hz, 1H), 7.00 – 6.98 (m, 2H), 6.90 – 6.79 (m, 3H), 4.62 (hept, J = 6.1 Hz, 1H), 2.27 (s, 3H), 2.09 (s, 3H), 1.32 (d, J = 6.0 Hz, 3H), 1.29 (d, J = 6.0 Hz, 3H) ppm. ^{13}C NMR (75 MHz, $CDCl_3$) δ 151.5, 144.9, 134.5, 132.6, 128.7, 127.9, 127.6, 127.54, 127.50, 127.1, 125.8, 125.6, 125.2, 123.0, 115.0, 69.6, 22.6, 22.5, 20.8, 20.7 ppm. HRMS (ESI) *m/z* calcd for $[C_{23}H_{24}O+H]^+$ 317.1900, found 317.1897. IR (neat, cm^{-1}) 3059, 2975, 1591, 1507, 1380, 1263, 1115, 747, 700. HPLC: Daicel Chiralcel IA-3, n-hexane/isopropanol 99.9/0.1, flow rate = 0.5 mL/min, uv-vis λ = 230 nm, t_{R1} = 9.0 min (minor), t_{R2} = 9.5 min (major).

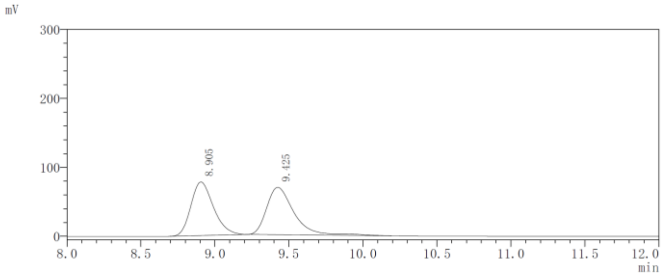

A Ch2 230nm

RetTime[min]	Area	Hight	Area%
8.905	826479	77717	48.372
9.425	882094	68744	51.628

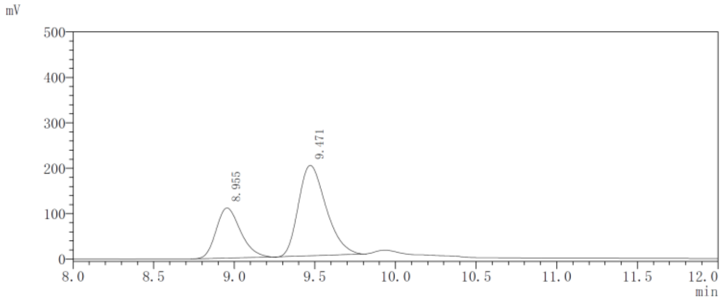

A Ch2 230nm

RetTime[min]	Area	Hight	Area%
8.955	1168706	110373	32.964
9.471	2376701	199051	67.036

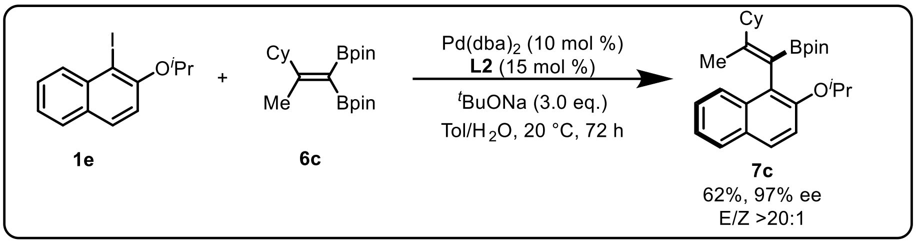

(R,E)-2-(2-cyclohexyl-1-(2-isopropoxynaphthalen-1-yl)prop-1-en-1-yl)-4,4,5,5-tetramethyl-1,3,2-dioxaborolane (7c) Prepared according to the general procedure. 26.8mg, 62% yield, 97% ee; R_f = 0.4 (PE/EA = 20/1); colorless oil, $[\alpha]^{20}_D$ = 12.5 (c = 0.044, $CHCl_3$). ^{1}H NMR (300 MHz, $CDCl_3$) δ 7.77 – 7.73 (m, 2H), 7.65 (d, *J* = 8.8 Hz, 1H), 7.38 – 7.27 (m, 2H), 7.19 (d, *J* = 8.8 Hz, 1H), 4.41 (hept, *J* = 6.0 Hz, 1H), 3.28 – 3.21 (m, 1H), 1.82 – 1.71 (m, 5H), 1.54 – 1.36 (m, 4H), 1.33 (s, 3H), 1.28 (d, *J* = 6.1 Hz, 3H), 1.23 (d, *J* = 6.1 Hz, 3H), 1.19 (s, 6H), 1.16 (s, 6H) ppm. ^{13}C NMR (75 MHz, $CDCl_3$) δ 159.3, 152.0, 133.5, 130.4, 130.1, 128.0, 126.9, 125.9, 125.5, 123.5, 120.2, 82.6, 72.9, 45.5, 32.3, 31.9, 26.9, 26.8, 26.6, 25.0, 24.6, 22.7, 22.5, 16.6 ppm. HRMS (ESI) *m/z* calcd for $[C_{28}H_{39}BO_3+H]^+$ 435.3065, found 435.3061. IR (neat, cm^{-1}) 3060, 2974, 1591, 1505, 1379, 1370, 1291, 1147, 1043, 749. HPLC: Lux Cellulose-1, n-hexane/isopropanol 99.9/0.1, flow rate = 0.5 mL/min, uv-vis λ = 230 nm, t_{R1} = 9.1 min (major), t_{R2} = 11.0 min (minor).

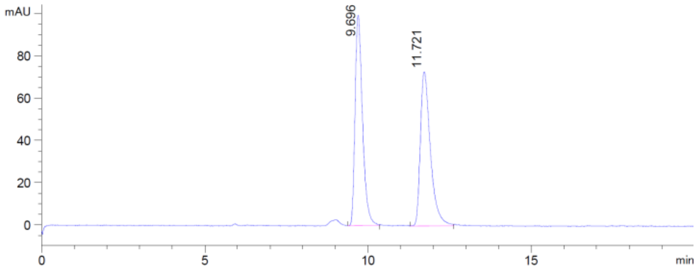

Signal 2: MWD1 B, Sig=230,4 Ref=off

Peak #	RT [min]	Type	Height	Width [min]	Area %	Area
1	9.696	BV	99.462	0.241	50.195	1.577e3
2	11.721	BV	72.973	0.320	49.805	1.564e3

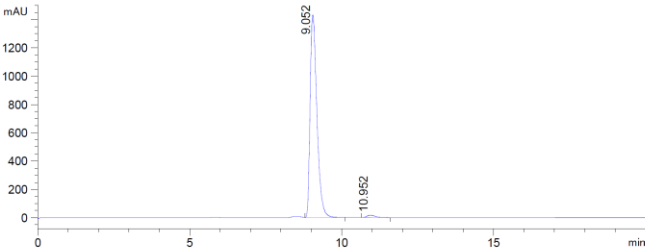

Signal 2: MWD1 B, Sig=230,4 Ref=off

Peak #	RT [min]	Type	Height	Width [min]	Area %	Area
1	9.052	VV	1.431e3	0.240	98.573	2.229e4
2	10.952	VB	17.022	0.285	1.427	322.632

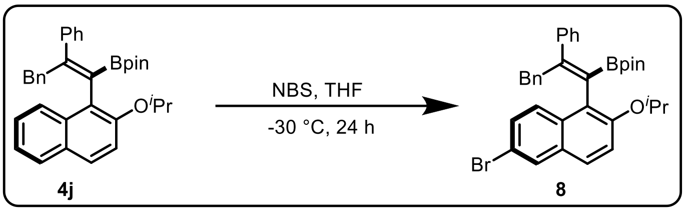

(R,E)-2-(1-(6-bromo-2-isopropoxynaphthalen-1-yl)-2,3-diphenylprop-1-en-1-yl)-4,4,5,5-tetramethyl-1,3,2-dioxaborolane (8) A solution of **4j** (50.4 mg, 0.1 mmol, 1.0 eq.) in 1.0 mL THF was stirred at -30 °C. NBS (178.0 mg, 1.0 mmol, 10.0 eq.) in 1.0 mL THF was added to the solution. Then the reaction mixture was stirred at same temperature for 24 h. The resulting mixture of product was allowed to reach ambient temperature. Then the crude product was purified by flash chromatography on silica gel (PE/EA as the eluent) to obtain target product (38.7 mg) in 68% yield with 97% ee as white solid; [11] R_f = 0.4 (PE/EA = 20/1), m. p. = 188 – 190 °C, $[\alpha]^{20}_{D}$ = –52.6 (c = 0.038, $CHCl_3$). ^{1}H NMR (300 MHz, $CDCl_3$) δ 7.92 (d, J = 2.0 Hz, 1H), 7.89 (d, J = 9.1 Hz, 1H), 7.62 (d, J = 9.0 Hz, 1H), 7.47 (dd, J = 9.0, 2.0 Hz, 1H), 7.29 – 7.19 (m, 6H), 7.04 – 6.97 (m, 3H), 6.88 – 6.85 (m, 2H), 4.70 (hept, J = 5.9 Hz, 1H), 3.45 (s, 2H), 1.43 – 1.39 (m, 6H), 0.93 (s, 6H), 0.88 (s, 6H) ppm. ^{13}C NMR (75 MHz, $CDCl_3$) δ 154.0, 152.4, 143.8, 139.2, 132.3, 130.3, 129.9, 129.5, 129.1, 128.9, 127.8, 127.6, 127.5, 127.0, 126.9, 126.4, 125.6, 117.7, 117.2, 83.0, 71.1, 41.8, 24.51, 24.49, 22.8, 22.5 ppm. HRMS (ESI) m/z calcd for $[C_{34}H_{36}BBrO_3+H]^+$ 583.2014, found 583.2011. IR (neat, cm^{-1}) 3057, 2929, 1584, 1493, 1360, 1345, 1266, 1144, 1116, 730, 701. HPLC: Daicel Chiralcel IC-3, n-hexane/isopropanol 99.7/0.3, flow rate = 0.5 mL/min, uv-vis λ = 230 nm, t_{R1} = 9.6 min (minor), t_{R2} = 10.5 min (major).

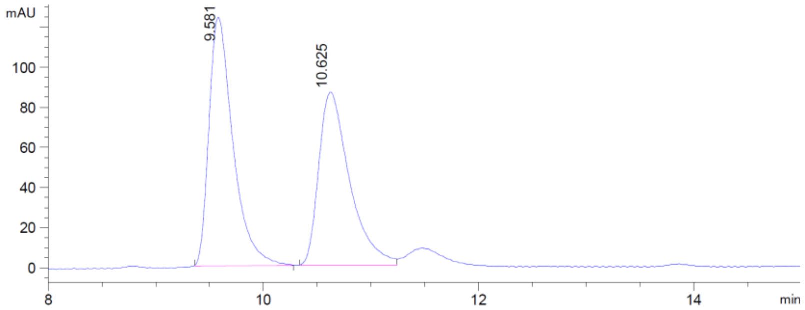

Signal 2: MWD1 B, Sig=230,4 Ref=off

Peak #	RT [min]	Type	Height	Width [min]	Area %	Area
1	9.581	MM	123.933	0.260	52.829	1.931e3
2	10.625	BV	86.273	0.297	47.171	1.724e3

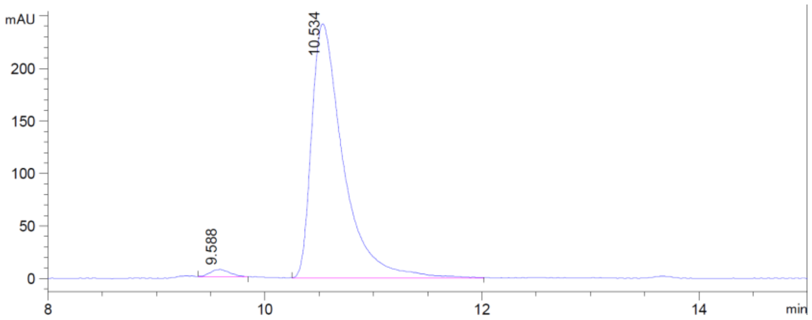

Signal 2: MWD1 B, Sig=230,4 Ref=off

Peak #	RT [min]	Type	Height	Width [min]	Area %	Area
1	9.588	MM	6.703	0.205	1.652	82.559
2	10.534	MM	241.937	0.339	98.348	4.916e3

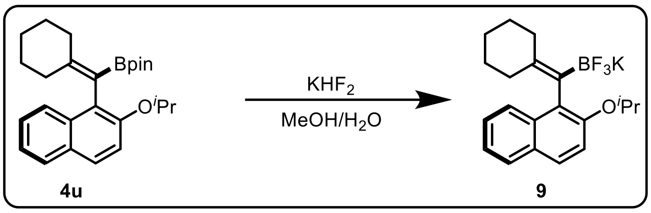

(R)-(cyclohexylidene(2-isopropoxynaphthalen-1-yl)methyl)trifluoro-l4-borane, potassium salt (9) To a 25-mL flask charged with **4u** (122 mg, 0.3 mmol) and MeOH (2.0 mL) was added KHF_2 (118 mg, 1.5 mmol) in H_2O (0.3 mL) at room temperature. The resulting mixture was allowed to stir until TLC shows that the raw material is completely consumed. The reaction was then concentrated. The residue was washed with acetone three times (3 × 5.0 mL). The combined acetone solution was then concentrated. Et_2O (6.0 mL) was then added and the resulting mixture was sonicated for 5 min. The white precipitate was then isolated by filtration to afford target product as white solid (97.9 mg, 85% yield). [12] M.p. = 154 – 156 °C. ^{1}H NMR (300 MHz, DMSO-d_6) δ 8.06 – 8.00 (m, 1H), 7.69 – 7.64 (m, 1H), 7.46 (d, J = 8.7 Hz, 1H), 7.25 – 7.20 (m, 2H), 7.04 (d, J = 8.7 Hz, 1H), 4.50 (hept, J = 6.1 Hz, 1H), 2.59 – 2.41 (m, 2H), 1.62 – 1.24 (m, 8H), 1.20 (d, J = 6.1 Hz, 3H), 1.11 (d, J = 6.0 Hz, 3H) ppm. ^{13}C NMR (75 MHz, DMSO-d_6) δ 150.5, 140.0 (two overlapping carbon signals), 136.6, 134.5, 129.7, 127.8, 126.7, 123.8, 123.6, 122.8, 122.0, 71.8, 33.4, 32.8, 28.6, 27.6, 27.0, 22.9, 22.3 ppm. ^{19}F NMR (282 MHz, DMSO-d_6) δ -131.20 ppm. HRMS (ESI) *m/z* calcd for $[C_{20}H_{23}BF_3KO\text{-}K]^-$ 347.1800, found 347.1799. IR (neat, cm^{-1}) 3053, 2975, 1588, 1504, 1371, 1219, 1135, 1049, 753.

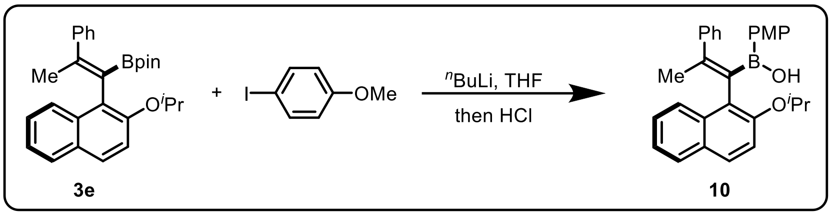

(R,E)-hydroxy(1-(2-isopropoxynaphthalen-1-yl)-2-phenylprop-1-en-1-yl)(4-methoxyphenyl)borane (10) Under the argon atmosphere, to a mixture of 1-iodo-4-methoxybenzenee (234 mg, 1.0 mmol) in dry THF (1 mL) was added dropwise n-butyllithium (0.4 mL, 2.5 M, 1.0 mmol) at -78 °C. The solution was stirred for 1 hour, and then **3e** (85.6 mg, 0.2 mmol) in 1.0 mL of dry THF was added to the above mixture. The solution was stirred for 1 hour, and then warming to room temperature and stirred for overnight. Then, 5 mL of 1M hydrochloric acid was added to reaction mixture and stirred for other 3 hours. The reaction mixture was extracted with ethyl acetate (5 mL × 3). The combined organic layers were dried over anhydrous sodium sulfate, filtered and concentrated under the reduced pressure. The crude residue was purified by flash column chromatography on silica gel using hexanes/ethyl acetate as eluent (30/1) to afford target product as white solid (51.3 mg, 58% yield).[13] R_f = 0.2 (PE/EA = 20/1), m. p. = 177 – 179 °C. ^{1}H NMR (300 MHz, $CDCl_3$) δ 8.26 (s, 1H), 8.06 (d, *J* = 8.5 Hz, 1H), 7.78 (d, *J* = 7.9 Hz, 1H), 7.73 (d, *J* = 8.9 Hz, 1H), 7.63 – 7.58 (m, 2H), 7.52 – 7.42 (m, 3H), 7.38 – 7.30 (m, 2H), 7.24 – 7.18 (m, 2H), 7.15 – 7.09 (m, 1H), 6.65 – 6.60 (m, 2H), 4.71 (hept, *J* = 6.1 Hz, 1H), 3.69 (s, 3H), 1.88 (s, 3H), 1.48 (d, *J* = 6.1 Hz, 3H), 1.32 (d, *J* = 6.1 Hz, 3H) ppm. ^{13}C NMR (75 MHz, $CDCl_3$) δ 161.4, 150.8, 146.0, 144.9, 137.1, 133.0, 130.3, 129.4, 128.4, 128.2, 128.04, 128.02, 127.7, 126.6, 125.6, 124.3, 118.4, 112.7, 73.7, 55.0, 22.9, 22.5, 21.3. HRMS (ESI) *m/z* calcd for $[C_{29}H_{29}BO_3+Na]^+$ 459.2102, found 459.2097. IR (neat, cm^{-1}) 3528, 3106, 2957, 2925, 1598, 1507, 1465, 1373, 136, 1248, 1177, 758, 700, 670.

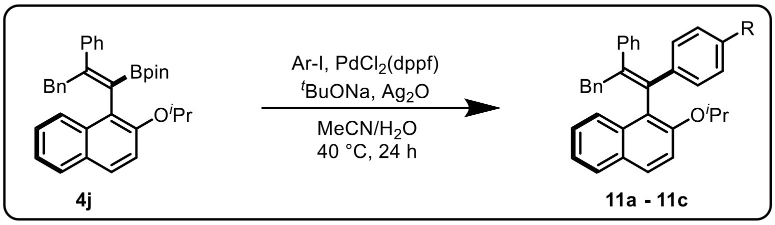

An oven-dried 10 mL Schlenk tube was charged with **4j** (50.4 mg, 0.1 mmol), $PdCl_2$(dppf) (7.3 mg, 0.01 mmol), Ag_2O (27.8 mg, 0.12 mmol), substituted phenyl iodide (0.12 mmol) and ^{t}BuONa (28.8 mg, 0.3 mmol) under argon atmosphere. Then MeCN (1.0 mL), and H_2O (0.1 mL) was added under argon atmosphere. The mixture was allowed to stir at 40 °C for 24 h. After cooled down to the room temperature, water (2.0 mL) was added to dilute the reaction and the mixture was extracted with EA three times (3 × 5.0 mL). The combined organic phase was then dried over anhydrous Na_2SO_4. After removal of the solvent, the residue was purified by column chromatography on silica gel using PE/EA (50:1) as the eluent to afford target product **11a** – **11c**.[14]

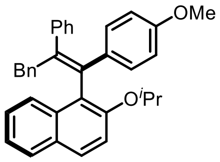

(R,E)-2-isopropoxy-1-(1-(4-methoxyphenyl)-2,3-diphenylprop-1-en-1-yl)naphthalene (11a)

27.3 mg, 56% yield, 92% ee; R_f = 0.3 (PE/EA = 20/1), colorless oil, $[\alpha]^{20}_{D}$ = +104.0 (c = 0.05, $CHCl_3$). ^1H NMR (300 MHz, $CDCl_3$) δ 8.13 (d, J = 8.5 Hz, 1H), 7.80 – 7.76 (m, 2H), 7.46 (ddd, J = 8.3, 6.8, 1.2 Hz, 1H), 7.33 (ddd, J = 8.0, 6.9, 1.0 Hz, 1H), 7.24 (d, J = 4.9 Hz, 1H), 7.14 – 7.02 (m, 10H), 6.97 – 6.92 (m, 2H), 6.50 – 6.46 (m, 2H), 4.68 (hept, J = 6.0 Hz, 1H), 3.63 – 3.59 (m, 4H), 3.42 (d, J = 14.7 Hz, 1H), 1.37 (d, J = 6.0 Hz, 3H), 1.02 (d, J = 6.0 Hz, 3H) ppm. ^{13}C NMR (75 MHz, $CDCl_3$) δ 157.6, 152.3, 143.0, 140.1, 139.8, 135.0, 134.6, 133.6, 131.5, 130.1, 129.6, 128.9, 128.7, 128.2, 127.9, 127.8, 126.8, 126.6, 126.2, 125.6, 125.0, 123.5, 115.1, 112.6, 69.8, 55.1, 42.8, 22.7, 21.9 ppm. HRMS (ESI) *m/z* calcd for $[C_{35}H_{32}O_2+H]^+$ 485.2475, found 485.2474. IR (neat, cm^{-1}) 3061, 2928, 1604, 1508, 1371, 1246, 1034, 746, 700. HPLC: Daicel Chiralcel IC-3, n-hexane/isopropanol 97/3, flow rate = 0.5 mL/min, uv-vis λ = 230 nm, t_{R1} = 7.6 min (minor), t_{R2} = 8.0 min (major).

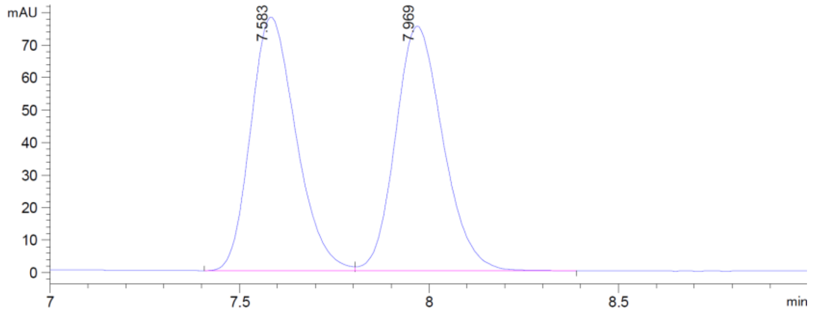

Signal 1: MWD1 A, Sig=254,4 Ref=off

Peak #	RT [min]	Type	Height	Width [min]	Area %	Area
1	7.583	BV	78.161	0.129	49.671	650.268
2	7.969	VV	75.461	0.134	50.329	658.880

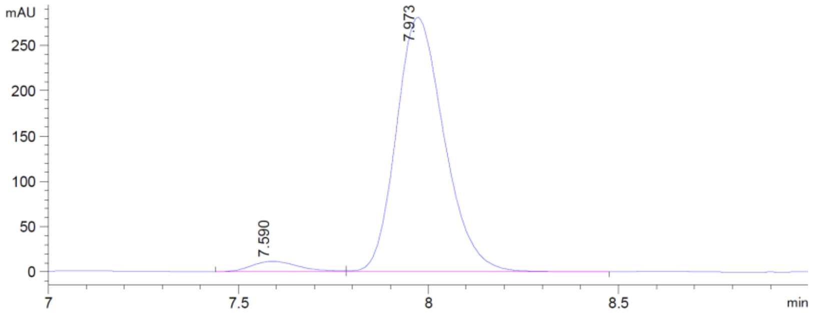

Signal 1: MWD1 A, Sig=254,4 Ref=off

Peak #	RT [min]	Type	Height	Width [min]	Area %	Area
1	7.590	BV	11.786	0.133	3.965	101.779
2	7.973	VB	280.944	0.136	96.035	2.465e3

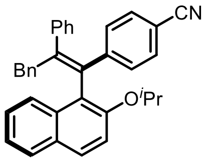

(R,E)-4-(1-(2-isopropoxynaphthalen-1-yl)-2,3-diphenylprop-1-en-1-yl)benzonitrile (11b)

26.5 mg, 55% yield, 95% ee; R_f = 0.3 (PE/EA = 20/1), colorless oil, $[\alpha]^{20}_D$ = +218.2 (c = 0.022, $CHCl_3$). ^{1}H NMR (300 MHz, $CDCl_3$) δ 8.08 (d, *J* = 8.5 Hz, 1H), 7.85 – 7.81 (m, 2H), 7.51 (ddd, *J* = 8.4, 6.8, 1.4 Hz, 1H), 7.38 (ddd, *J* = 8.1, 6.8, 1.2 Hz, 1H), 7.25 – 7.20 (m, 3H), 7.15 – 7.08 (m, 8H), 7.06 – 7.00 (m, 4H), 4.68 (hept, *J* = 6.0 Hz, 1H), 3.66 (d, *J* = 14.5 Hz, 1H), 3.42 (d, *J* = 14.5 Hz, 1H), 1.37 (d, *J* = 6.0 Hz, 3H), 0.96 (d, *J* = 6.0 Hz, 3H) ppm. ^{13}C NMR (75 MHz, $CDCl_3$) δ 152.6, 147.9, 143.7, 141.8, 139.2, 133.8, 133.4, 131.1 (two overlapping carbon signals), 129.9, 129.6, 129.5, 128.9, 128.5, 128.1 (two overlapping carbon signals), 127.3, 127.0, 126.0, 124.8, 124.3, 123.7, 119.4, 114.7, 109.1, 69.8, 42.8, 22.6, 21.8 ppm. HRMS (ESI) *m/z* calcd for $[C_{35}H_{29}NO+H]^+$ 480.2322, found 480.2319. IR (neat, cm^{-1}) 3061, 2974, 2224, 1601, 1505, 1373, 1266, 1114, 1000, 753, 701. HPLC: Daicel Chiralcel IC-3, n-hexane/isopropanol 95/5, flow rate = 0.5 mL/min, uv-vis λ = 230 nm, t_{R1} = 19.6 min (major), t_{R2} = 21.6 min (minor).

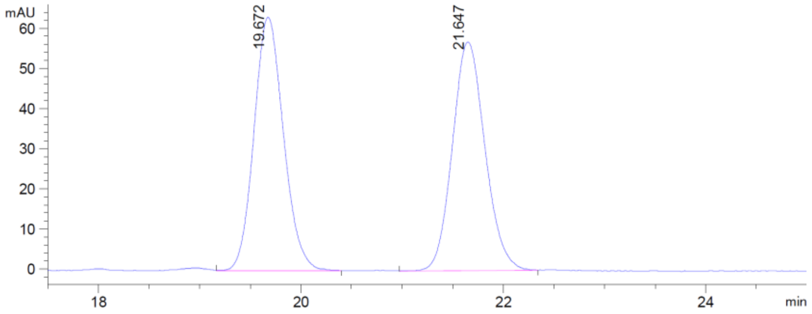

Signal 2: MWD1 B, Sig=230,4 Ref=off

Peak #	RT [min]	Type	Height	Width [min]	Area %	Area
1	19.672	VV	63.190	0.311	50.148	1.283e3
2	21.647	BV	57.010	0.349	49.852	1.275e3

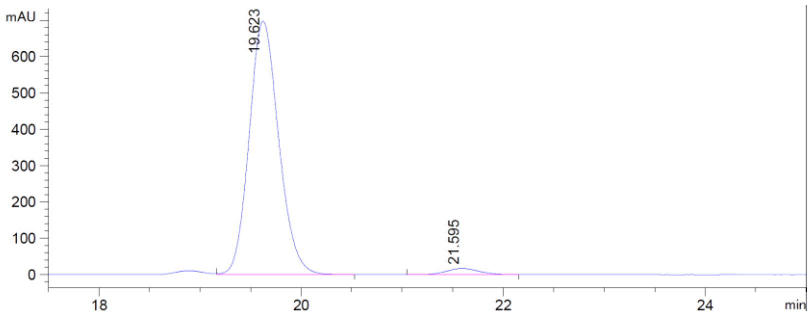

Signal 2: MWD1 B, Sig=230,4 Ref=off

Peak #	RT [min]	Type	Height	Width [min]	Area %	Area
1	19.623	VB	696.880	0.314	97.382	1.419e4
2	21.595	VV	16.959	0.336	2.618	381.457

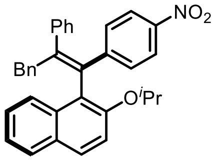

(R,E)-2-isopropoxy-1-(1-(4-nitrophenyl)-2,3-diphenylprop-1-en-1-yl)naphthalene (11c)

32.6 mg, 65% yield, 96% ee; R_f = 0.3 (PE/EA = 20/1), yellow oil, $[\alpha]^{20}_{D}$ = +209.7 (c = 0.036, $CHCl_3$). ^1H NMR (300 MHz, $CDCl_3$) δ 8.08 (d, J = 8.5 Hz, 1H), 7.85 – 7.84 (m, 1H), 7.82 – 7.78 (m, 3H), 7.54 – 7.49 (m, 1H), 7.40 – 7.35 (m, 1H), 7.24 (d, J = 8.1 Hz, 1H), 7.18 – 7.08 (m, 8H), 7.05 – 7.01 (m, 4H), 4.69 (hept, J = 6.0 Hz, 1H), 3.67 (d, J = 14.5 Hz, 1H), 3.44 (d, J = 14.5 Hz, 1H), 1.38 (d, J = 6.0 Hz, 3H), 0.99 (d, J = 6.0 Hz, 3H) ppm. ^{13}C NMR (75 MHz, $CDCl_3$) δ 152.6, 150.1, 145.6, 144.3, 141.7, 139.1, 133.5, 133.4, 131.1, 129.9, 129.7, 129.5, 128.9, 128.5, 128.14, 128.07, 127.4, 127.1, 126.0, 124.5, 124.2, 123.8, 122.5, 114.7, 69.8, 42.9, 22.6, 21.9 ppm. HRMS (ESI) m/z calcd for $[C_{34}H_{29}NO_3+H]^+$ 500.2220, found 500.2226. IR (neat, cm^{-1}) 3059, 2975, 1621, 1591, 1342, 1266, 1113, 854, 748, 700. HPLC: Daicel Chiralcel IC-3, n-hexane/isopropanol 97/3, flow rate = 0.5 mL/min, uv-vis λ = 230 nm, t_{R1} = 17.6 min (major), t_{R2} = 19.8 min (minor).

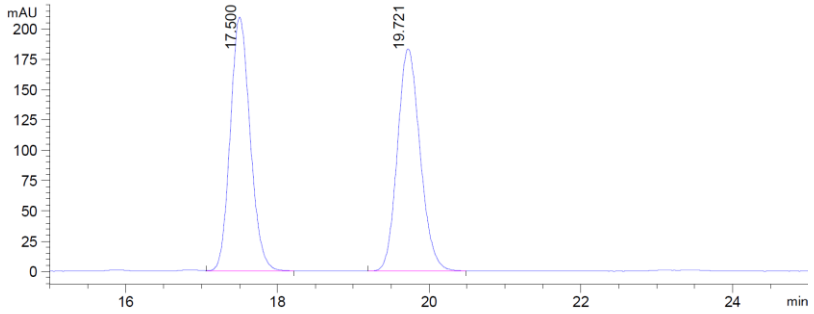

Signal 2: MWD1 B, Sig=230,4 Ref=off

Peak #	RT [min]	Type	Height	Width [min]	Area %	Area
1	17.500	VV	209.402	0.281	49.964	3.818e3
2	19.721	BV	183.446	0.321	50.036	3.823e3

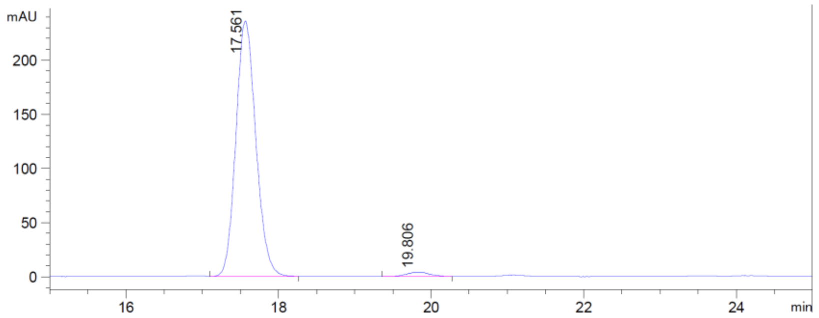

Signal 2: MWD1 B, Sig=230,4 Ref=off

Peak #	RT [min]	Type	Height	Width [min]	Area %	Area
1	17.561	VV	235.816	0.281	98.045	4.305e3
2	19.806	VV	4.029	0.263	1.955	85.858

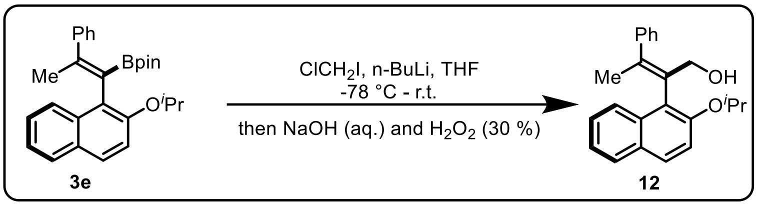

(S,Z)-2-(2-isopropoxynaphthalen-1-yl)-3-phenylbut-2-en-1-ol (12) To a 25-mL oven-dried Schlenk tube charged with **3e** (85.6 mg, 0.2 mmol), $ClCH_2I$ (73 µL, 1.0 mmol) and THF (2 mL) was added n-BuLi (0.4 mL, 2.5 M in THF, 1.0 mmol) at -78 ˚C under argon atmosphere. The resulting mixture was allowed to stir at the same temperature for 1 h and then allowed to stir at room temperature for 24 h. The mixture was then diluted by H_2O (1.0 mL). An aqueous solution (1.0 mL) of NaOH (3.0 M) and 30 % H_2O_2 (1.0 mL) was introduced to the above reaction mixture at 0 °C. The resulting biphasic mixture was then continued to stir at room temperature for 2 h. The mixture was then diluted by H_2O (10.0 mL) and quenched by saturated aqueous $Na_2S_2O_3$ (1.0 mL). The biphasic mixture was then extracted by EA three times (3 × 10.0 mL). The combined organic phase was dried over anhydrous Na_2SO_4. After removal of the solvent, the residue was purified by column chromatography on silica gel using PE/EA (50:1) as the eluent to afford target product as white solid (56.3 mg, 85% yield, 94% ee)[15]. R_f = 0.3 (PE/EA = 10/1), m. p. = 90 – 92 °C, $[\alpha]^{20}_D$ = –131.5 (c = 0.054, $CHCl_3$). ^{1}H NMR (300 MHz, $CDCl_3$) δ 7.85 – 7.78 (m, 3H), 7.51 – 7.40 (m, 5H), 7.38 – 7.30 (m, 3H), 4.71 (hept, J = 6.0 Hz, 1H), 4.40 (d, J = 11.1 Hz, 1H), 4.10 (d, J = 11.1 Hz, 1H), 2.81 (s, 1H), 1.70 (s, 3H), 1.45 (d, J = 6.0 Hz, 3H), 1.33 (d, J = 6.0 Hz, 3H) ppm. ^{13}C NMR (75 MHz, $CDCl_3$) δ 152.1, 142.8, 140.3, 133.0, 131.0, 129.8, 128.9, 128.40, 128.37, 128.1, 127.2, 126.8, 125.9, 124.7, 124.1, 116.9, 72.2, 64.9, 22.9, 22.7, 22.5 ppm. HRMS (ESI) m/z calcd for $[C_{23}H_{24}O_2+H]^+$ 333.1849, found 333.1838. IR (neat, cm^{-1}) 3504, 3055, 2978, 1590, 1507, 1375, 1234, 1015, 747, 708. HPLC: Daicel Chiralcel IC-3, n-hexane/isopropanol 80/20, flow rate = 0.5 mL/min, uv-vis λ = 230 nm, t_{R1} = 11.5 min (major), t_{R2} = 16.5 min (minor).

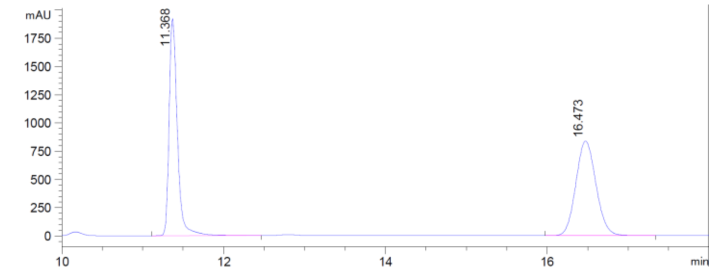

Signal 2: MWD1 B, Sig=230,4 Ref=off

Peak #	RT [min]	Type	Height	Width [min]	Area %	Area
1	11.368	VV	1.919e3	0.109	48.579	1.364e4
2	16.473	BB	837.046	0.267	51.421	1.444e4

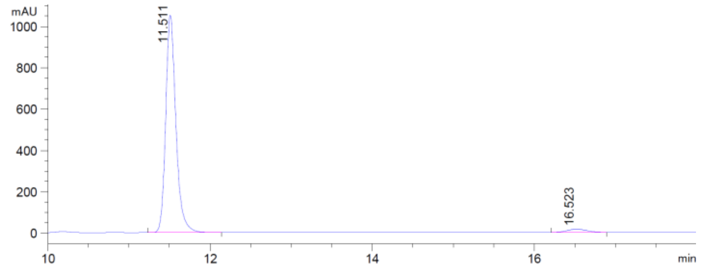

Signal 2: MWD1 B, Sig=230,4 Ref=off

Peak #	RT [min]	Type	Height	Width [min]	Area %	Area
1	11.511	BV	1.052e3	0.129	97.232	9.133e3
2	16.523	MM	15.394	0.281	2.768	259.984

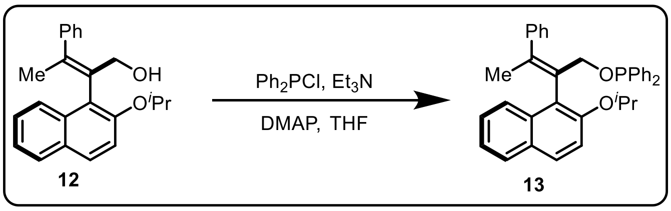

(S,Z)-((2-(2-isopropoxynaphthalen-1-yl)-3-phenylbut-2-en-1-yl)oxy)diphenyl phosphane (13) To a solution of **12** (33.2 mg, 0.1 mmol), Et_3N (35.4 mg, 0.35 mmol) and DMAP (2.4 mg, 0.02 mmol) in THF (2.0 mL) was added Ph_2PCl (66.2 mg, 0.3 mmol) at room temperature, then the mixture was stirred at that temperature for 24 h, after which the solvent was removed in vacuo, the residue was purified by column chromatography on silica gel using PE/EA (2:1) as the eluent to afford target product as white solid 13 (42.9mg, 83% yield, 94% ee) [16]. R_f = 0.3 (PE/EA = 2/1), m. p. = 114 – 116 °C, $[\alpha]^{20}_D$ = –40.0 (c = 0.04, $CHCl_3$). ^{1}H NMR (300 MHz, $CDCl_3$) δ 8.08 (d, J = 8.5 Hz, 1H), 7.87 (d, J = 7.4 Hz, 1H), 7.85 (d, J = 8.9 Hz, 1H), 7.54 – 7.48 (m, 1H), 7.44 – 7.38 (m, 1H), 7.37 – 7.28 (m, 8H), 7.21 (d, J = 9.1 Hz, 1H), 7.18 – 7.12 (m, 2H), 7.10 – 6.95 (m, 4H), 4.86 (dd, J = 10.6, 2.3 Hz, 1H), 4.71 – 4.65 (m, 1H), 4.48 (hept, J = 6.1 Hz, 1H), 1.739 – 1.737 (d, J = 0.7 Hz, 3H), 1.26 (d, J = 6.0 Hz, 3H), 1.05 (d, J = 6.0 Hz, 3H) ppm. ^{13}C NMR (75 MHz, $CDCl_3$) δ 152.5, 142.3, 141.7, 134.1, 132.8, 131.9, 131.8, 131.70, 131.67, 131.6, 131.52, 131.45, 131.42, 131.38, 131.1, 130.9, 130.1, 129.0, 128.8, 128.6, 128.21, 128.15, 128.13, 128.09, 128.04, 127.98, 127.7, 127.6, 127.3, 126.5, 125.6, 123.6, 123.5, 115.8, 70.6, 65.6 (d, J = 5.3 Hz), 22.8, 22.6, 22.4 ppm. ^{31}P NMR (121 MHz, $CDCl_3$) δ 30.25 ppm. HRMS (ESI) *m/z* calcd for $[C_{35}H_{33}O_2P+H]^+$ 517.2291, found 517.2292. IR (neat, cm^{-1}) 3057, 2977, 1584, 1493, 1360, 1345, 1266, 1144, 1116, 730, 701. HPLC: Daicel Chiralcel AD-H, n-hexane/isopropanol 85/15, flow rate = 0.5 mL/min, uv-vis λ = 230 nm, t_{R1} = 13.6 min (major), t_{R2} = 17.6 min (minor).

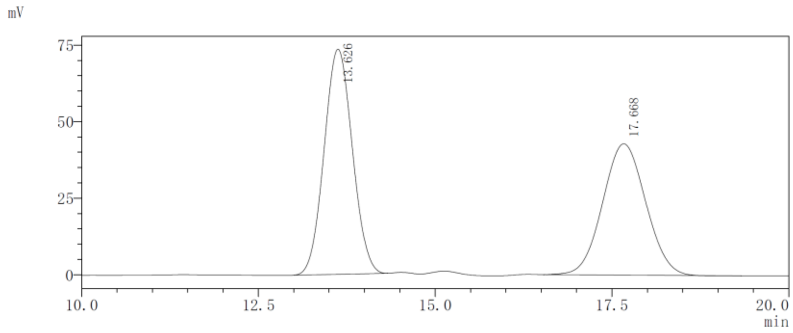

A Ch2 230nm

RetTime[min]	Area	Hight	Area%
13.626	2044270	73541	52.960
17.668	1815790	42903	47.040

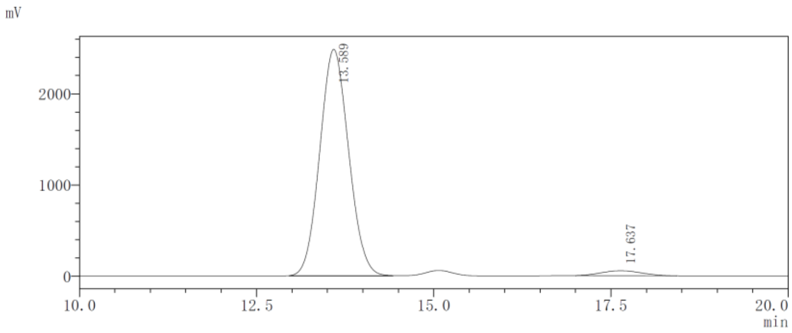

A Ch2 230nm

RetTime[min]	Area	Hight	Area%
13.589	70215431	2483278	97.030
17.637	2149040	54592	2.970

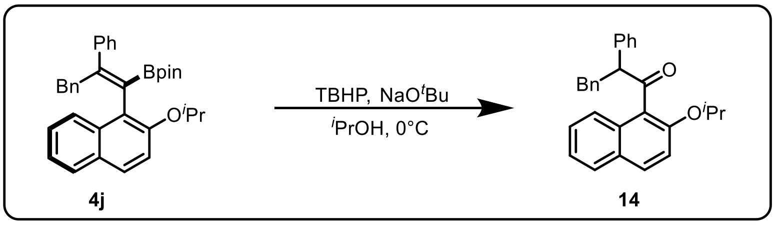

1-(2-isopropoxynaphthalen-1-yl)-2,3-diphenylpropan-1-one (14) To a solution of **4j** (50.4 mg, 0.1 mmol) in ^{i}PrOH (1.0 mL) at 0 °C was added NaO^{t}Bu (9.6 mg, 0.1 mmol). Then 5M tert-butyl hydroperoxide(0.1mL) was dropwise to the mixture. The mixture was stirred at the same temperature for 3 h, after which the solvent was removed in vacuo, the residue was purified by column chromatography on silica gel using PE/EA (50:1) as the eluent to afford target product as colorless 14 (32.7mg, 83% yield, 92% ee). R_f = 0.3 (PE/EA = 20/1), $[\alpha]^{20}_D$ = –130.0 (c = 0.04, $CHCl_3$). ^{1}H NMR (300 MHz, $CDCl_3$) δ 7.75 (d, J = 9.1 Hz, 1H), 7.65 – 7.62 (m, 1H), 7.23 – 7.04 (m, 14H), 4.78 (dd, J = 9.2, 5.6 Hz, 1H), 4.68 (hept, J = 6.1 Hz, 1H), 3.68 (dd, J = 13.8, 5.6 Hz, 1H), 3.31 (dd, J = 13.8, 9.3 Hz, 1H), 1.35 (d, J = 6.0 Hz, 3H), 1.18 (d, J = 6.1 Hz, 3H) ppm. ^{13}C NMR (75 MHz, $CDCl_3$) δ 206.0, 152.4, 140.0, 137.0, 131.5, 131.4, 129.4, 129.3, 128.7, 128.4, 128.2, 127.7, 127.2, 127.1, 126.3, 126.1, 124.0, 115.1, 71.7, 62.0, 38.1, 22.5, 22.1 ppm. HRMS (ESI) m/z calcd for $[C_{28}H_{26}O_2+H]^+$ 395.2006, found 395.2001. IR (neat, cm^{-1}) 3061, 2972, 1694, 1593, 1509, 1358, 1244, 1098, 747, 698. HPLC: Daicel

Chiralcel IC-3, n-hexane/isopropanol 90/10, flow rate = 0.5 mL/min, uv-vis λ = 230 nm, t_{R1} = 13.6 min (major), t_{R2} = 17.6 min (minor).

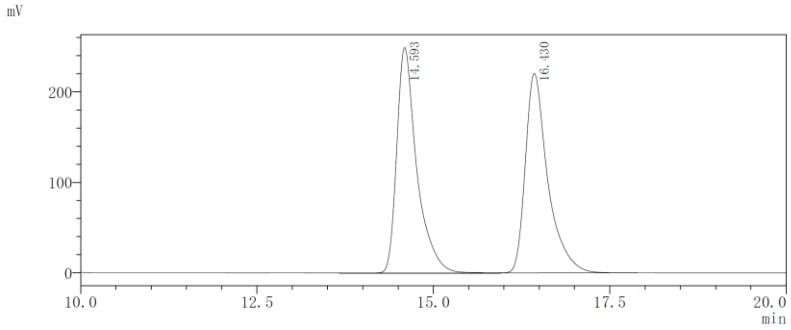

A Ch2 230nm

RetTime[min]	Area	Hight	Area%
14.593	4923723	249343	50.182
16.430	4888011	220513	49.818

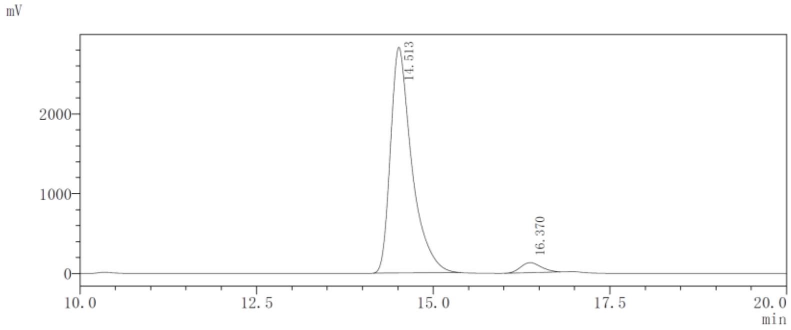

A Ch2 230nm

RetTime[min]	Area	Hight	Area%
14.513	59434252	2827234	95.946
16.370	2511405	125038	4.054

9. Crystal Structures of 4j

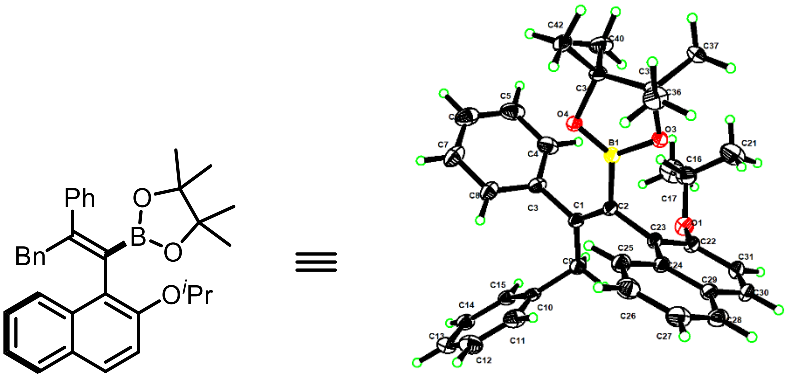

ORTEP plot of the crystal structure of 4j, and thermal ellipsoid is set at 50% probability.

X-ray crystallographic data of **4j**

CCDC number	2293683
Empirical formula	$C_{34}H_{37}BO_3$
Formula weight	504.44
Temperature	170 K
Wavelength	1.54178
Space group	P 21 21 21
Unit cell dimensions	a = 9.7702(3) Å = 90º
	b = 14.5939(5) Å = 90º
	c = 20.2249(6) Å = 90º
Volume	2883.77(16)
Z	4
Density	1.162
F(000)	1080.0
Completeness to theta = 74.655	1.74/0.98
Max. and min. transmission	0.754 and 0.677
R indices (all data)	R = 0.0430 (5419)<br>wR2 = 0.1152 (5799)

10. Enantiomerization Barrier Determination of 4j

The enantiomerization barrier, corresponding to the barrier to rotation for the following atropisomers, was obtained by kinetic of racemization of an enantiomer. The slope of the first order kinetic line gives the racemization constant ($k_{racemization} = 2 \times k_{enantiomerization}$). Eyring equation gives the enantiomerization barrier ($\Delta G^{\ddagger}_{enantiomerization}$) from enantiomerization constant ($k_{enantiomerization}$), R = Gas constant = 8.31451 J.K^{-1}.mol^{-1}, h = Planck constant = 6.62608 × 10^{-34} J.s and k_B = Boltzmann constant = 1.38066 × 10^{-23} J.K^{-1}.

$$\Delta G^{\ddagger}_{\text{enantiomerization}} = RT_1.\ln\frac{k_B T_1}{\text{h}k_{\text{enantiomerization}}}$$

The half-life time ($t_{1/2}$) given below, is at the temperature used for the kinetic.

Racemization of **4j** in isopropanol at 80 °C.

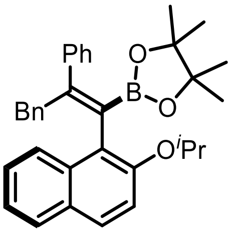

4j

Time (second)	Enantiomeric Excess(ee)	First Order Racemization ln(ee$_0$/ee$_t$)
0	95	0
3600	91	0.043017385
5400	89	0.065240522
7200	87	0.087968773
9000	85	0.111225635
10800	83	0.135036284
12600	81	0.159427737
14400	78	0.197168065
16200	76	0.223143551

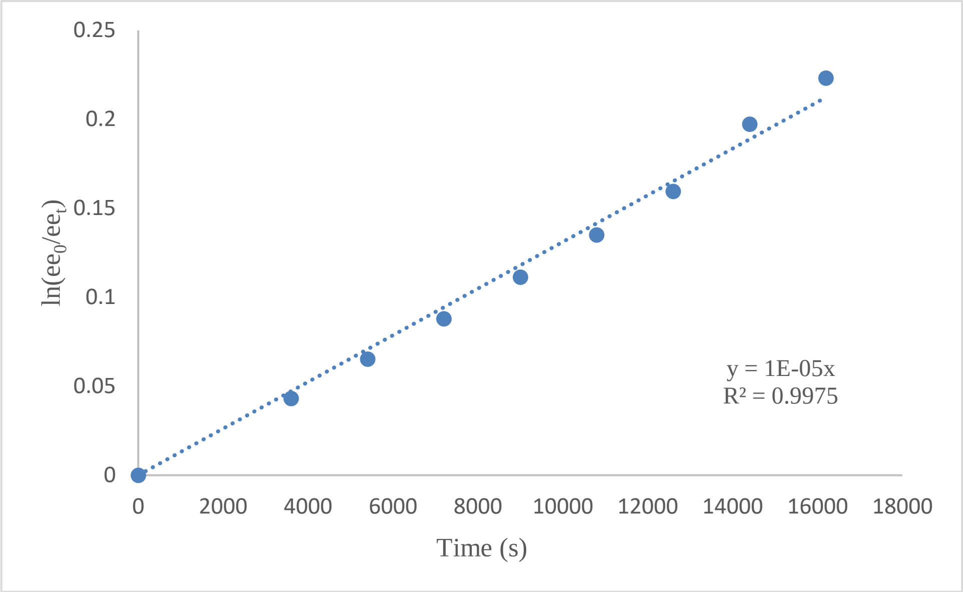

$k_{racemization}$ (80 °C) = 1×10^{-5} s^{-1}
$k_{enantiomerization}$ (80 °C) = 5×10^{-6} s^{-1}
$\Delta G^{\ddagger}_{\ enantiomerization}$ = 122.833 kJ/mol = 29.36 kcal/mol
$t_{1/2}$ (80 °C) = 19.3 hours

11. ^{1}H, ^{13}C ^{19}F and ^{31}P NMR Spectra of Title Compounds

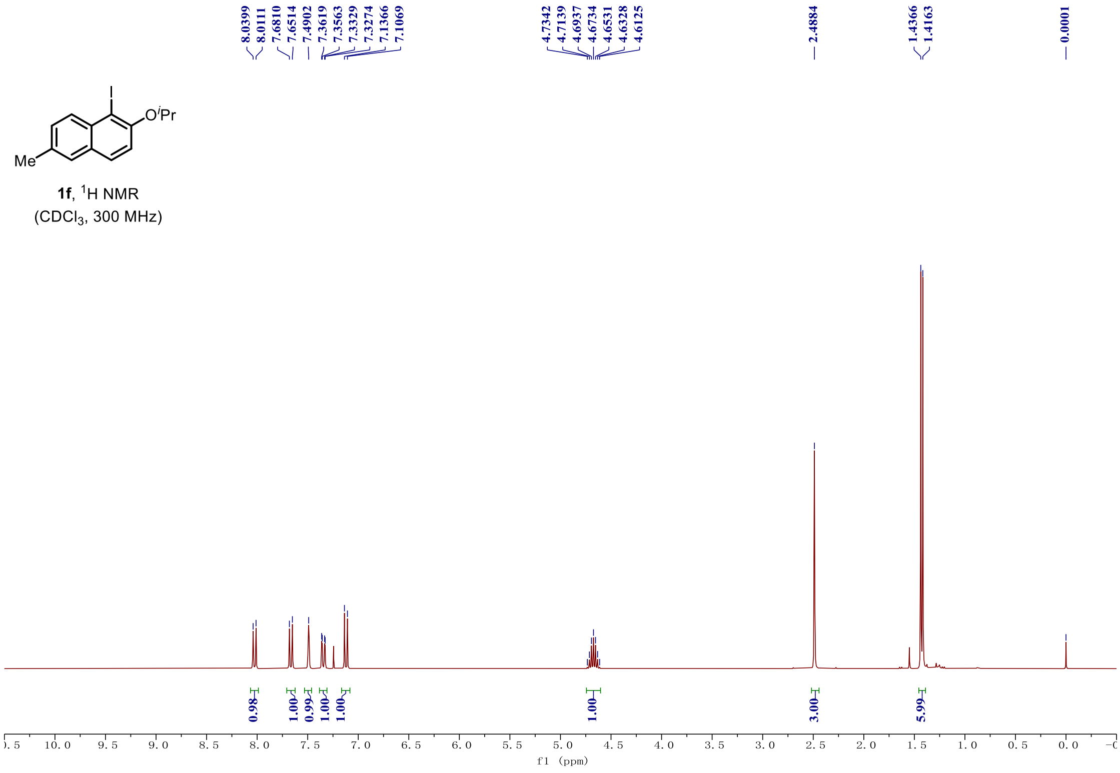

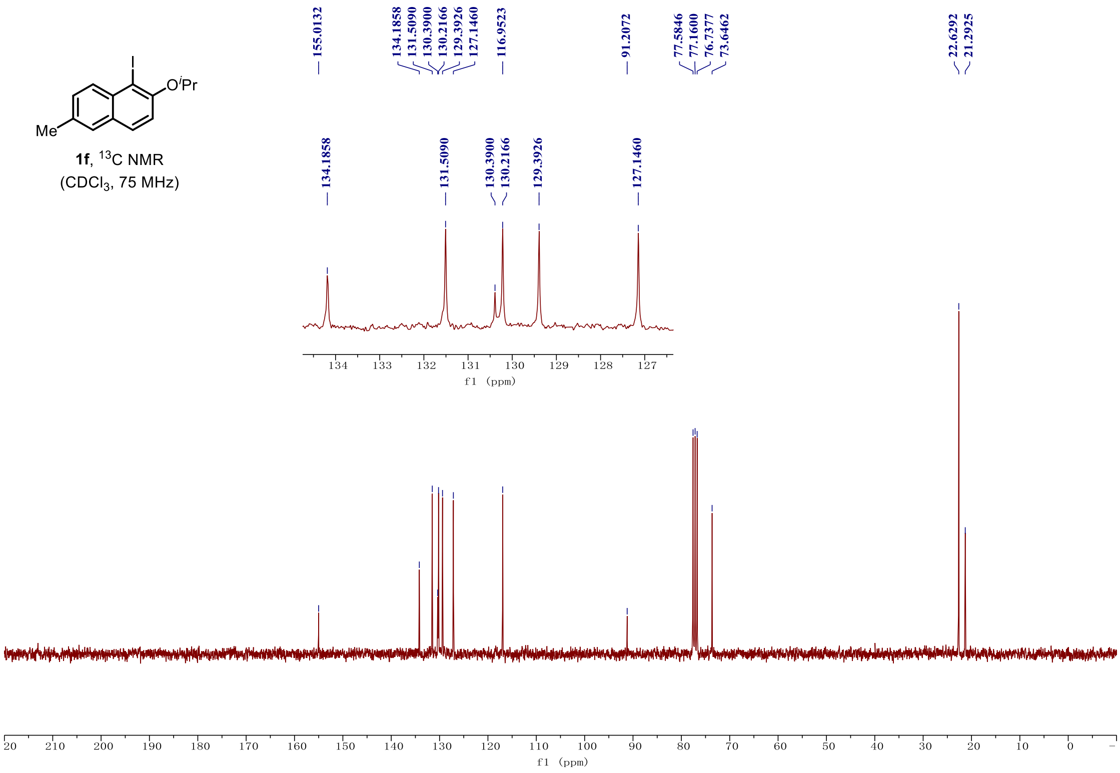

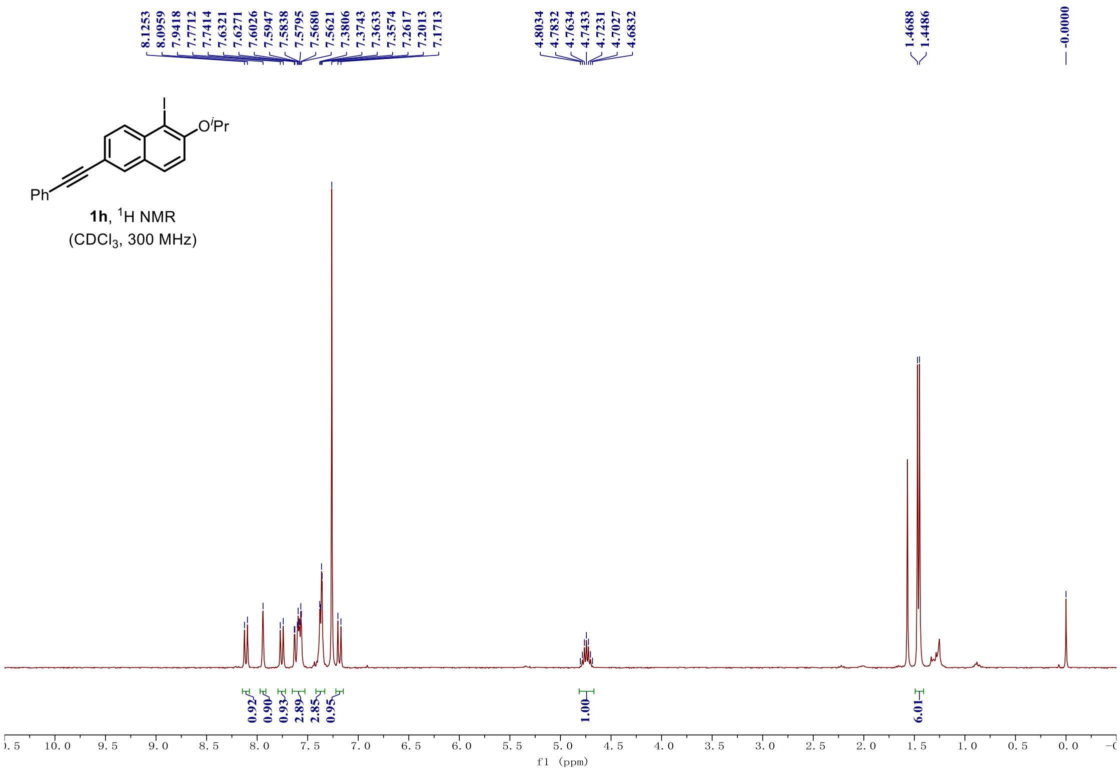
8.1253
8.0959
7.9418
7.7712
7.7414
7.6321
7.6271
7.6026
7.5947
7.5838
7.5795
7.5680
7.5621
7.3806
7.3743
7.3633
7.3574
7.2617
7.2013
7.1713
4.8034
4.7832
4.7634
4.7433
4.7231
4.7027
4.6832
1.4688
1.4486
-0.0000
I
O^iPr
Ph
1h, 1H NMR
(CDCl3, 300 MHz)
0.92
0.90
0.93
2.89
2.85
0.95
1.00
6.01
f1 (ppm)

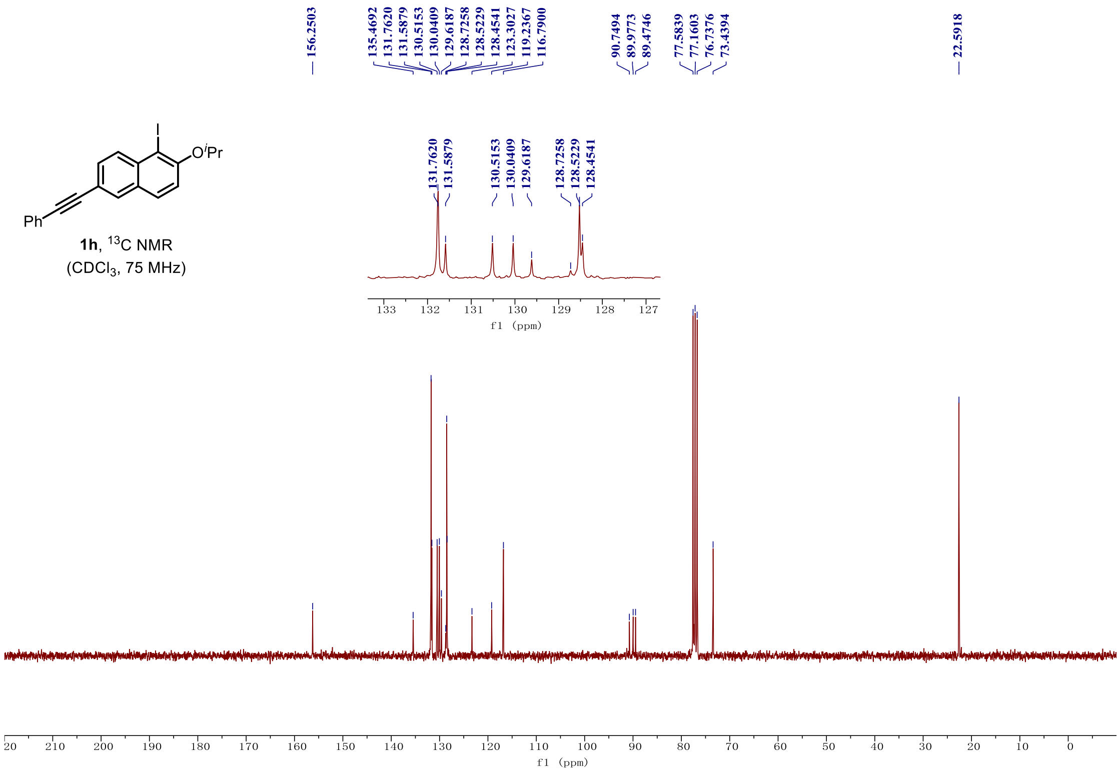
156.2503
135.4692
131.7620
131.5879
130.5153
130.0409
129.6187
128.7258
128.5229
128.4541
123.3027
119.2367
116.7900
90.7494
89.9773
89.4746
77.5839
77.1603
76.7376
73.4394
22.5918
I
O^iPr
Ph
1h, 13C NMR
(CDCl3, 75 MHz)
f1 (ppm)

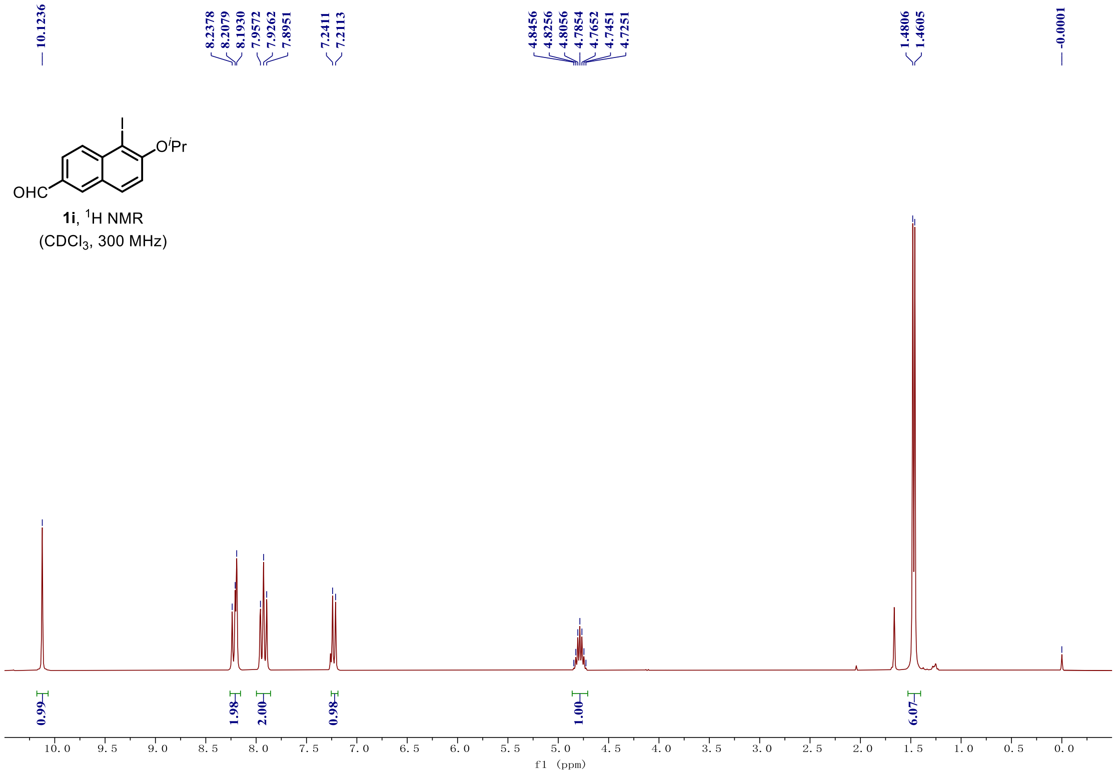

1i, ¹H NMR
($CDCl_3$, 300 MHz)

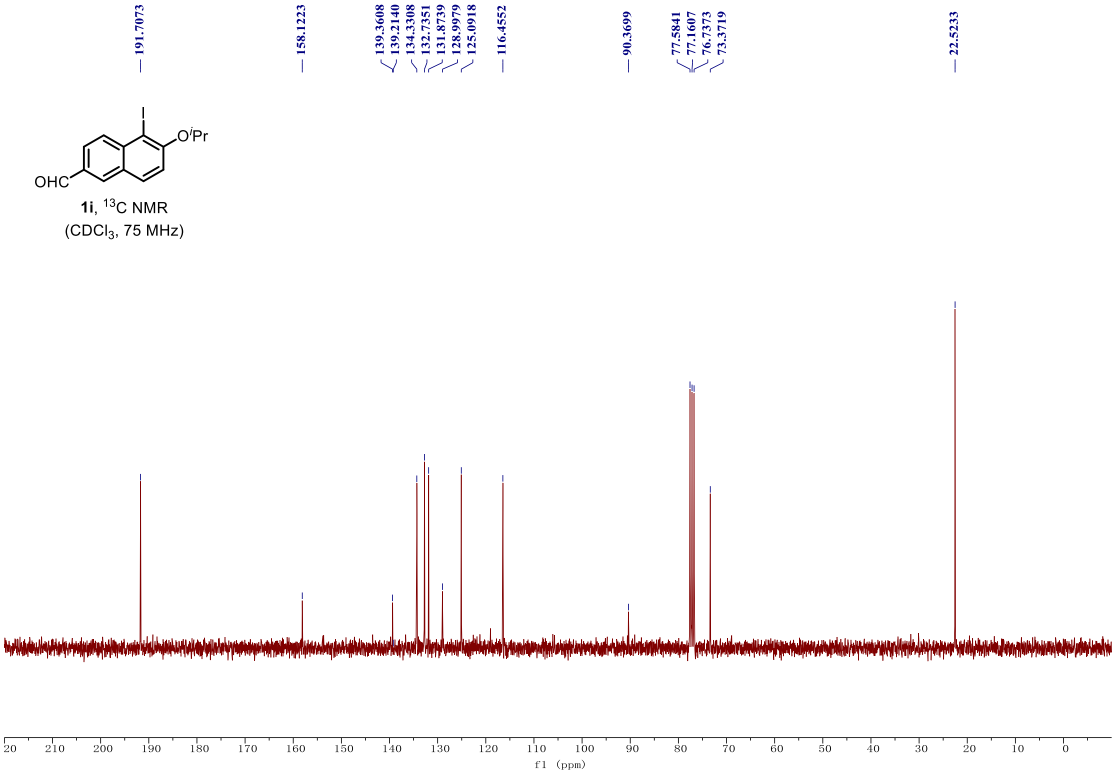

1i, ¹³C NMR
($CDCl_3$, 75 MHz)

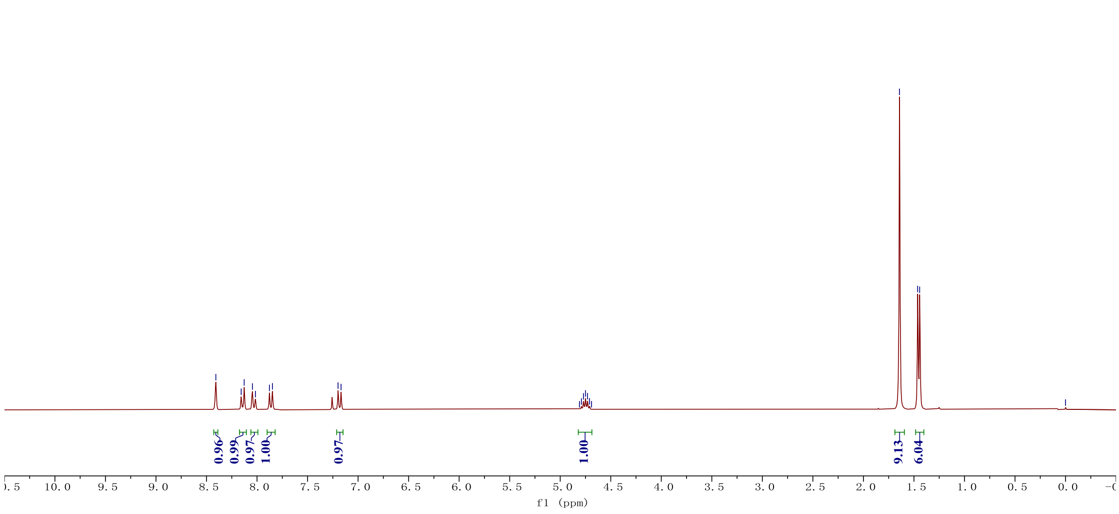

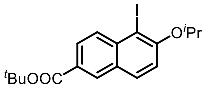

1j, ^{1}H NMR
(CDCl$_3$, 300 MHz)

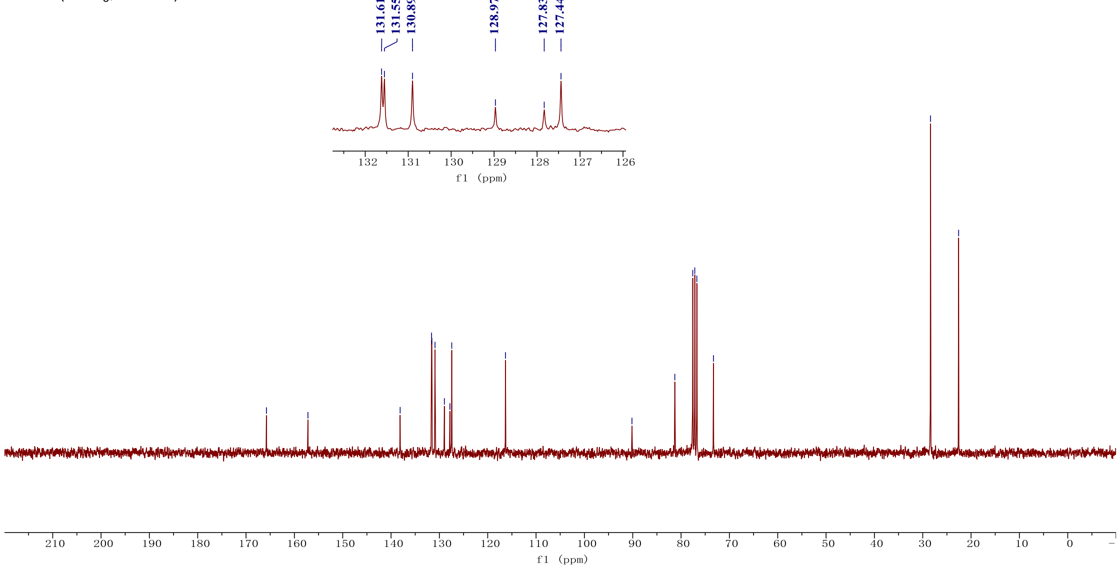

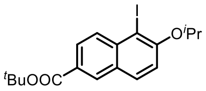

1j, ^{13}C NMR
(CDCl$_3$, 75 MHz)

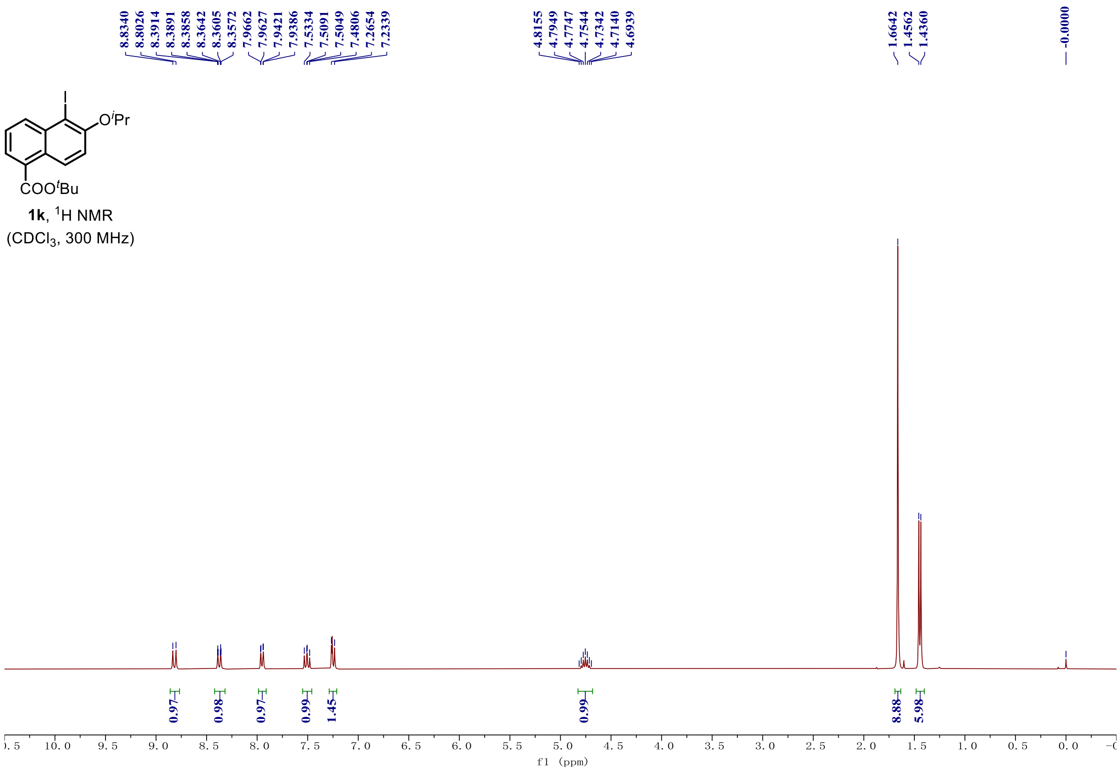
8.8340
8.8026
8.3914
8.3891
8.3858
8.3642
8.3605
8.3572
7.9662
7.9627
7.9421
7.9386
7.5334
7.5091
7.5049
7.4806
7.2654
7.2339
4.8155
4.7949
4.7747
4.7544
4.7342
4.7140
4.6939
1.6642
1.4562
1.4360
-0.0000
1k, ^{1}H NMR
($CDCl_3$, 300 MHz)
0.97
0.98
0.97
0.99
1.45
0.99
8.88
5.98
f1 (ppm)

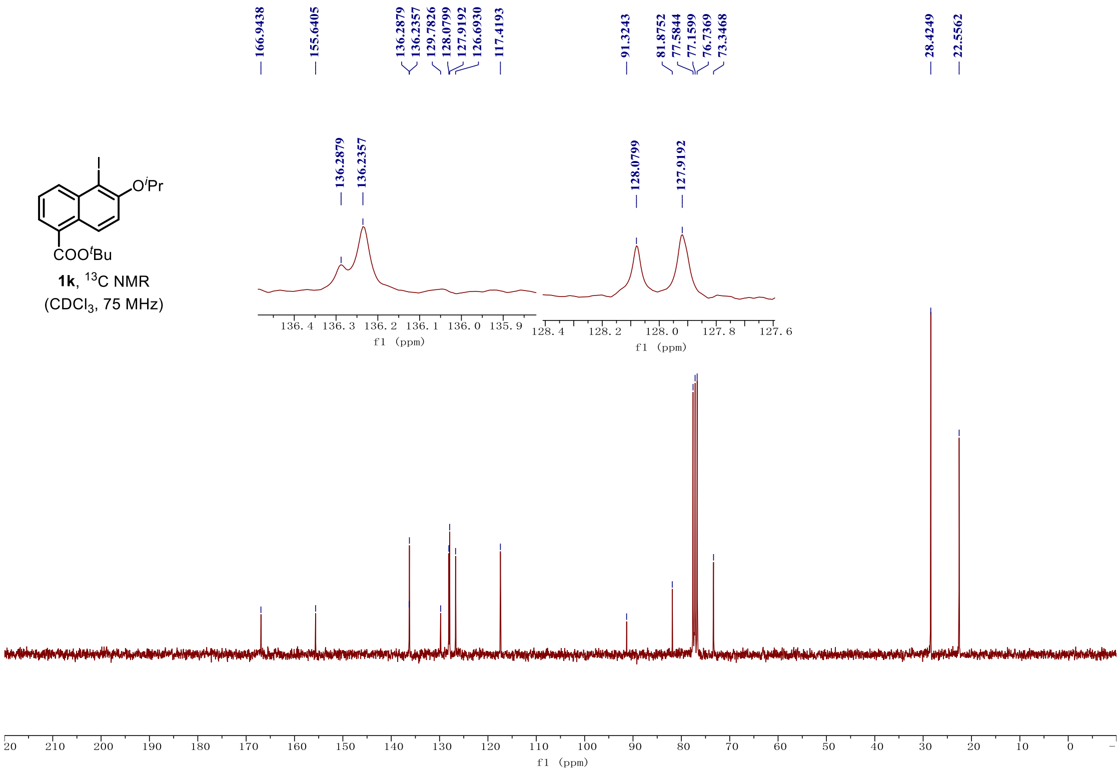
166.9438
155.6405
136.2879
136.2357
129.7826
128.0799
127.9192
126.6930
117.4193
91.3243
81.8752
77.5844
77.1599
76.7369
73.3468
28.4249
22.5562
1k, ^{13}C NMR
($CDCl_3$, 75 MHz)
f1 (ppm)

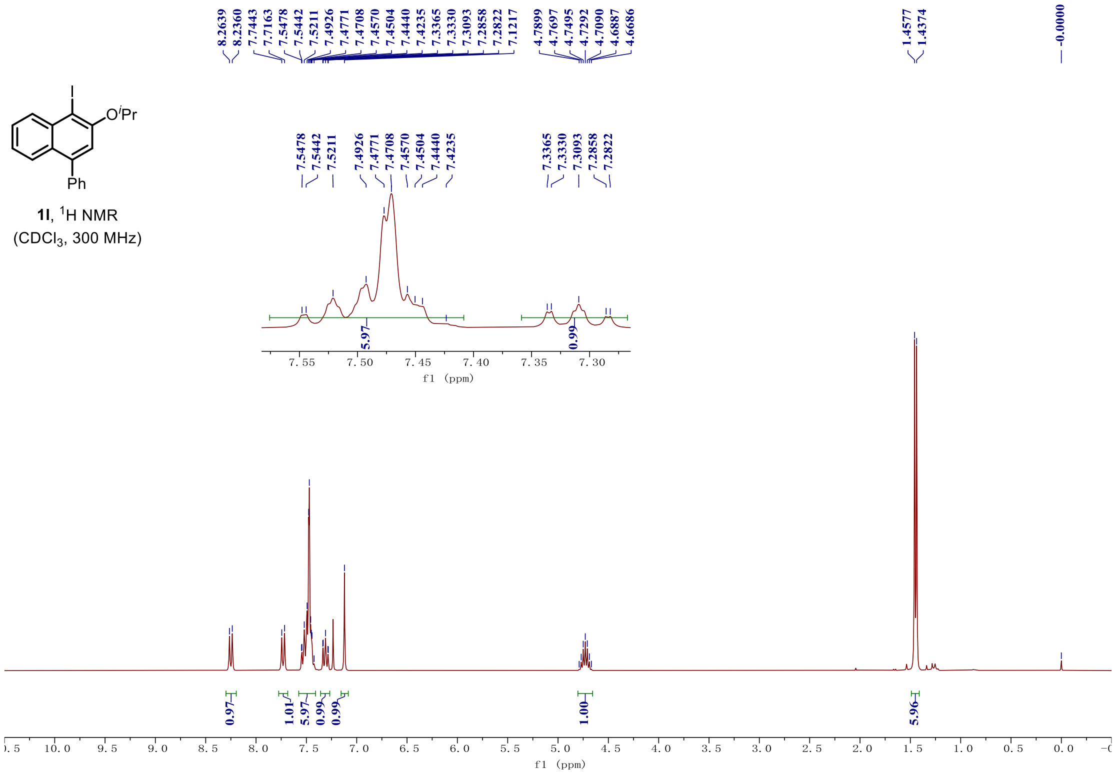

1l, ^{1}H NMR
($CDCl_3$, 300 MHz)

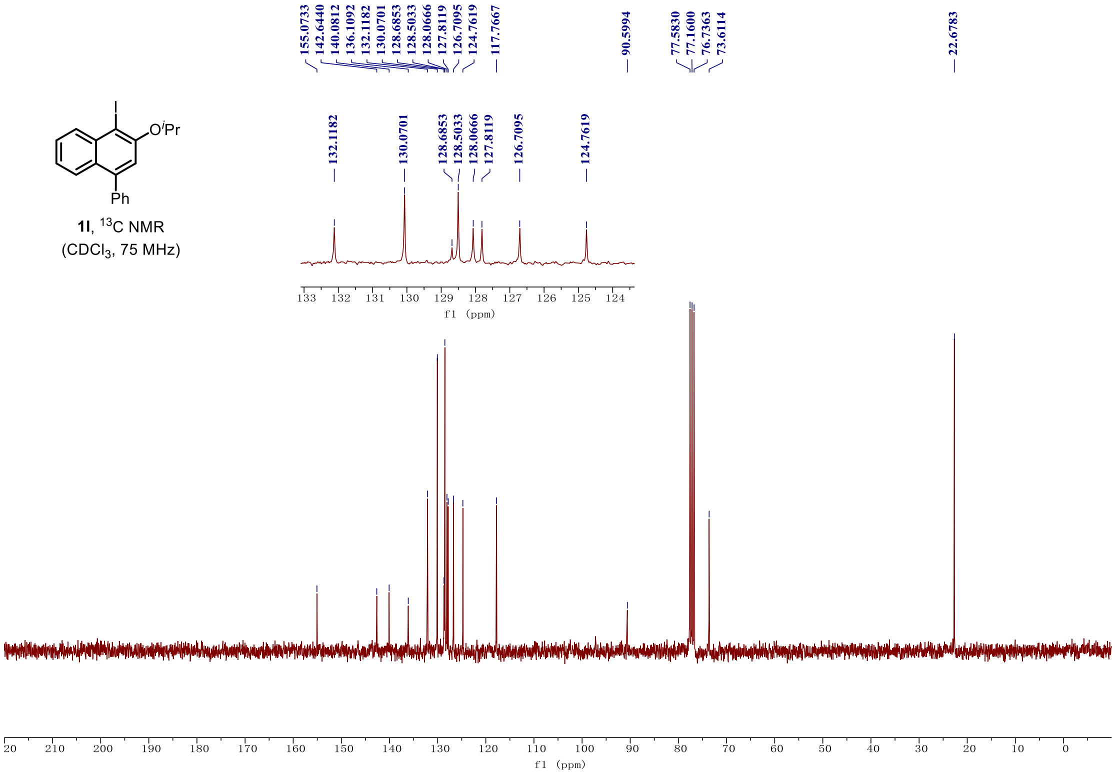

1l, ^{13}C NMR
($CDCl_3$, 75 MHz)

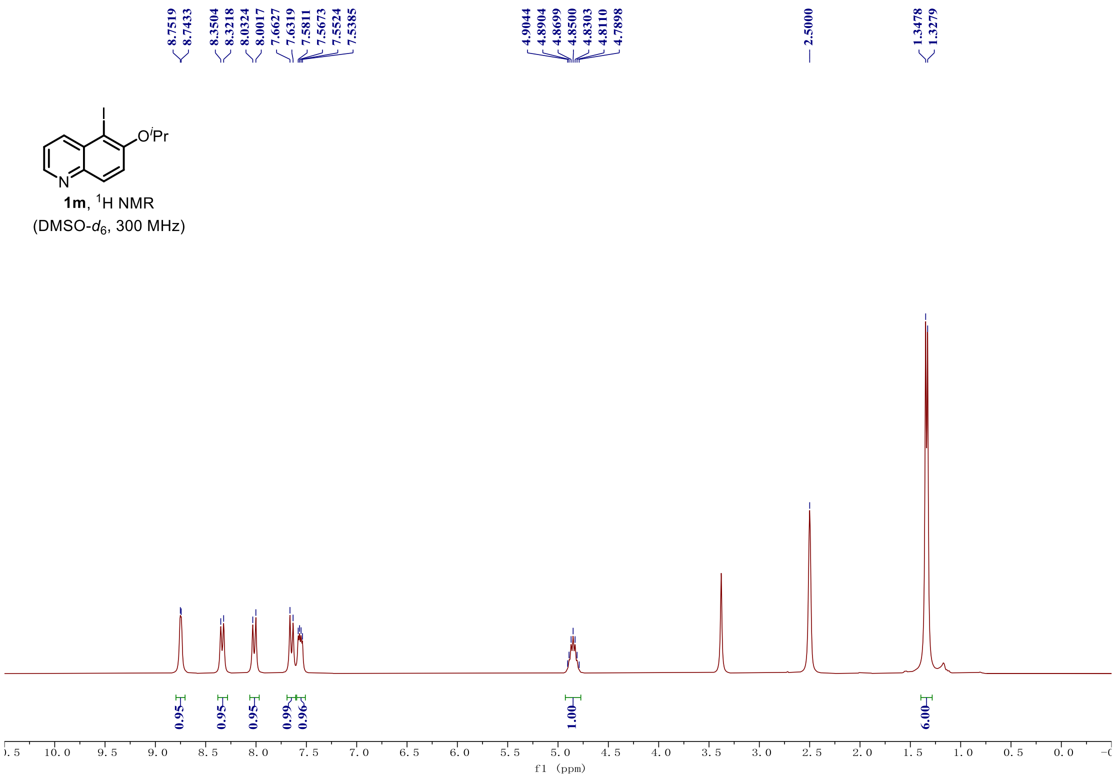
8.7519
8.7433
8.3504
8.3218
8.0324
8.0017
7.6627
7.6319
7.5811
7.5673
7.5524
7.5385
4.9044
4.8904
4.8699
4.8500
4.8303
4.8110
4.7898
2.5000
1.3478
1.3279
O^{i}Pr
I
N
1m, ^{1}H NMR
(DMSO-d_6, 300 MHz)
0.95
0.95
0.95
0.99
0.96
1.00
6.00
f1 (ppm)

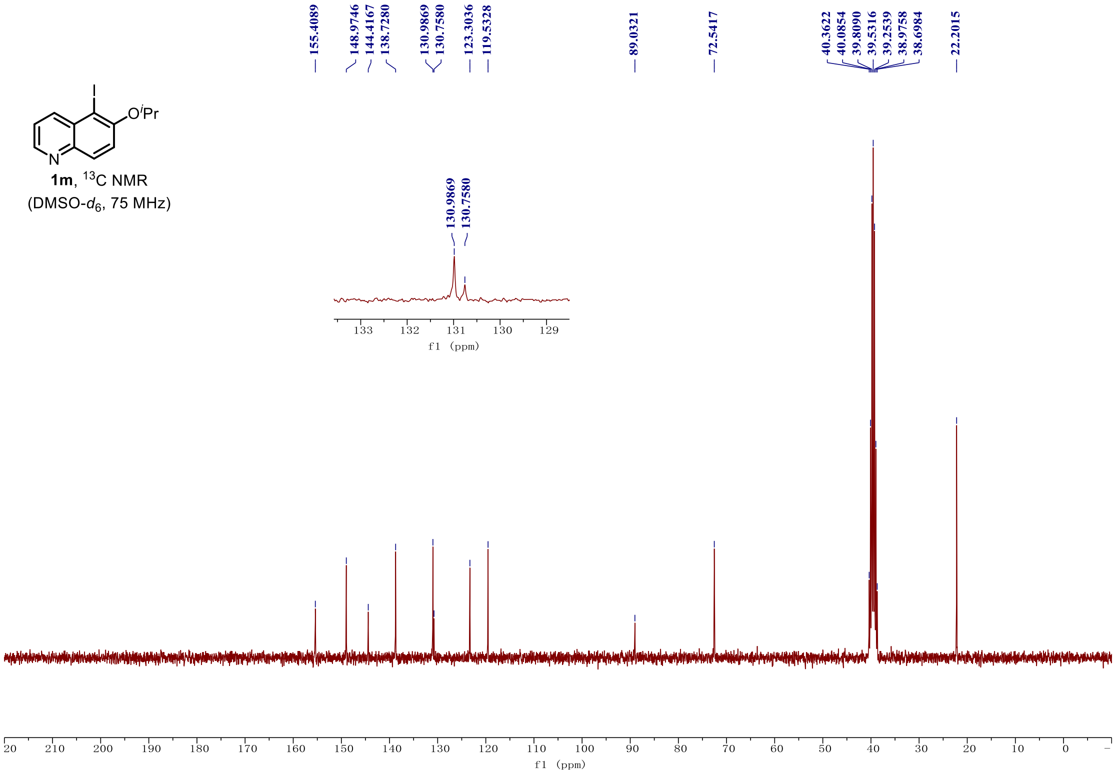
155.4089
148.9746
144.4167
138.7280
130.9869
130.7580
123.3036
119.5328
89.0321
72.5417
40.3622
40.0854
39.8090
39.5316
39.2539
38.9758
38.6984
22.2015
O^{i}Pr
I
N
1m, ^{13}C NMR
(DMSO-d_6, 75 MHz)
f1 (ppm)

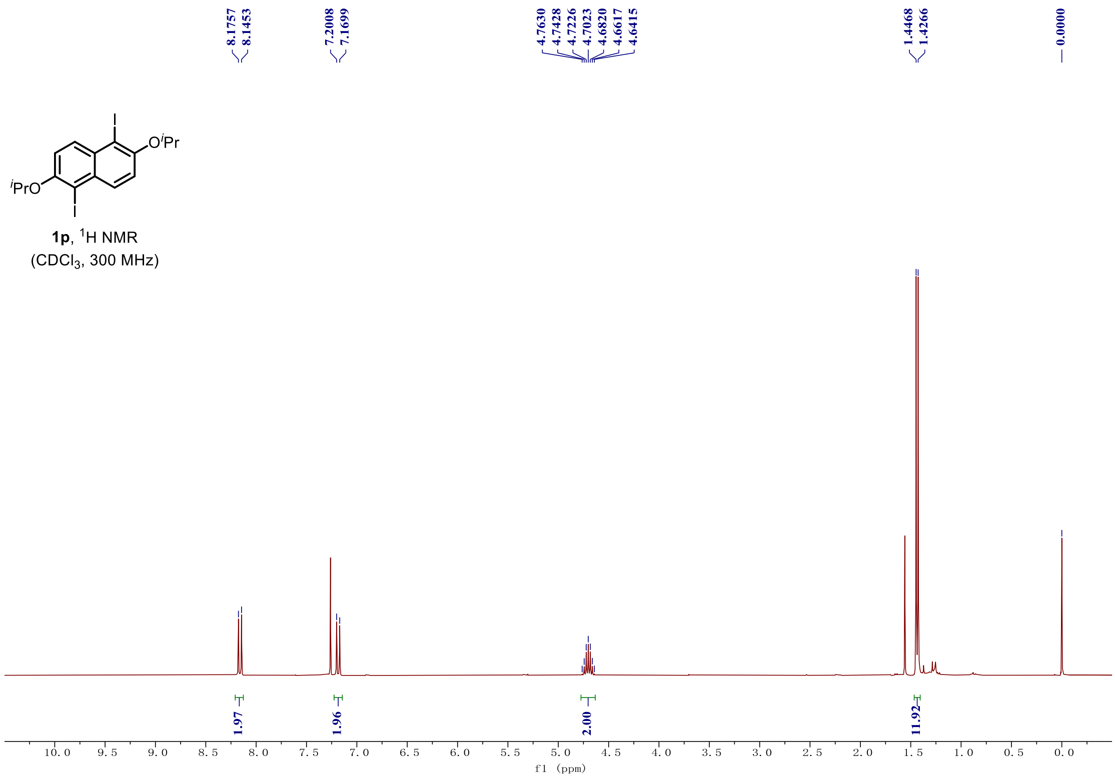

O^{i}Pr
^{i}PrO
I
1p, ^{1}H NMR
(CDCl$_3$, 300 MHz)
8.1757
8.1453
7.2008
7.1699
4.7630
4.7428
4.7226
4.7023
4.6820
4.6617
4.6415
1.4468
1.4266
0.0000
1.97
1.96
2.00
11.92
f1 (ppm)

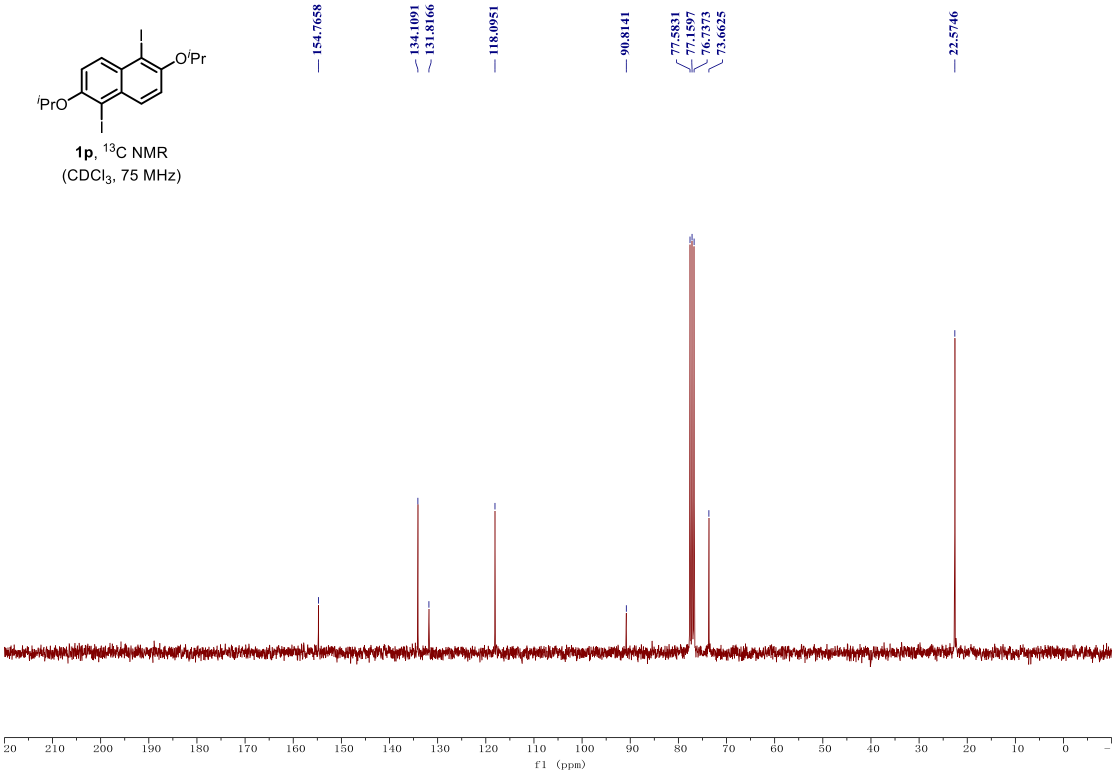

O^{i}Pr
^{i}PrO
I
1p, ^{13}C NMR
(CDCl$_3$, 75 MHz)
154.7658
134.1091
131.8166
118.0951
90.8141
77.5831
77.1597
76.7373
73.6625
22.5746
f1 (ppm)

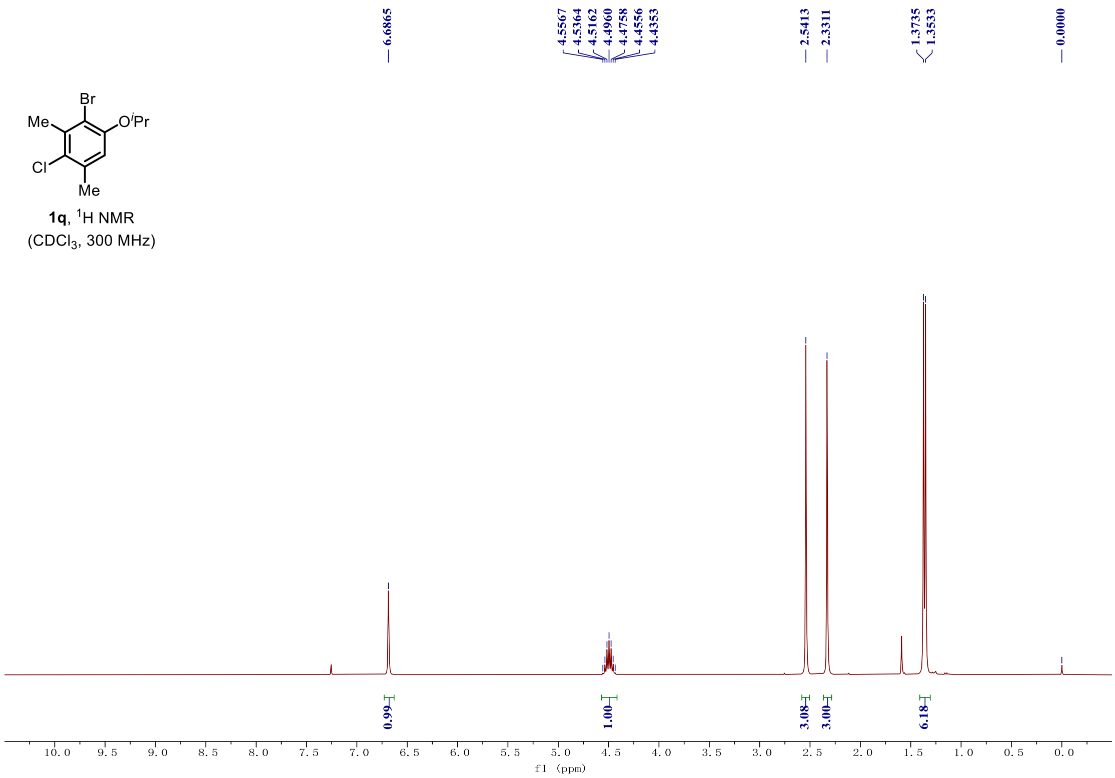

Br
Me
O^{i}Pr
Cl
Me
1q, ^{1}H NMR
($CDCl_3$, 300 MHz)
6.6865
4.5567
4.5364
4.5162
4.4960
4.4758
4.4556
4.4353
2.5413
2.3311
1.3735
1.3533
0.0000
0.99
1.00
3.08
3.00
6.18
10.0 9.5 9.0 8.5 8.0 7.5 7.0 6.5 6.0 5.5 5.0 4.5 4.0 3.5 3.0 2.5 2.0 1.5 1.0 0.5 0.0
f1 (ppm)

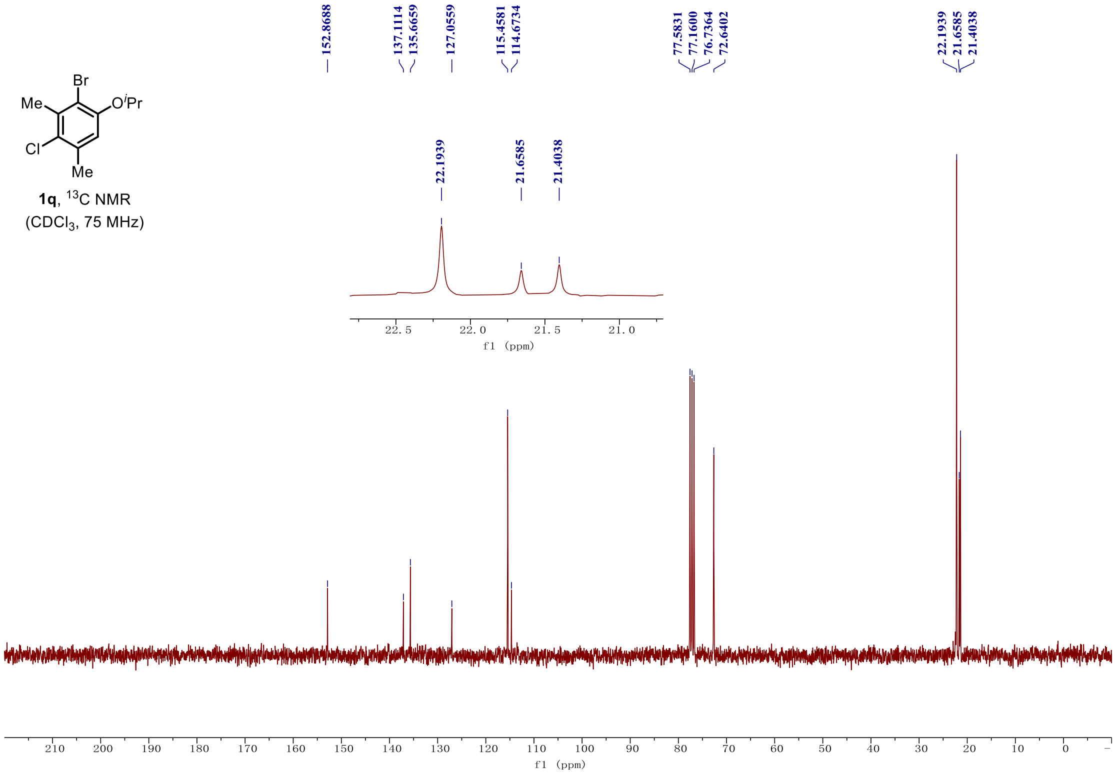

Br
Me
O^{i}Pr
Cl
Me
1q, ^{13}C NMR
($CDCl_3$, 75 MHz)
152.8688
137.1114
135.6659
127.0559
115.4581
114.6734
77.5831
77.1600
76.7364
72.6402
22.1939
21.6585
21.4038
22.1939
21.6585
21.4038
22.5 22.0 21.5 21.0
f1 (ppm)
210 200 190 180 170 160 150 140 130 120 110 100 90 80 70 60 50 40 30 20 10 0
f1 (ppm)

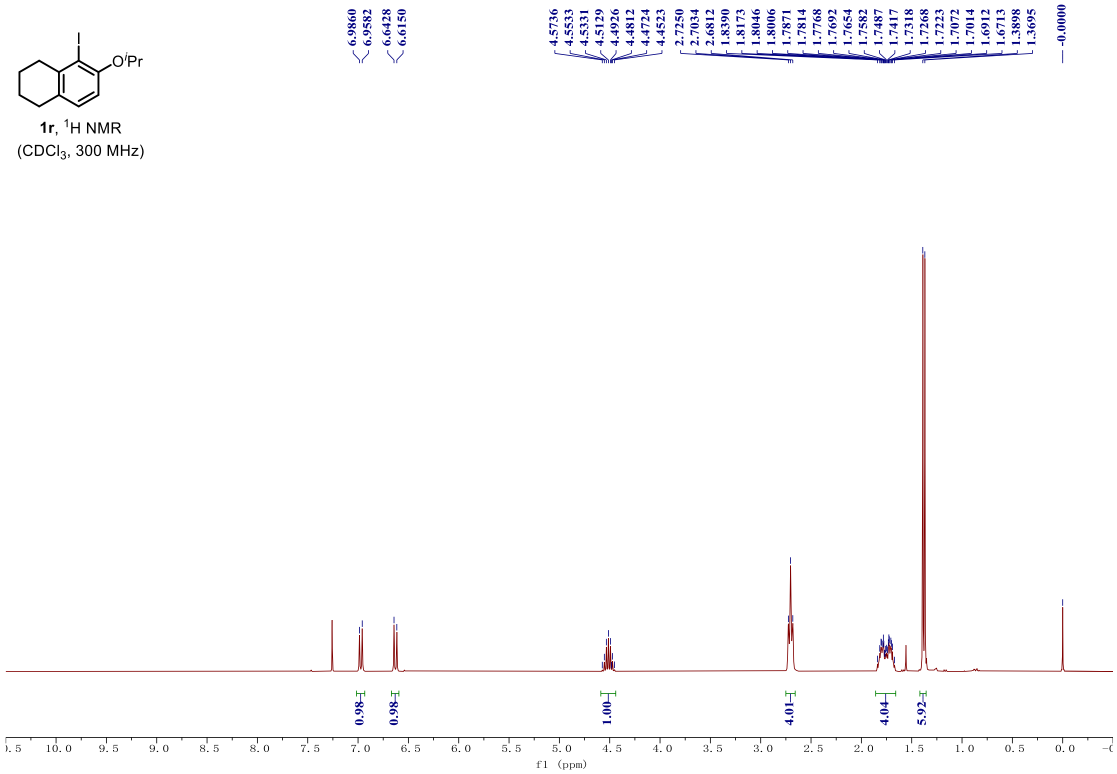
O^{i}Pr
I
1r, ^{1}H NMR
(CDCl$_3$, 300 MHz)
6.9860
6.9582
6.6428
6.6150
4.5736
4.5533
4.5331
4.5129
4.4926
4.4812
4.4724
4.4523
2.7250
2.7034
2.6812
1.8390
1.8173
1.8046
1.8006
1.7871
1.7814
1.7768
1.7692
1.7654
1.7582
1.7487
1.7417
1.7318
1.7268
1.7223
1.7072
1.7014
1.6912
1.6713
1.3898
1.3695
-0.0000
0.98
0.98
1.00
4.01
4.04
5.92
f1 (ppm)

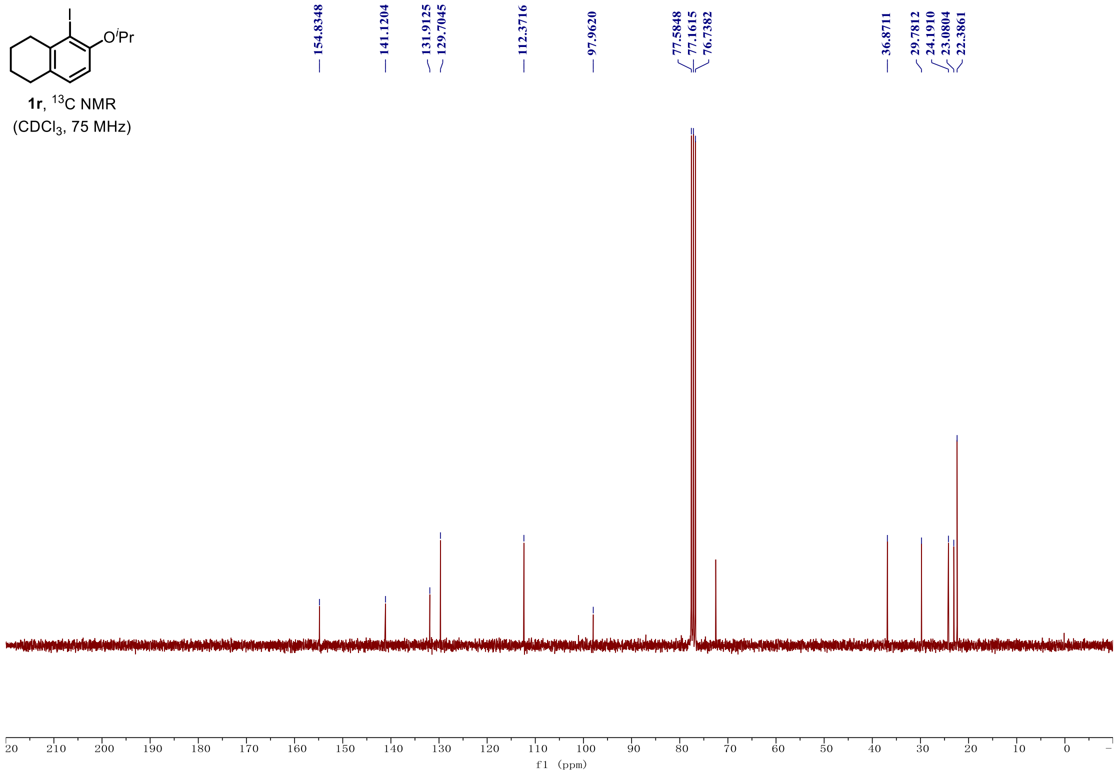
O^{i}Pr
I
1r, ^{13}C NMR
(CDCl$_3$, 75 MHz)
154.8348
141.1204
131.9125
129.7045
112.3716
97.9620
77.5848
77.1615
76.7382
36.8711
29.7812
24.1910
23.0804
22.3861
f1 (ppm)

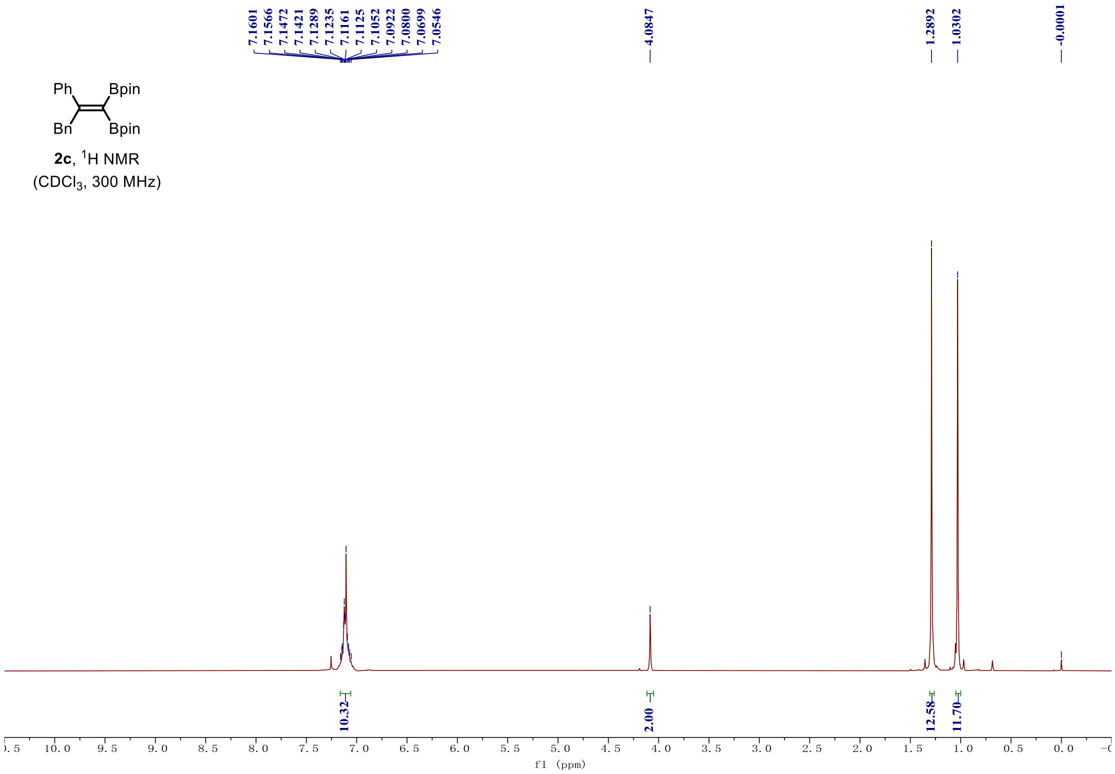
7.1601 7.1566 7.1472 7.1421 7.1289 7.1235 7.1161 7.1125 7.1052 7.0922 7.0800 7.0699 7.0546
4.0847
1.2892
1.0302
-0.0001
Ph Bpin
Bn Bpin
2c, 1H NMR
(CDCl3, 300 MHz)
10.32
2.00
12.58
11.70
f1 (ppm)

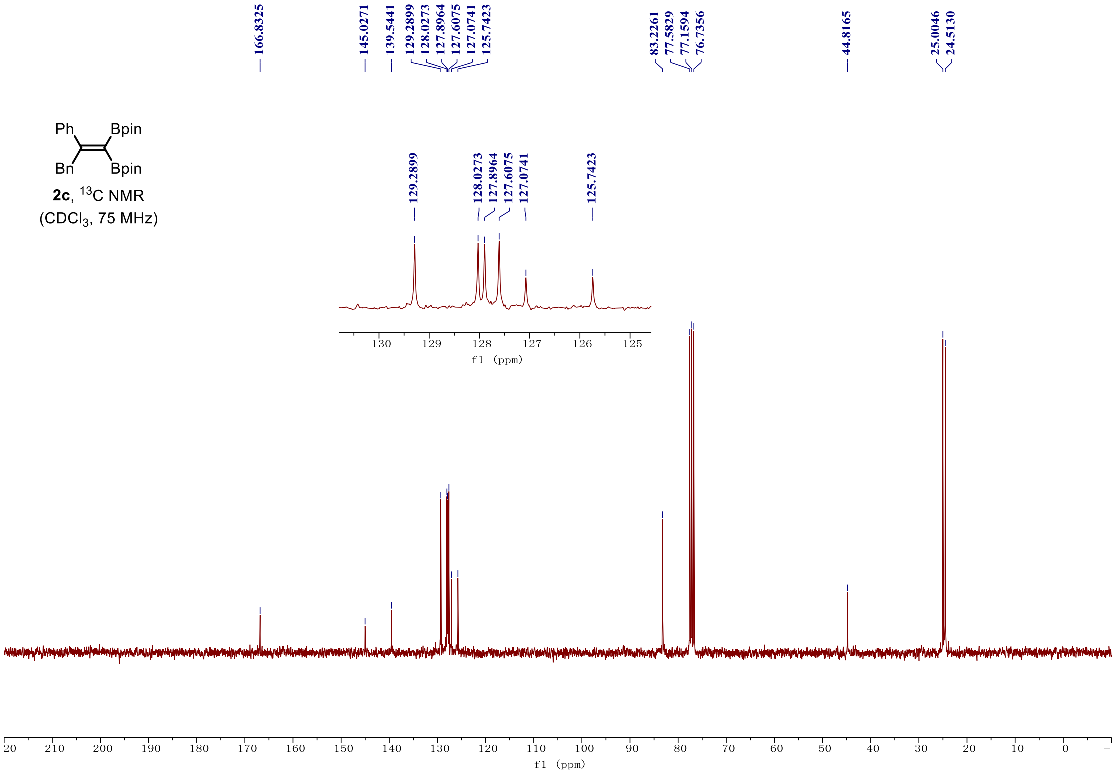
166.8325
145.0271
139.5441
129.2899 128.0273 127.8964 127.6075 127.0741 125.7423
83.2261 77.5829 77.1594 76.7356
44.8165
25.0046 24.5130
Ph Bpin
Bn Bpin
2c, 13C NMR
(CDCl3, 75 MHz)
129.2899
128.0273 127.8964 127.6075 127.0741
125.7423
f1 (ppm)
f1 (ppm)

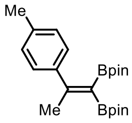

2d, ^{1}H NMR
($CDCl_3$, 300 MHz)

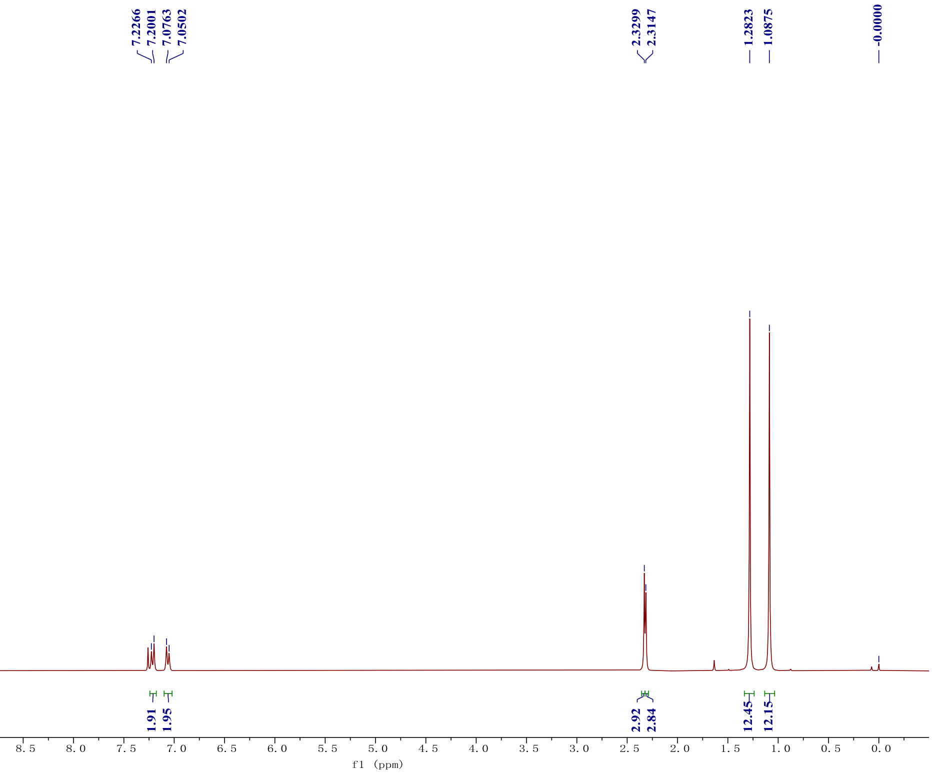

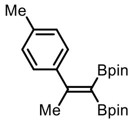

2d, ^{13}C NMR
($CDCl_3$, 75 MHz)

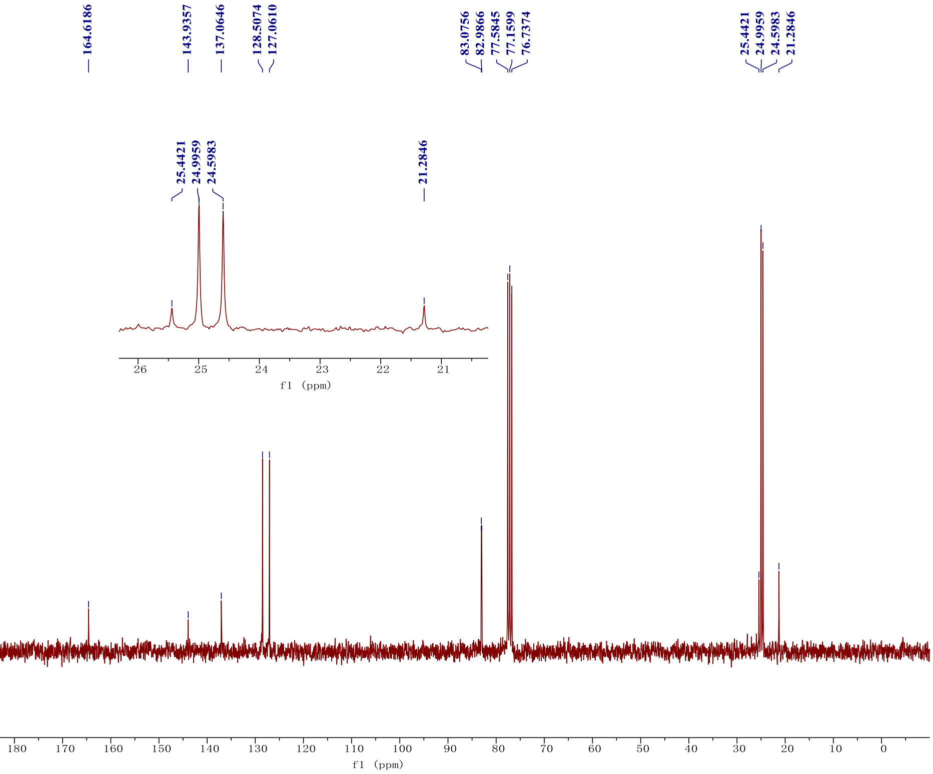

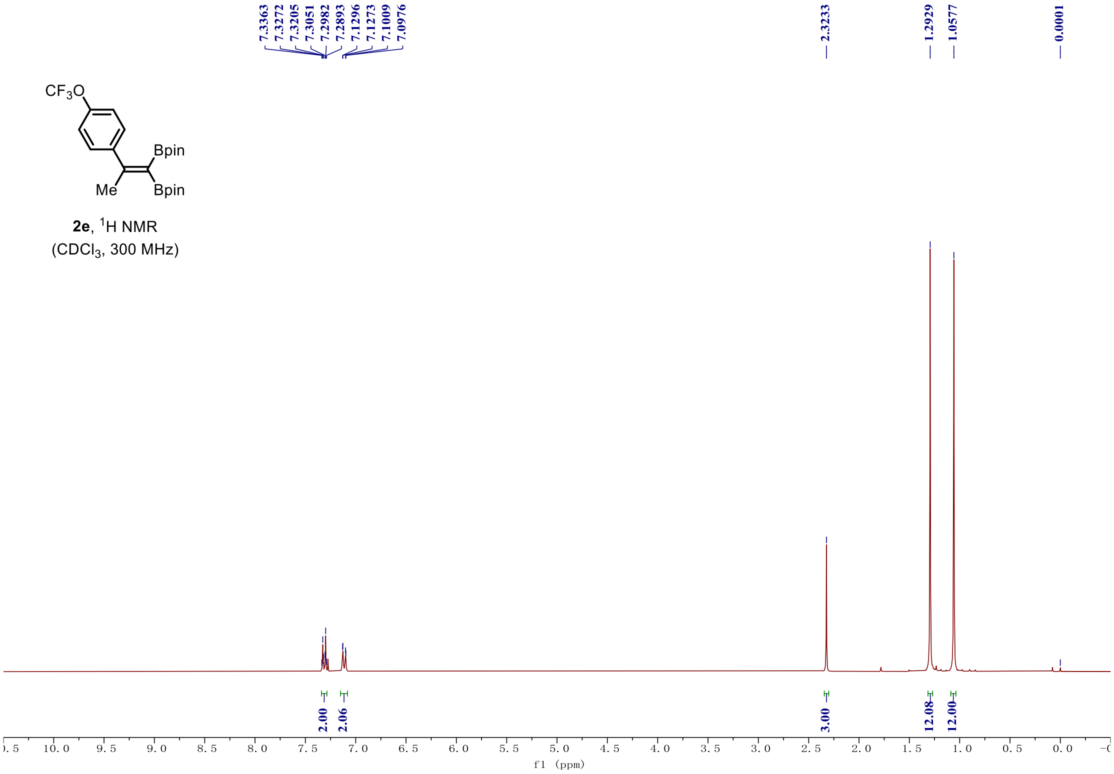

2e, ^{1}H NMR
($CDCl_3$, 300 MHz)

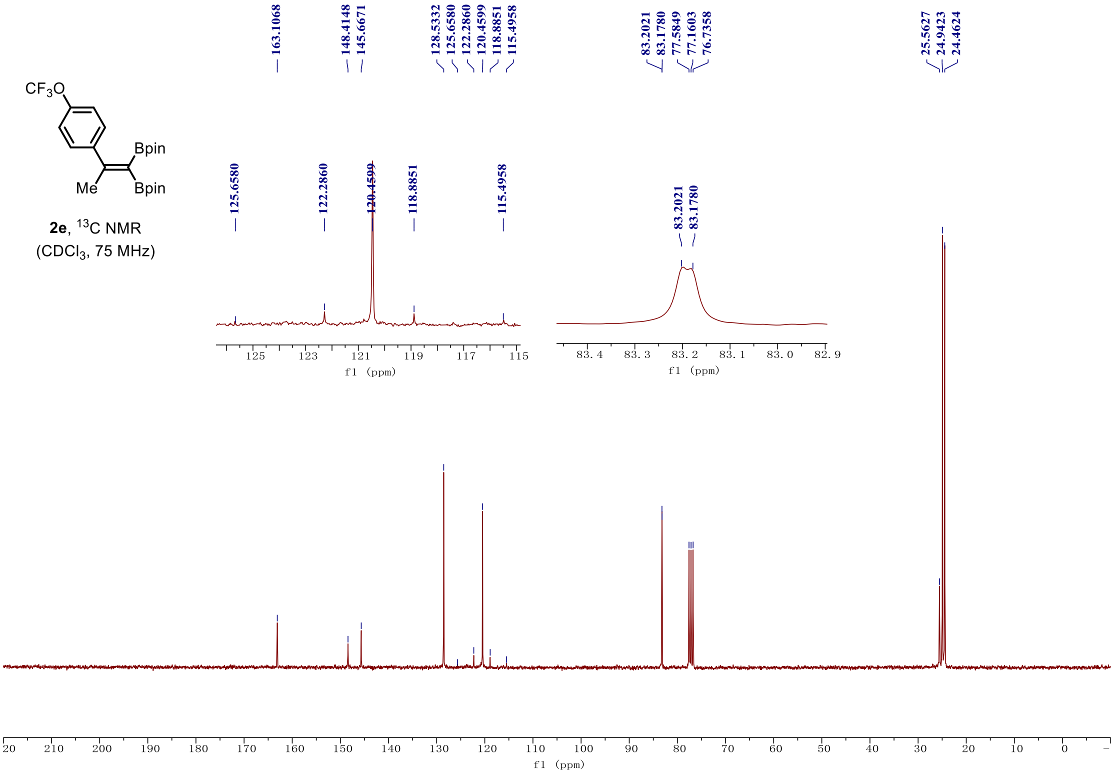

2e, ^{13}C NMR
($CDCl_3$, 75 MHz)

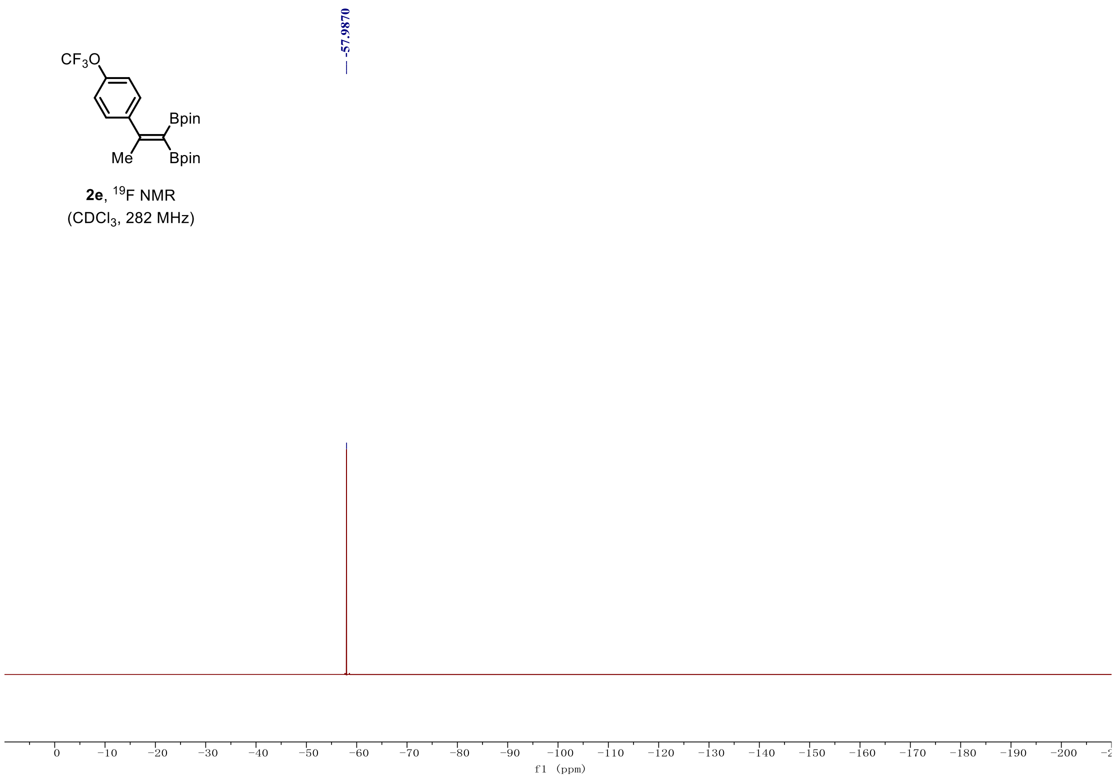
CF3O
Bpin
Me
Bpin
2e, 19F NMR
(CDCl3, 282 MHz)
-57.9870
f1 (ppm)

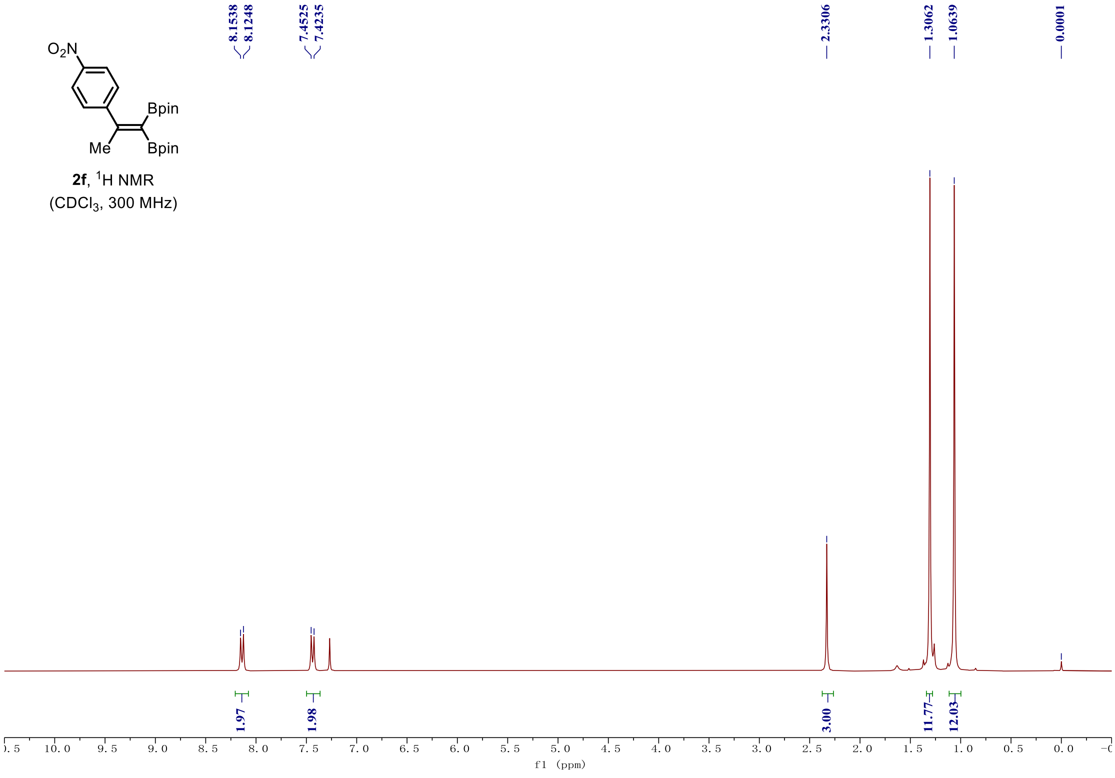
O2N
Bpin
Me
Bpin
2f, 1H NMR
(CDCl3, 300 MHz)
8.1538
8.1248
7.4525
7.4235
2.3306
1.3062
1.0639
0.0001
1.97
1.98
3.00
11.77
12.03
f1 (ppm)

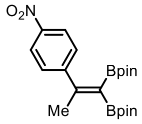

2f, ^{13}C NMR
($CDCl_3$, 75 MHz)

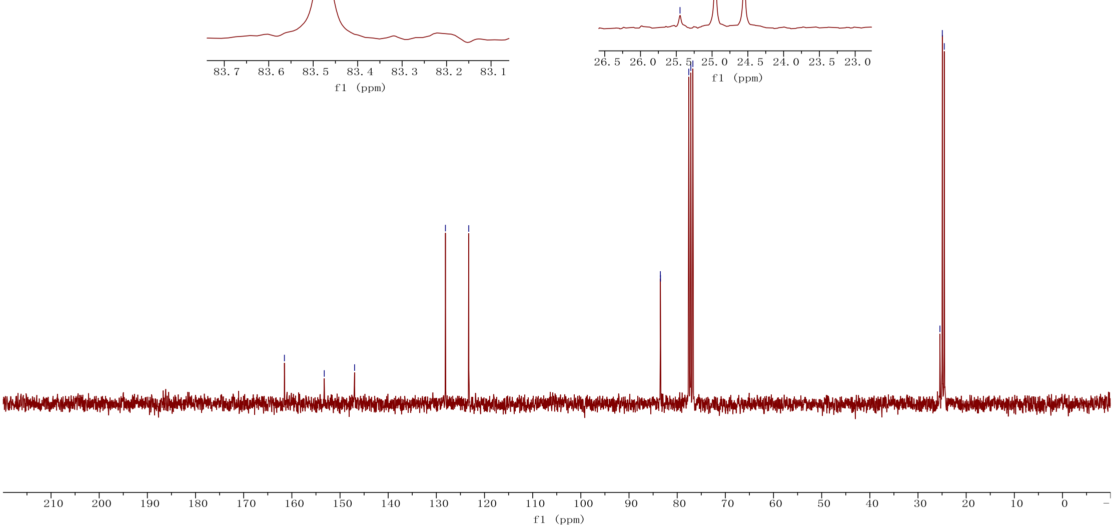

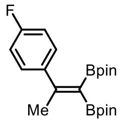

2g, ^{1}H NMR
($CDCl_3$, 300 MHz)

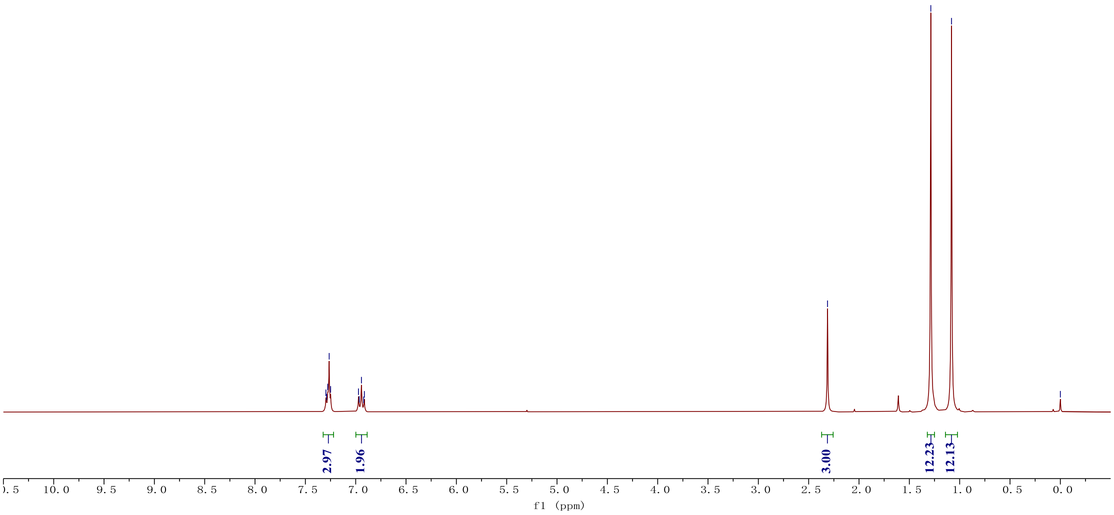

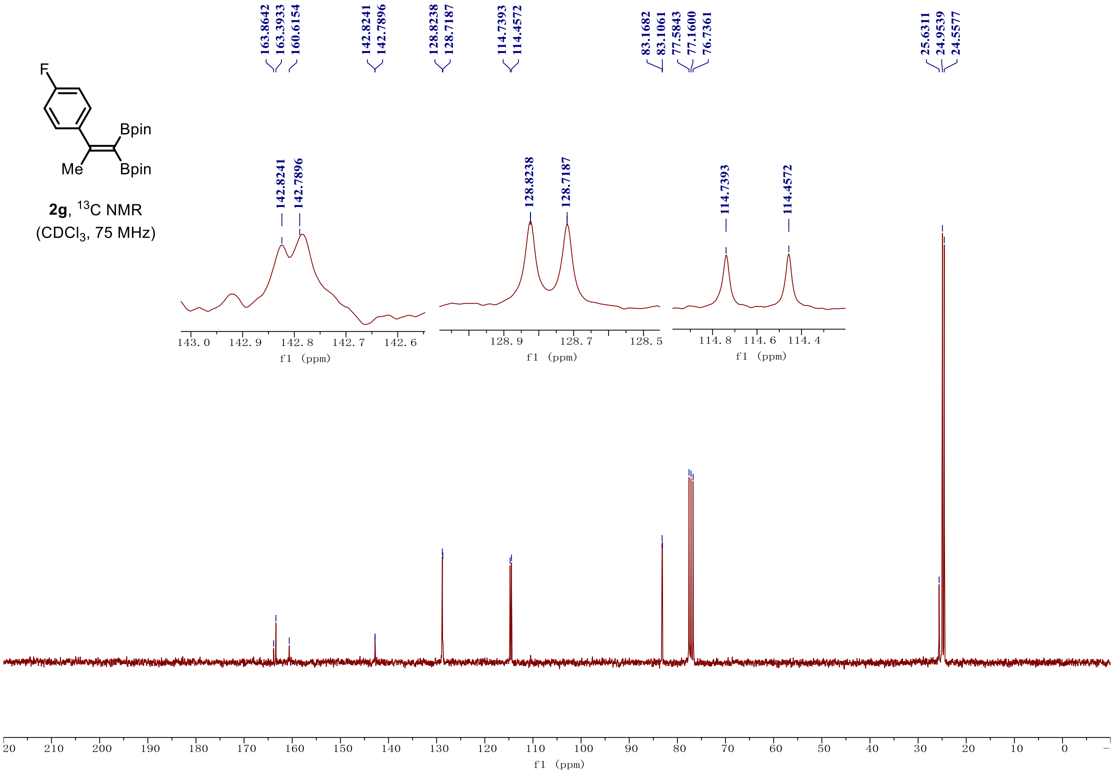

2g, ^{13}C NMR
($CDCl_3$, 75 MHz)

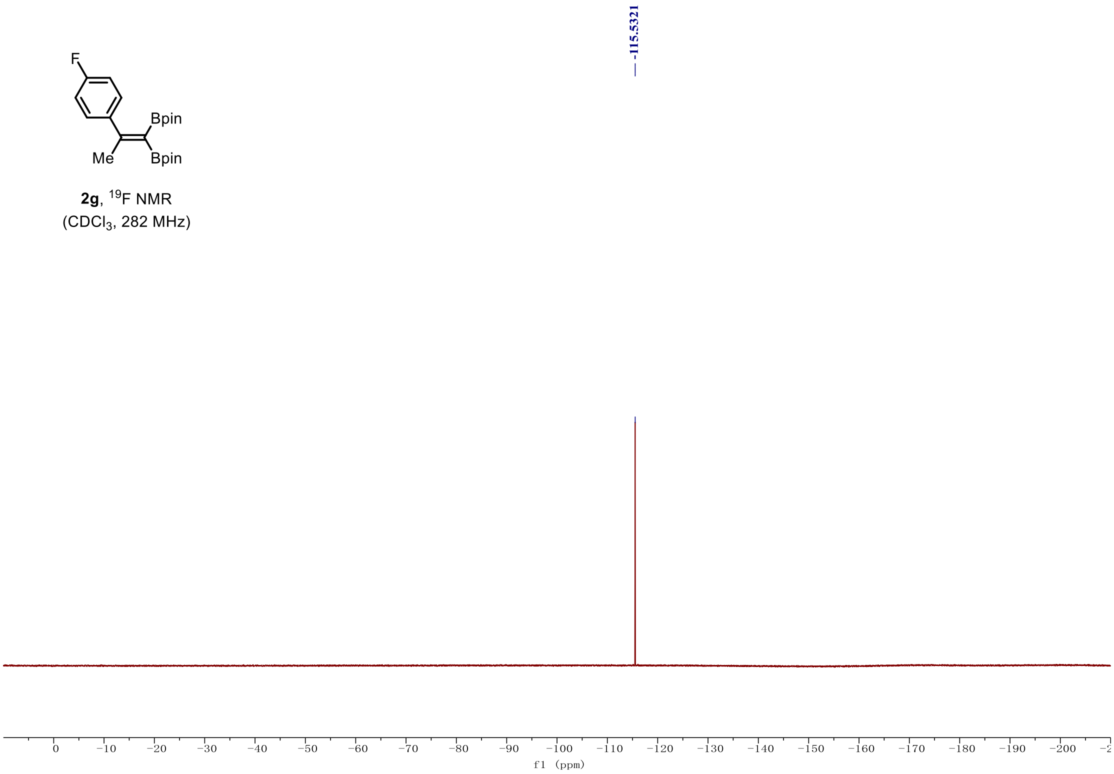

2g, ^{19}F NMR
($CDCl_3$, 282 MHz)

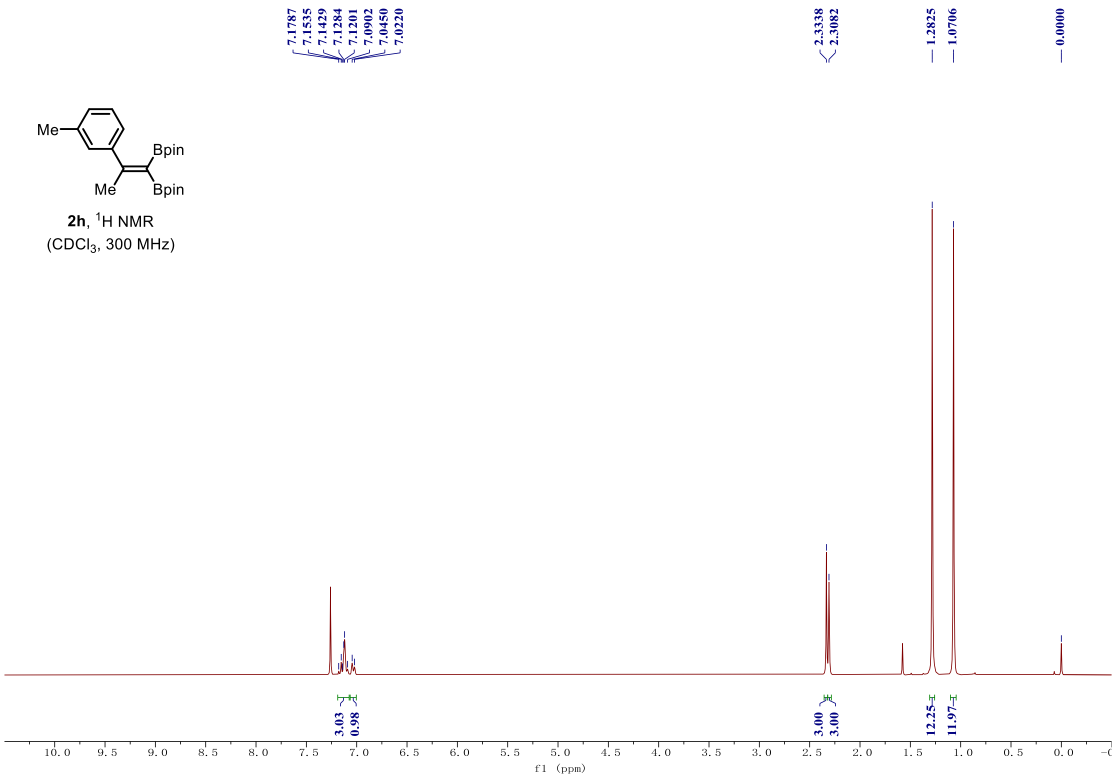

7.1787
7.1535
7.1429
7.1284
7.1201
7.0902
7.0450
7.0220
2.3338
2.3082
1.2825
1.0706
0.0000
Me
Bpin
Me
Bpin
2h, ^{1}H NMR
($CDCl_3$, 300 MHz)
3.03
0.98
3.00
3.00
12.25
11.97
f1 (ppm)

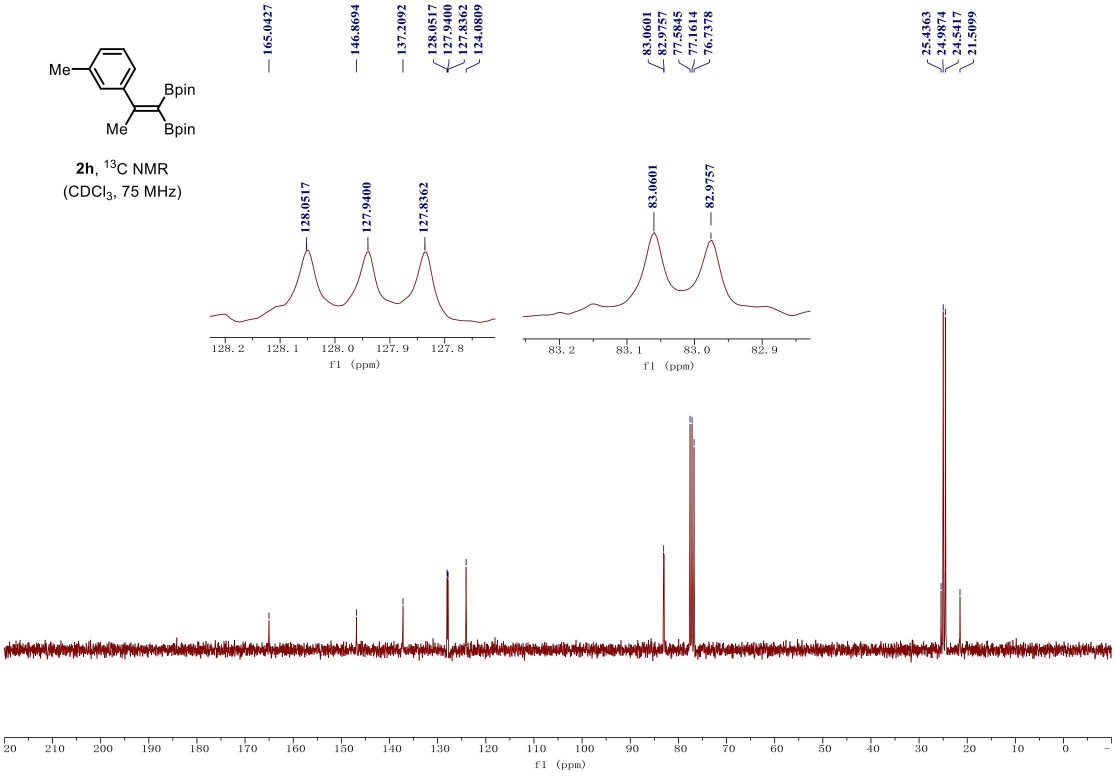

165.0427
146.8694
137.2092
128.0517
127.9400
127.8362
124.0809
83.0601
82.9757
77.5845
77.1614
76.7378
25.4363
24.9874
24.5417
21.5099
Me
Bpin
Me
Bpin
2h, ^{13}C NMR
($CDCl_3$, 75 MHz)
128.0517
127.9400
127.8362
83.0601
82.9757
f1 (ppm)

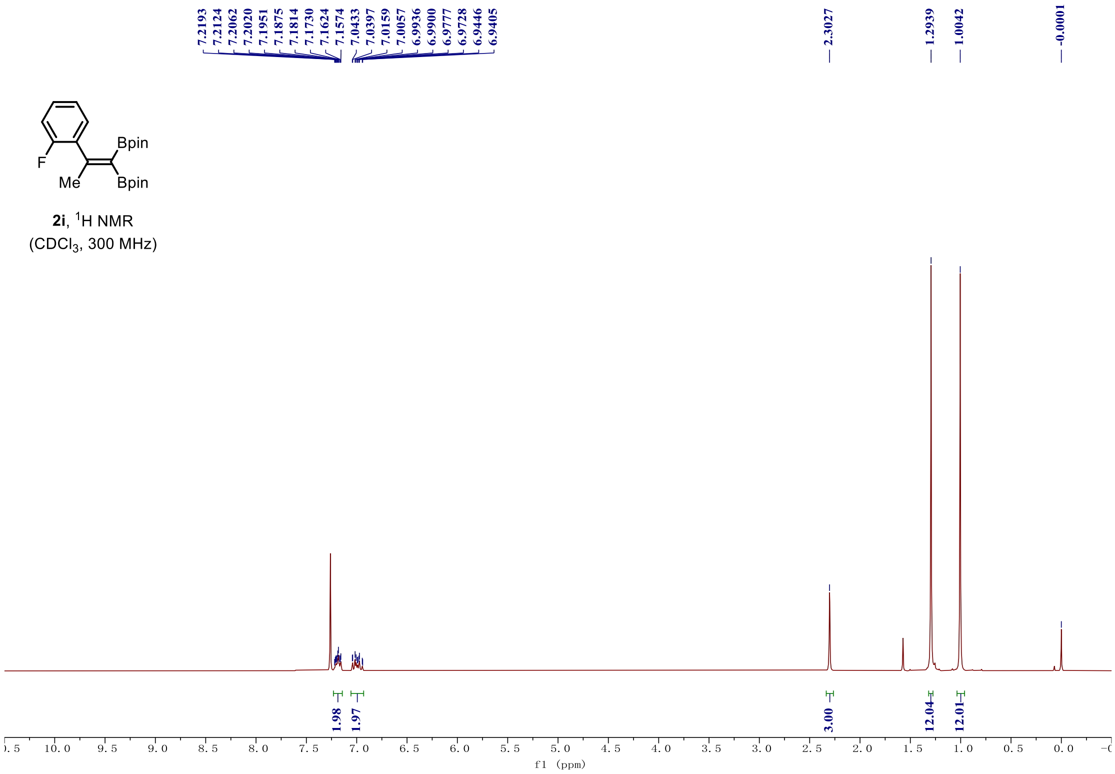

2i, ^{1}H NMR
($CDCl_3$, 300 MHz)

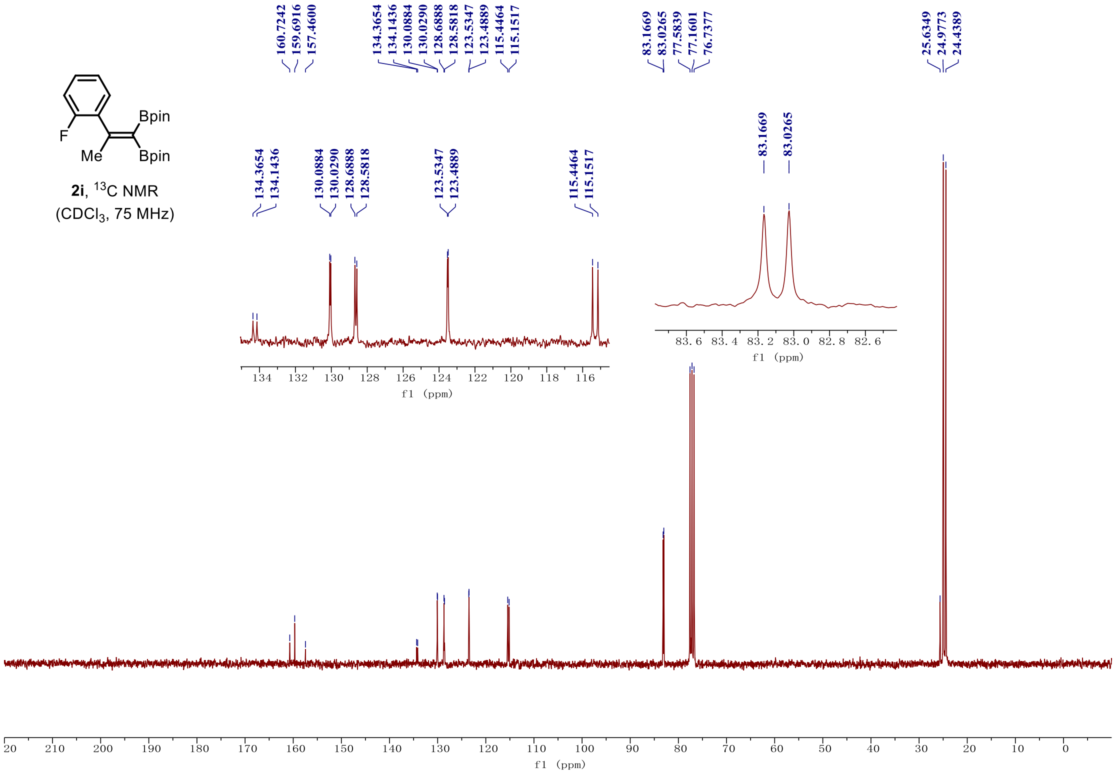

2i, ^{13}C NMR
($CDCl_3$, 75 MHz)

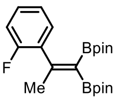

2i, ^{19}F NMR
($CDCl_3$, 282 MHz)

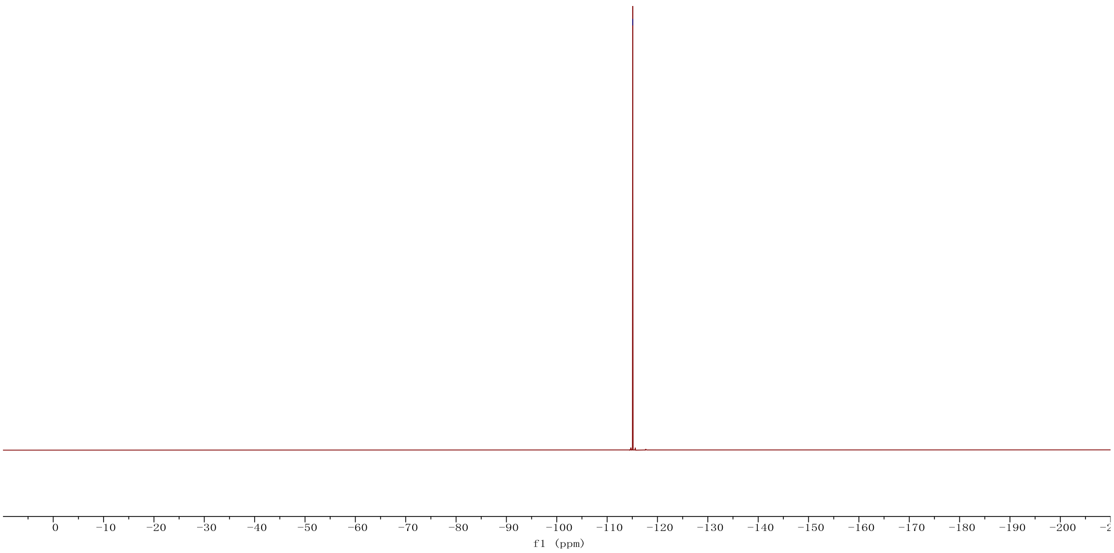

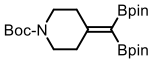

2k, ^{1}H NMR
($CDCl_3$, 300 MHz)

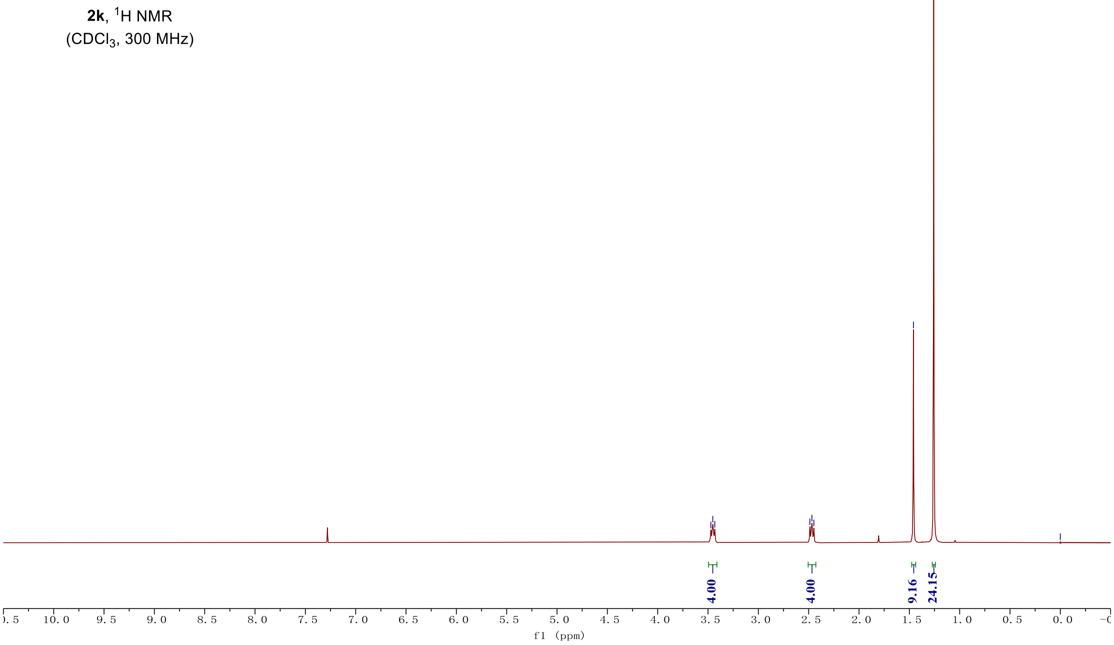

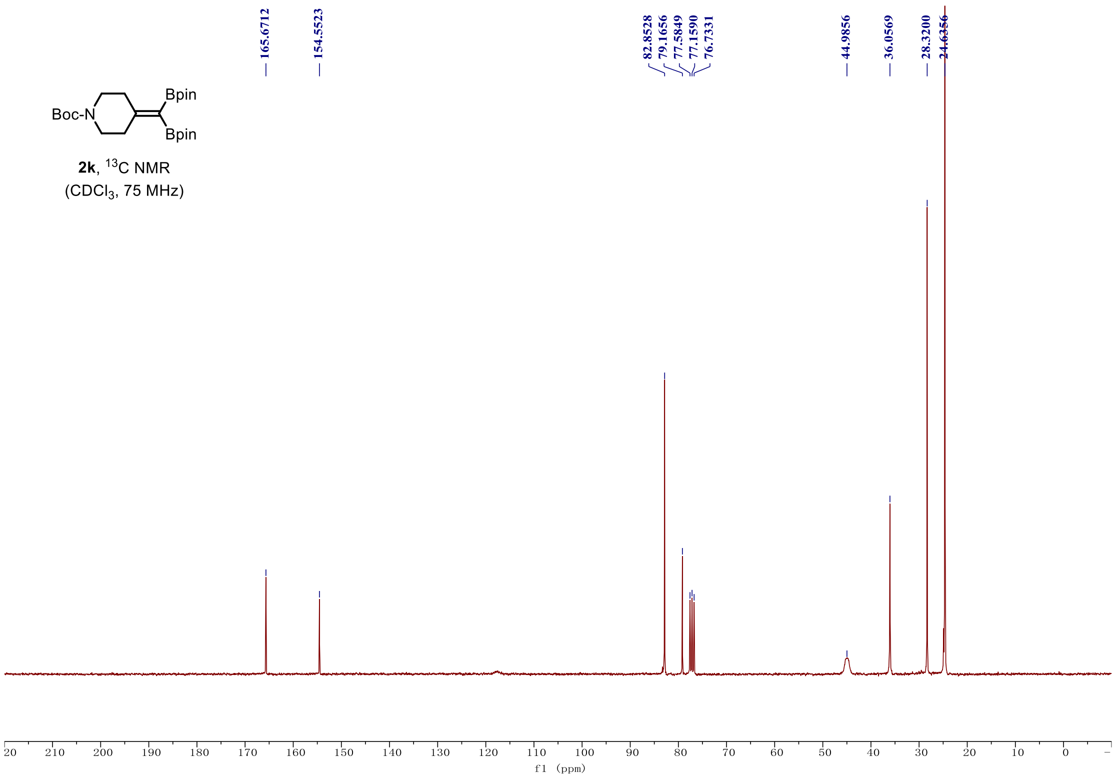
165.6712
154.5523
82.8528
79.1656
77.5849
77.1590
76.7331
44.9856
36.0569
28.3200
24.6356
Boc-N
Bpin
Bpin
2k, 13C NMR
(CDCl3, 75 MHz)
f1 (ppm)

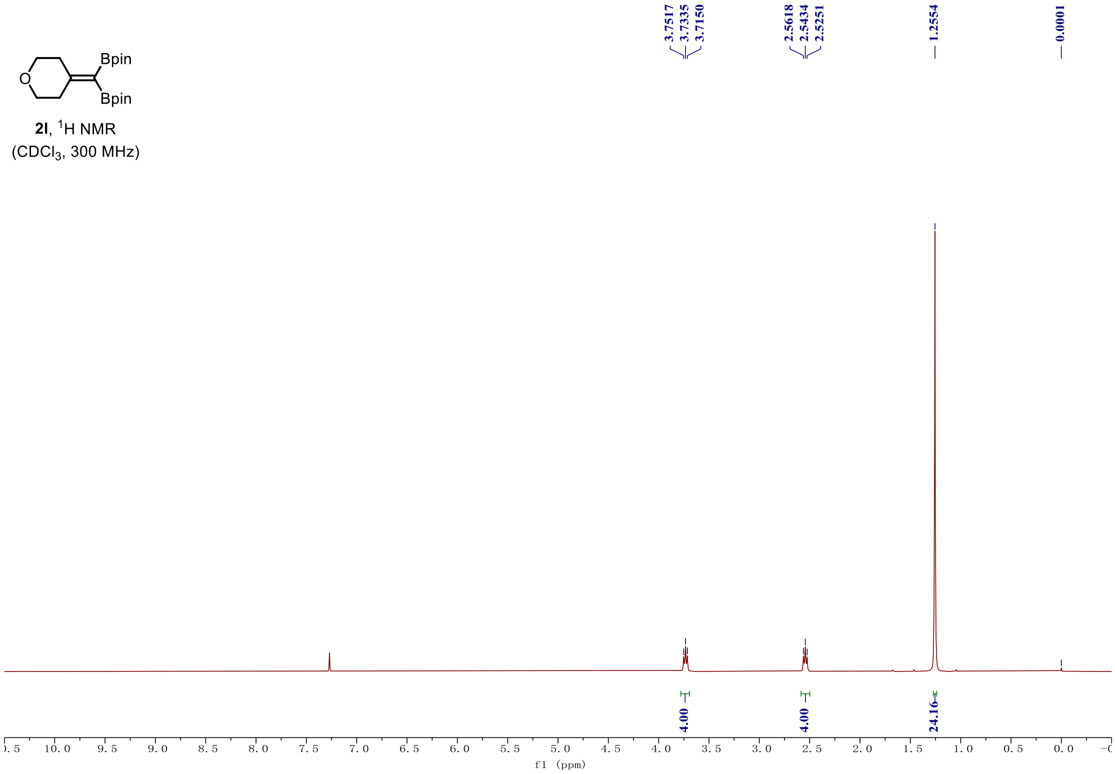
3.7517
3.7335
3.7150
2.5618
2.5434
2.5251
1.2554
0.0001
O
Bpin
Bpin
2l, 1H NMR
(CDCl3, 300 MHz)
4.00
4.00
24.16
f1 (ppm)

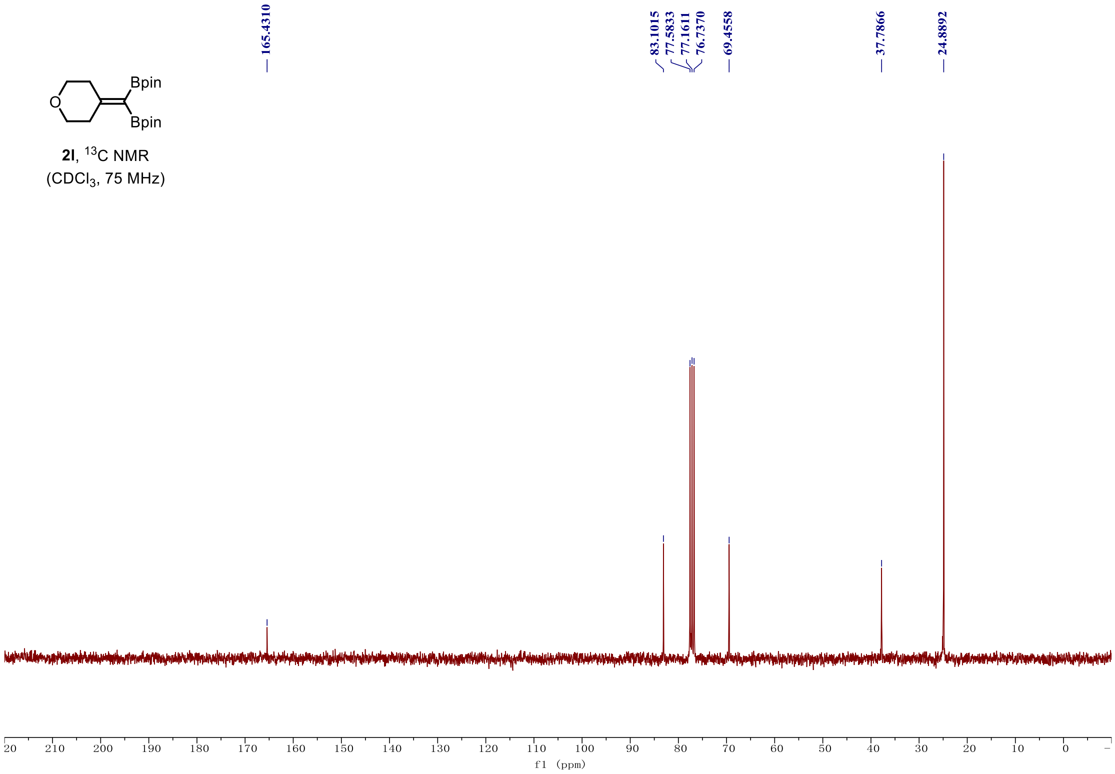

2l, ^{13}C NMR
($CDCl_3$, 75 MHz)

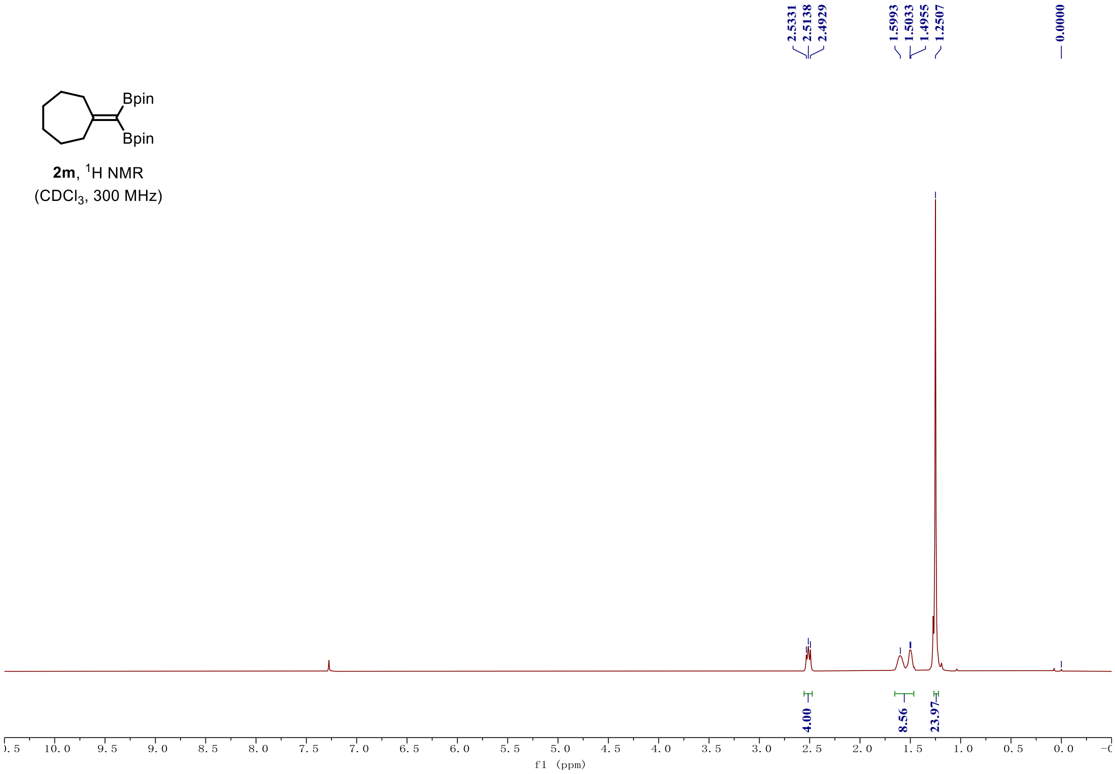

2m, ^{1}H NMR
($CDCl_3$, 300 MHz)

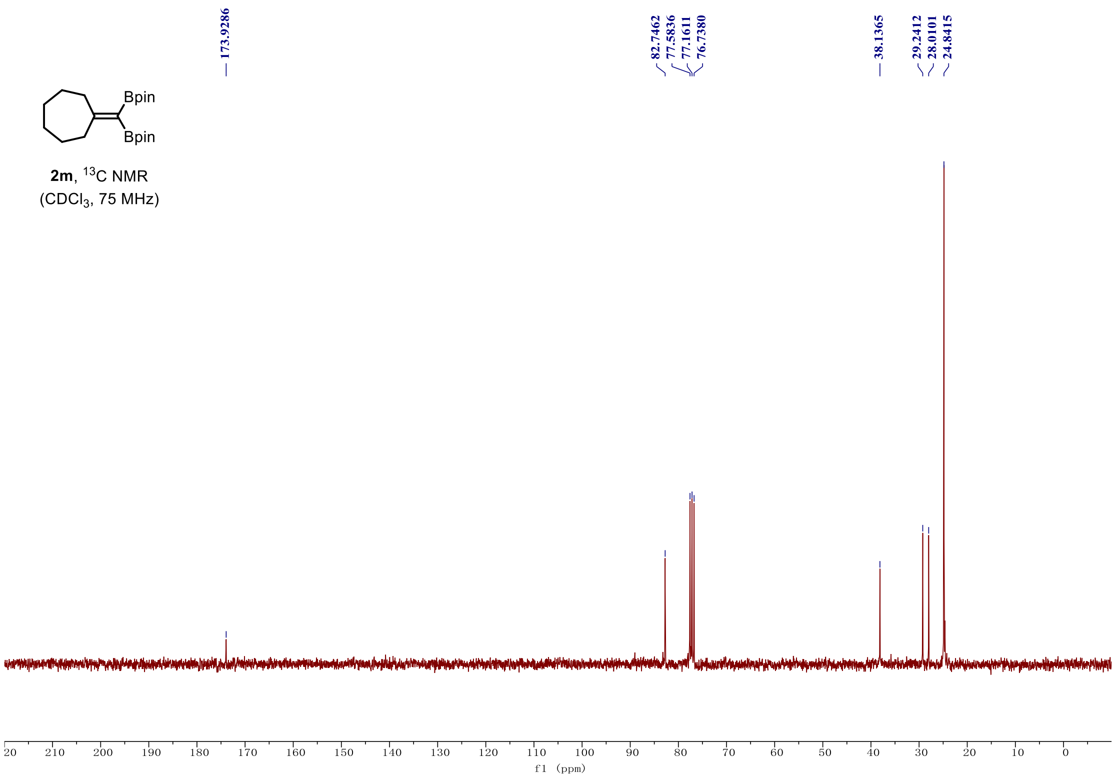
Bpin
Bpin
2m, 13C NMR
(CDCl3, 75 MHz)
173.9286
82.7462
77.5836
77.1611
76.7380
38.1365
29.2412
28.0101
24.8415
f1 (ppm)

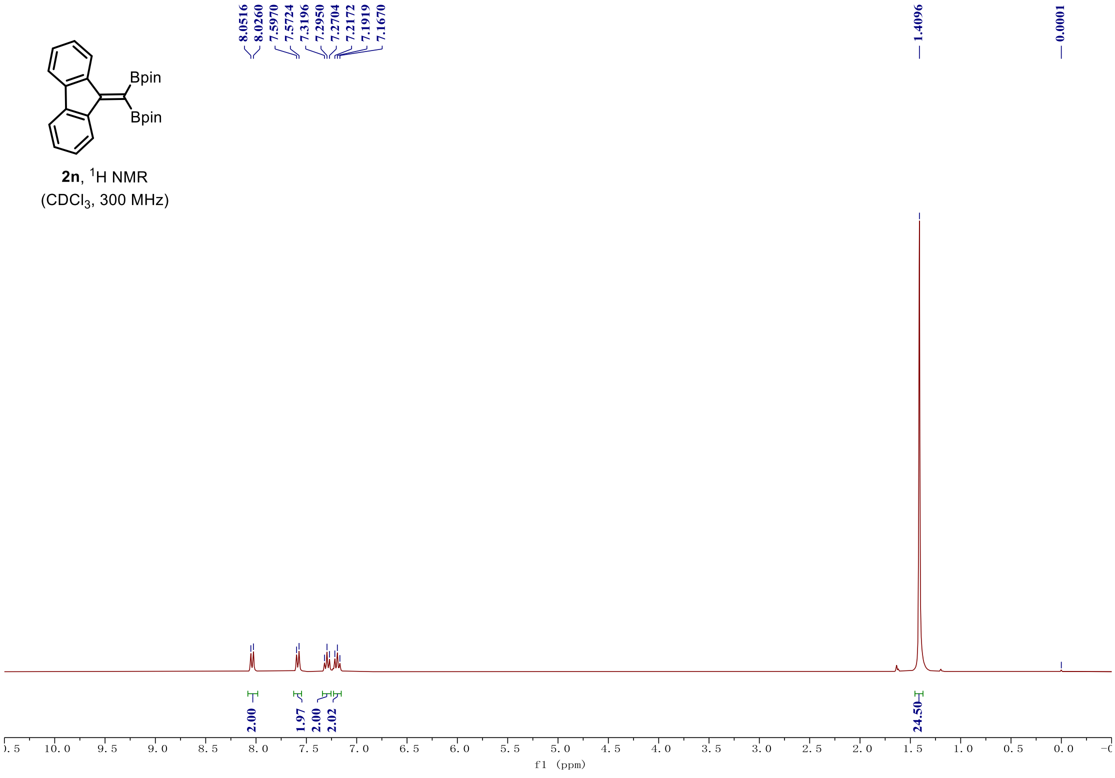
Bpin
Bpin
2n, 1H NMR
(CDCl3, 300 MHz)
8.0516
8.0260
7.5970
7.5724
7.3196
7.2950
7.2704
7.2172
7.1919
7.1670
1.4096
0.0001
2.00
1.97
2.00
2.02
24.50
f1 (ppm)

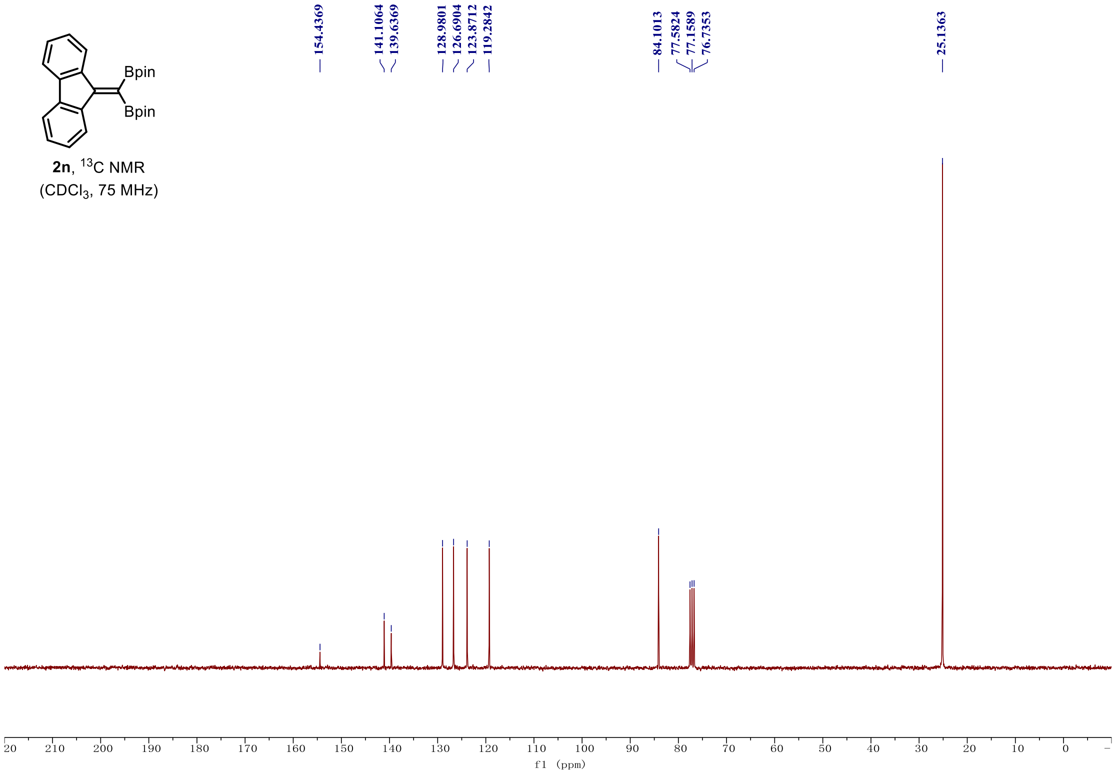

2n, ^{13}C NMR
($CDCl_3$, 75 MHz)

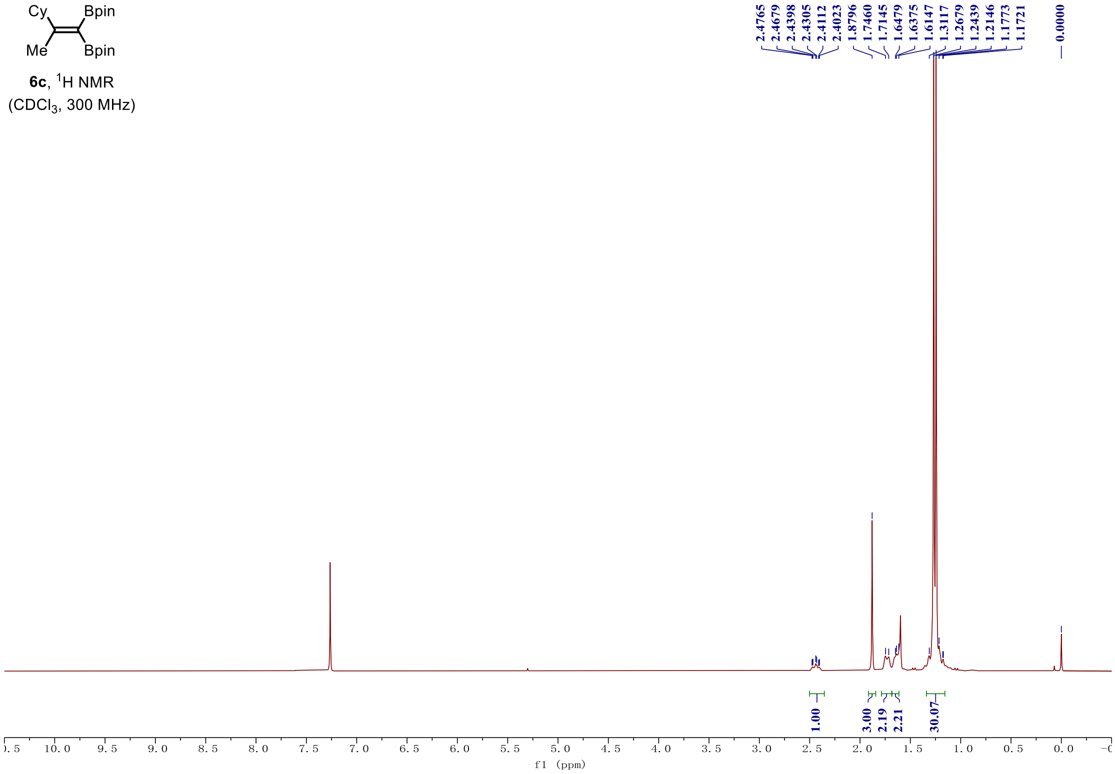

6c, ^{1}H NMR
($CDCl_3$, 300 MHz)

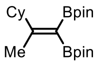

6c, ^{13}C NMR
($CDCl_3$, 75 MHz)

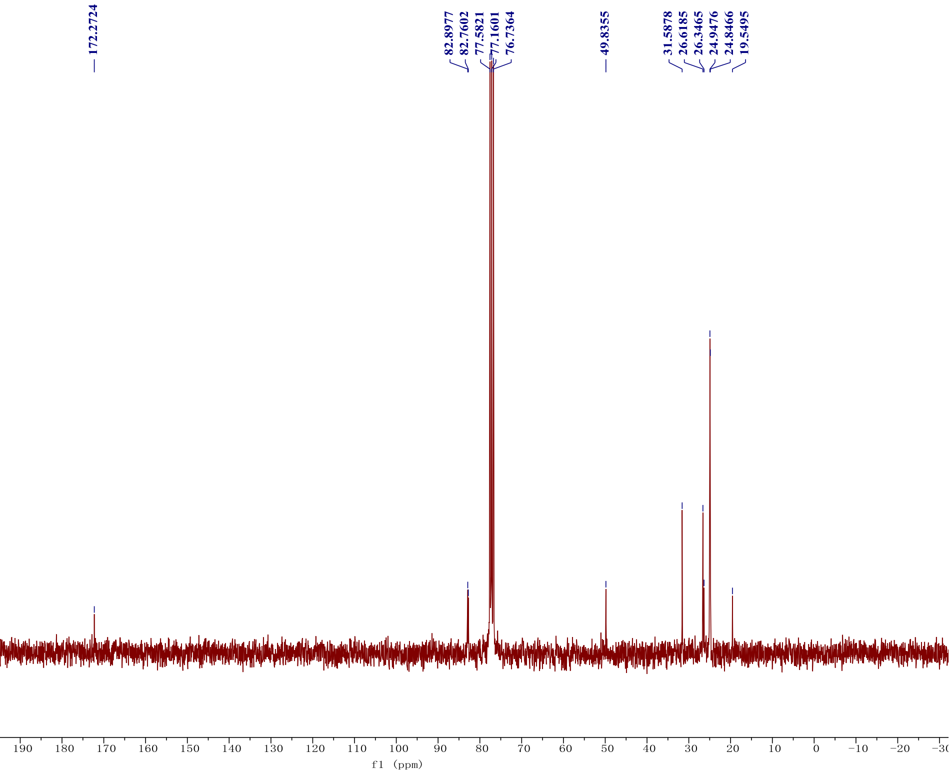

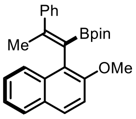

3a, ^{1}H NMR
($CDCl_3$, 300 MHz)

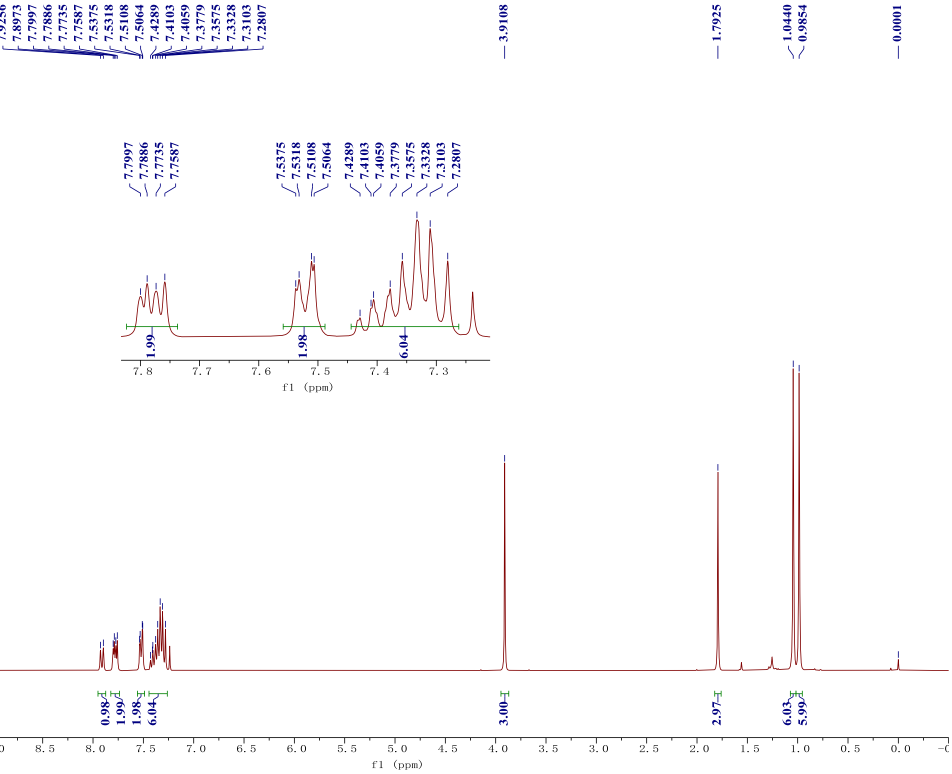

3a, ^{13}C NMR
($CDCl_3$, 75 MHz)

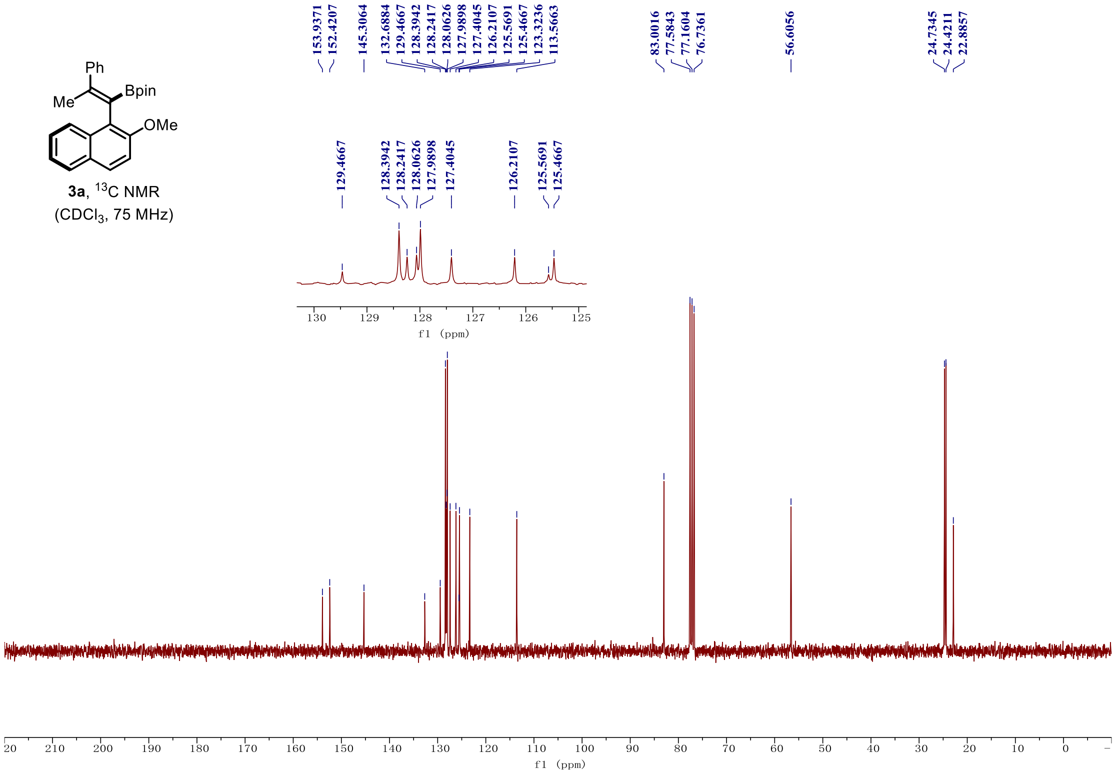

3b, ^{1}H NMR
($CDCl_3$, 300 MHz)

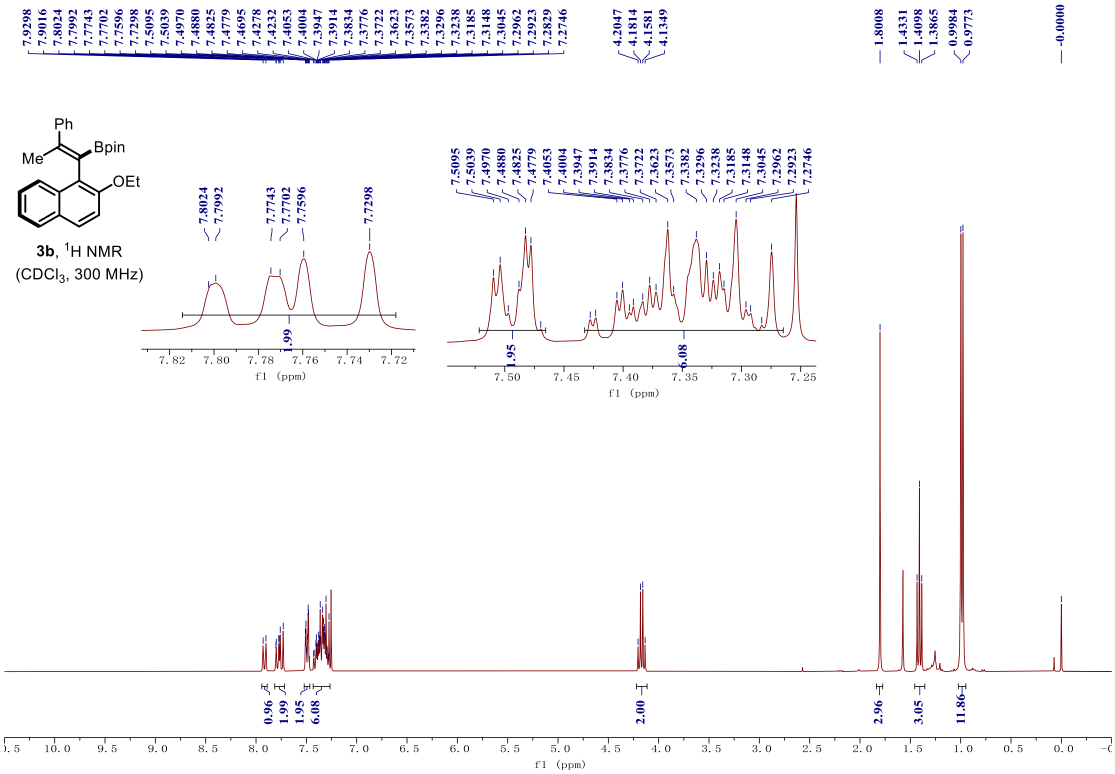

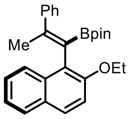

3b, ^{13}C NMR
($CDCl_3$, 75 MHz)

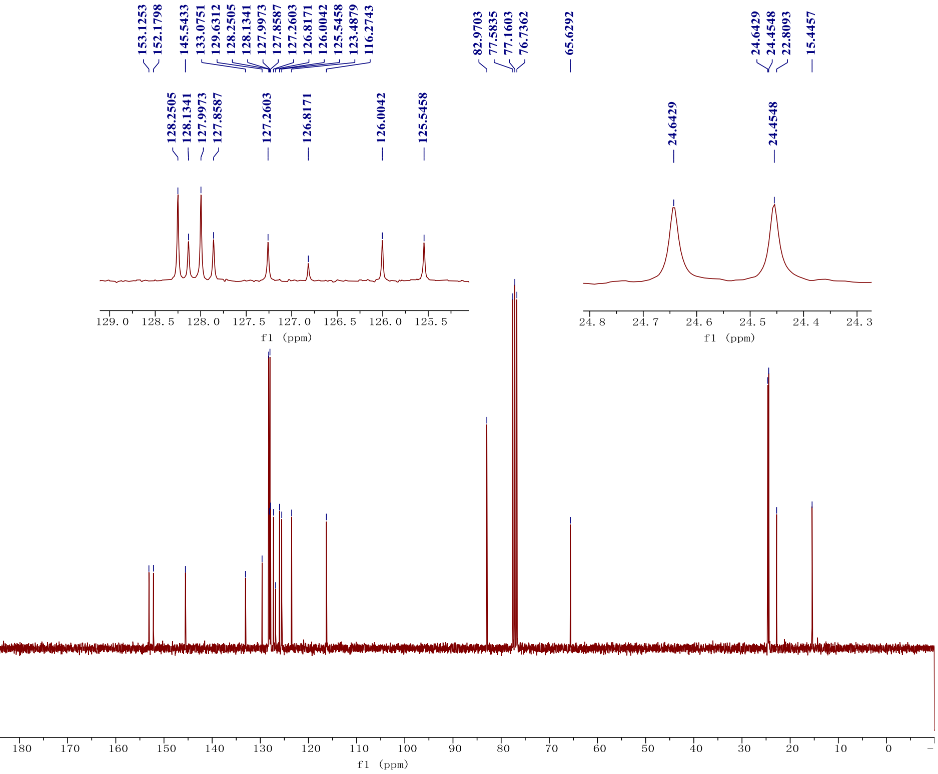

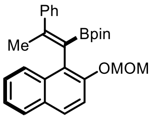

3c, ^1H NMR
($CDCl_3$, 300 MHz)

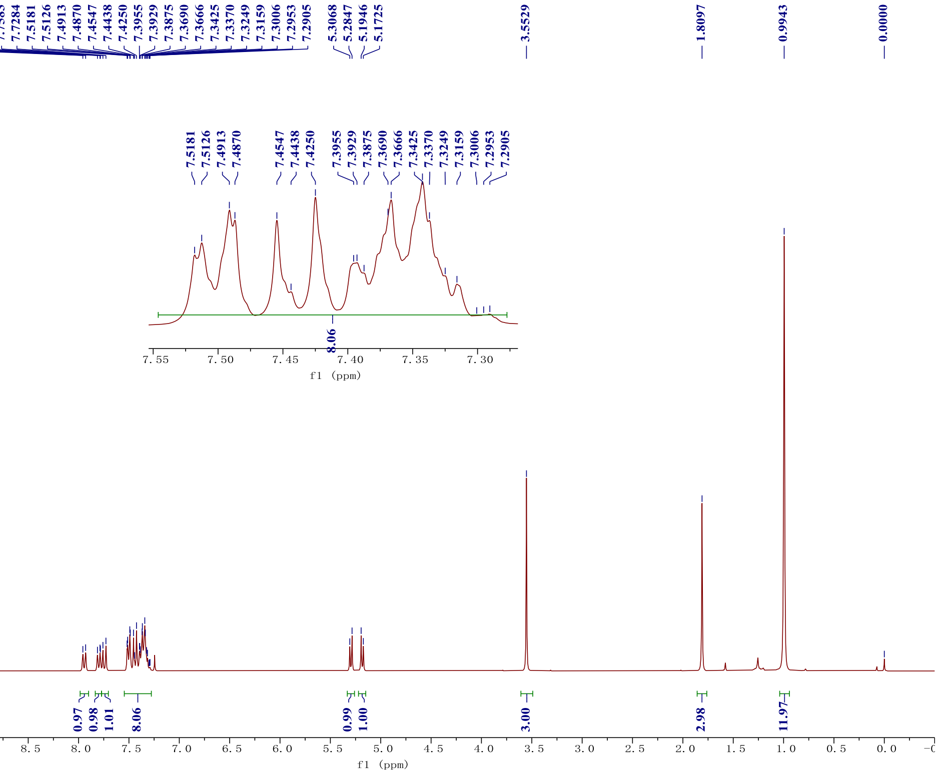

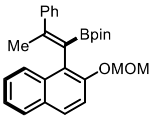

3c, ^{13}C NMR
($CDCl_3$, 75 MHz)

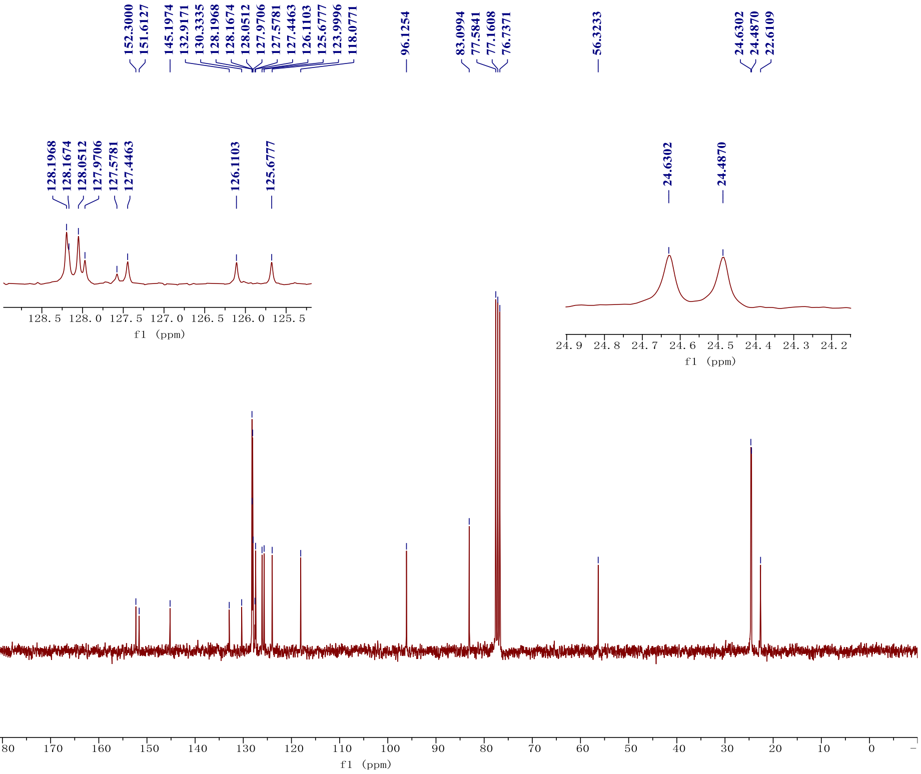

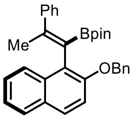

3d, ^{1}H NMR
($CDCl_3$, 300 MHz)

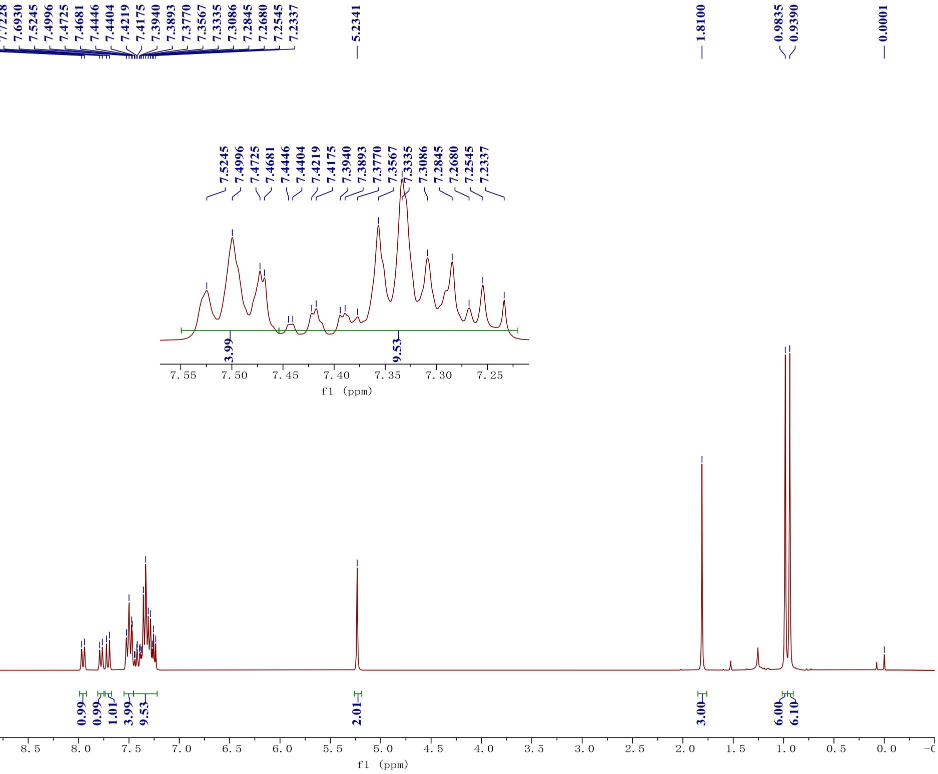

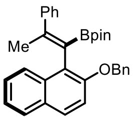

3d, ^{13}C NMR
($CDCl_3$, 75 MHz)

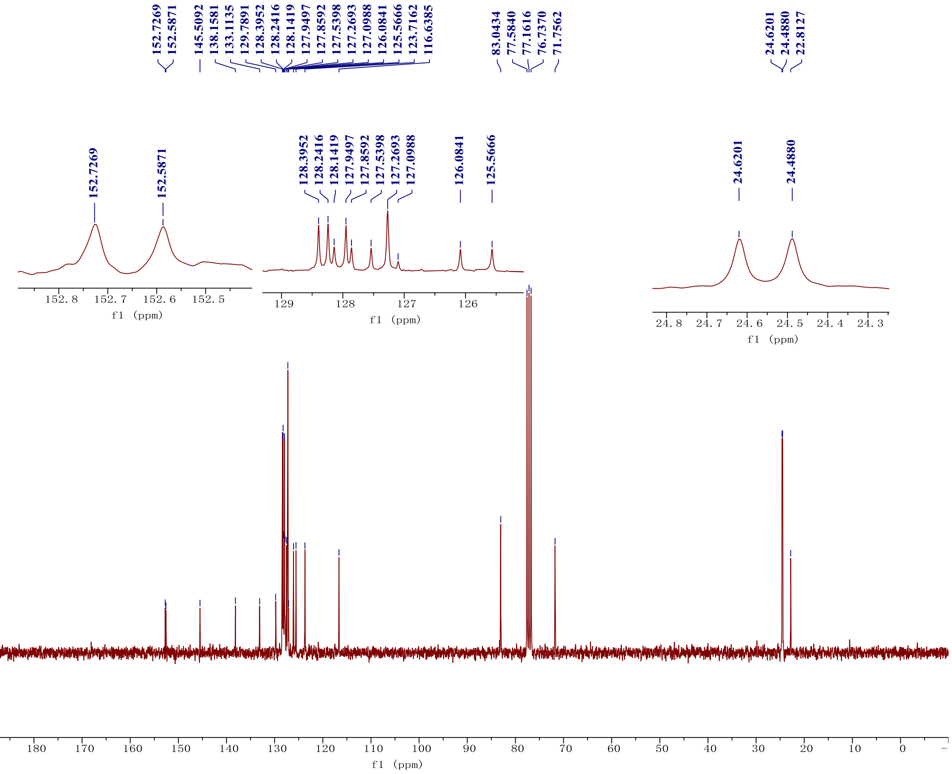

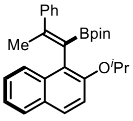

3e, ^{1}H NMR
($CDCl_3$, 300 MHz)

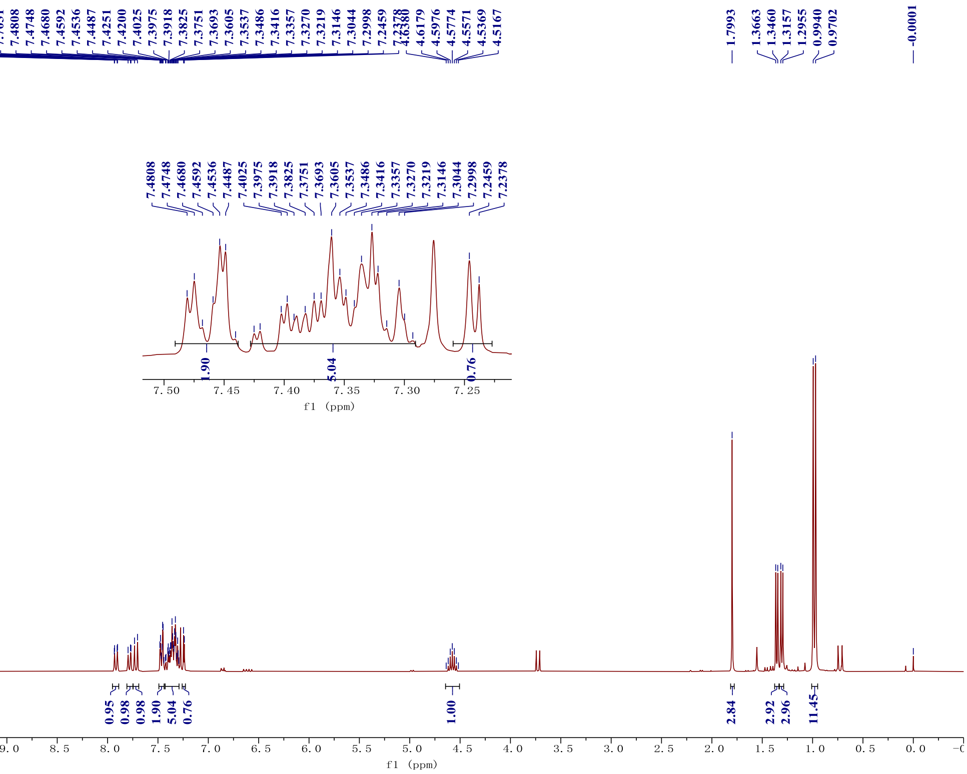

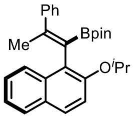

3e, ^{13}C NMR
($CDCl_3$, 75 MHz)

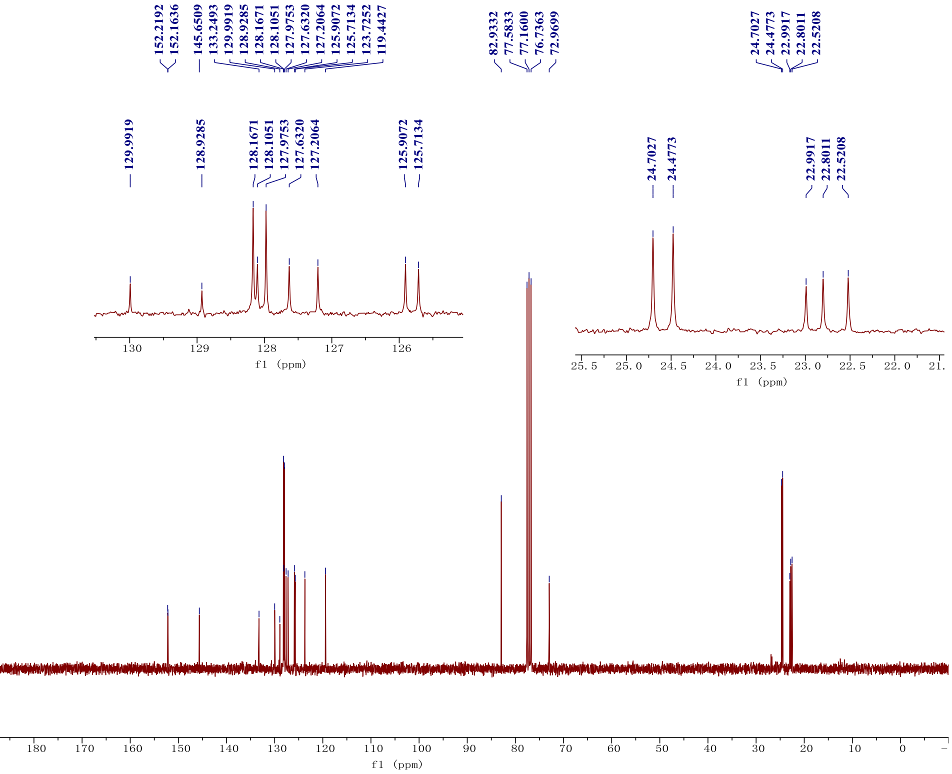

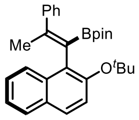

3f, ^{1}H NMR
($CDCl_3$, 300 MHz)

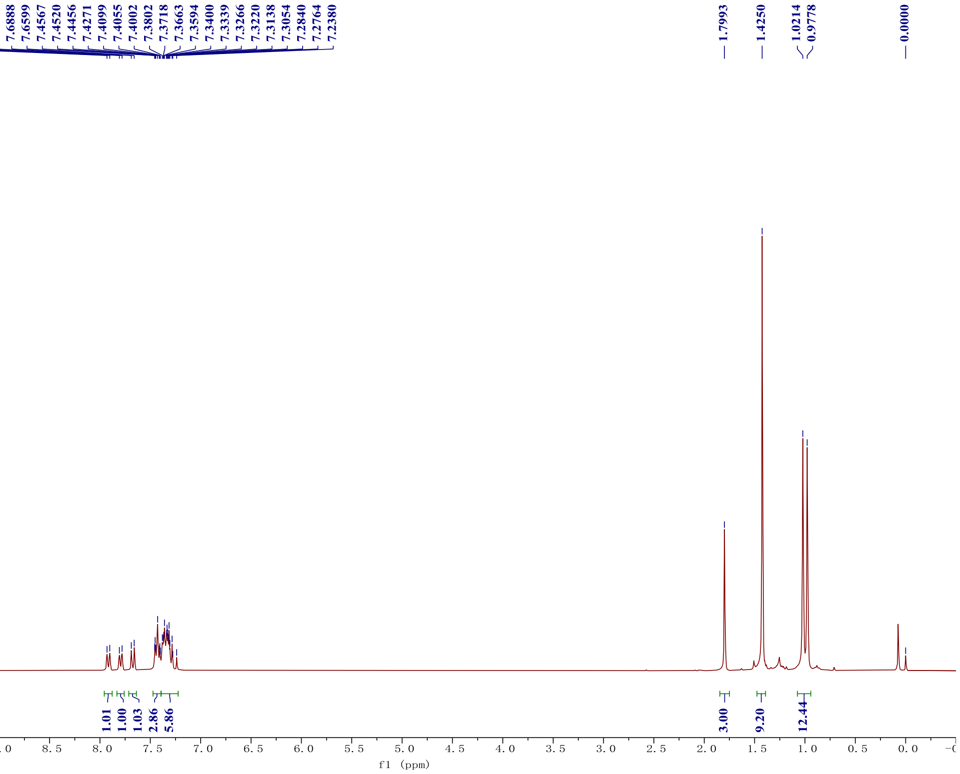

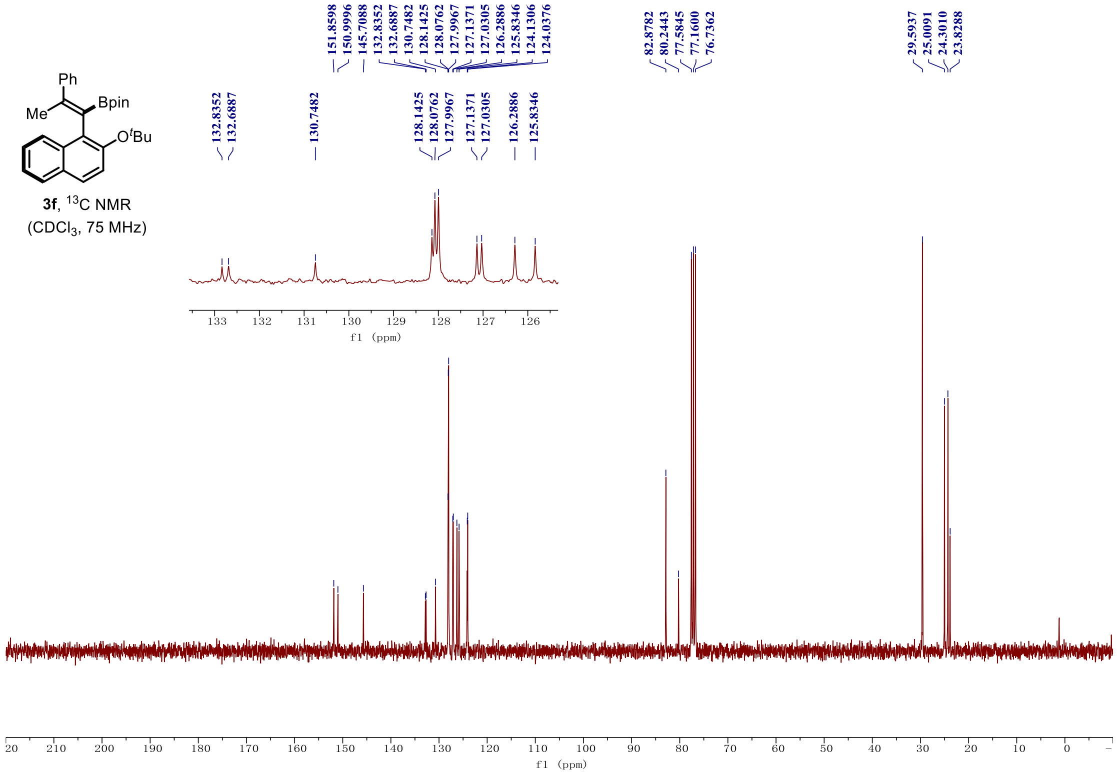
3f, ^{13}C NMR
(CDCl$_3$, 75 MHz)
151.8598
150.9996
145.7088
132.8352
132.6887
130.7482
128.1425
128.0762
127.9967
127.1371
127.0305
126.2886
125.8346
124.1306
124.0376
82.8782
80.2443
77.5845
77.1600
76.7362
29.5937
25.0091
24.3010
23.8288
f1 (ppm)

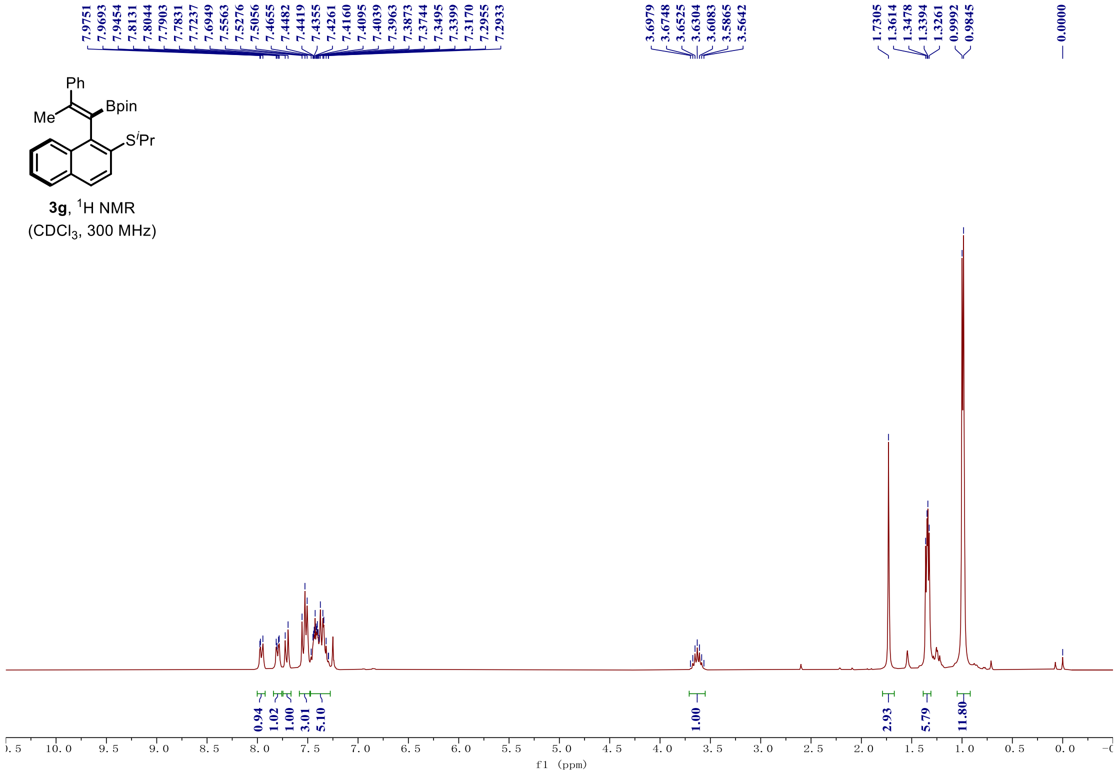
3g, ^{1}H NMR
(CDCl$_3$, 300 MHz)
7.9751
7.9693
7.9454
7.8131
7.8044
7.7903
7.7831
7.7237
7.6949
7.5563
7.5276
7.5056
7.4655
7.4482
7.4419
7.4355
7.4261
7.4160
7.4095
7.4039
7.3963
7.3873
7.3744
7.3495
7.3399
7.3170
7.2955
7.2933
3.6979
3.6748
3.6525
3.6304
3.6083
3.5865
3.5642
1.7305
1.3614
1.3478
1.3394
1.3261
0.9992
0.9845
0.0000
0.94
1.02
1.00
3.01
5.10
1.00
2.93
5.79
11.80
f1 (ppm)

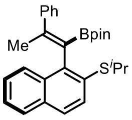

3g, ^{13}C NMR
(CDCl$_3$, 75 MHz)

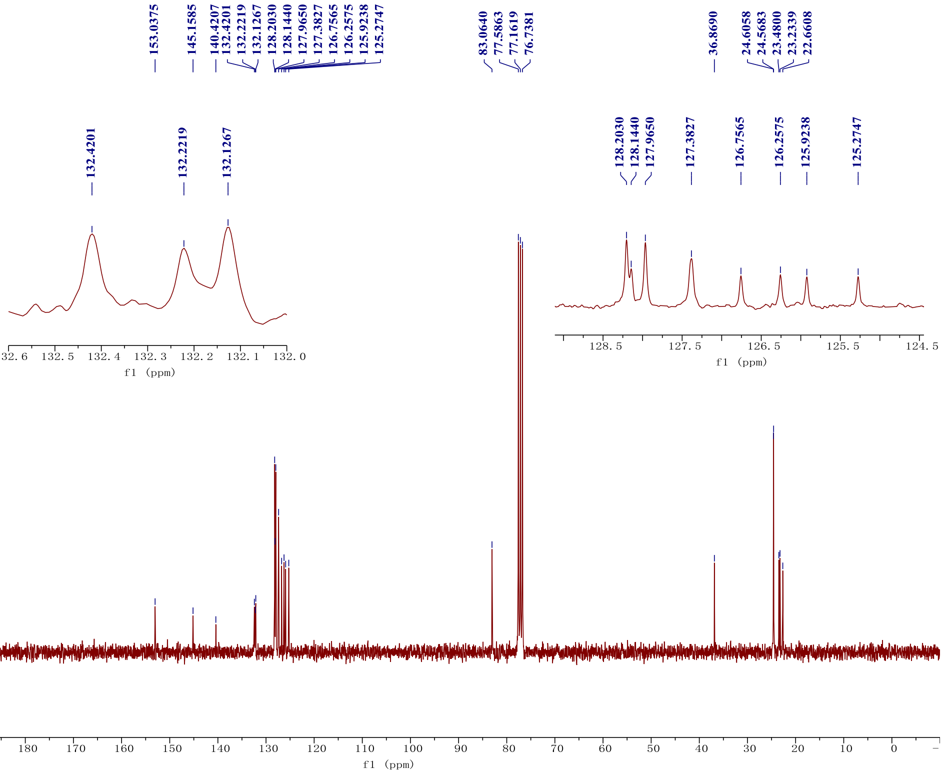

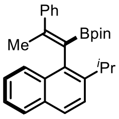

3h, ^{1}H NMR
(CDCl$_3$, 300 MHz)

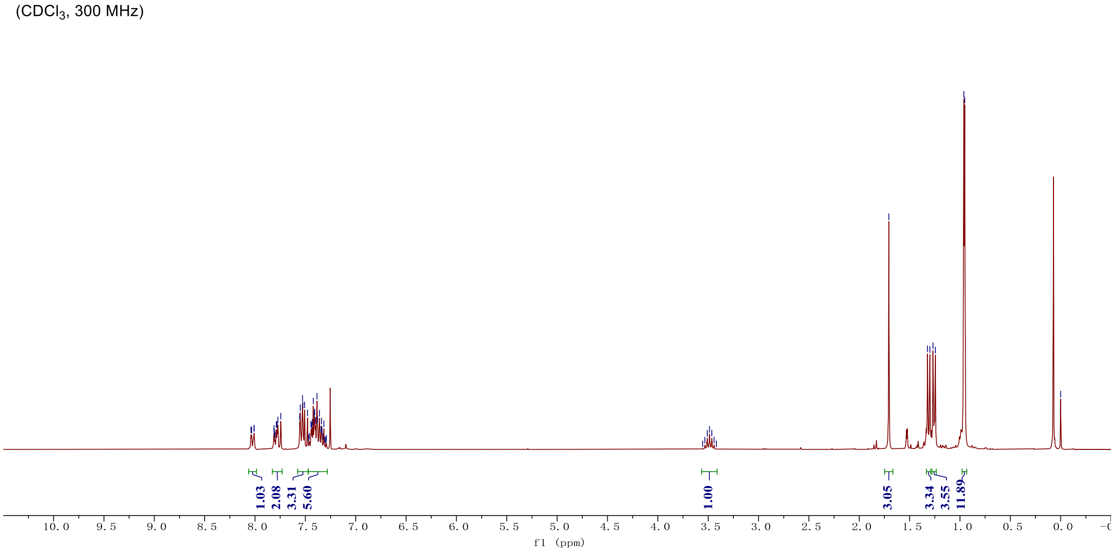

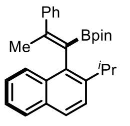

3h, ^{13}C NMR
($CDCl_3$, 75 MHz)

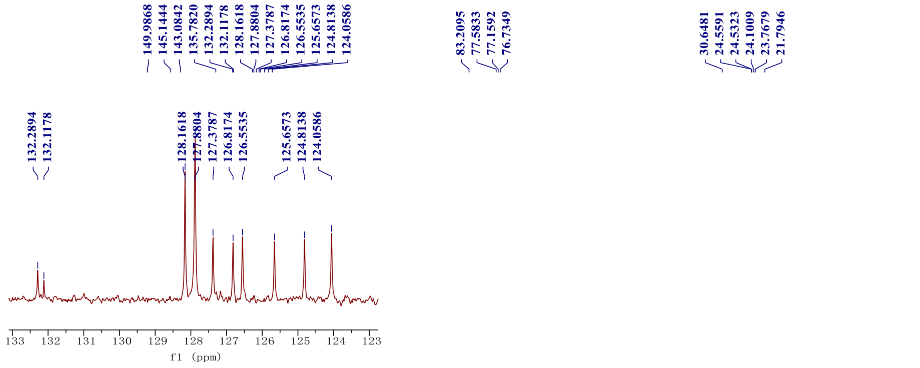

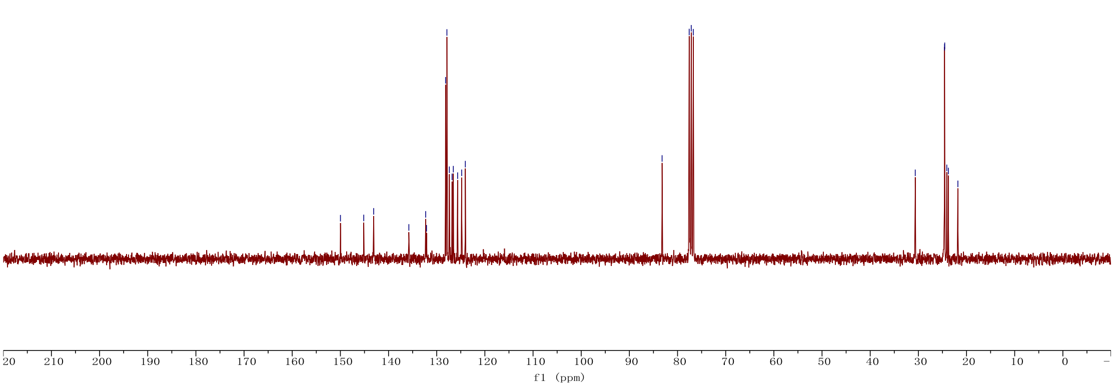

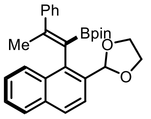

3i, ^{1}H NMR
($CDCl_3$, 300 MHz)

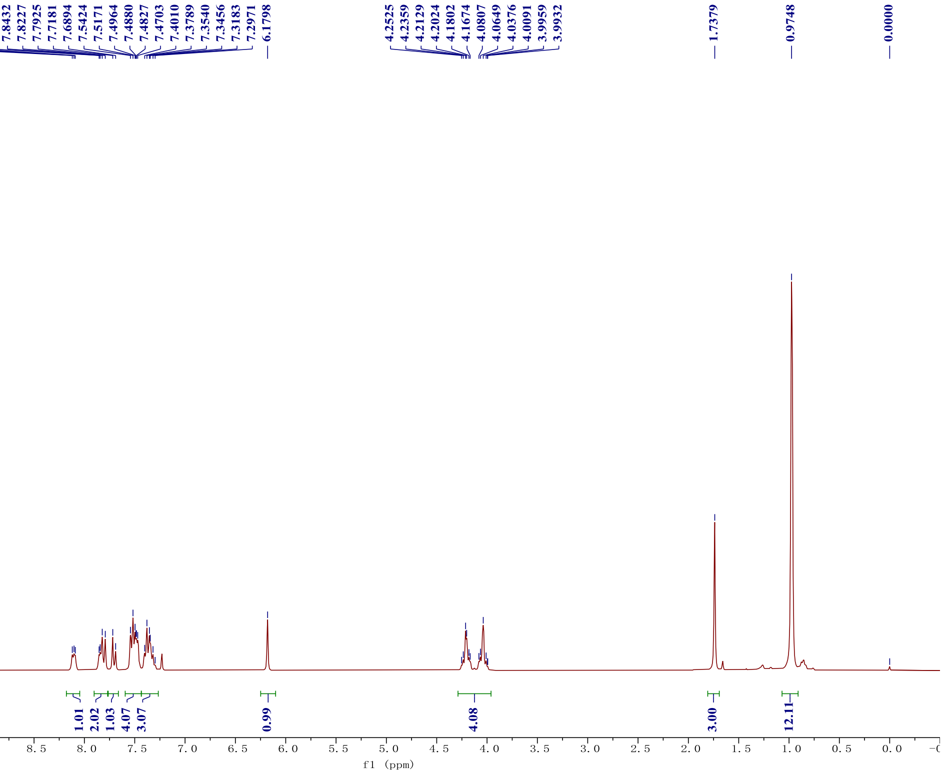

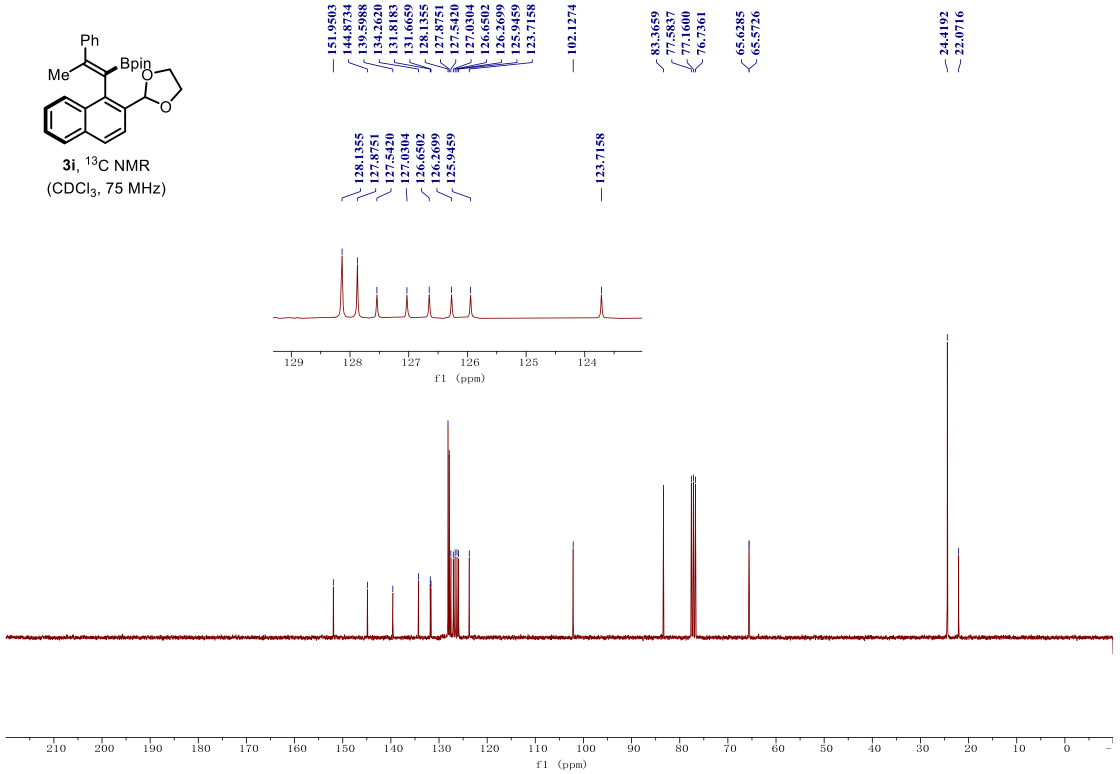

3i, ^{13}C NMR
($CDCl_3$, 75 MHz)

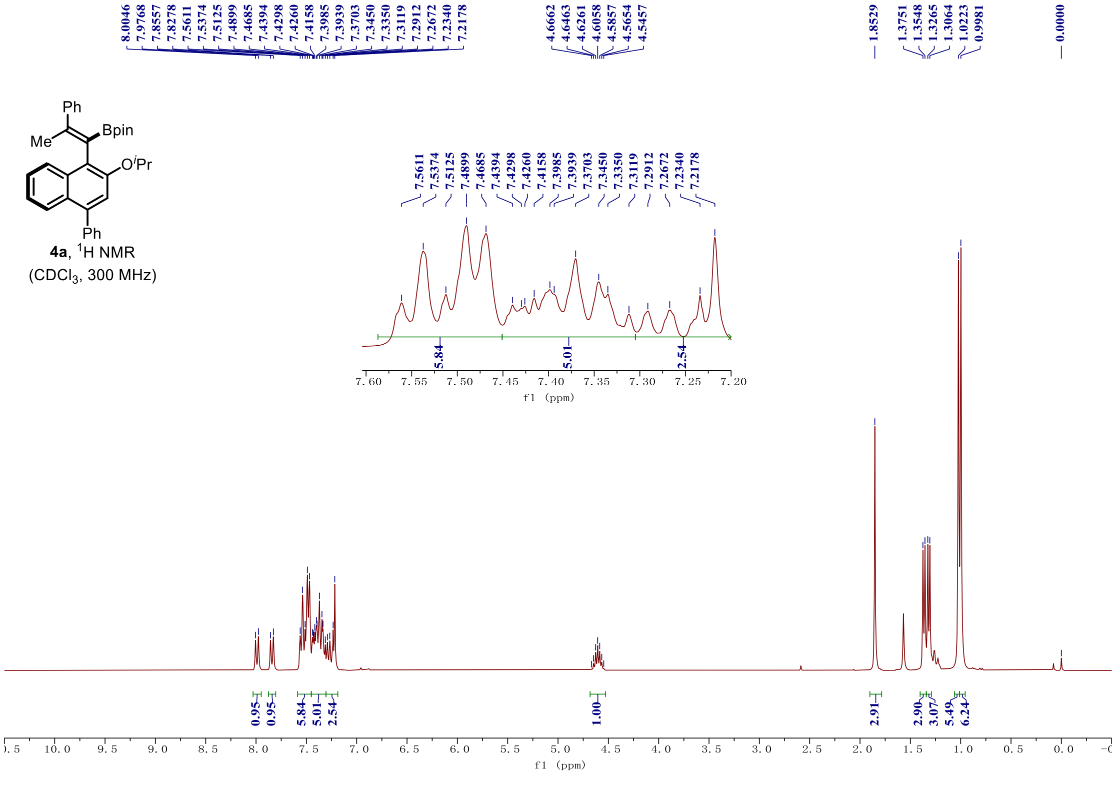

4a, ^{1}H NMR
($CDCl_3$, 300 MHz)

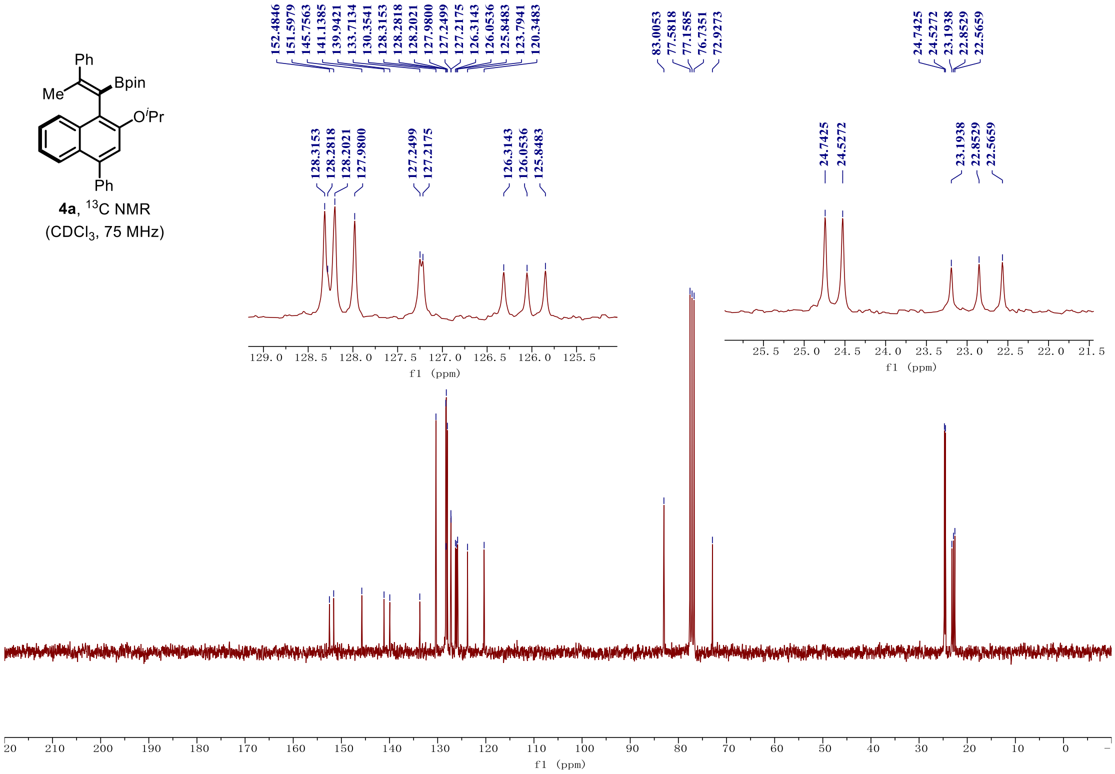

4a, ^{13}C NMR
($CDCl_3$, 75 MHz)

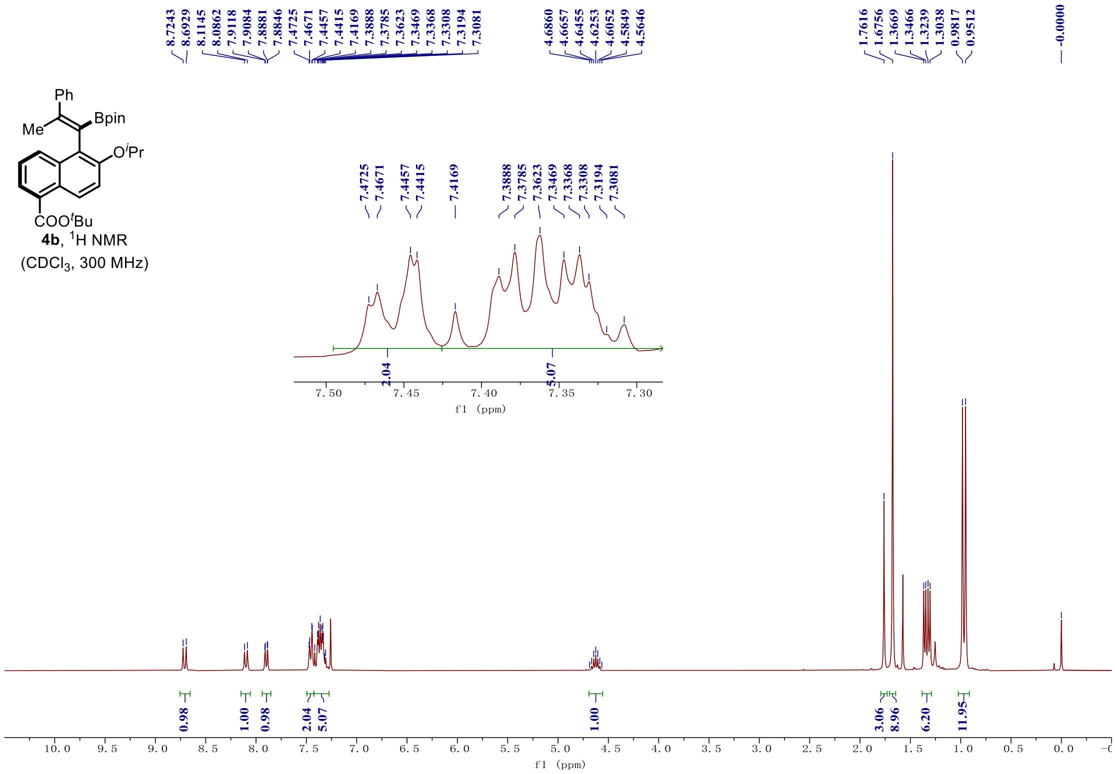

4b, ^{1}H NMR
($CDCl_3$, 300 MHz)

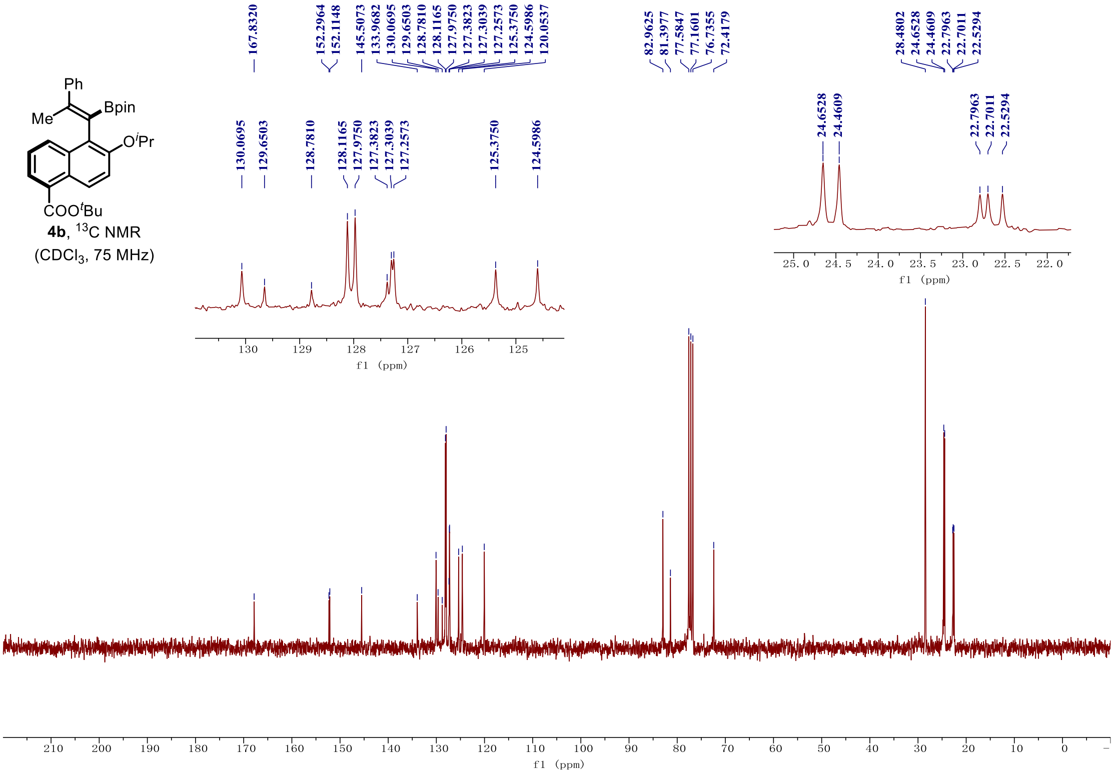

4b, ^{13}C NMR
(CDCl$_3$, 75 MHz)

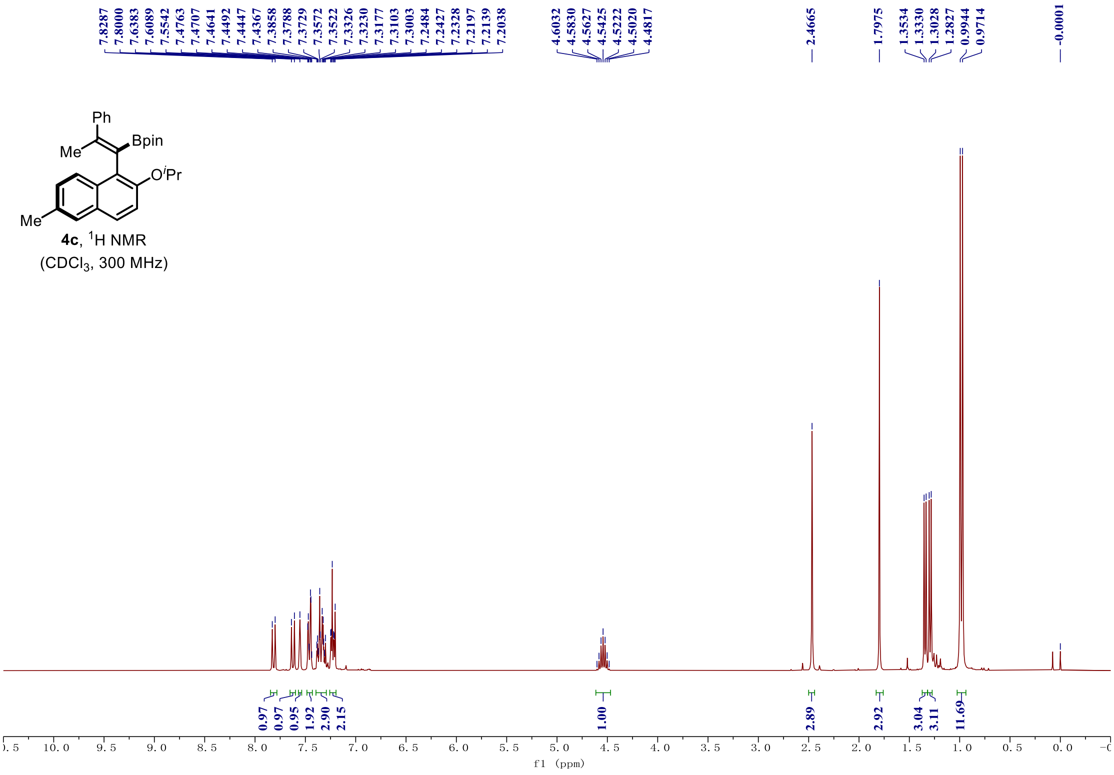

4c, ^{1}H NMR
(CDCl$_3$, 300 MHz)

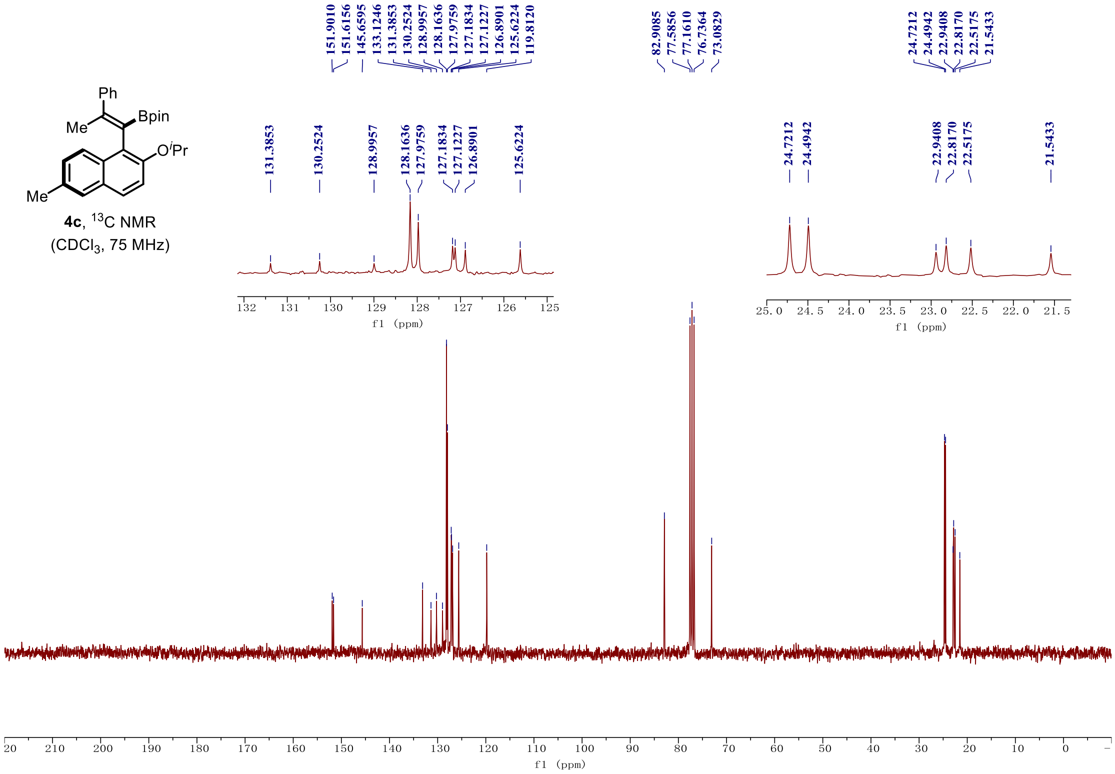

4c, ^{13}C NMR ($CDCl_3$, 75 MHz)

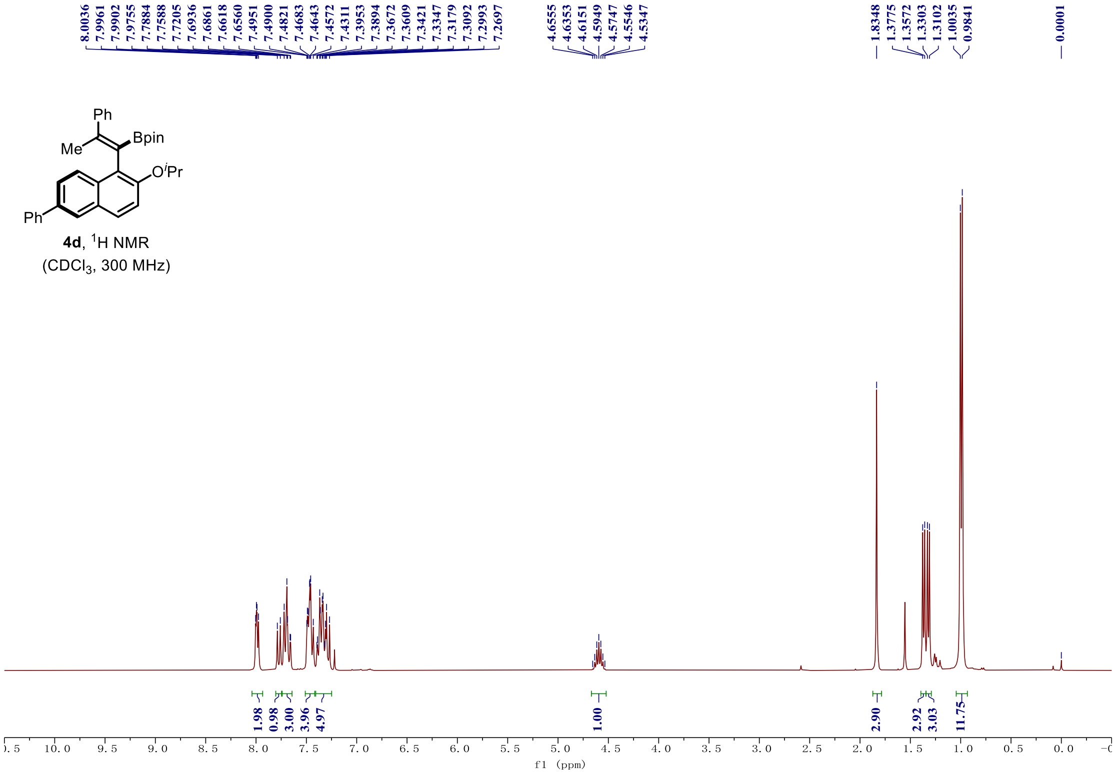

4d, ^{1}H NMR ($CDCl_3$, 300 MHz)

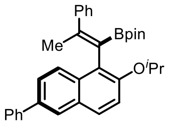

4d, ^{13}C NMR
($CDCl_3$, 75 MHz)

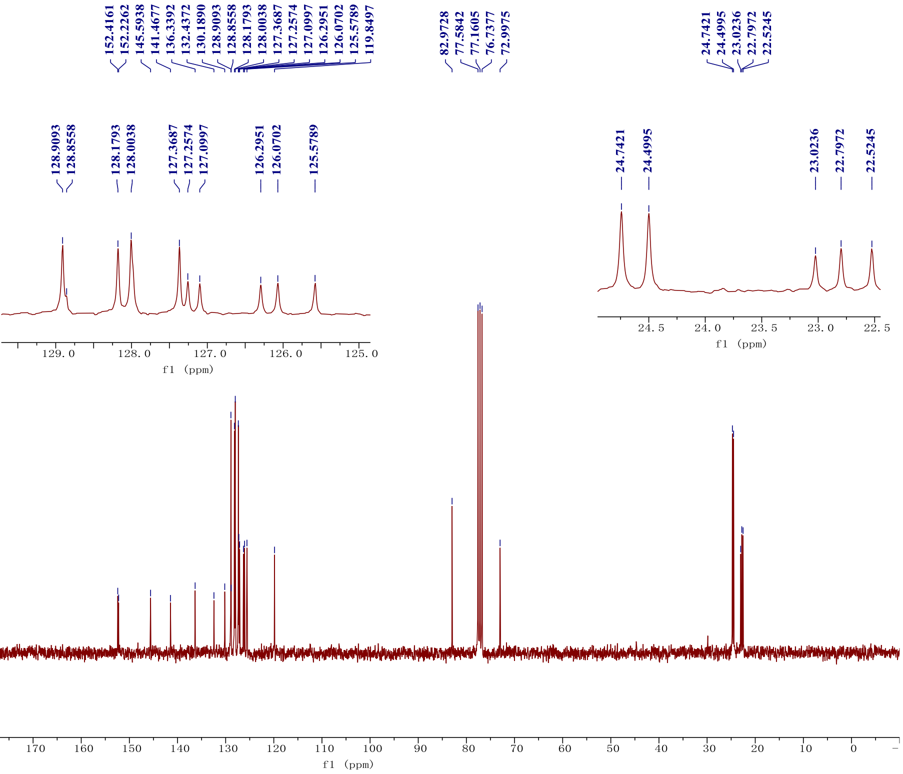

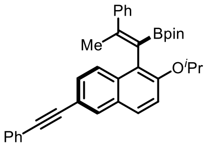

4e, ^1H NMR
($CDCl_3$, 300 MHz)

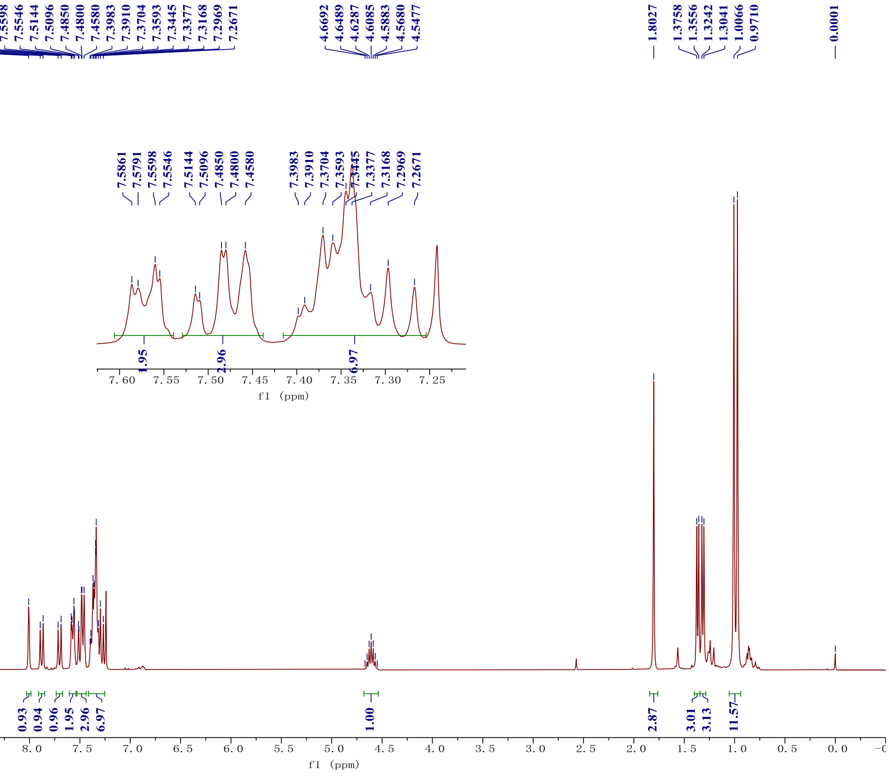

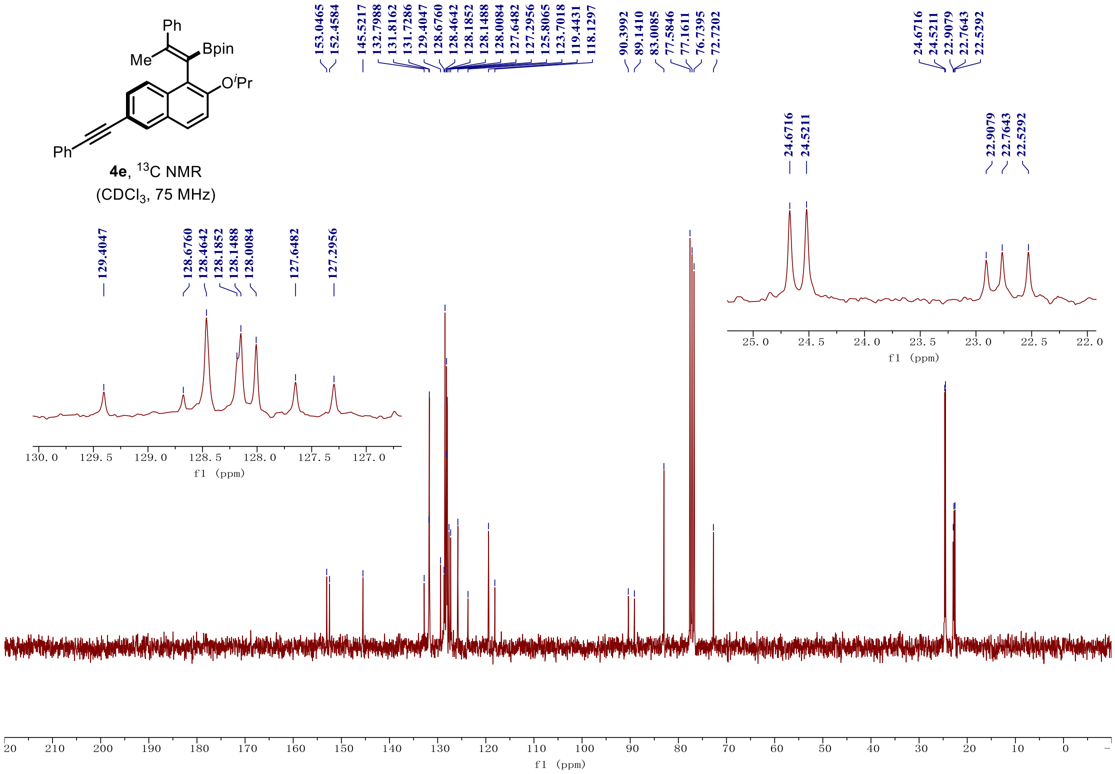

4e, ^{13}C NMR
(CDCl$_3$, 75 MHz)

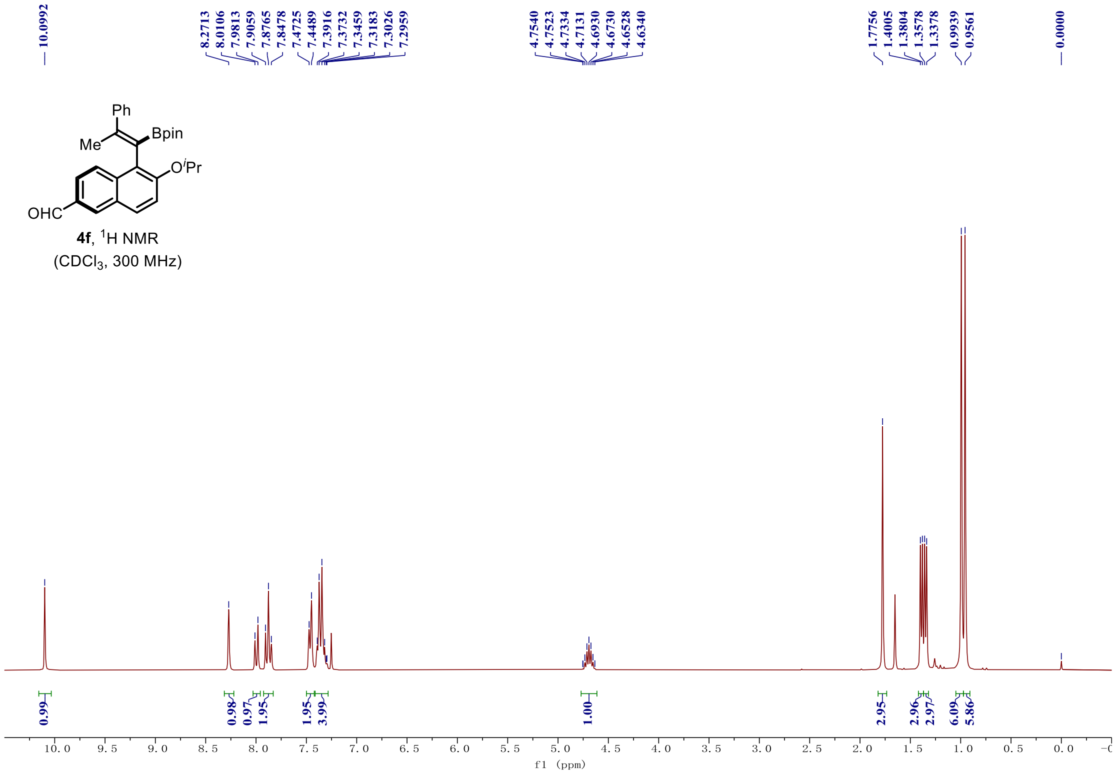

4f, ^{1}H NMR
(CDCl$_3$, 300 MHz)

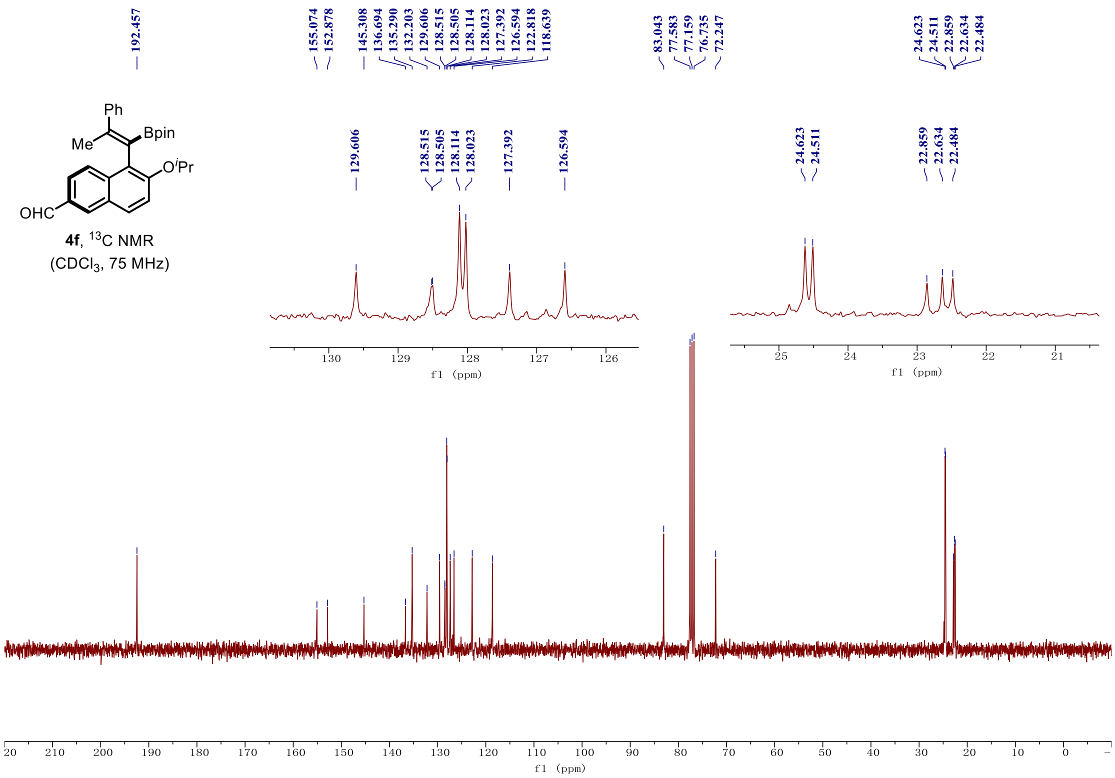

4f, ^{13}C NMR
($CDCl_3$, 75 MHz)

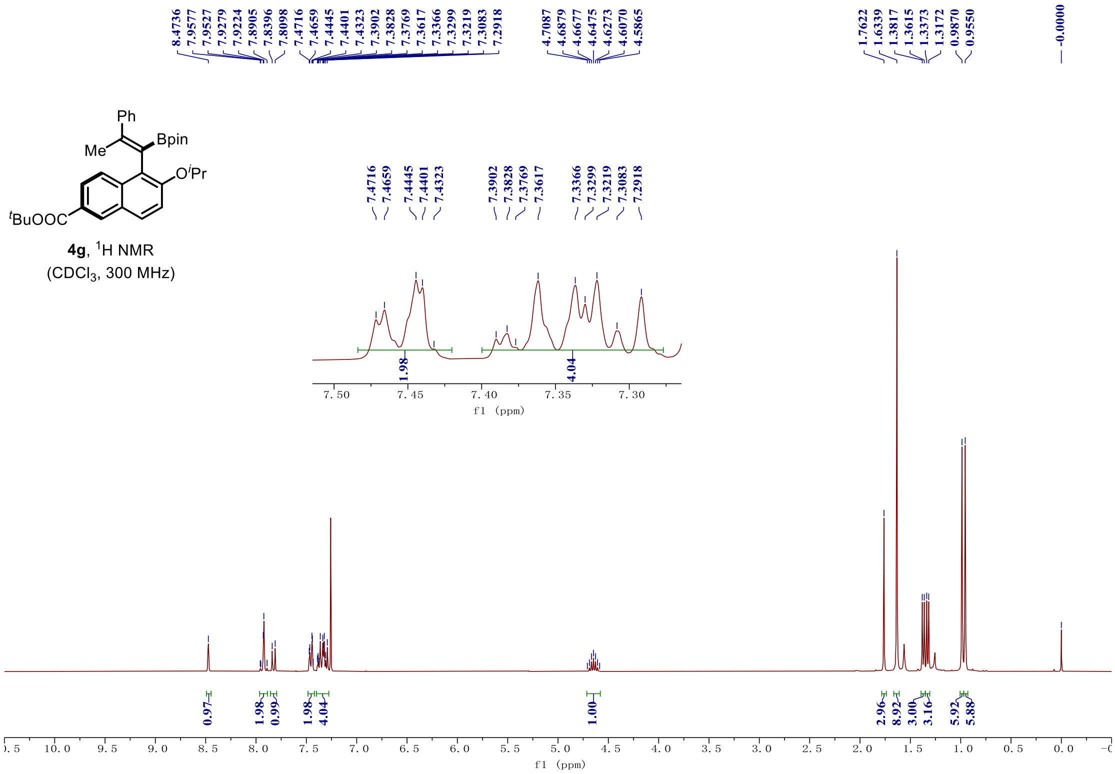

4g, ^{1}H NMR
($CDCl_3$, 300 MHz)

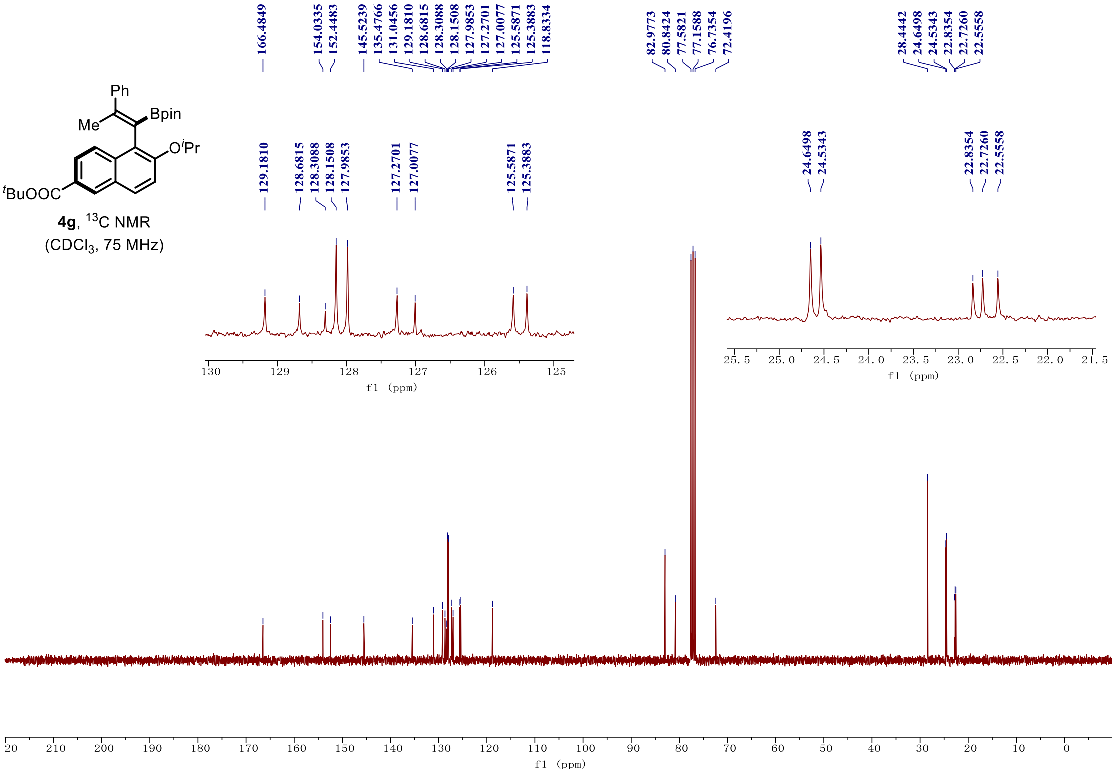

4g, ^{13}C NMR (CDCl$_3$, 75 MHz)

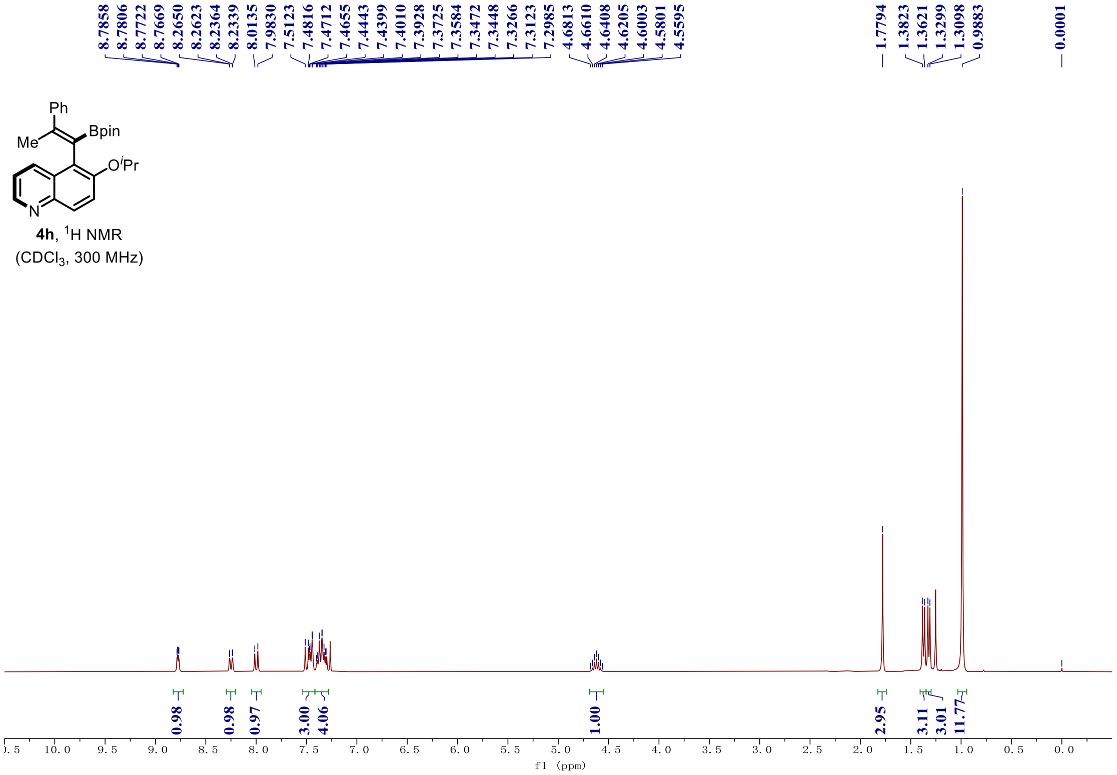

4h, ^{1}H NMR (CDCl$_3$, 300 MHz)

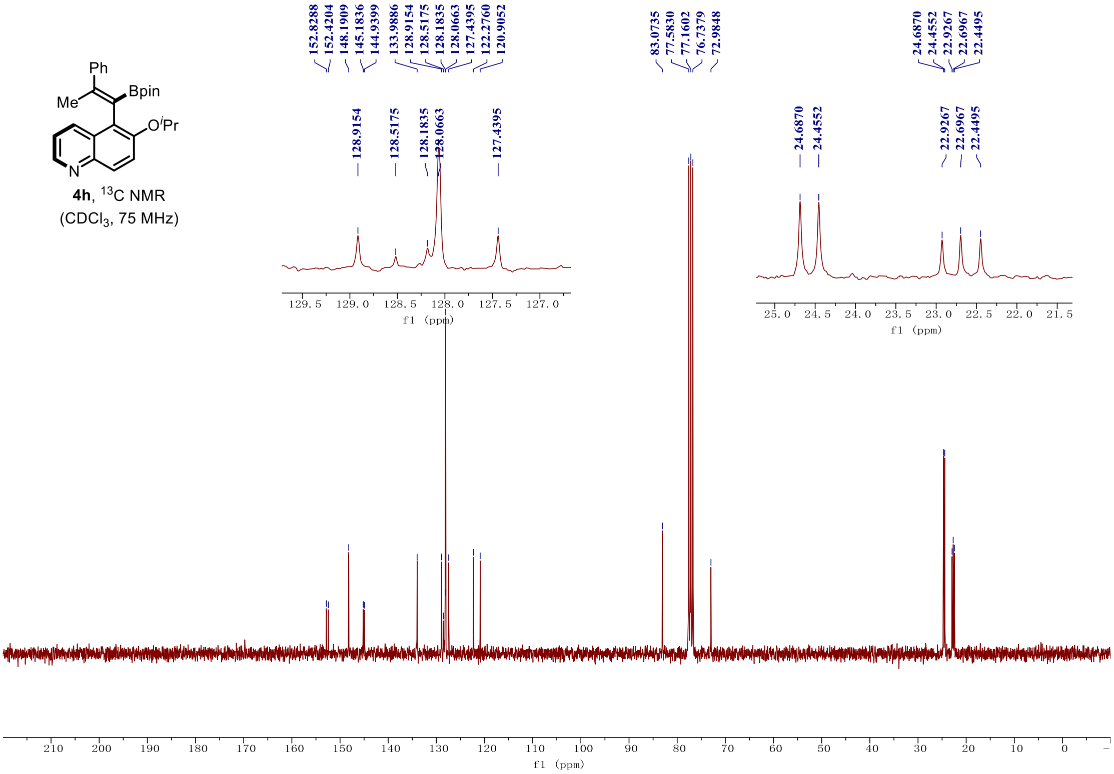

4h, ^{13}C NMR
($CDCl_3$, 75 MHz)

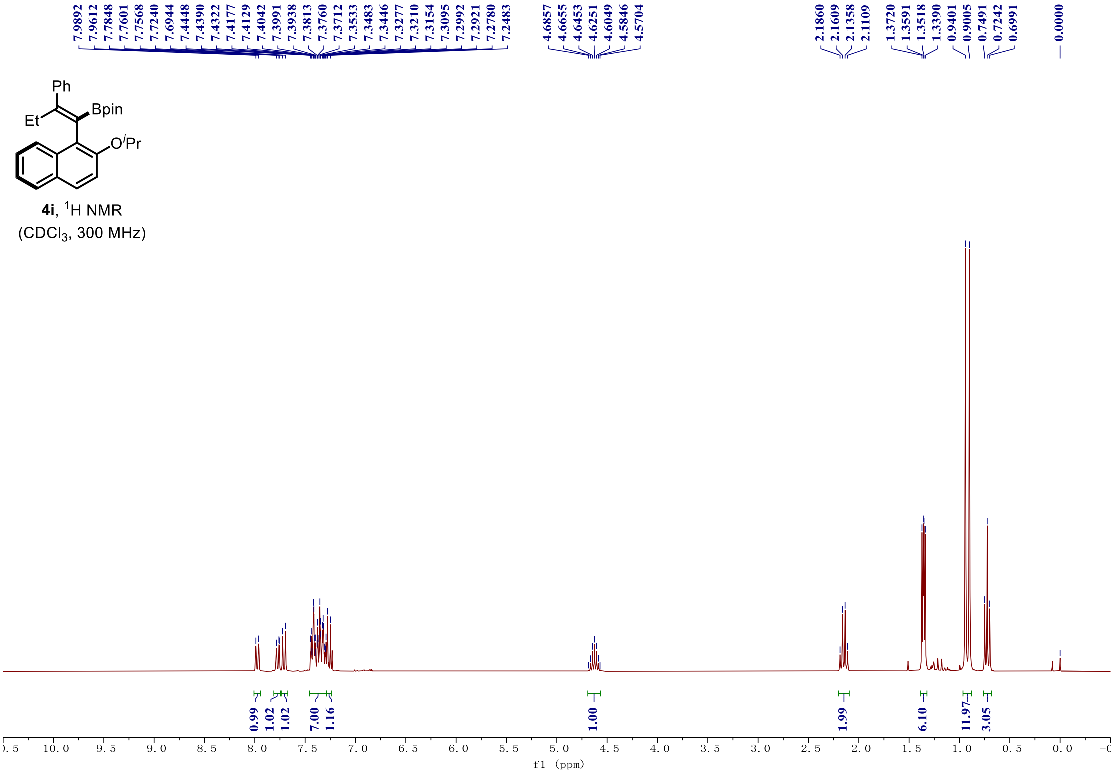

4i, ^{1}H NMR
($CDCl_3$, 300 MHz)

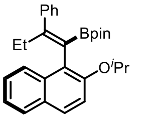

4i, ^{13}C NMR
($CDCl_3$, 75 MHz)

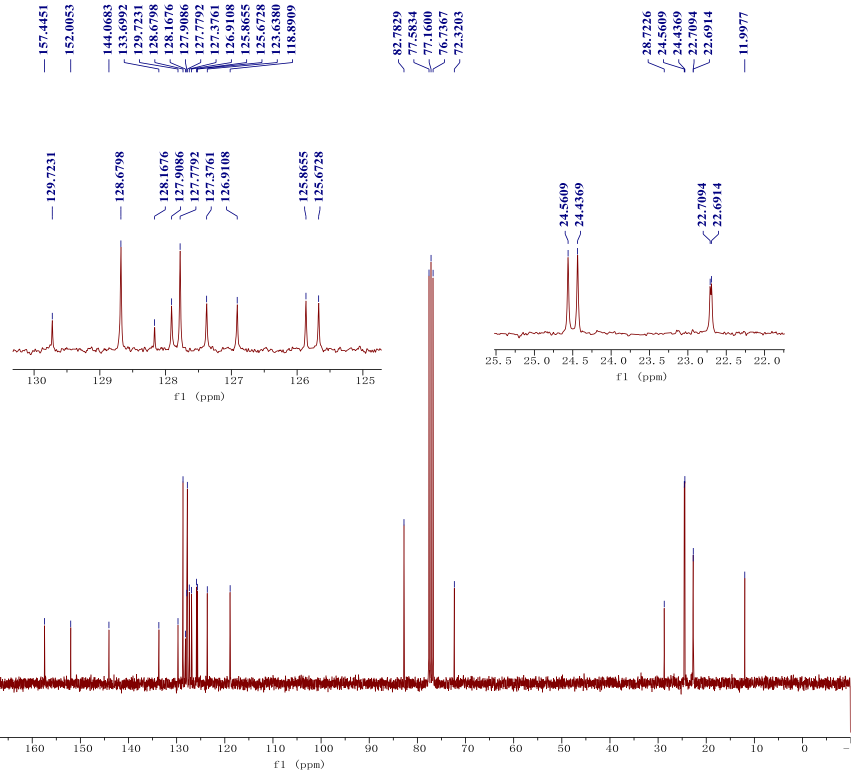

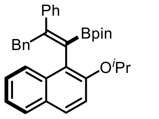

4j, ^{1}H NMR
($CDCl_3$, 300 MHz)

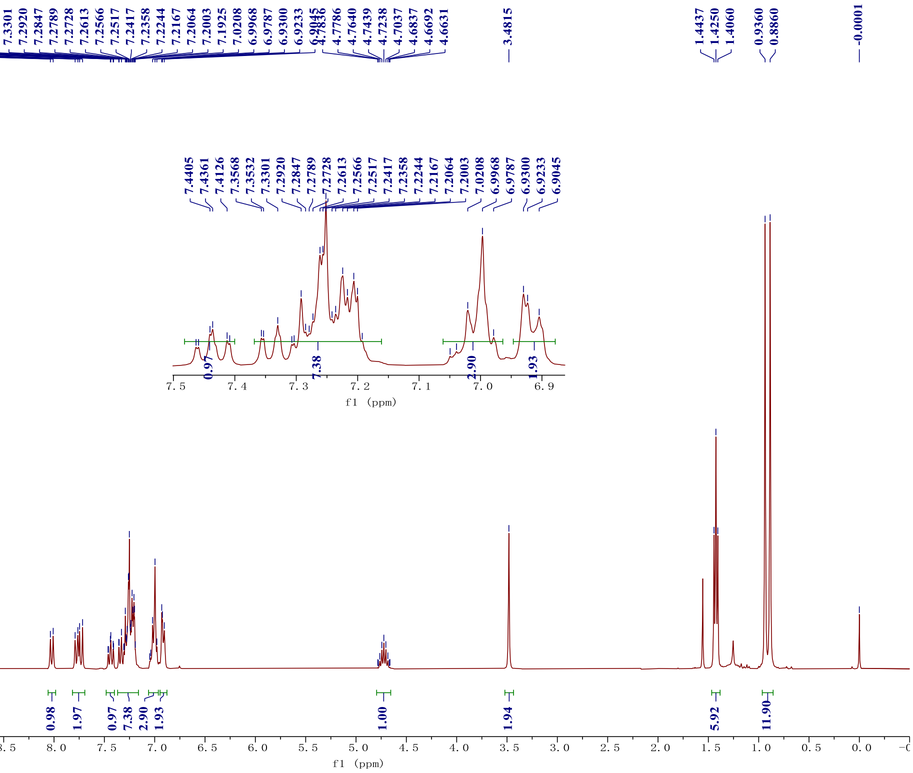

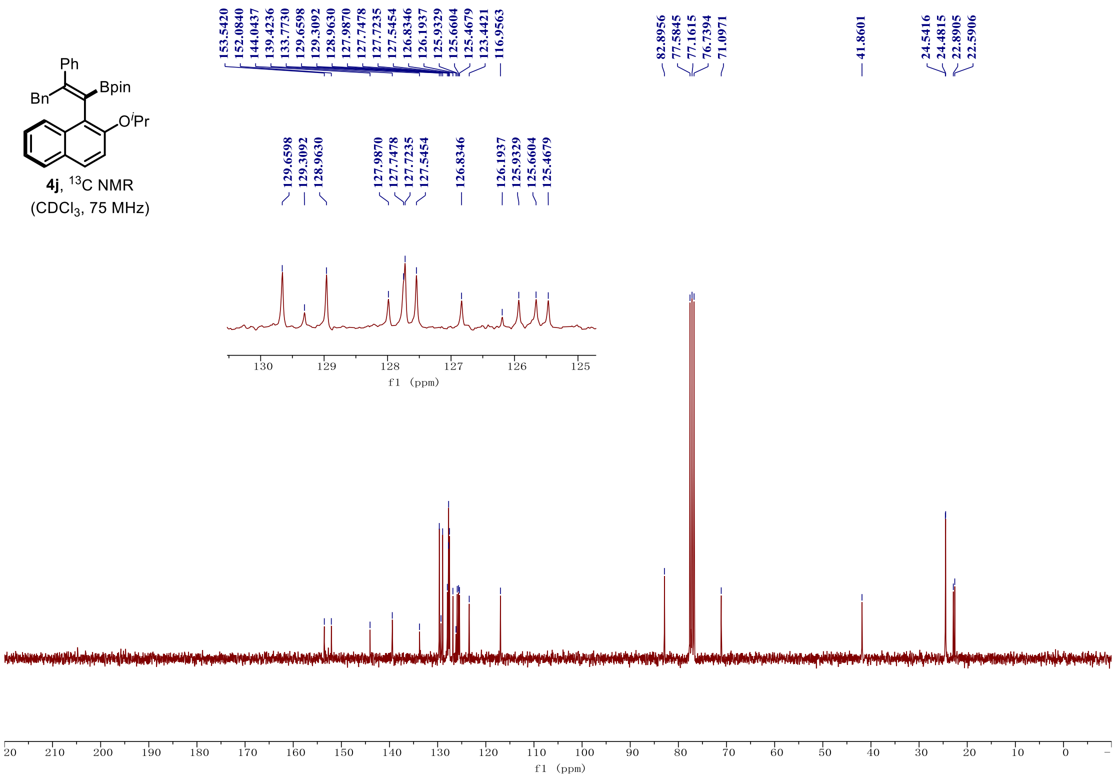
Ph
Bn
Bpin
O^{i}Pr
4j, ^{13}C NMR
(CDCl$_3$, 75 MHz)
153.5420 152.0840 144.0437 139.4236 133.7730 129.6598 129.3092 128.9630 127.9870 127.7478 127.7235 127.5454 126.8346 126.1937 125.9329 125.6604 125.4679 123.4421 116.9563
82.8956 77.5845 77.1615 76.7394 71.0971
41.8601
24.5416 24.4815 22.8905 22.5906
129.6598 129.3092 128.9630 127.9870 127.7478 127.7235 127.5454 126.8346 126.1937 125.9329 125.6604 125.4679
f1 (ppm)

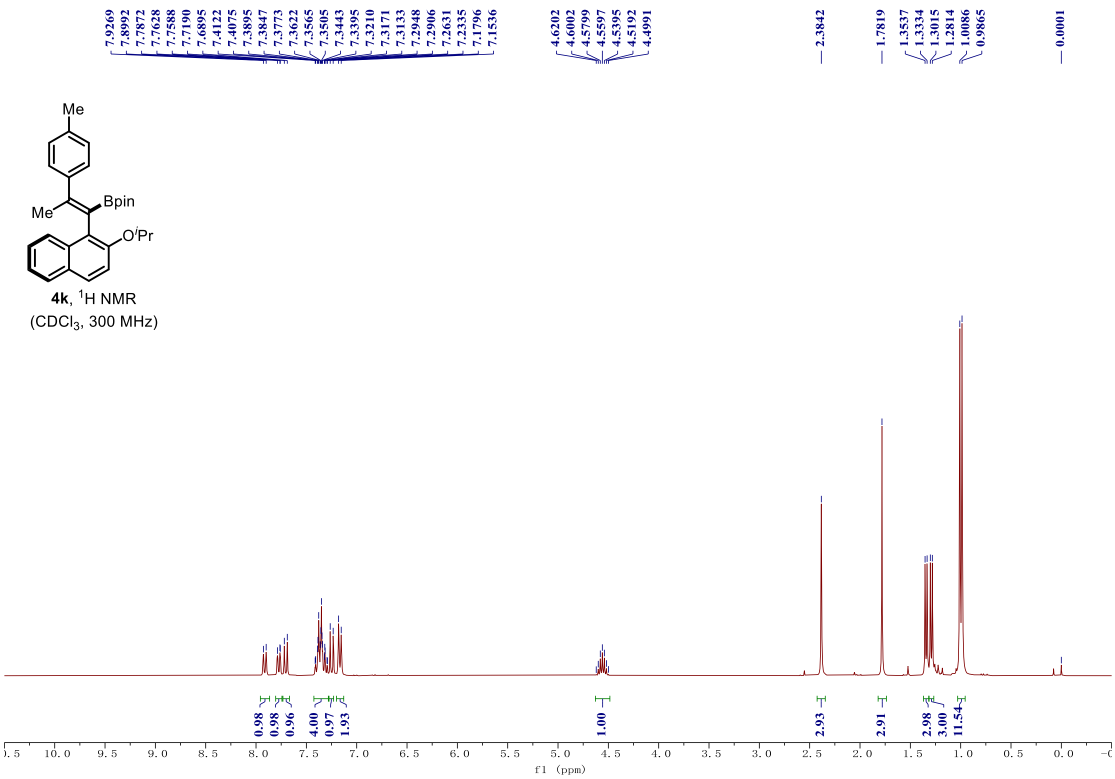
Me
Me
Bpin
O^{i}Pr
4k, ^{1}H NMR
(CDCl$_3$, 300 MHz)
7.9269 7.8992 7.7628 7.7588 7.7190 7.6895 7.4122 7.4075 7.3895 7.3847 7.3773 7.3622 7.3565 7.3505 7.3443 7.3395 7.3210 7.3171 7.3133 7.2948 7.2906 7.2631 7.2335 7.1796 7.1536
4.6202 4.6002 4.5799 4.5597 4.5395 4.5192 4.4991
2.3842
1.7819
1.3537 1.3334 1.3015 1.2814 1.0086 0.9865
0.0001
0.98 0.98 0.96 4.00 0.97 1.93 1.00 2.93 2.91 2.98 3.00 11.54
f1 (ppm)

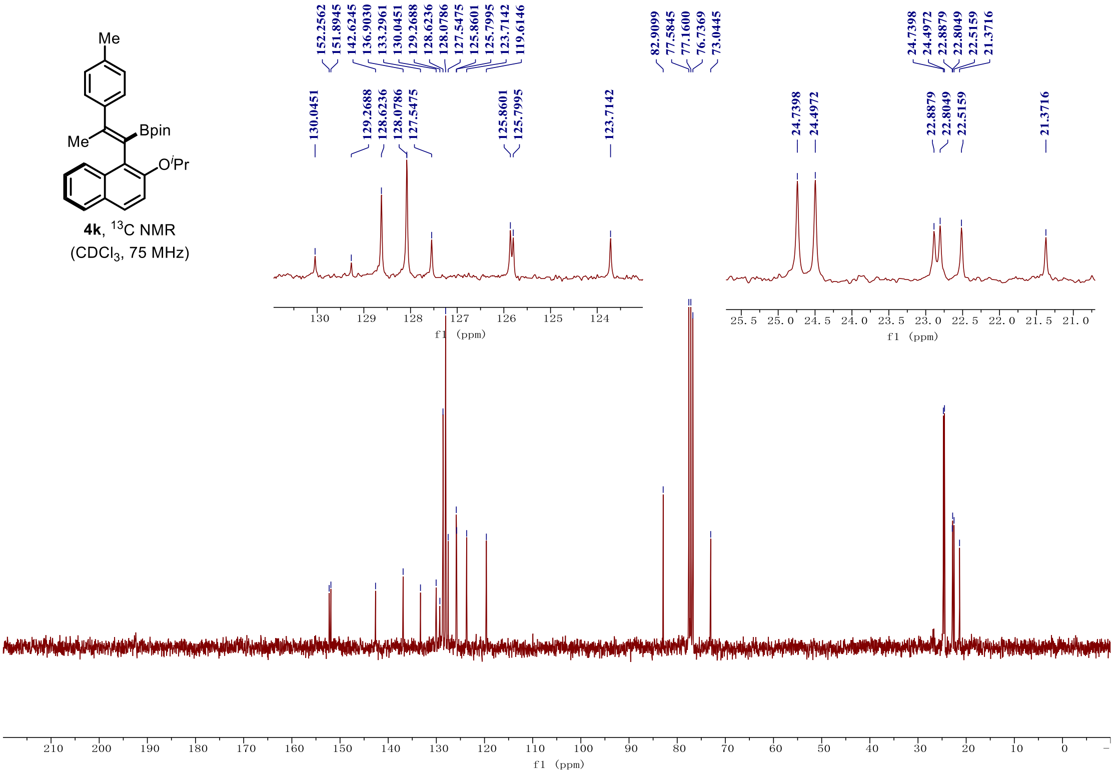

4k, ^{13}C NMR (CDCl$_3$, 75 MHz)

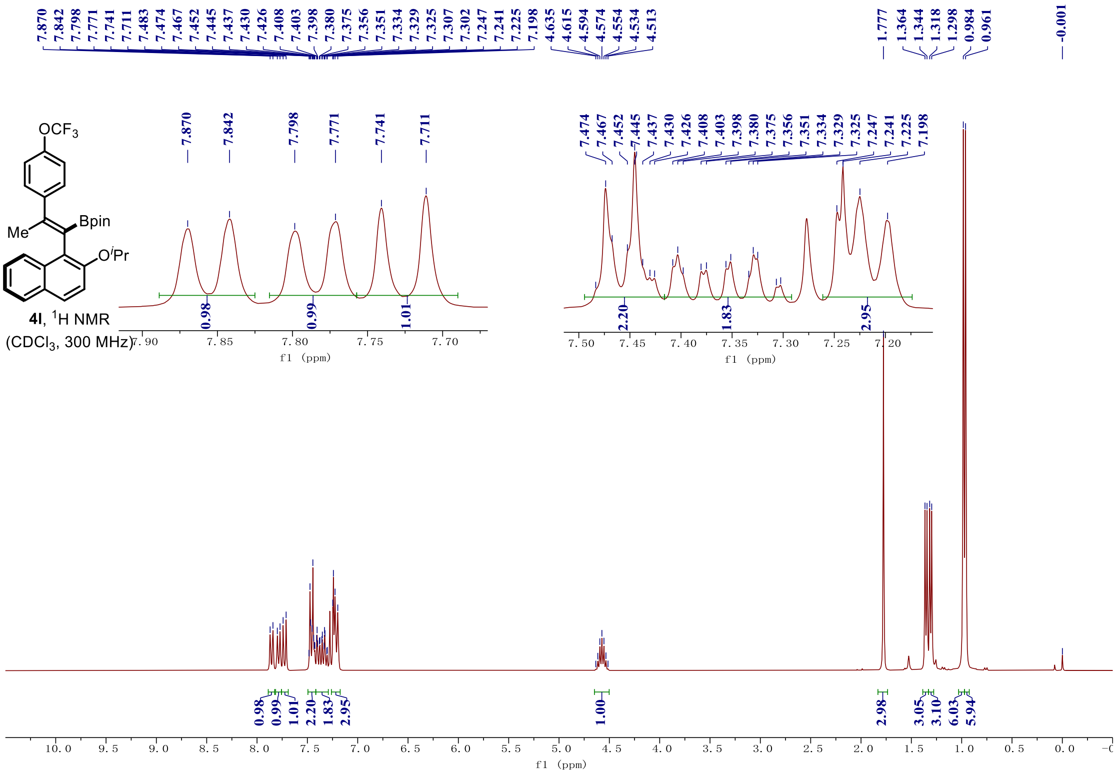

4l, ^{1}H NMR (CDCl$_3$, 300 MHz)

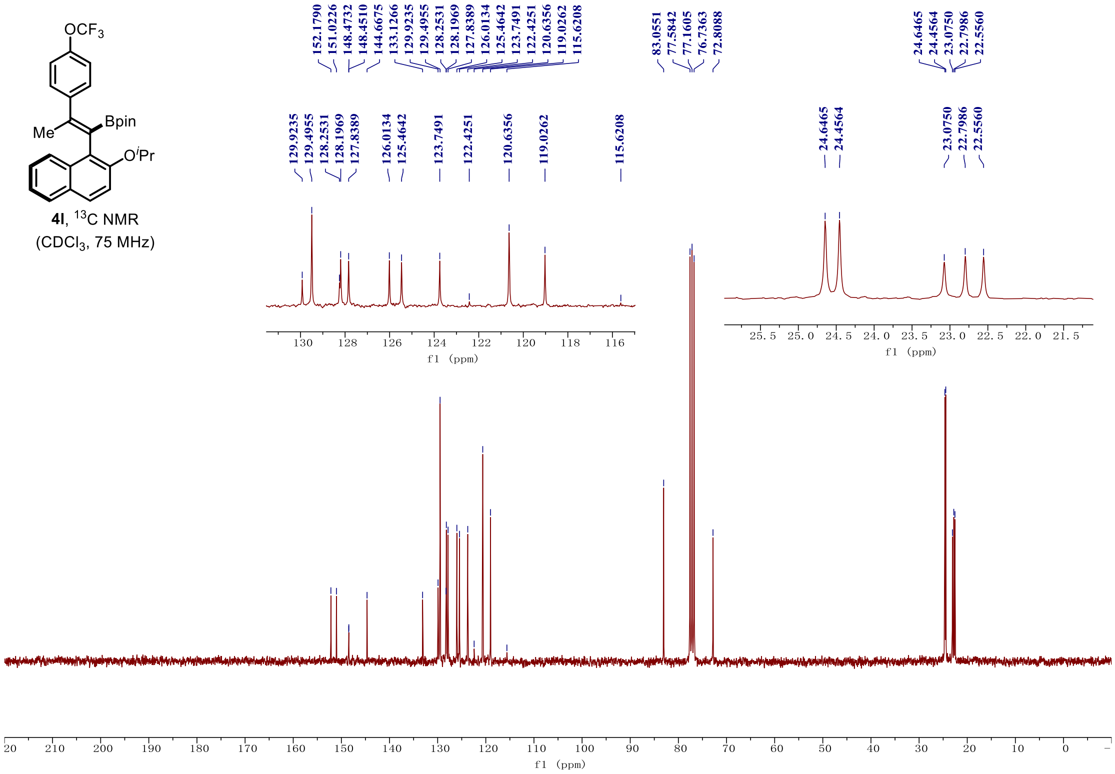

4l, ^{13}C NMR
($CDCl_3$, 75 MHz)

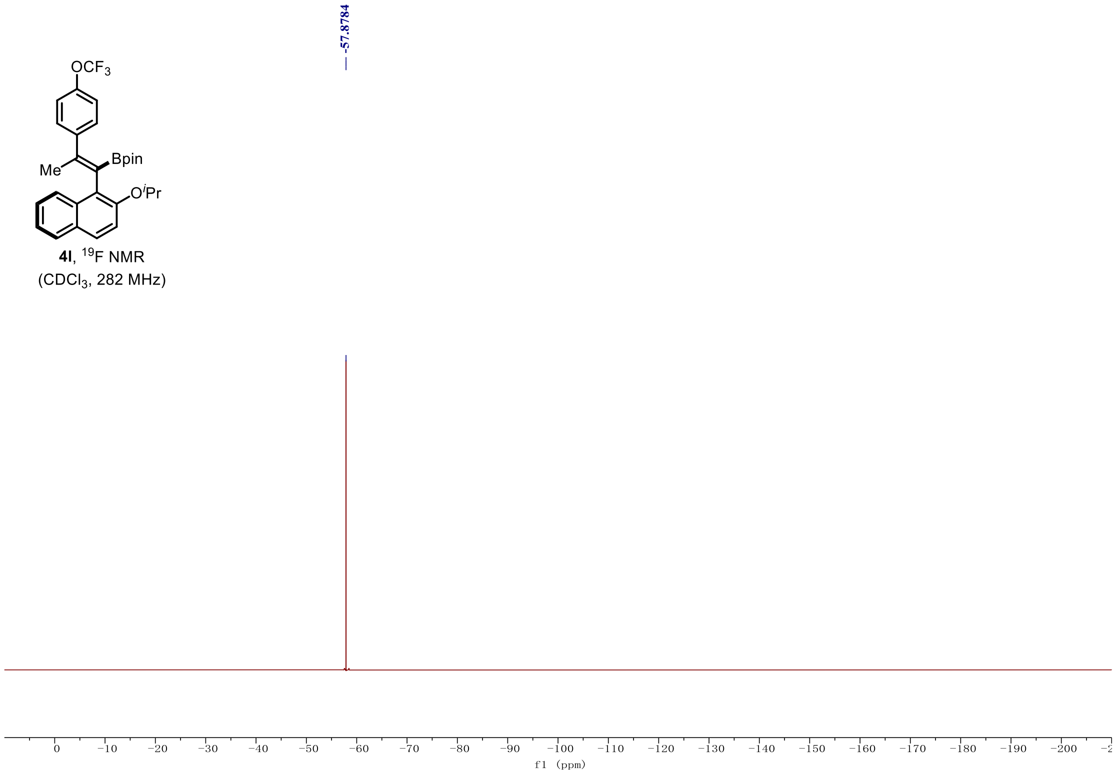

4l, ^{19}F NMR
($CDCl_3$, 282 MHz)

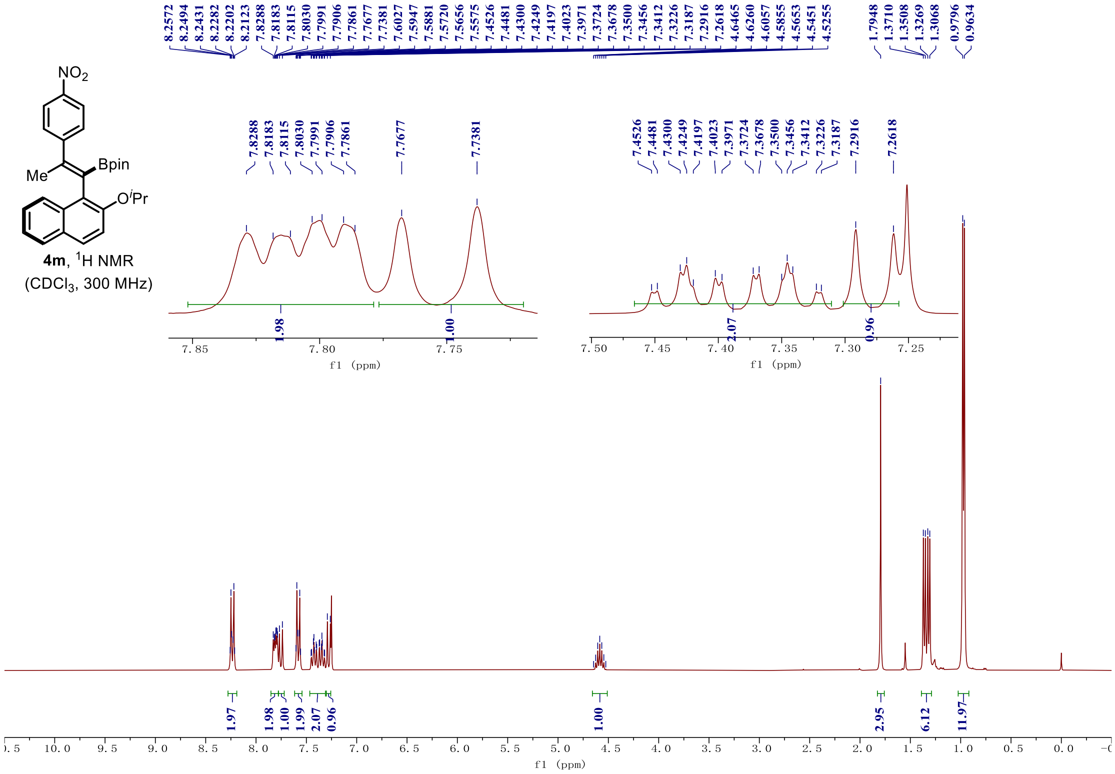
NO2
Me
Bpin
O^iPr
4m, 1H NMR
(CDCl3, 300 MHz)
f1 (ppm)

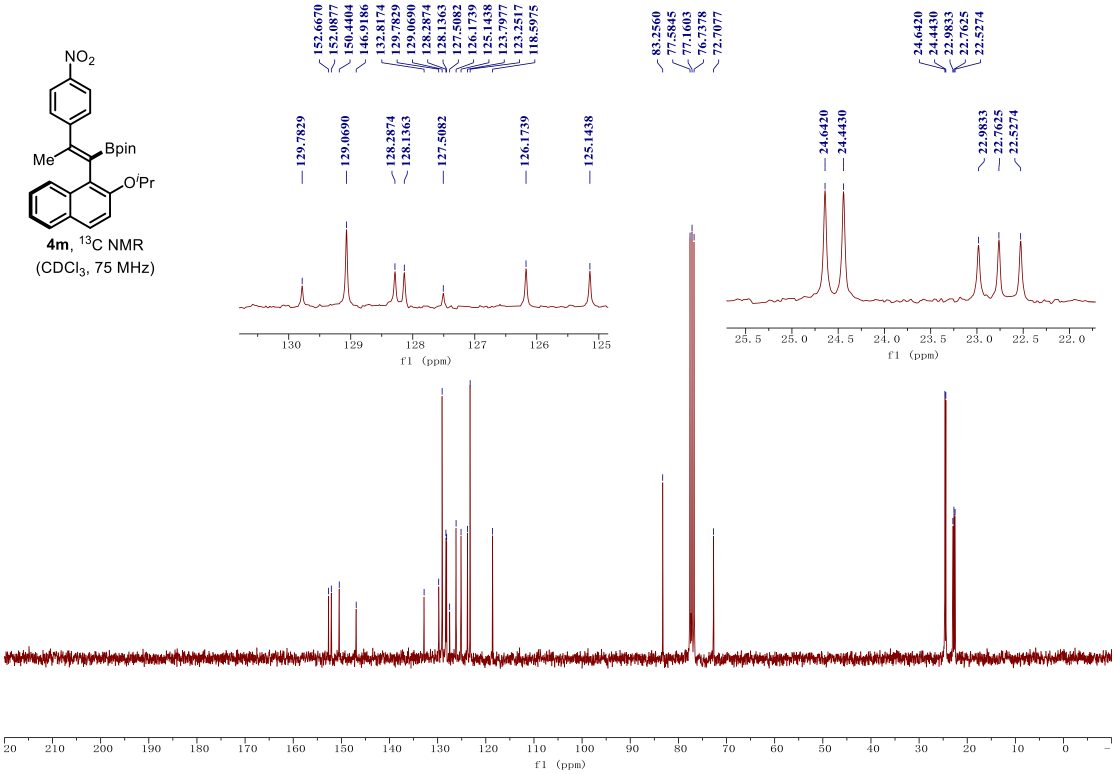
NO2
Me
Bpin
O^iPr
4m, 13C NMR
(CDCl3, 75 MHz)
f1 (ppm)

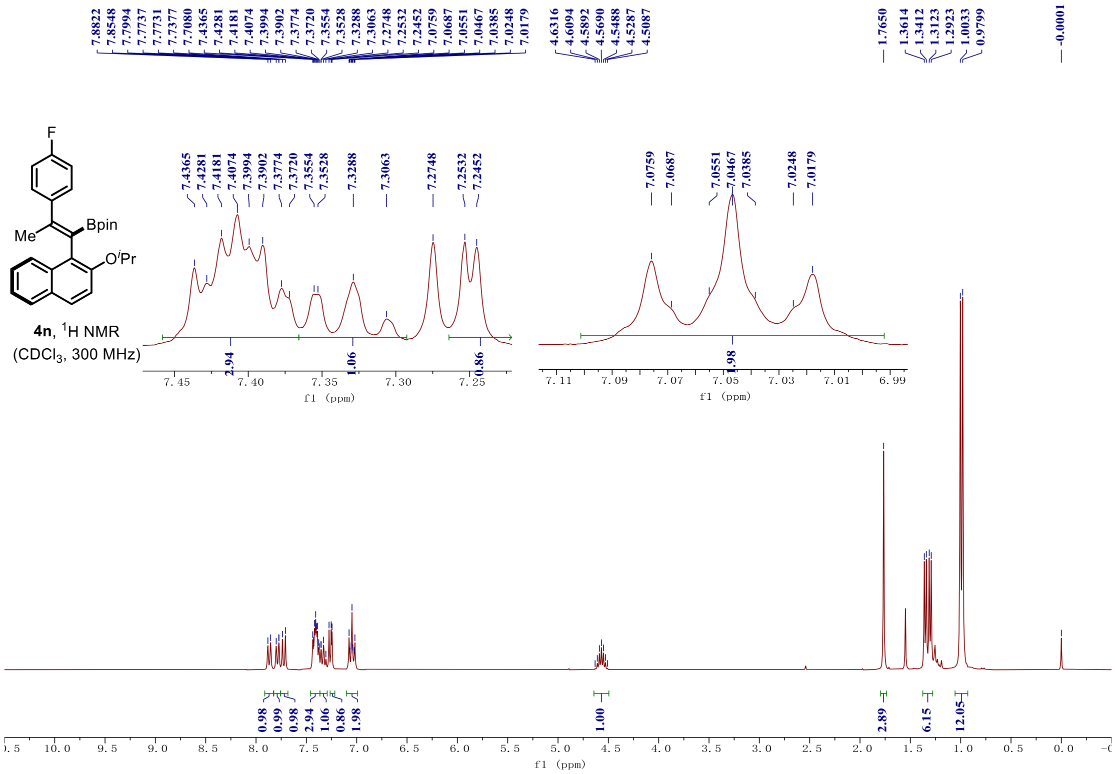

4n, ^{1}H NMR (CDCl$_3$, 300 MHz)

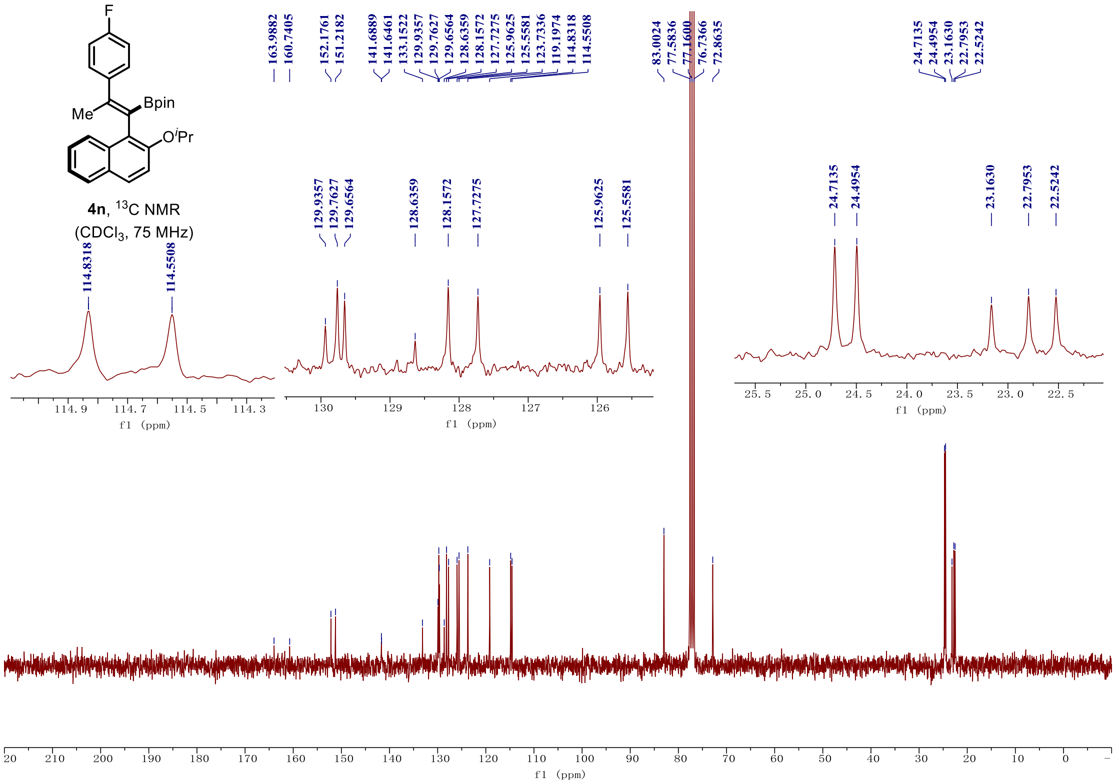

4n, ^{13}C NMR (CDCl$_3$, 75 MHz)

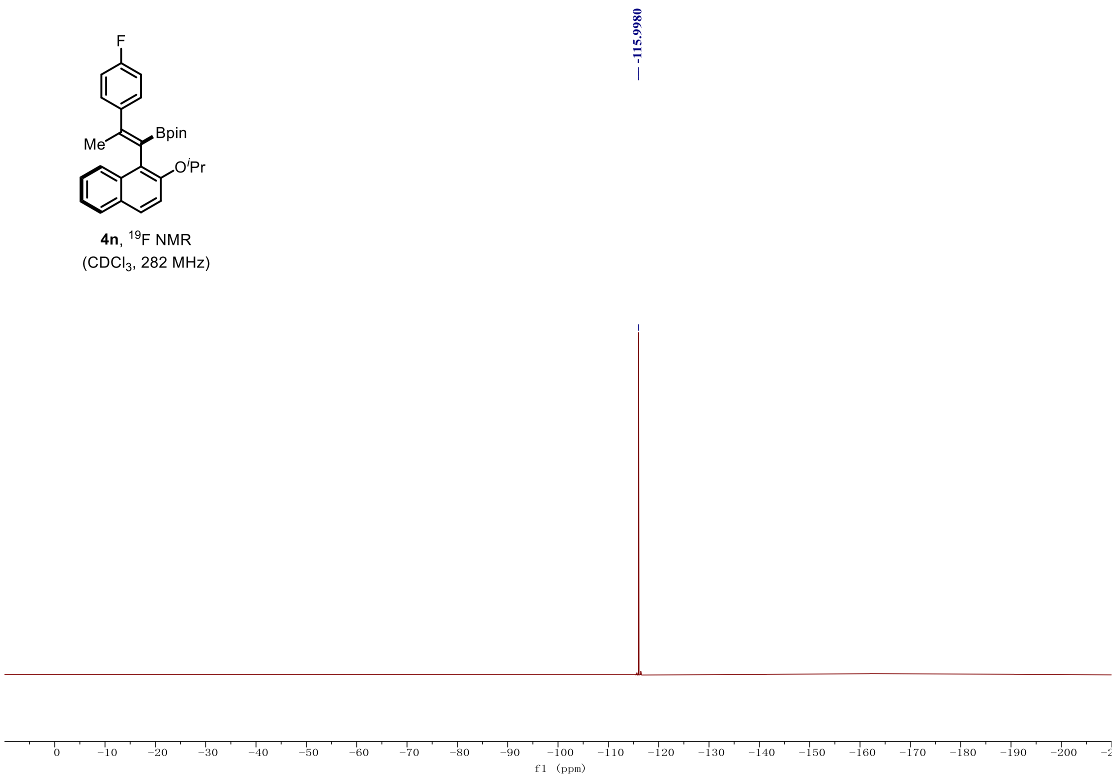

4n, ^{19}F NMR
($CDCl_3$, 282 MHz)

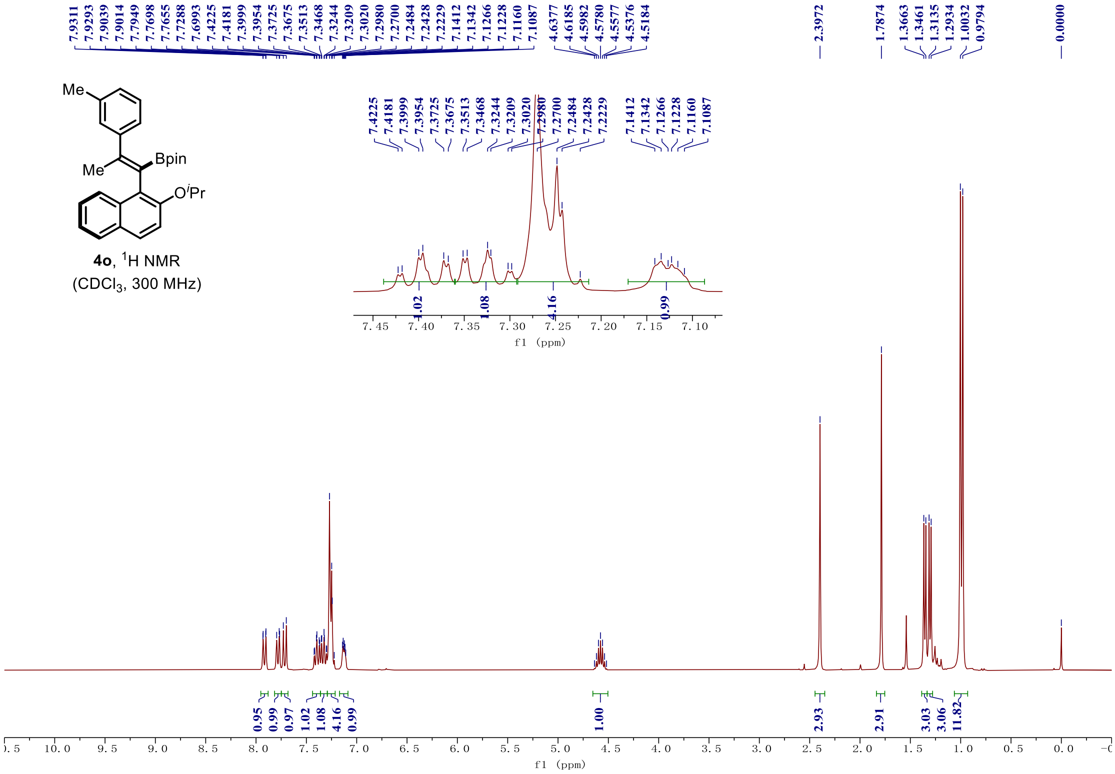

4o, ^{1}H NMR
($CDCl_3$, 300 MHz)

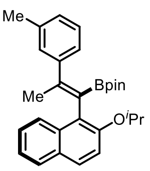

4o, ^{13}C NMR
($CDCl_3$, 75 MHz)

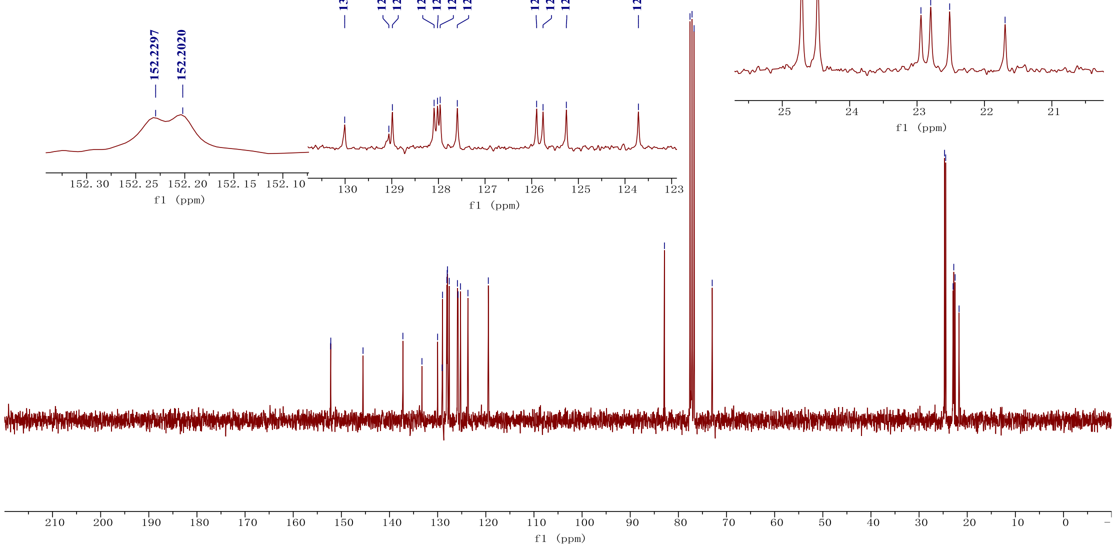

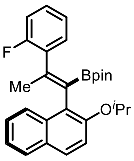

4p, ^{1}H NMR
($CDCl_3$, 300 MHz)

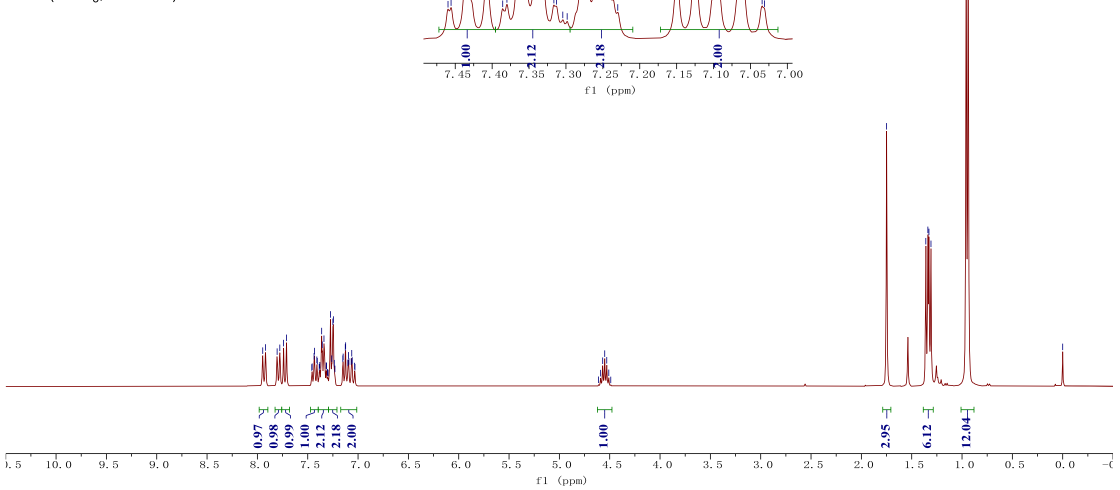

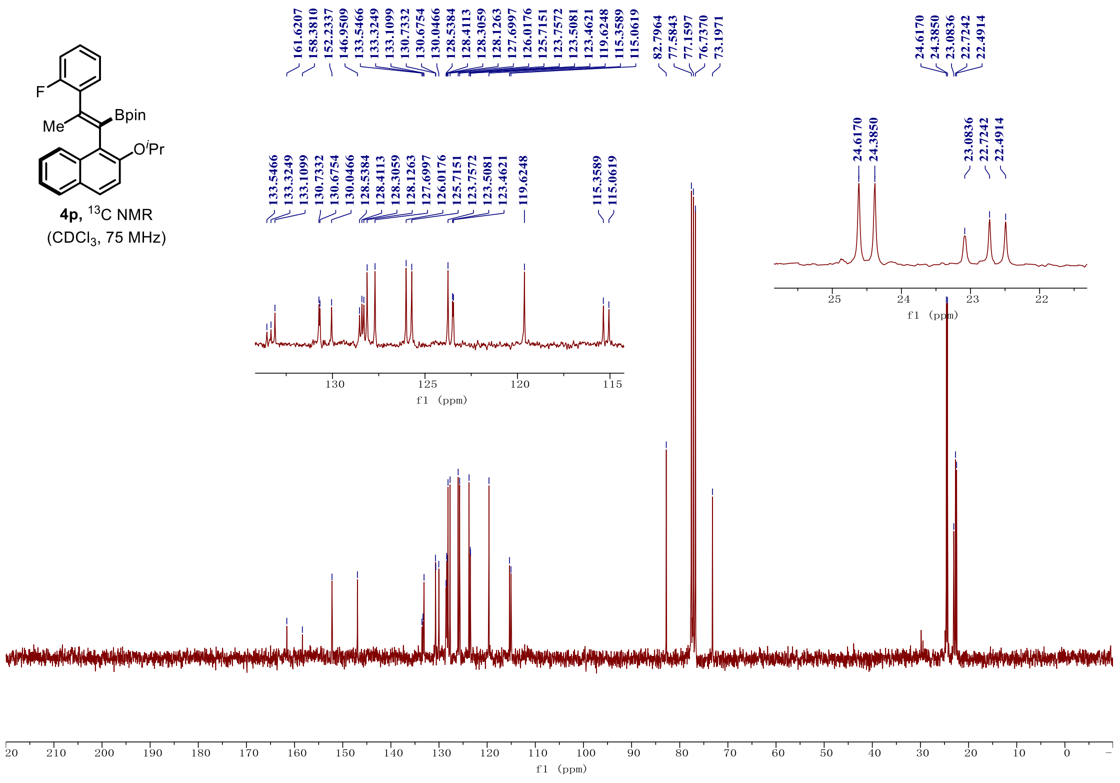

4p, ^{13}C NMR
($CDCl_3$, 75 MHz)

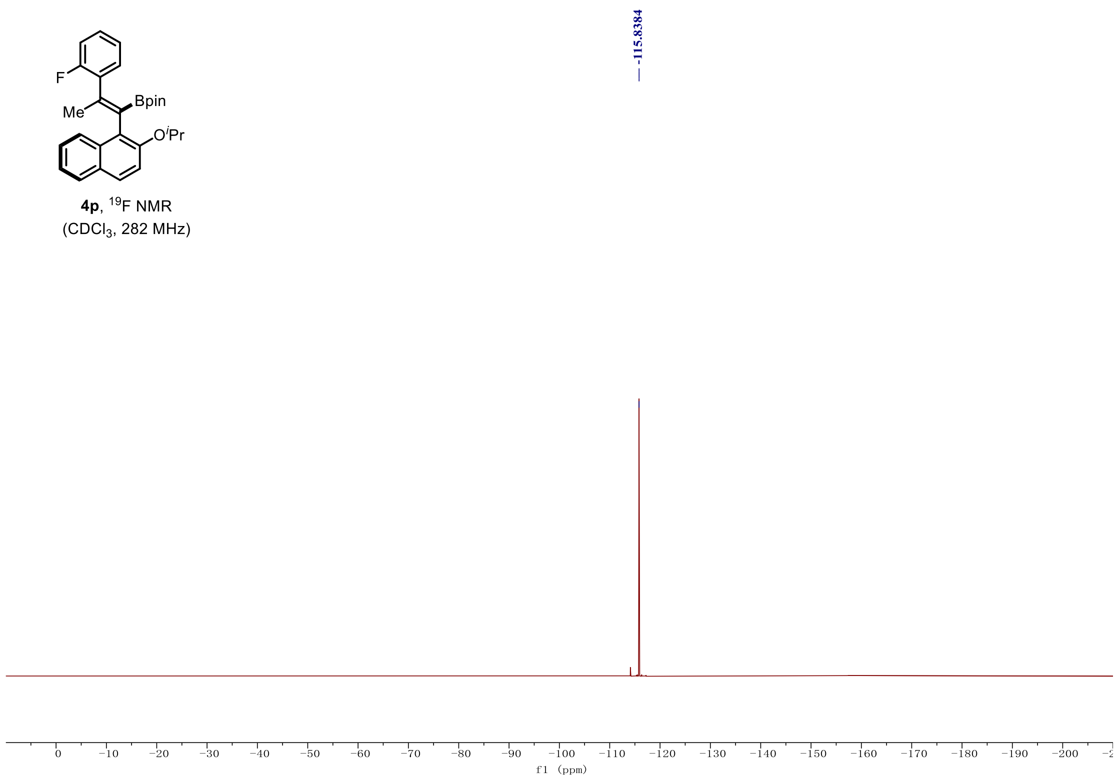

4p, ^{19}F NMR
($CDCl_3$, 282 MHz)

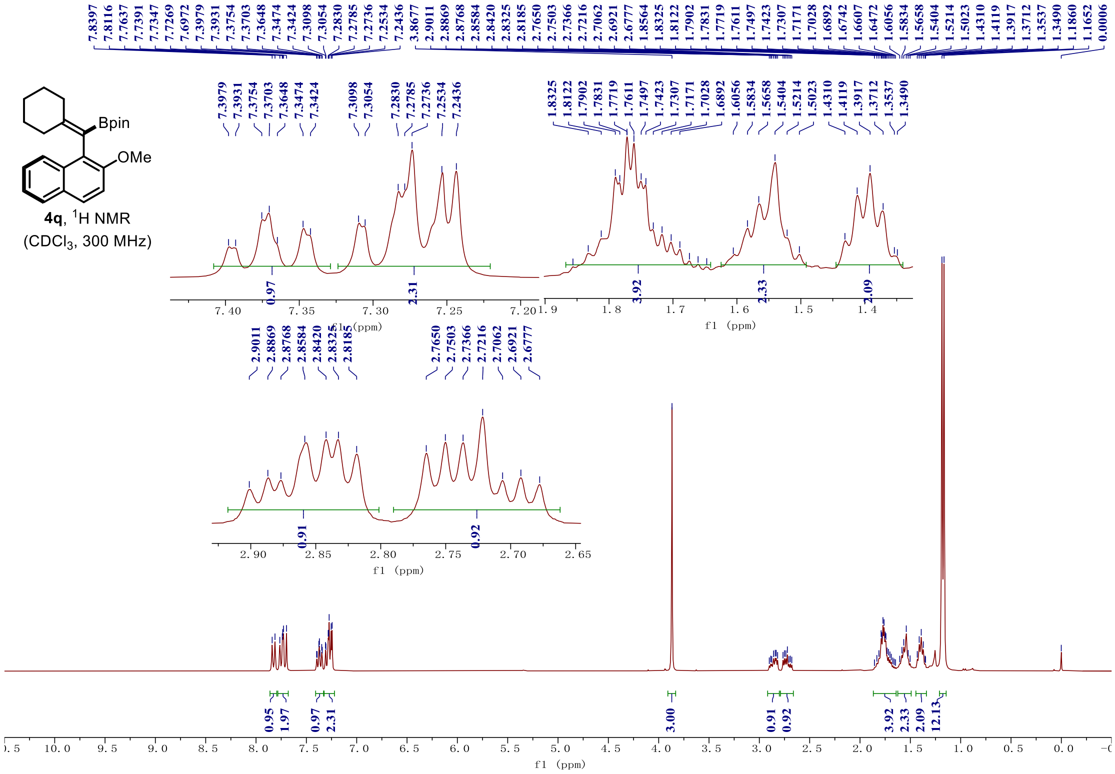
Bpin
OMe
4q, ^{1}H NMR
(CDCl$_3$, 300 MHz)
7.8397 7.8116 7.7637 7.7391 7.7347 7.7269 7.6972 7.3979 7.3931 7.3754 7.3703 7.3648 7.3474 7.3424 7.3098 7.3054 7.2830 7.2785 7.2736 7.2534 7.2436 3.8677 2.9011 2.8869 2.8768 2.8584 2.8420 2.8325 2.8185 2.7650 2.7503 2.7366 2.7216 2.7062 2.6921 2.6777 1.8564 1.8325 1.8122 1.7902 1.7831 1.7719 1.7611 1.7497 1.7423 1.7307 1.7171 1.7028 1.6892 1.6742 1.6607 1.6472 1.6056 1.5834 1.5658 1.5404 1.5214 1.5023 1.4310 1.4119 1.3917 1.3712 1.3537 1.3490 1.1860 1.1652 0.0006
0.95 1.97 0.97 2.31 3.00 0.91 0.92 3.92 2.33 2.09 12.13
f1 (ppm)

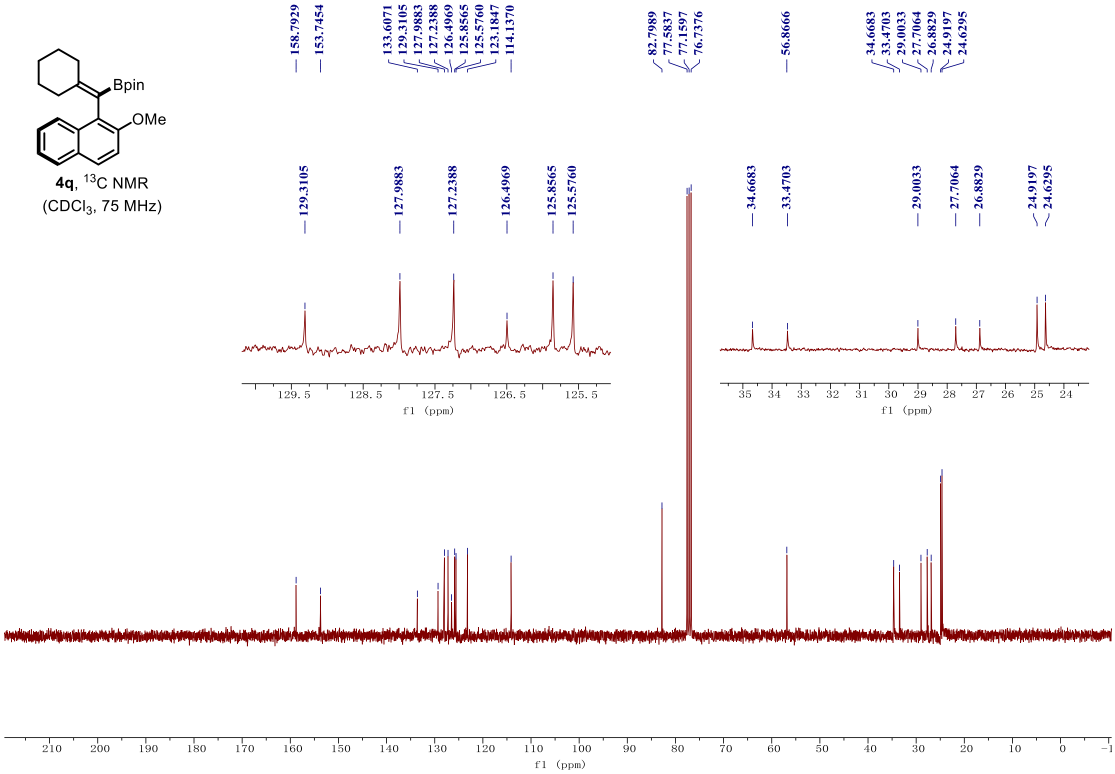
Bpin
OMe
4q, ^{13}C NMR
(CDCl$_3$, 75 MHz)
158.7929 153.7454 133.6071 129.3105 127.9883 127.2388 126.4969 125.8565 125.5760 123.1847 114.1370 82.7989 77.5837 77.1597 76.7376 56.8666 34.6683 33.4703 29.0033 27.7064 26.8829 24.9197 24.6295
f1 (ppm)

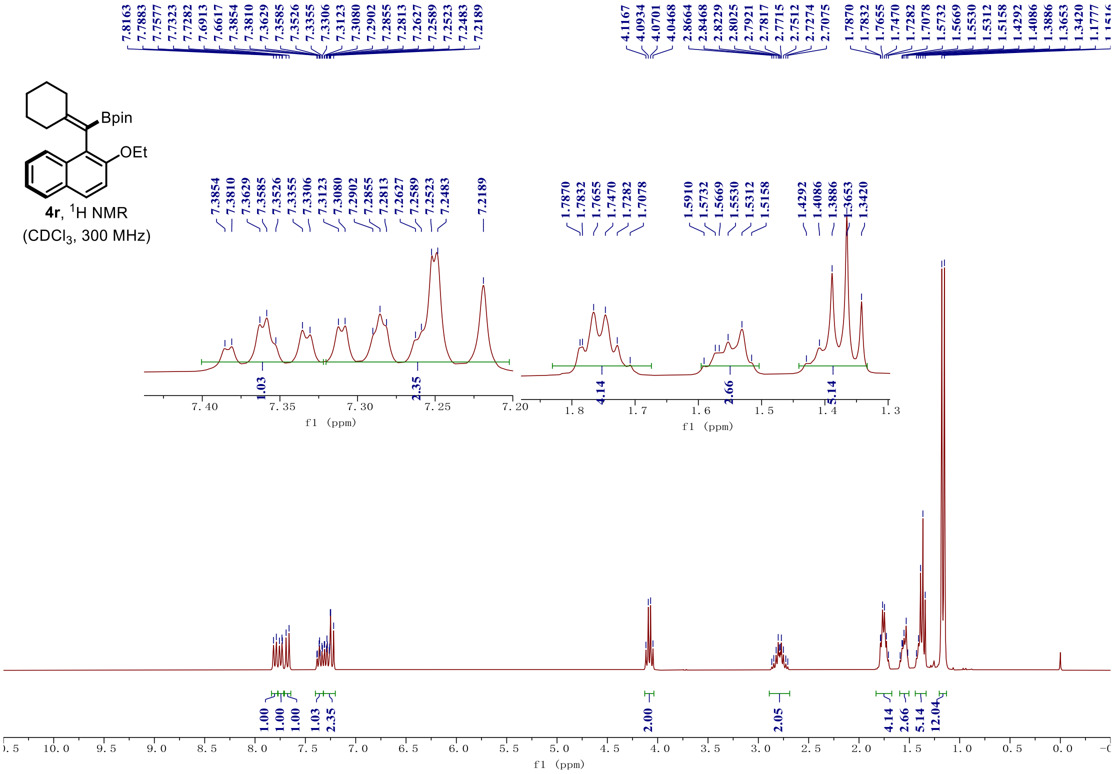

4r, ^{1}H NMR
($CDCl_3$, 300 MHz)

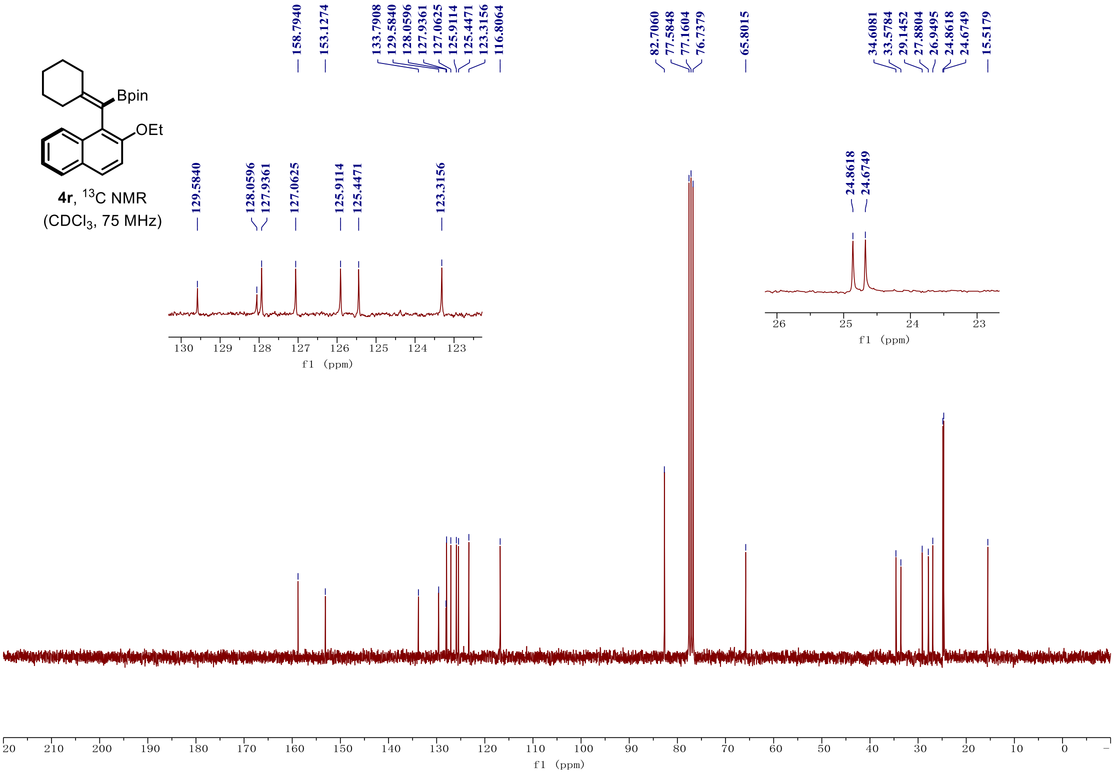

4r, ^{13}C NMR
($CDCl_3$, 75 MHz)

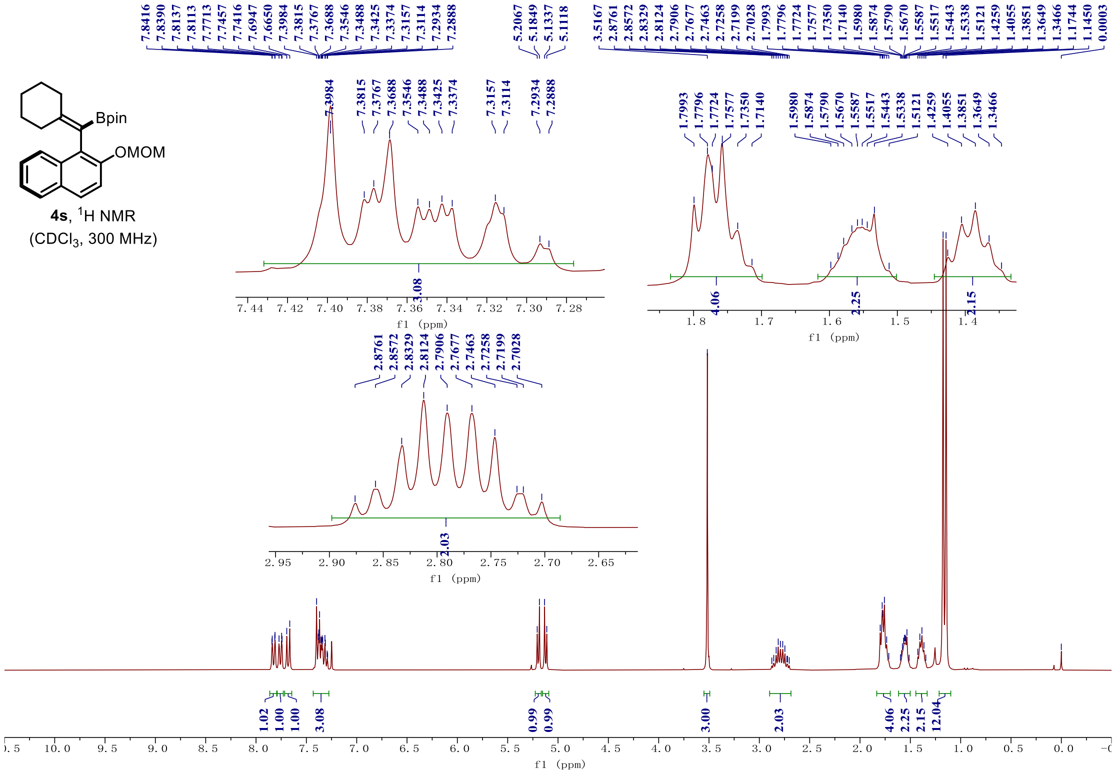

4s, ^{1}H NMR
($CDCl_3$, 300 MHz)

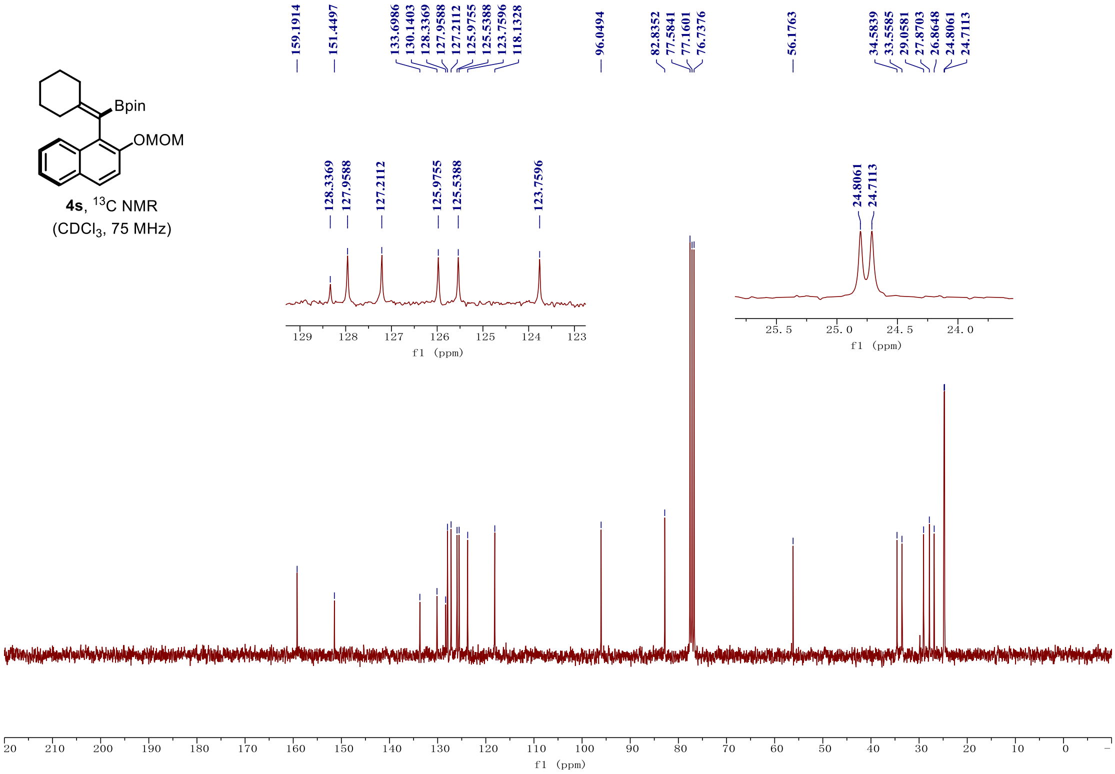

4s, ^{13}C NMR
($CDCl_3$, 75 MHz)

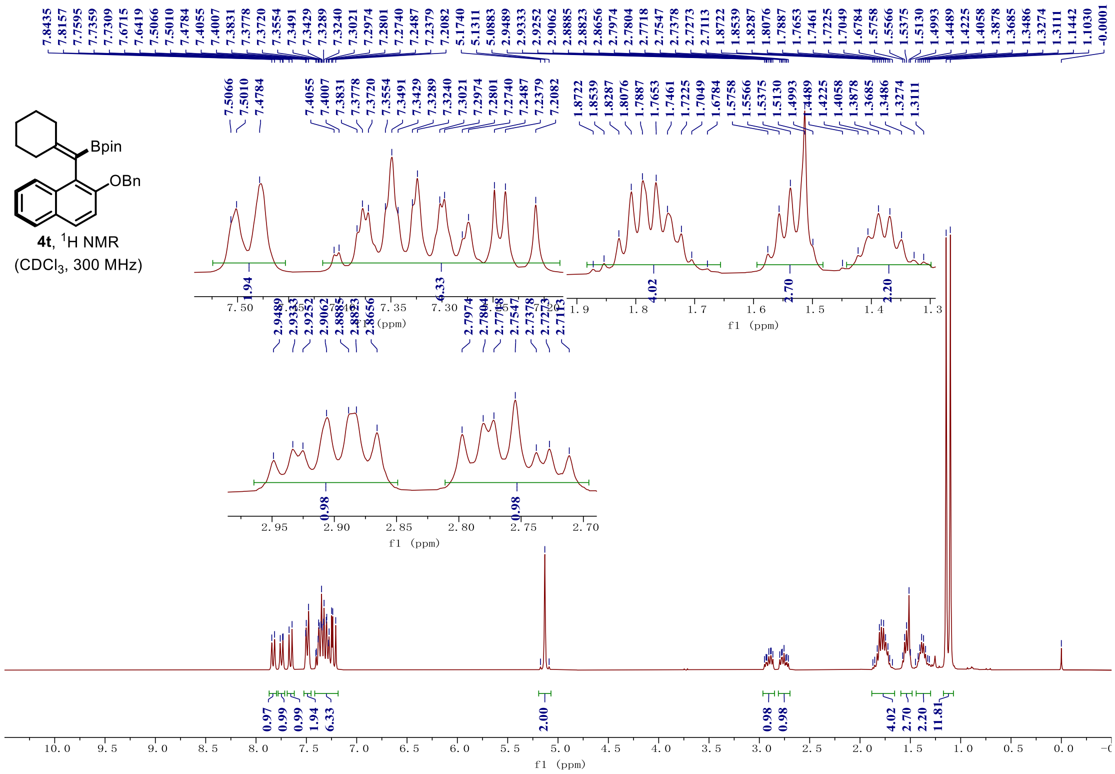

4t, ^{1}H NMR (CDCl$_3$, 300 MHz)

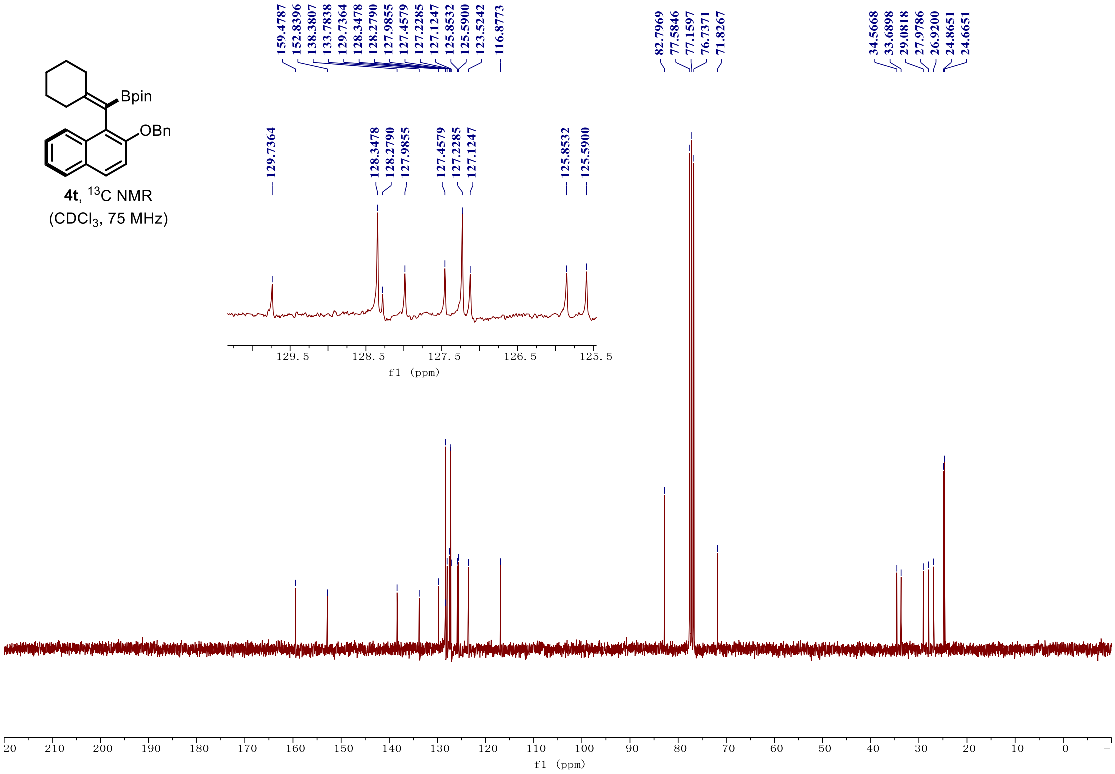

4t, ^{13}C NMR (CDCl$_3$, 75 MHz)

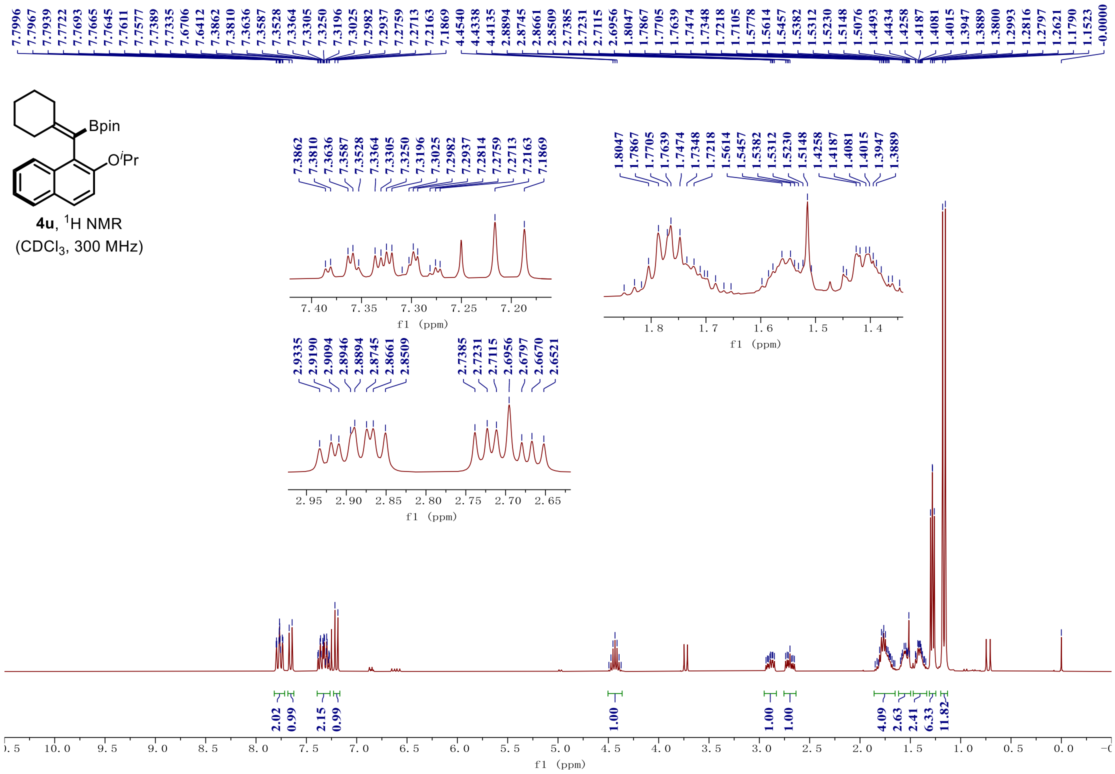
Bpin
O^{i}Pr
4u, ^{1}H NMR
(CDCl$_3$, 300 MHz)
f1 (ppm)

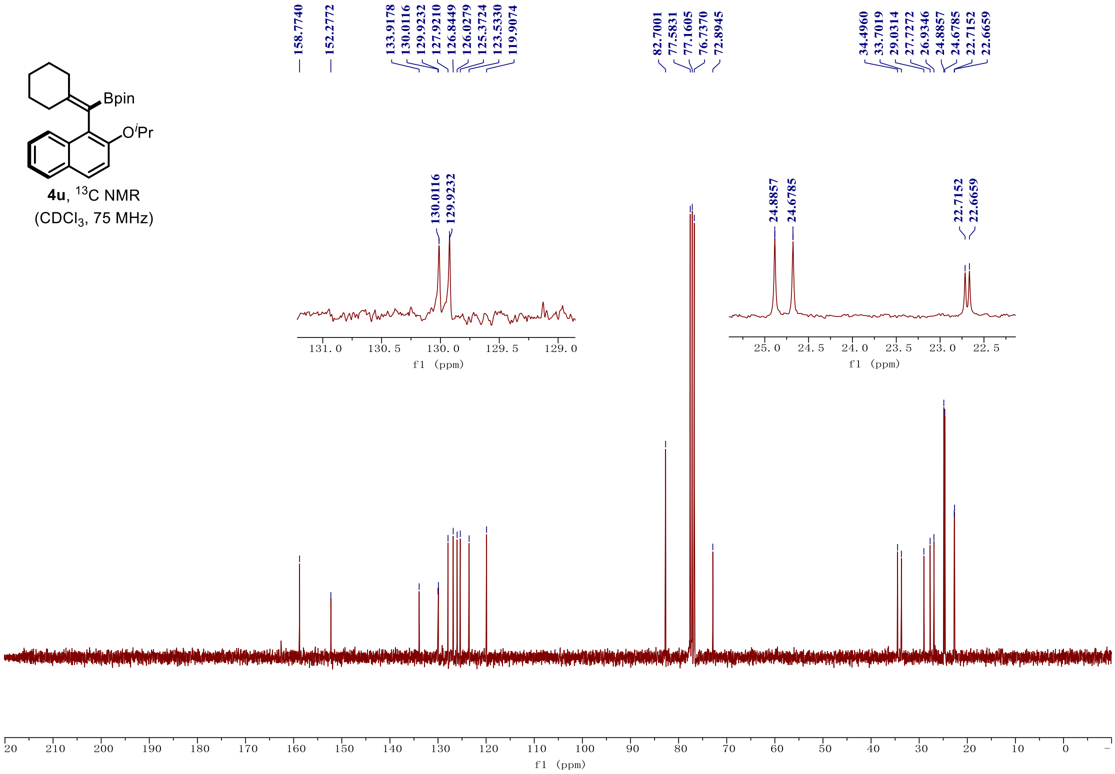
Bpin
O^{i}Pr
4u, ^{13}C NMR
(CDCl$_3$, 75 MHz)
f1 (ppm)

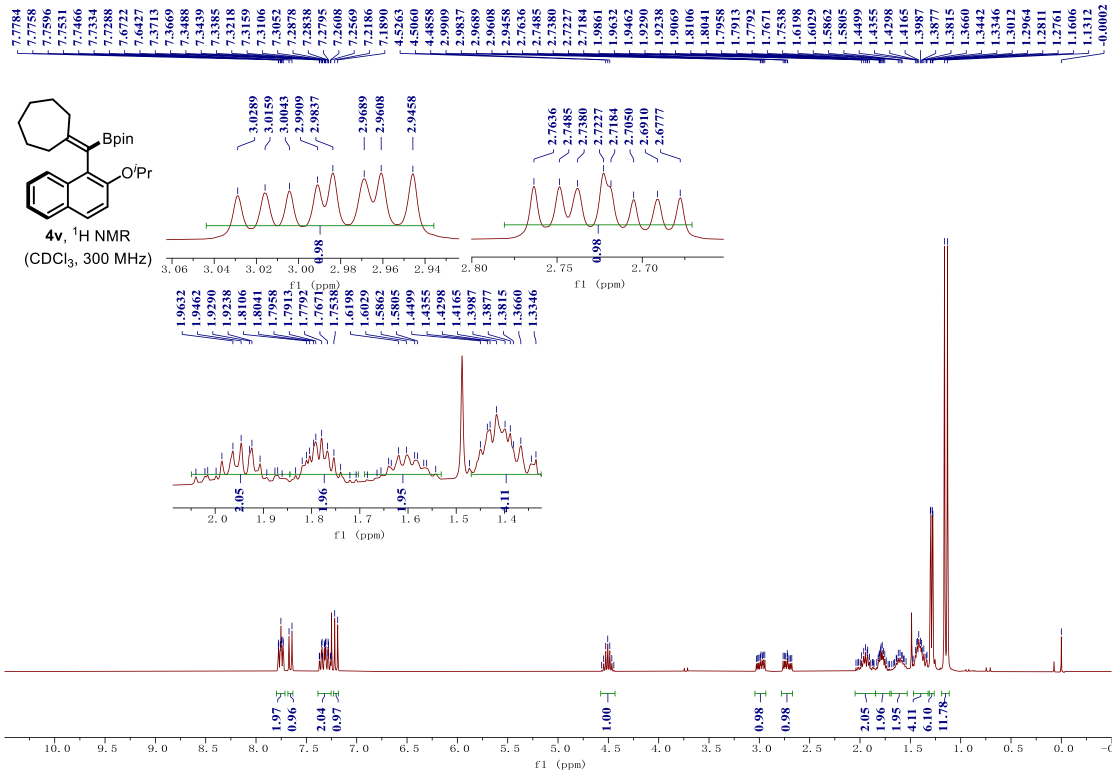

Bpin
O^iPr
4v, 1H NMR
(CDCl3, 300 MHz)
f1 (ppm)

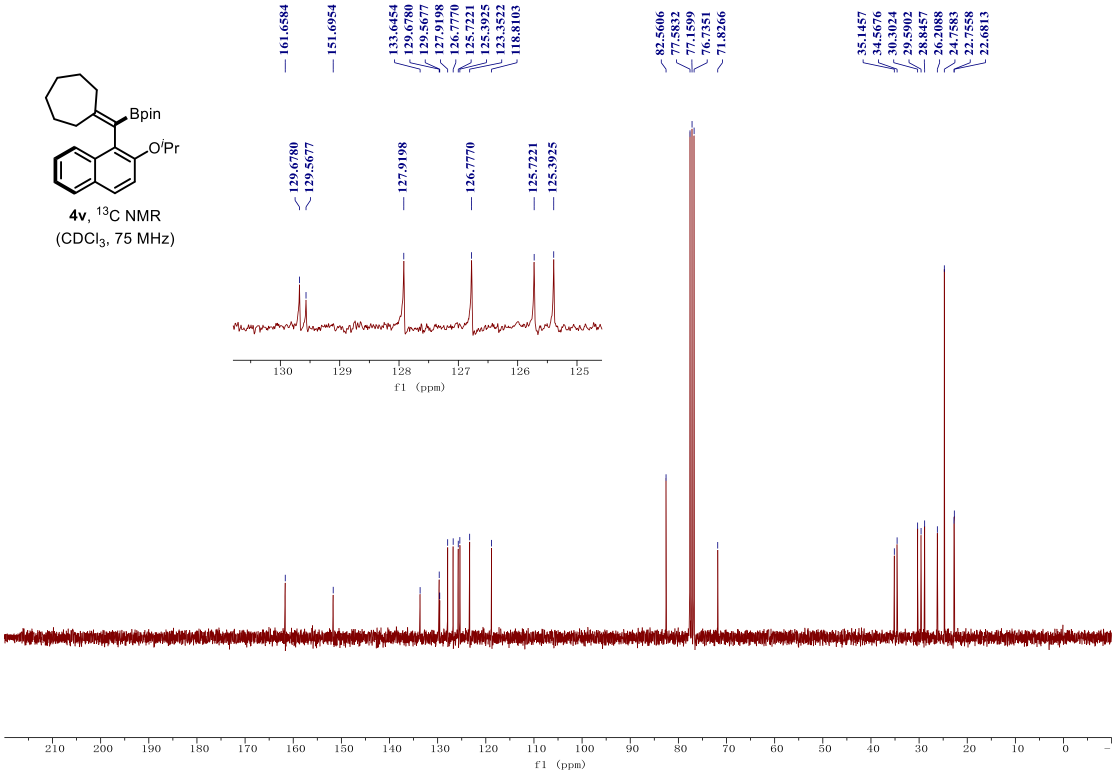

Bpin
O^iPr
4v, 13C NMR
(CDCl3, 75 MHz)
f1 (ppm)

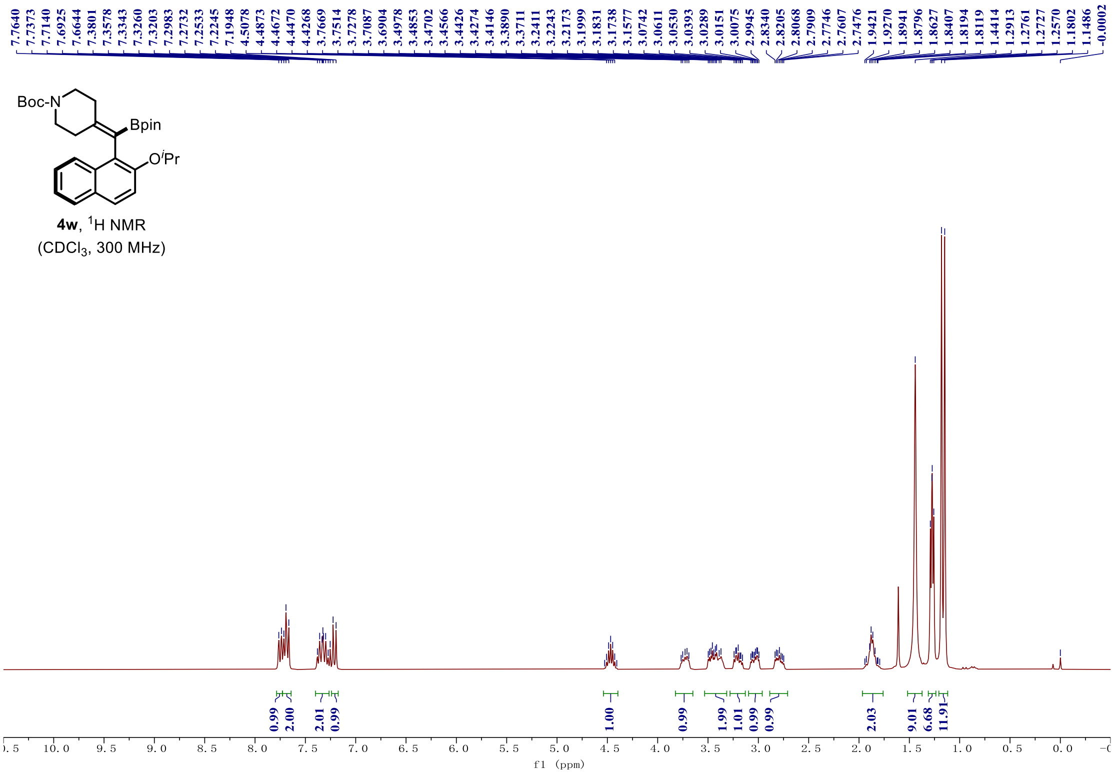

4w, ^{1}H NMR
($CDCl_3$, 300 MHz)

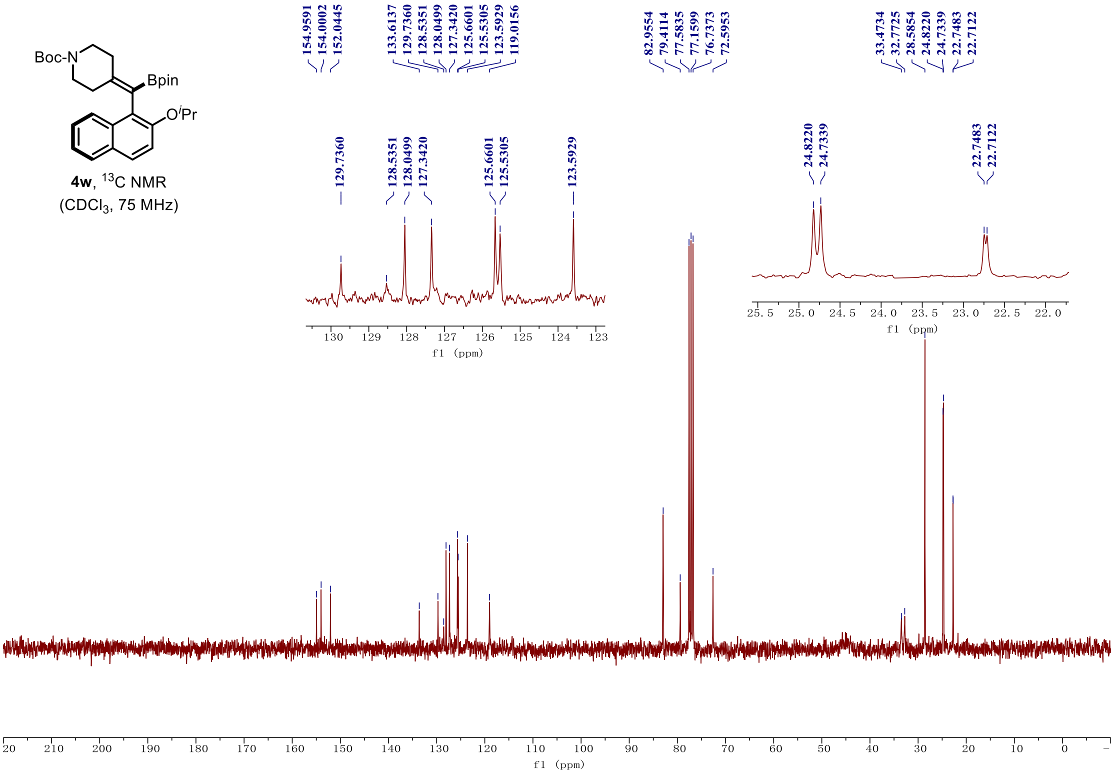

4w, ^{13}C NMR
($CDCl_3$, 75 MHz)

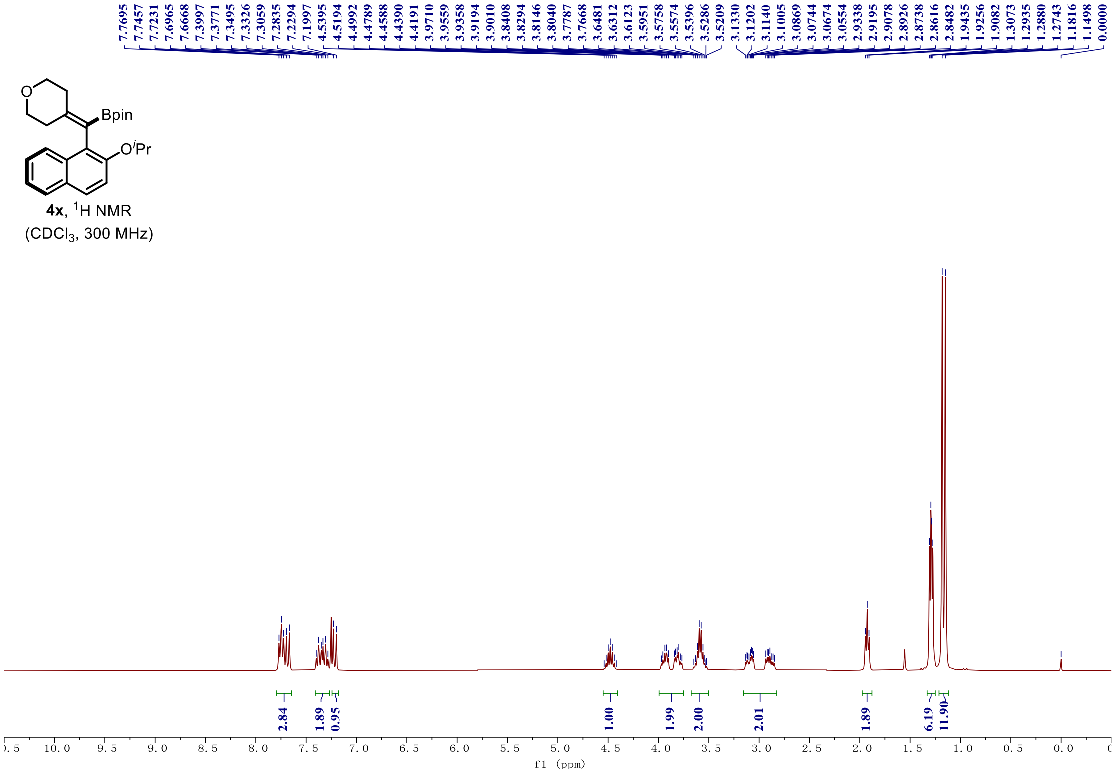

4x, ^{1}H NMR
($CDCl_3$, 300 MHz)

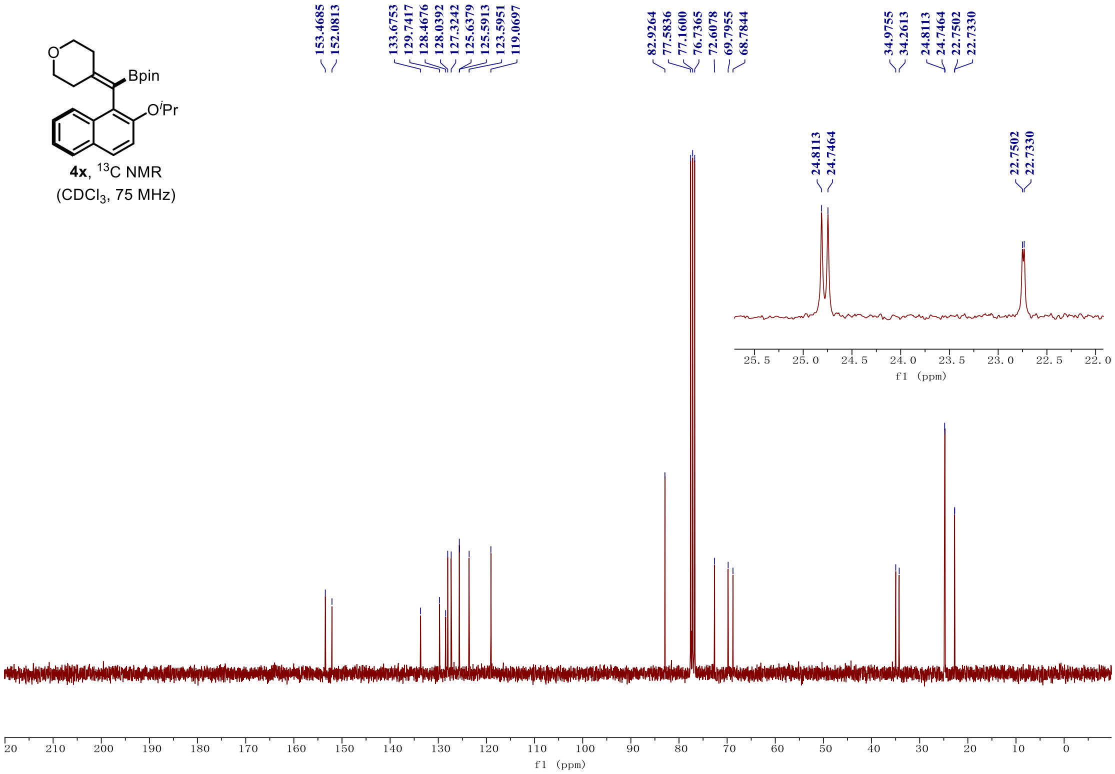

4x, ^{13}C NMR
($CDCl_3$, 75 MHz)

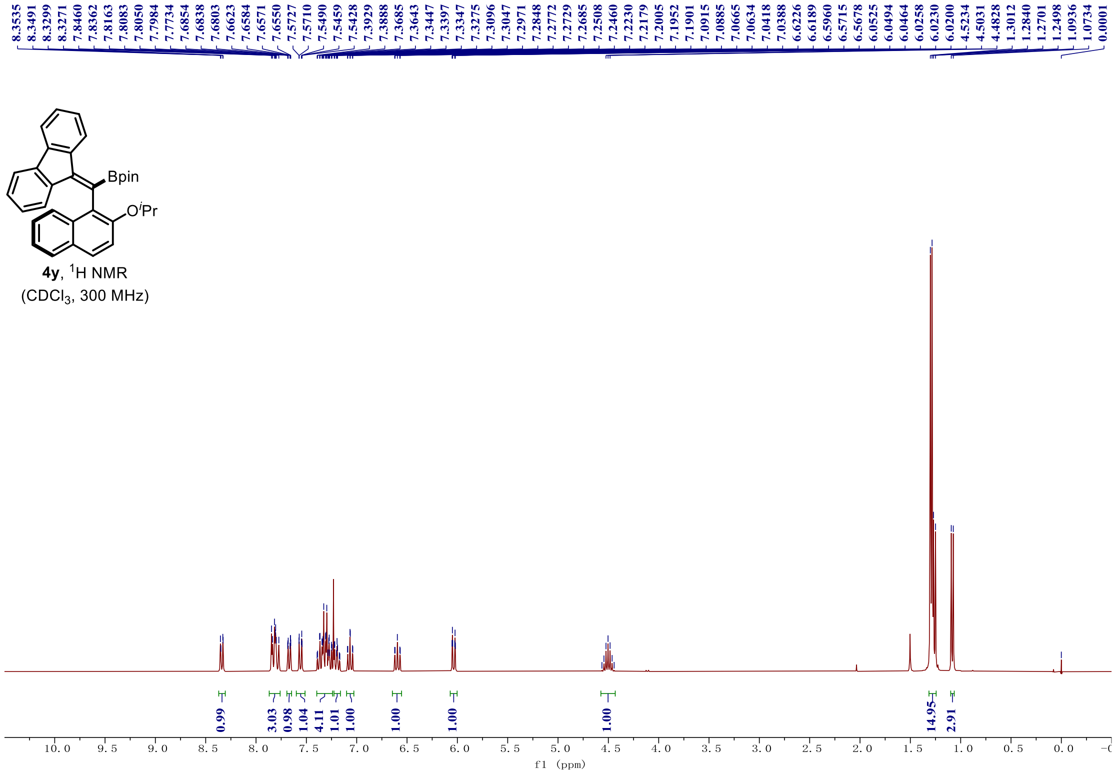

Bpin
O^iPr
4y, ¹H NMR
(CDCl3, 300 MHz)
0.99
3.03
0.98
1.04
4.11
1.01
1.00
1.00
1.00
1.00
14.95
2.91
f1 (ppm)

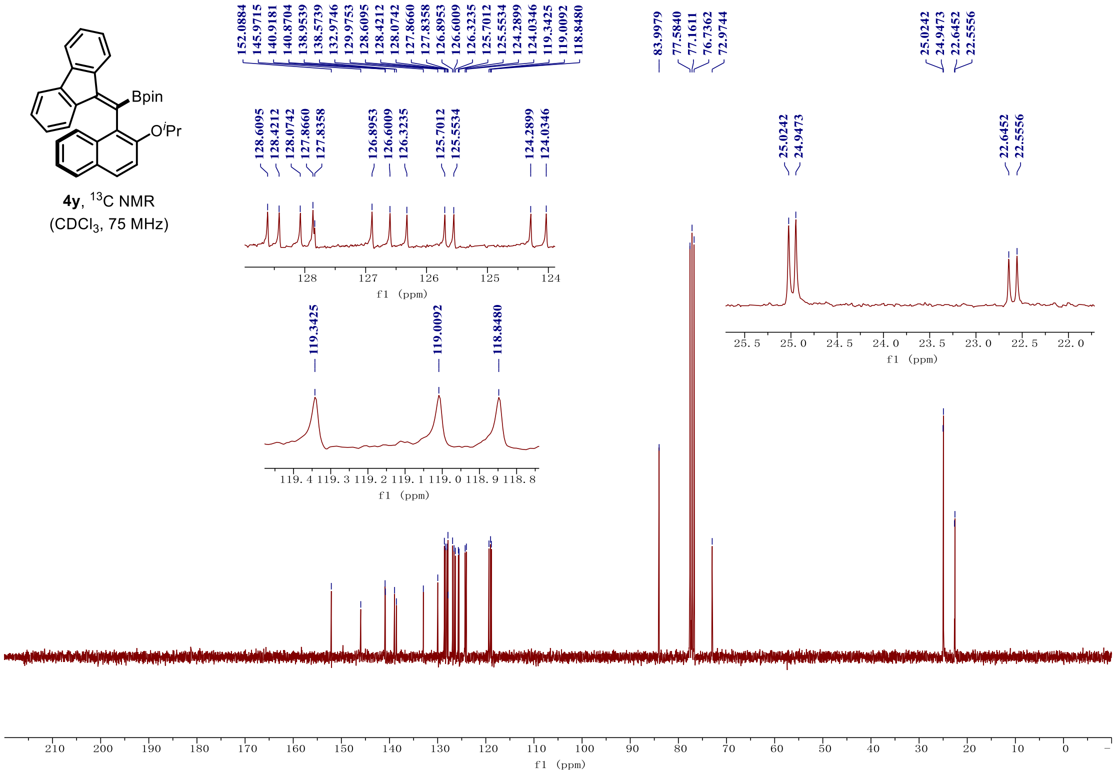

Bpin
O^iPr
4y, ¹³C NMR
(CDCl3, 75 MHz)
152.0884
145.9715
140.9181
140.8704
138.9539
138.5739
132.9746
129.9753
128.6095
128.4212
128.0742
127.8660
127.8358
126.8953
126.6009
126.3235
125.7012
125.5534
124.2899
124.0346
119.3425
119.0092
118.8480
83.9979
77.5840
77.1611
76.7362
72.9744
25.0242
24.9473
22.6452
22.5556
f1 (ppm)

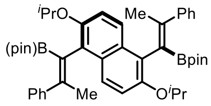

4z, ^{1}H NMR
($CDCl_3$, 300 MHz)

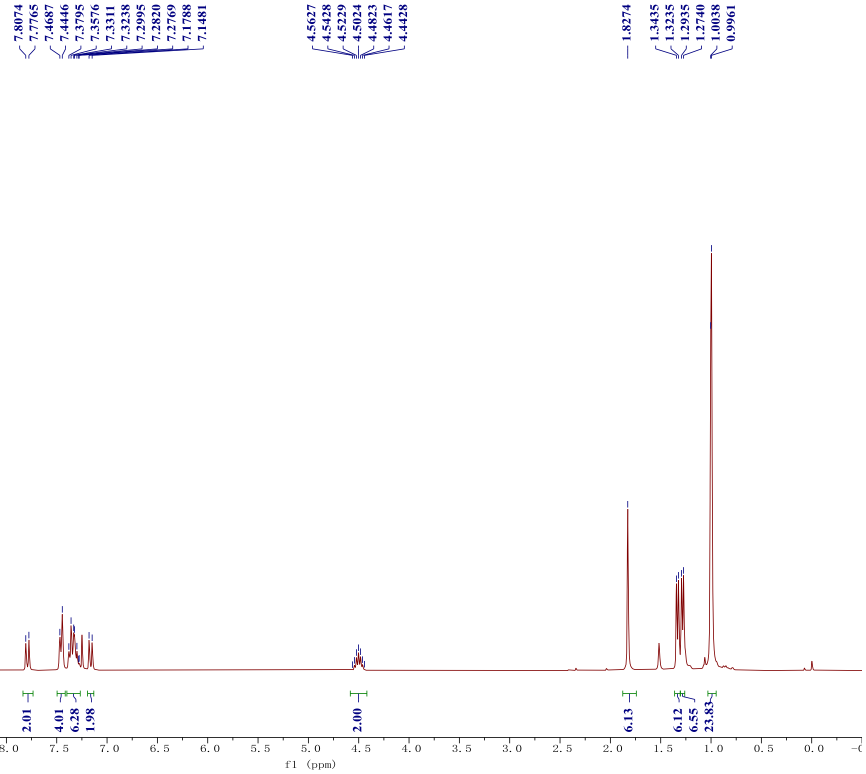

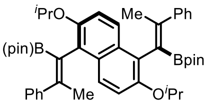

4z, ^{13}C NMR
($CDCl_3$, 75 MHz)

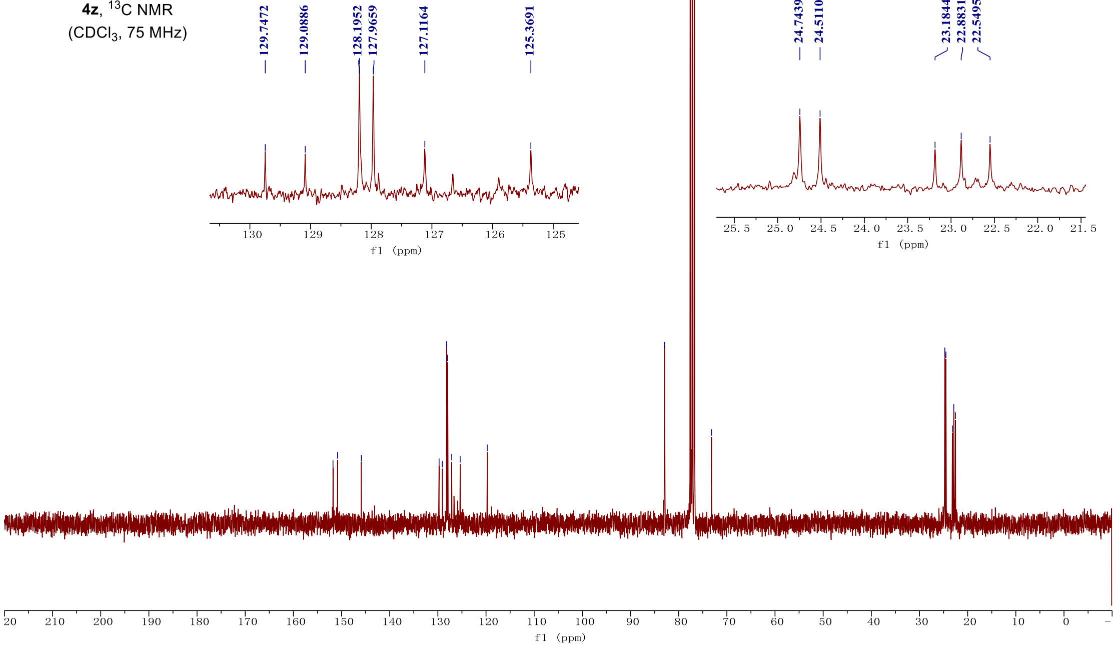

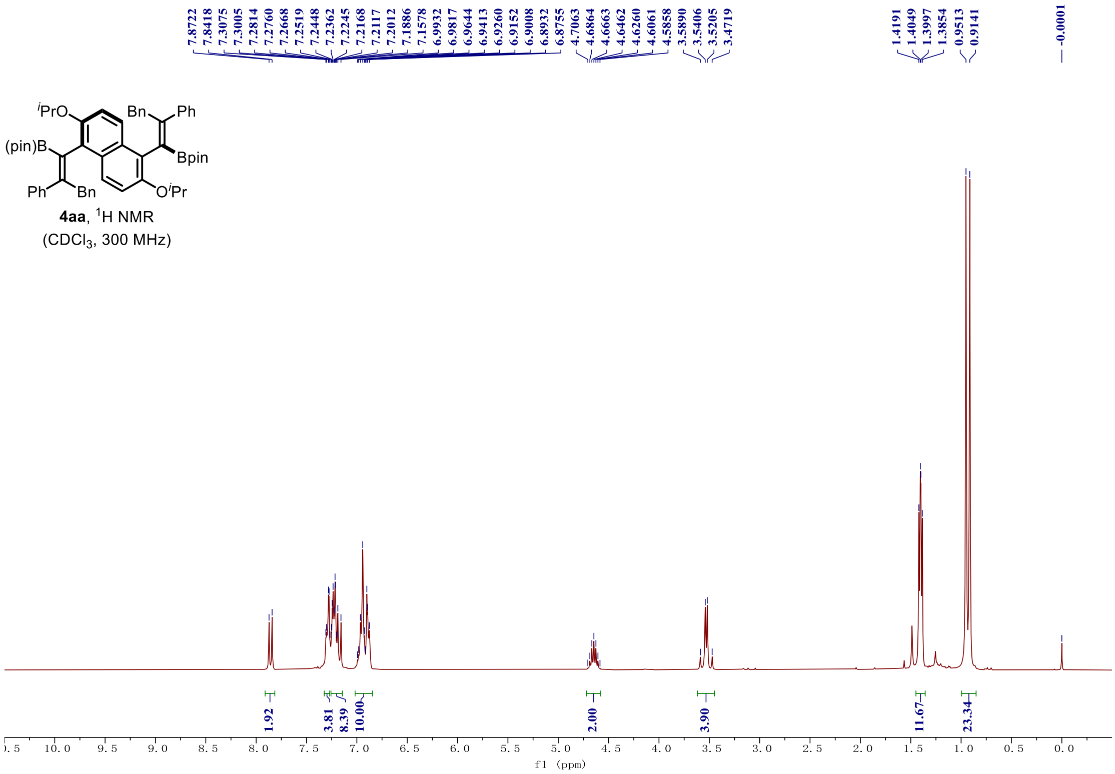
7.8722 7.8418 7.3075 7.3005 7.2814 7.2760 7.2668 7.2519 7.2448 7.2362 7.2245 7.2168 7.2117 7.2012 7.1886 7.1578 6.9932 6.9817 6.9644 6.9413 6.9260 6.9152 6.9008 6.8932 6.8755 4.7063 4.6864 4.6663 4.6462 4.6260 4.6061 4.5858 3.5890 3.5406 3.5205 3.4719
1.4191 1.4049 1.3997 1.3854 0.9513 0.9141
-0.0001
^{i}PrO
Bn
Ph
(pin)B
Bpin
Ph
Bn
O^{i}Pr
4aa, ^{1}H NMR
($CDCl_3$, 300 MHz)
1.92
3.81
8.39
10.00
2.00
3.90
11.67
23.34
f1 (ppm)

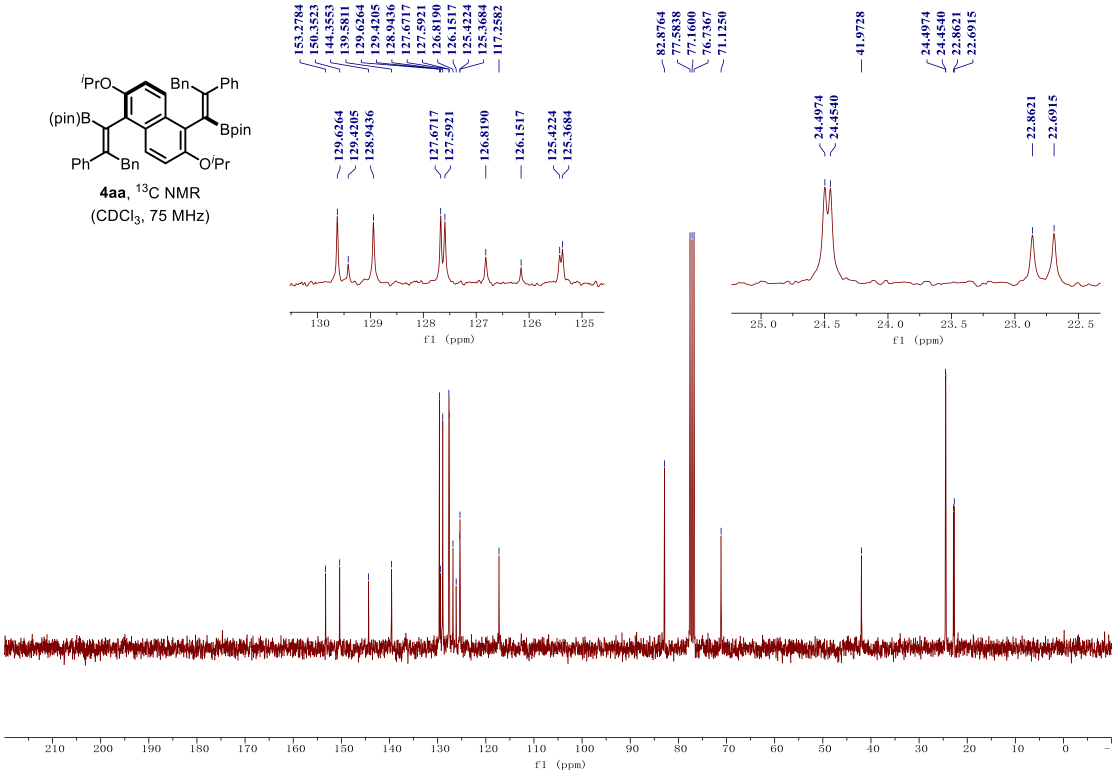
153.2784 150.3523 144.3553 139.5811 129.6264 129.4205 128.9436 127.6717 127.5921 126.8190 126.1517 125.4224 125.3684 117.2582
82.8764 77.5838 77.1600 76.7367 71.1250
41.9728
24.4974 24.4540 22.8621 22.6915
^{i}PrO
Bn
Ph
(pin)B
Bpin
Ph
Bn
O^{i}Pr
4aa, ^{13}C NMR
($CDCl_3$, 75 MHz)
129.6264 129.4205 128.9436
127.6717 127.5921
126.8190
126.1517
125.4224 125.3684
24.4974 24.4540
22.8621 22.6915
f1 (ppm)

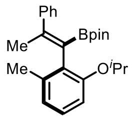

5a, ^{1}H NMR
($CDCl_3$, 300 MHz)

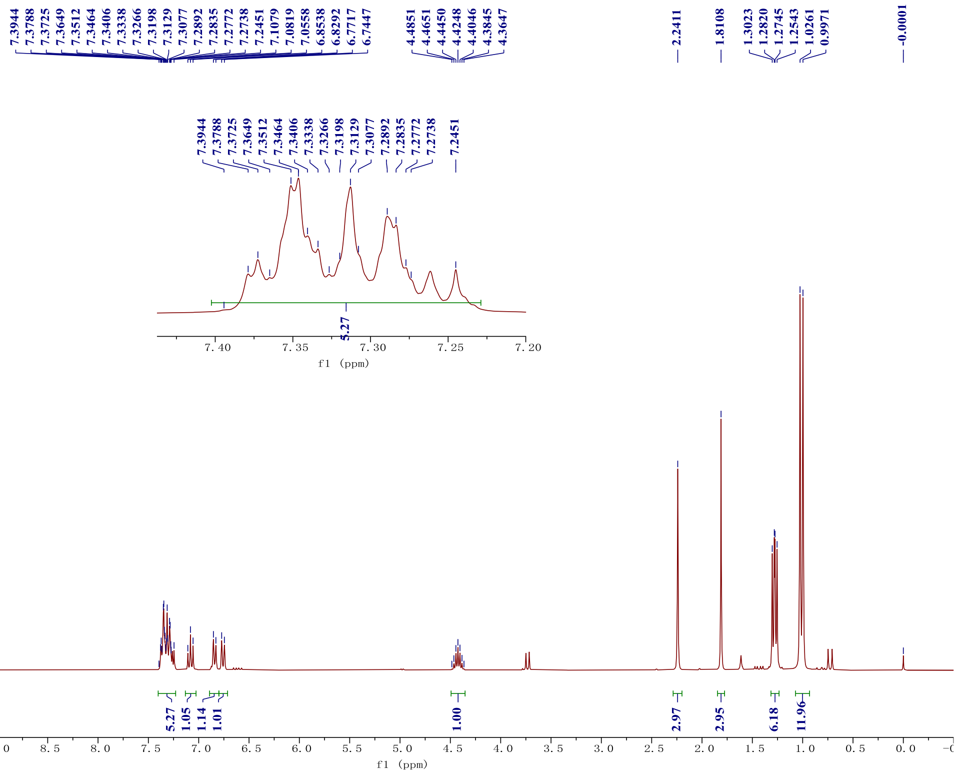

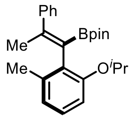

5a, ^{13}C NMR
($CDCl_3$, 75 MHz)

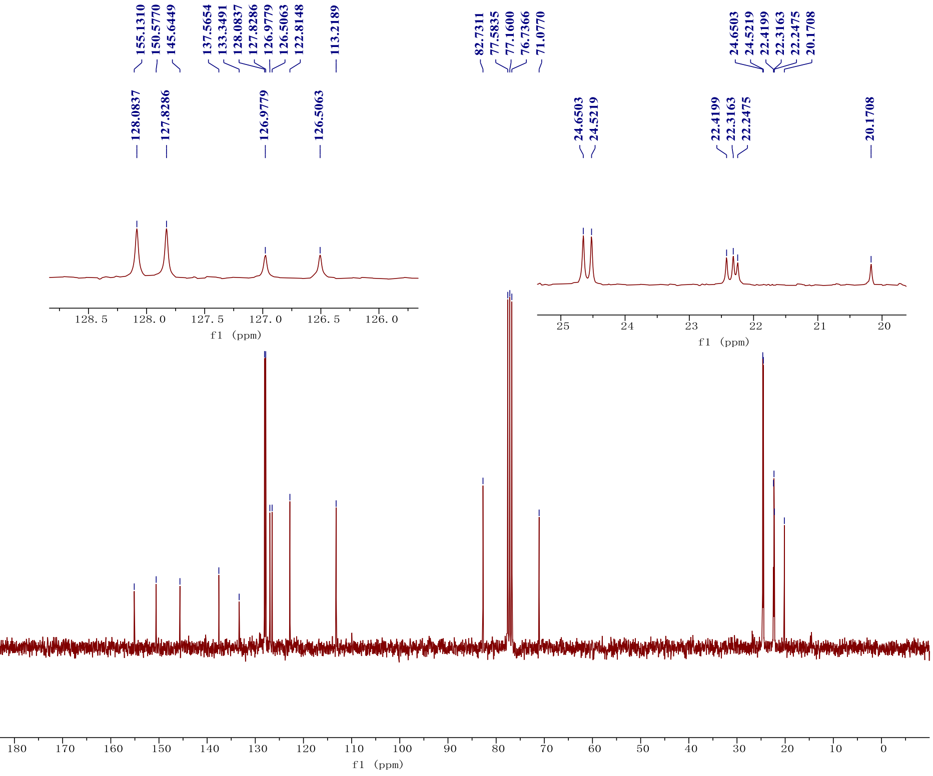

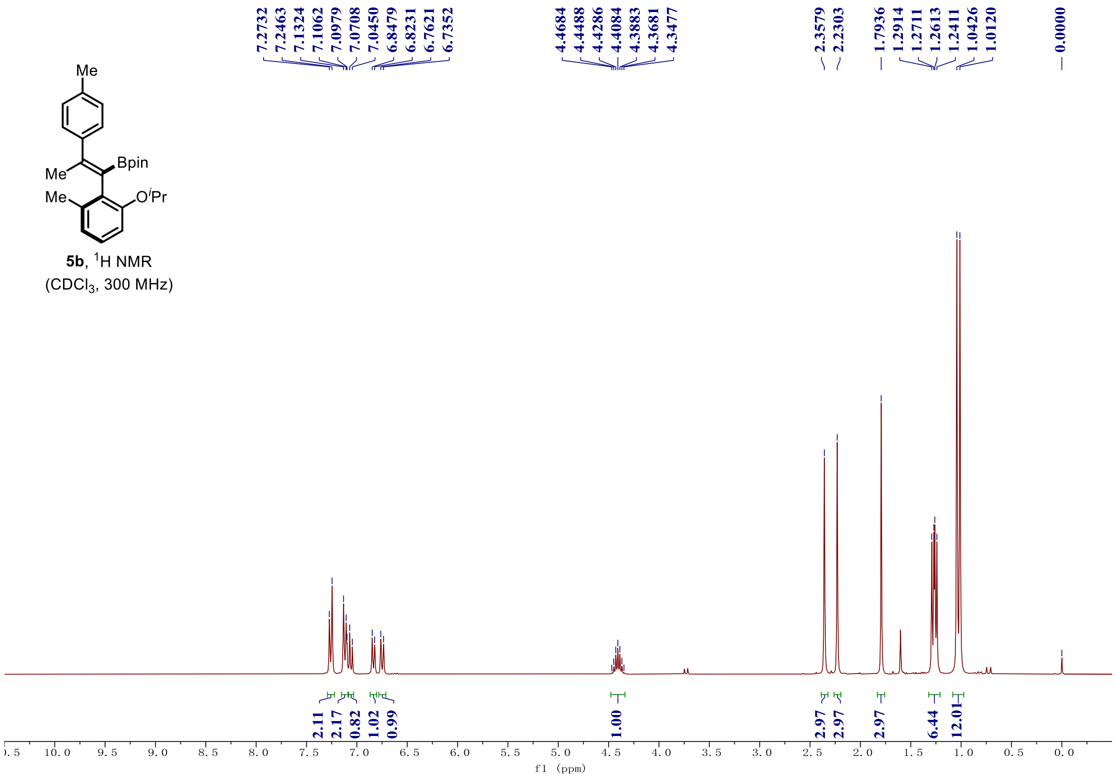

Me
Bpin
Me
Me
O^{i}Pr
5b, ^{1}H NMR
(CDCl$_3$, 300 MHz)
7.2732
7.2463
7.1324
7.1062
7.0979
7.0708
7.0450
6.8479
6.8231
6.7621
6.7352
4.4684
4.4488
4.4286
4.4084
4.3883
4.3681
4.3477
2.3579
2.2303
1.7936
1.2914
1.2711
1.2613
1.2411
1.0426
1.0120
0.0000
2.11
2.17
0.82
1.02
0.99
1.00
2.97
2.97
2.97
6.44
12.01
f1 (ppm)

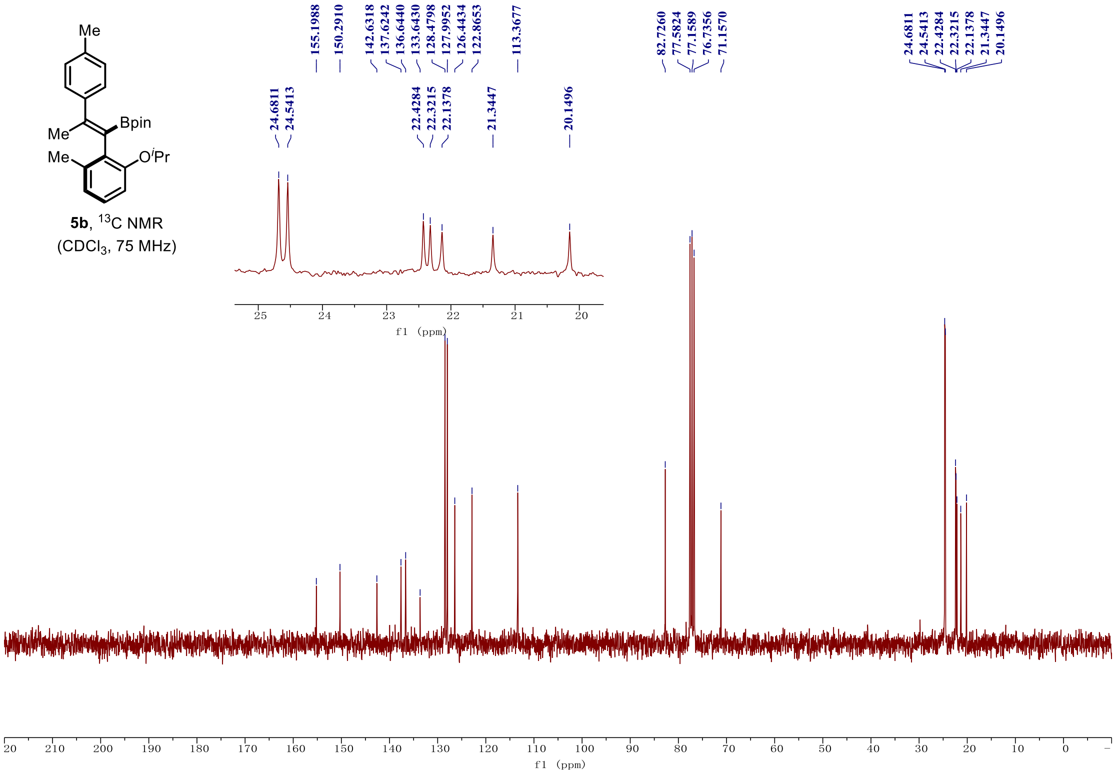

Me
Bpin
Me
Me
O^{i}Pr
5b, ^{13}C NMR
(CDCl$_3$, 75 MHz)
155.1988
150.2910
142.6318
137.6242
136.6440
133.6430
128.4798
127.9952
126.4434
122.8653
113.3677
82.7260
77.5824
77.1589
76.7356
71.1570
24.6811
24.5413
22.4284
22.3215
22.1378
21.3447
20.1496
f1 (ppm)

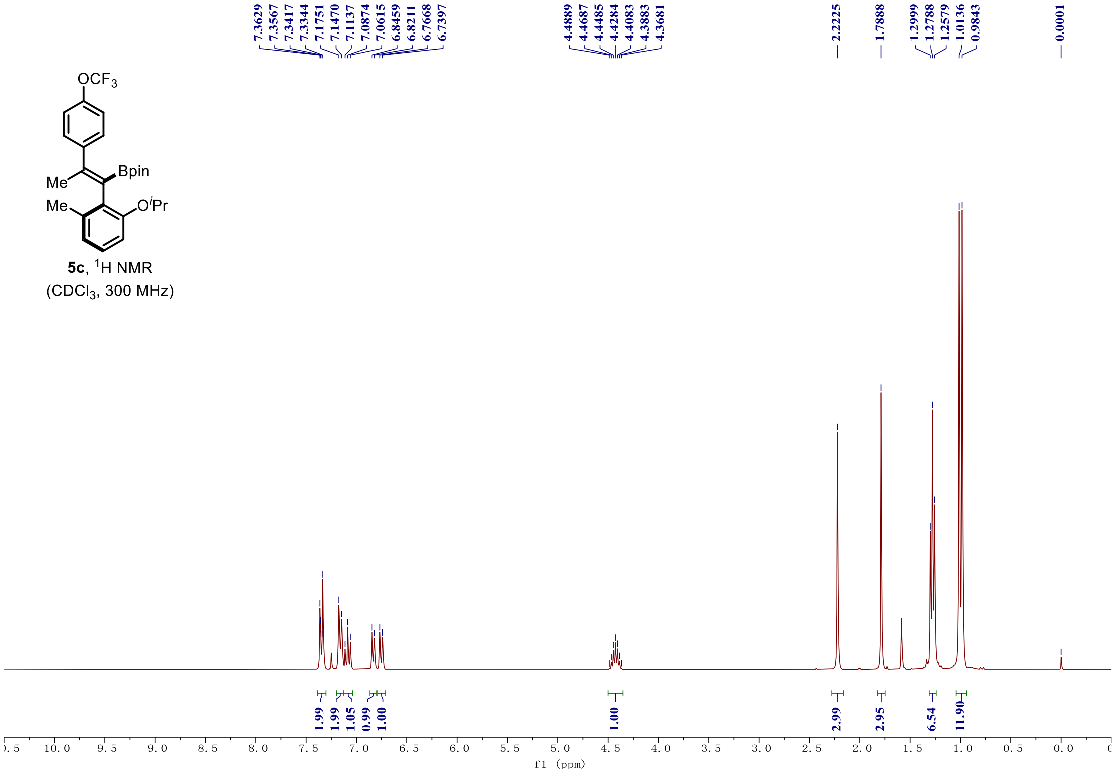

5c, ^{1}H NMR (CDCl$_3$, 300 MHz)

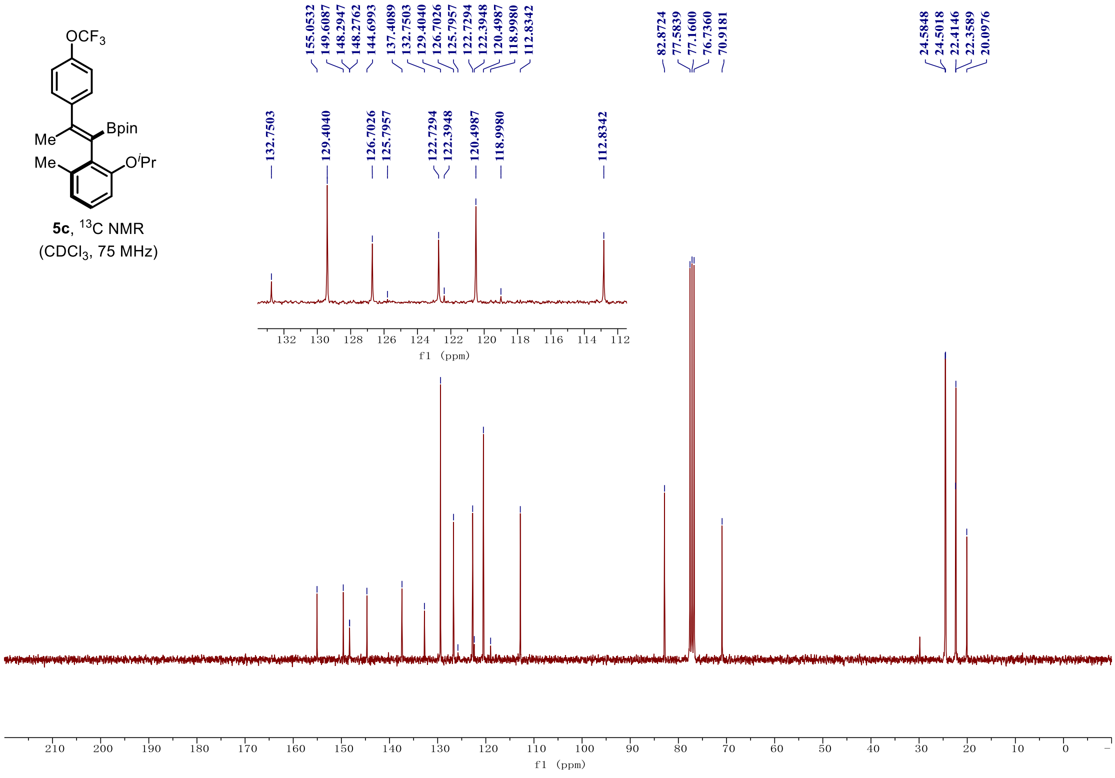

5c, ^{13}C NMR (CDCl$_3$, 75 MHz)

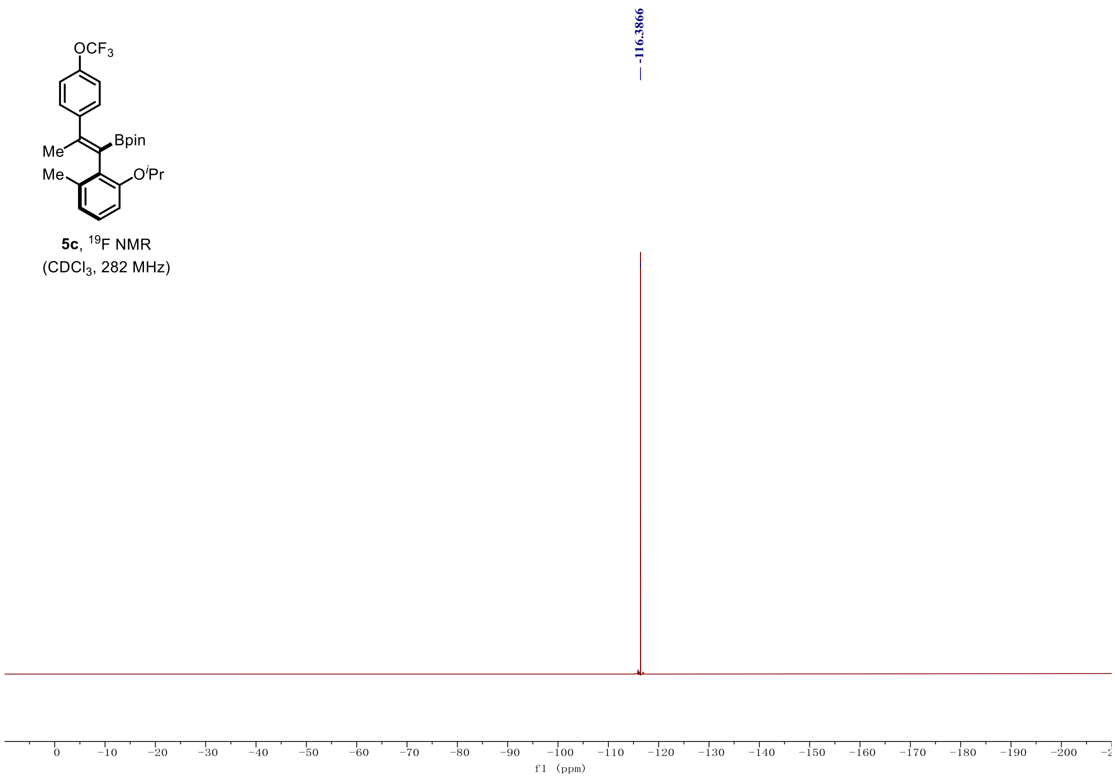

5c, ^{19}F NMR
($CDCl_3$, 282 MHz)

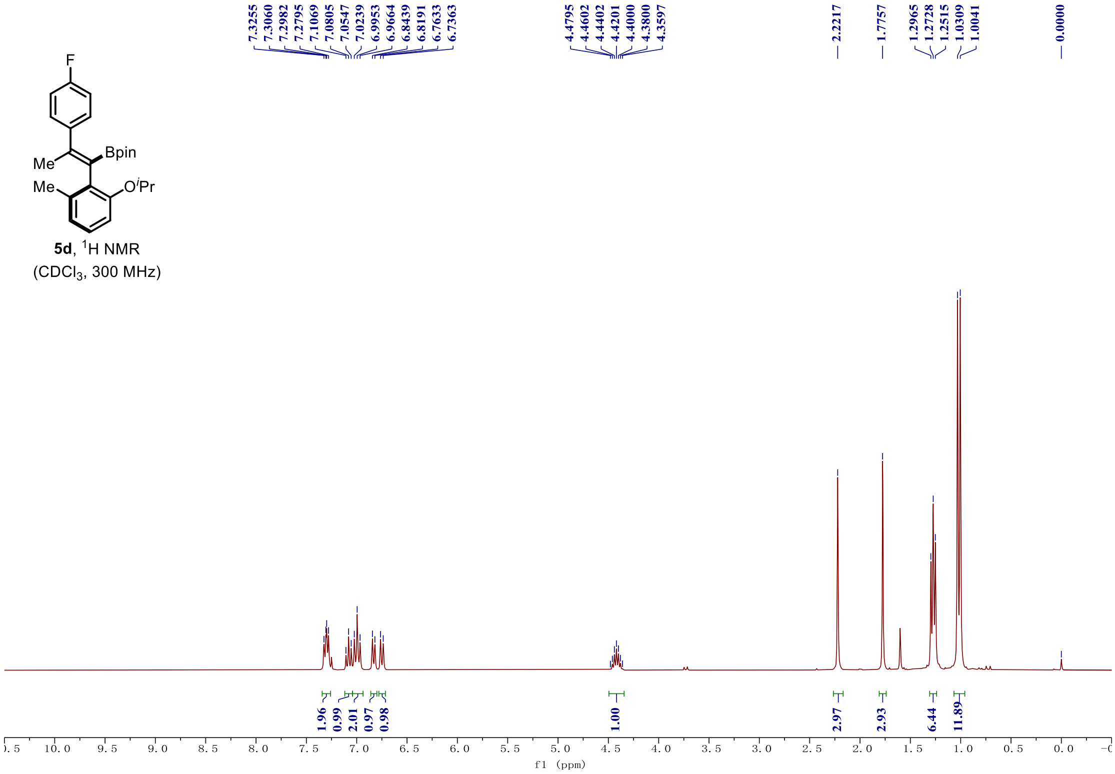

5d, ^{1}H NMR
($CDCl_3$, 300 MHz)

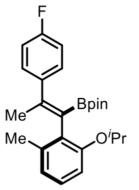

5d, ^{13}C NMR
($CDCl_3$, 75 MHz)

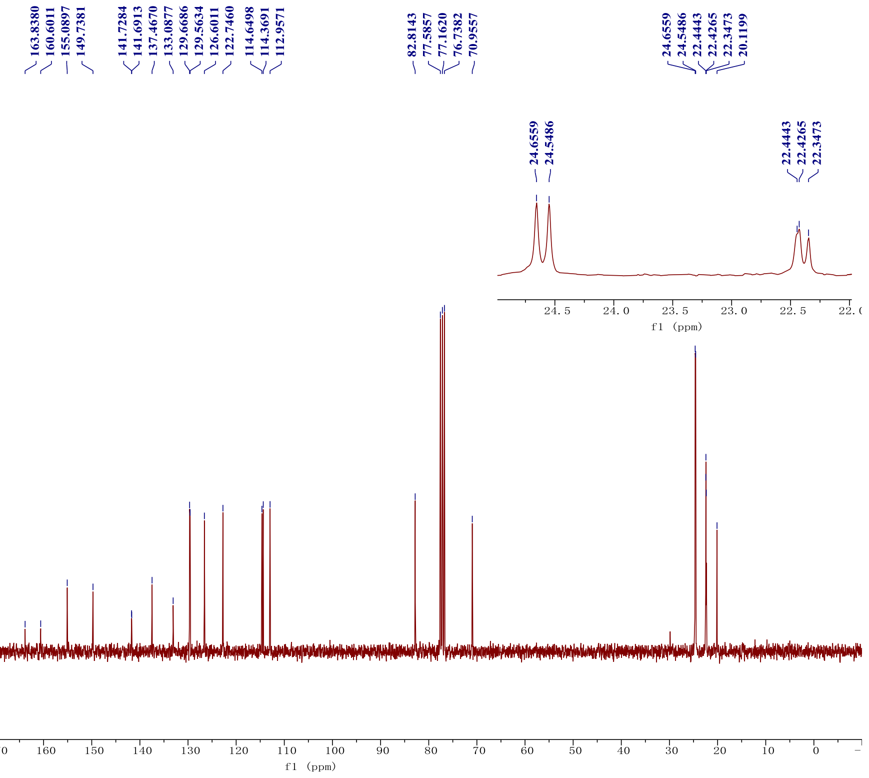

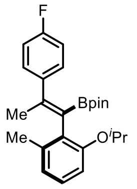

5d, ^{19}F NMR
($CDCl_3$, 282 MHz)

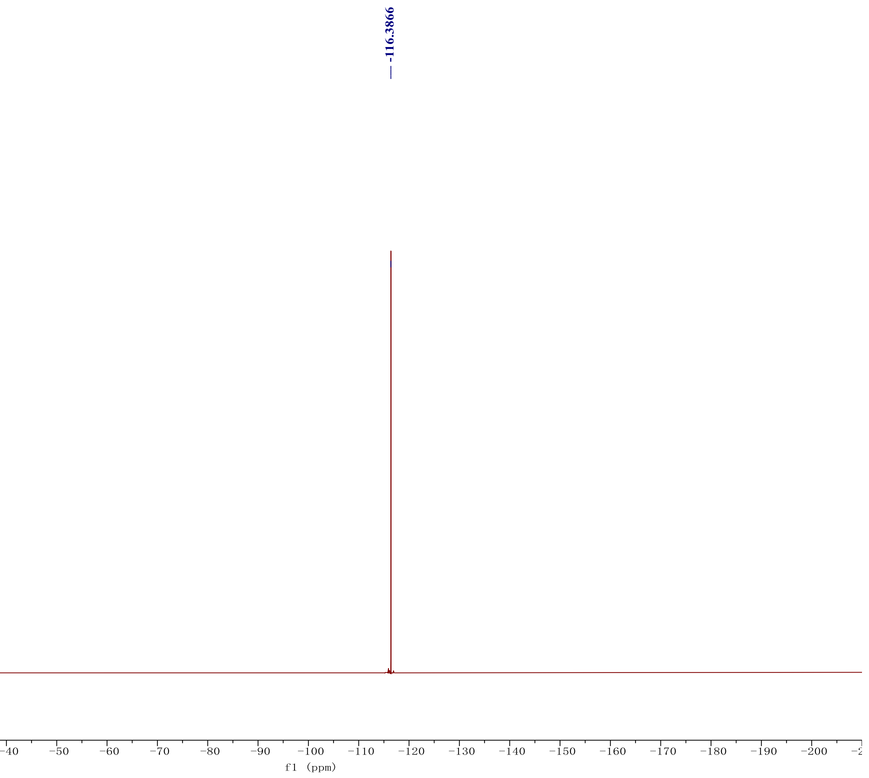

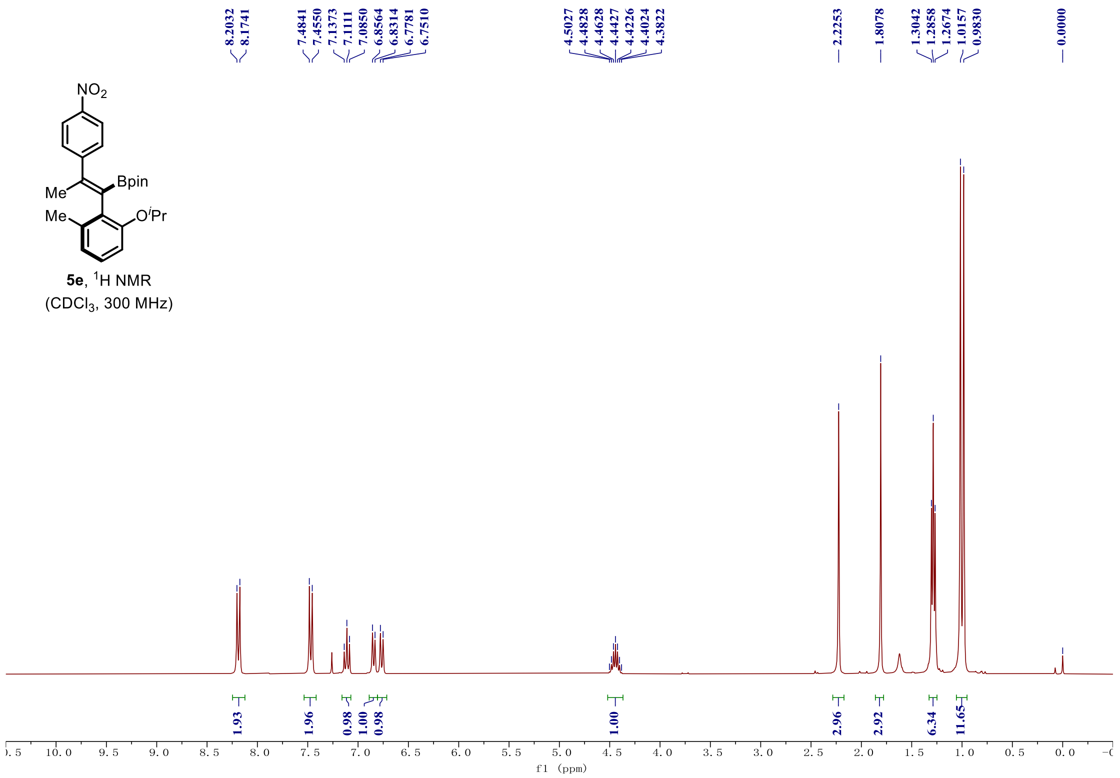

5e, ^{1}H NMR (CDCl$_3$, 300 MHz)

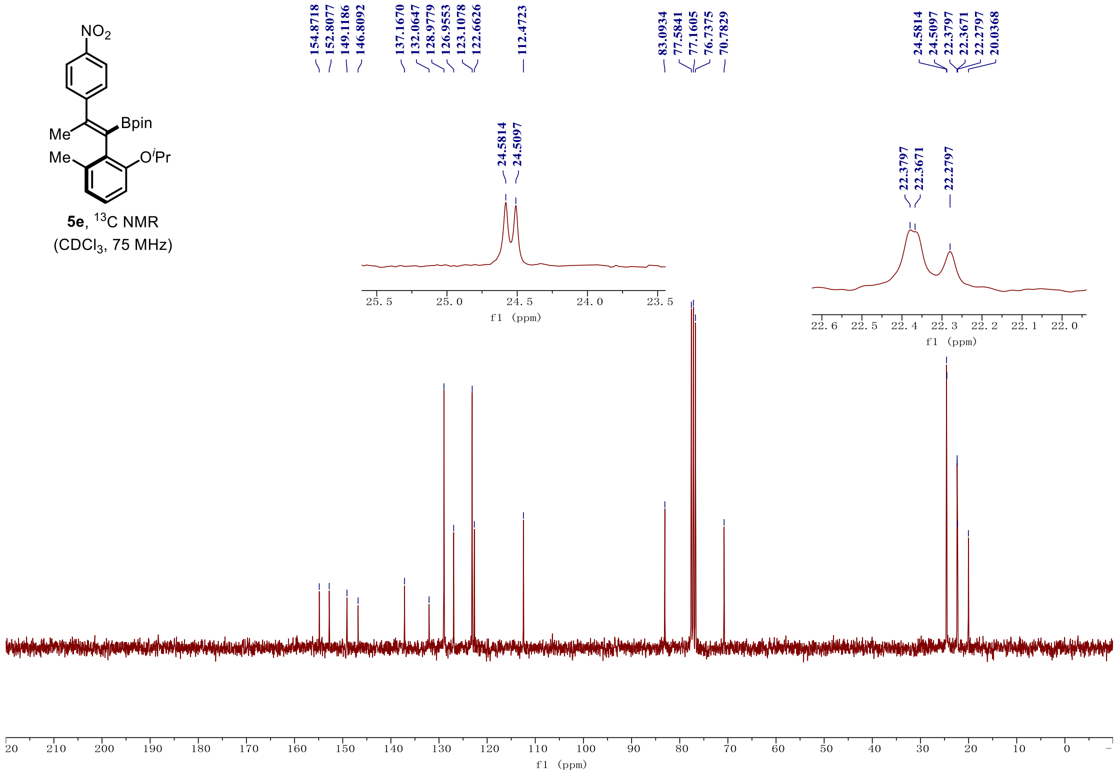

5e, ^{13}C NMR (CDCl$_3$, 75 MHz)

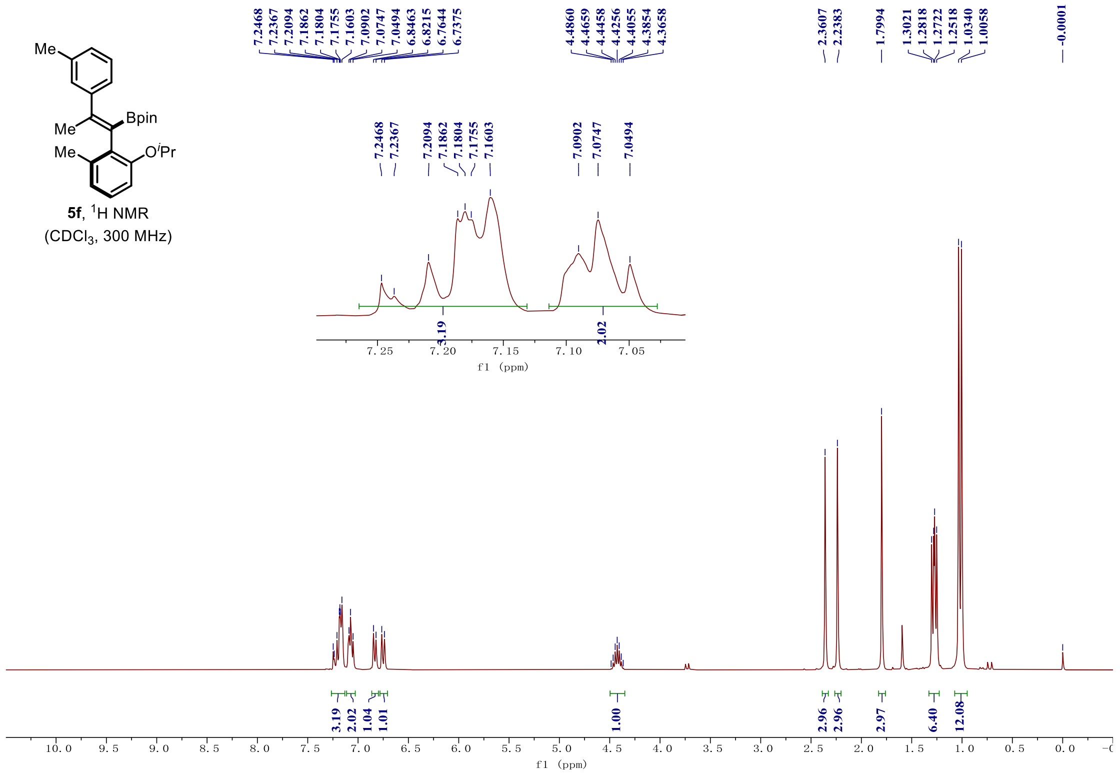
Me
Me
Bpin
Me
O^{i}Pr
5f, ^{1}H NMR
(CDCl$_3$, 300 MHz)
7.2468 7.2367 7.2094 7.1862 7.1804 7.1755 7.1603 7.0902 7.0747 7.0494 6.8463 6.8215 6.7644 6.7375
4.4860 4.4659 4.4458 4.4256 4.4055 4.3854 4.3658
2.3607 2.2383
1.7994
1.3021 1.2818 1.2722 1.2518 1.0340 1.0058
-0.0001
3.19 2.02 1.04 1.01 1.00 2.96 2.96 2.97 6.40 12.08
f1 (ppm)

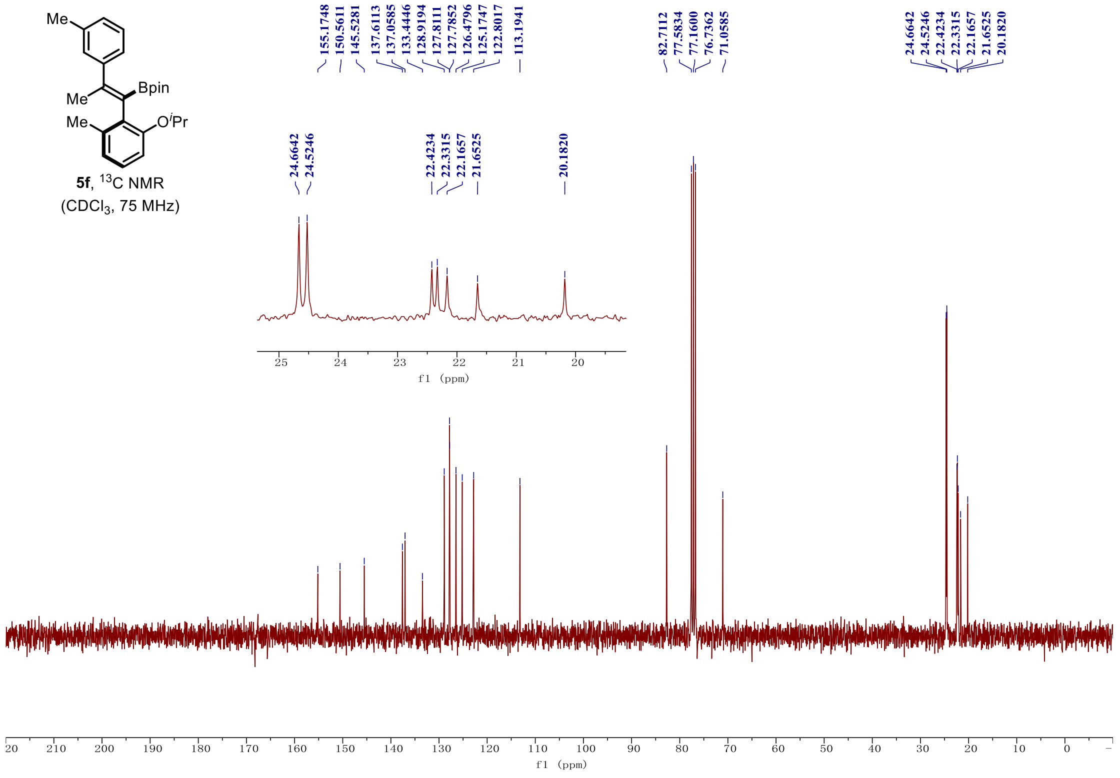
Me
Me
Bpin
Me
O^{i}Pr
5f, ^{13}C NMR
(CDCl$_3$, 75 MHz)
155.1748 150.5611 145.5281 137.6113 137.0585 133.4446 128.9194 127.8111 127.7852 126.4796 125.1747 122.8017 113.1941
82.7112 77.5834 77.1600 76.7362 71.0585
24.6642 24.5246 22.4234 22.3315 22.1657 21.6525 20.1820
f1 (ppm)

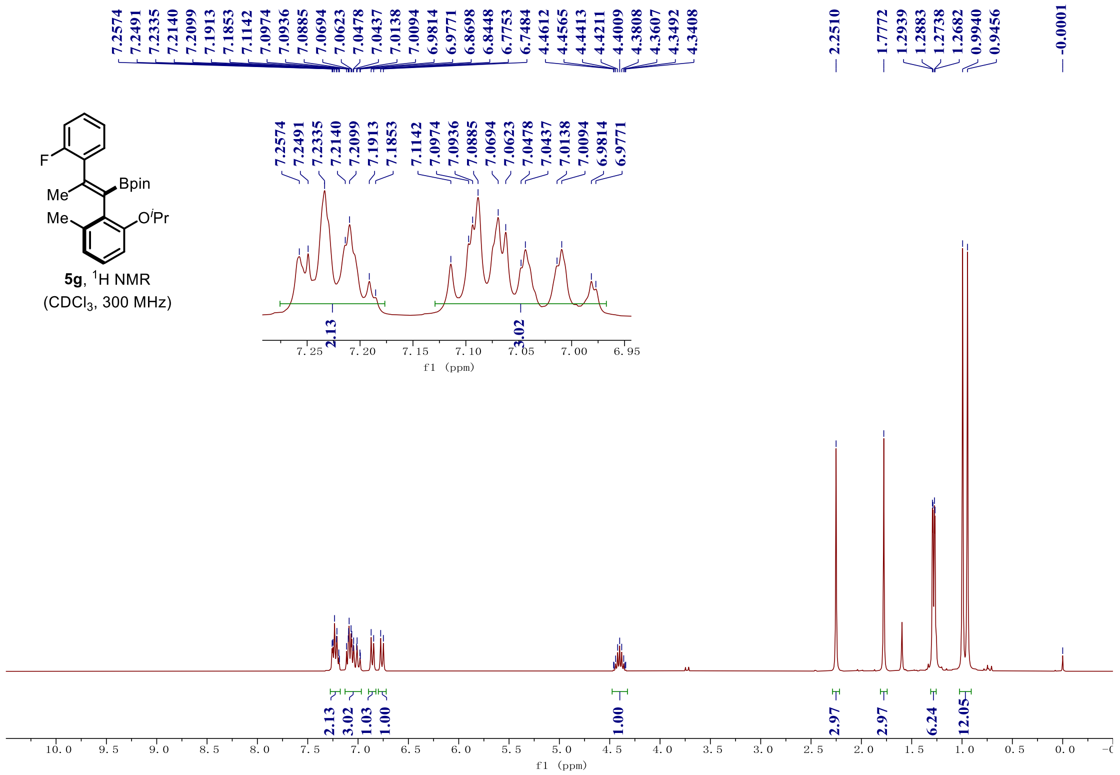
5g, 1H NMR
(CDCl3, 300 MHz)
Bpin
O^iPr
Me
Me
F
7.2574 7.2491 7.2335 7.2140 7.2099 7.1913 7.1853 7.1142 7.0974 7.0936 7.0885 7.0694 7.0623 7.0478 7.0437 7.0138 7.0094 6.9814 6.9771 6.8698 6.8448 6.7753 6.7484 4.4612 4.4565 4.4413 4.4211 4.4009 4.3808 4.3607 4.3492 4.3408
2.2510
1.7772 1.2939 1.2883 1.2738 1.2682 0.9940 0.9456
-0.0001
2.13 3.02 1.03 1.00 1.00 2.97 2.97 6.24 12.05
f1 (ppm)

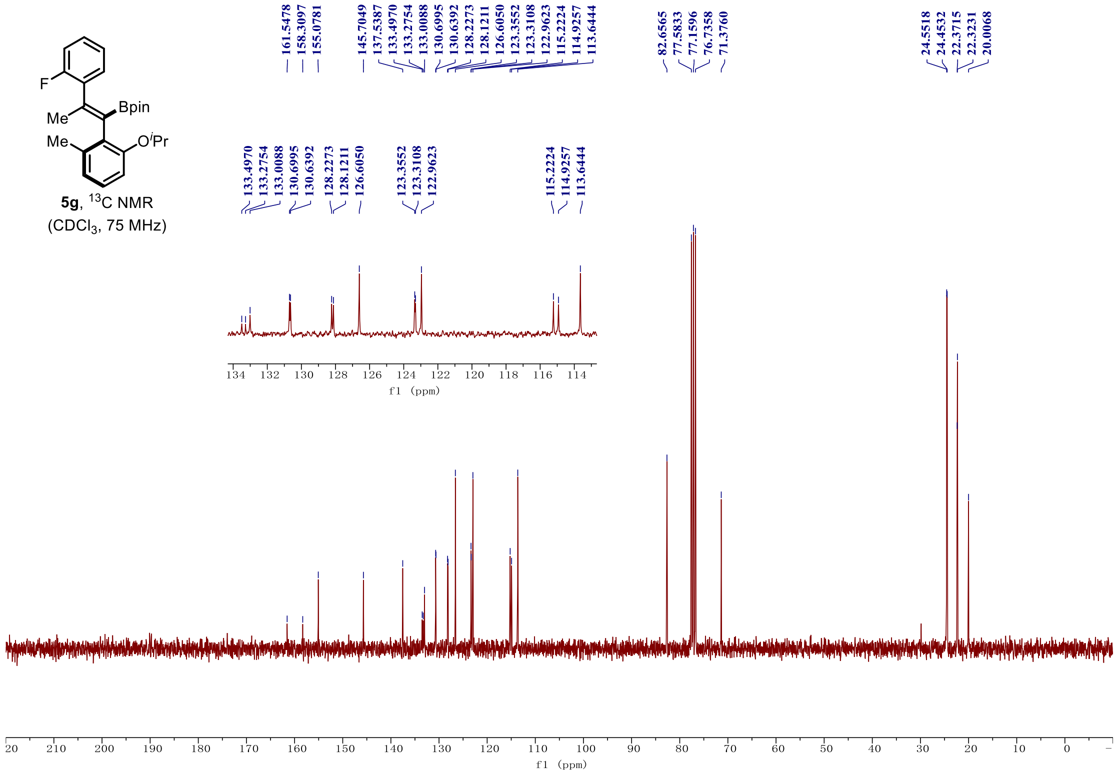
5g, 13C NMR
(CDCl3, 75 MHz)
Bpin
O^iPr
Me
Me
F
161.5478 158.3097 155.0781
145.7049 137.5387 133.4970 133.2754 133.0088 130.6995 130.6392 128.2273 128.1211 126.6050 123.3552 123.3108 122.9623 115.2224 114.9257 113.6444
82.6565 77.5833 77.1596 76.7358 71.3760
24.5518 24.4532 22.3715 22.3231 20.0068
f1 (ppm)

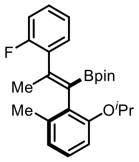

5g, ^{19}F NMR
($CDCl_3$, 282 MHz)

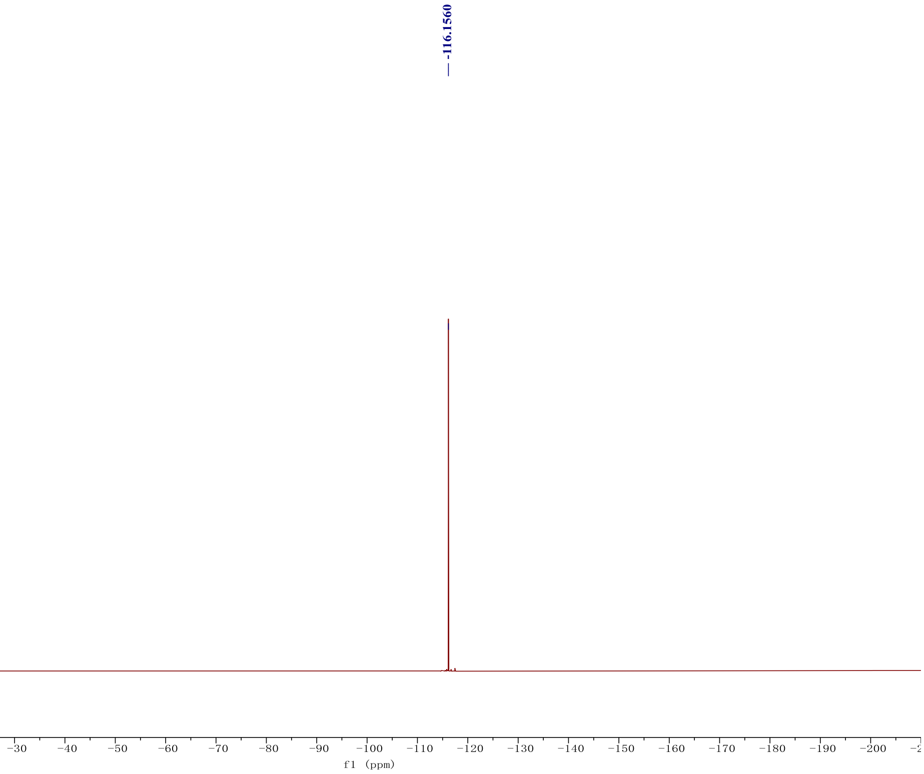

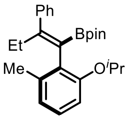

5h, ^{1}H NMR
($CDCl_3$, 300 MHz)

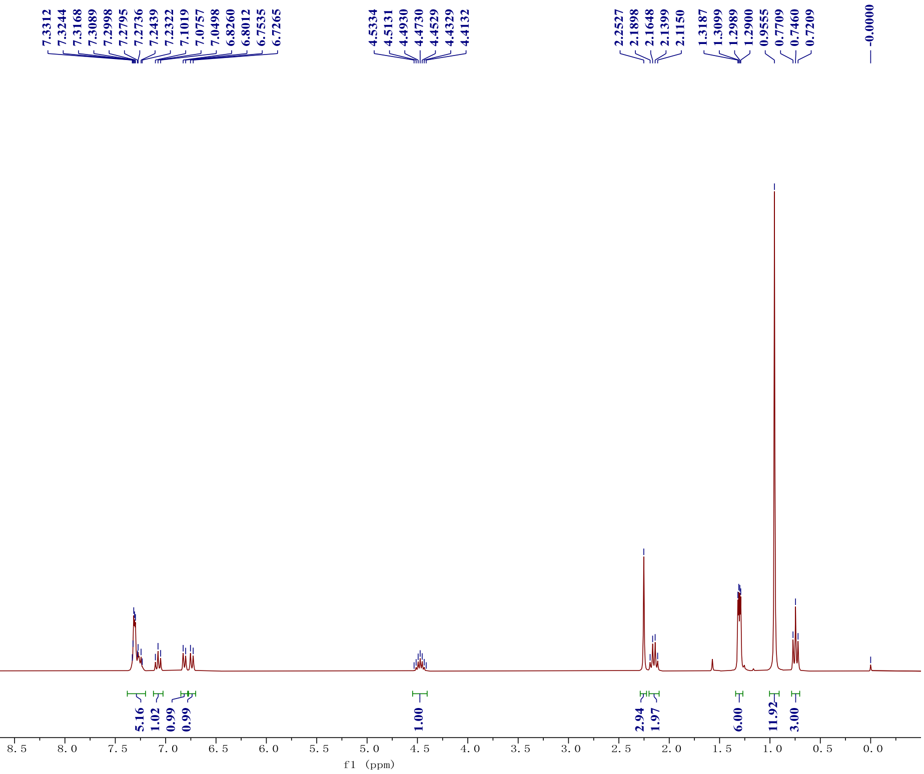

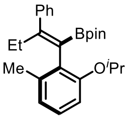

5h, ^{13}C NMR
($CDCl_3$, 75 MHz)

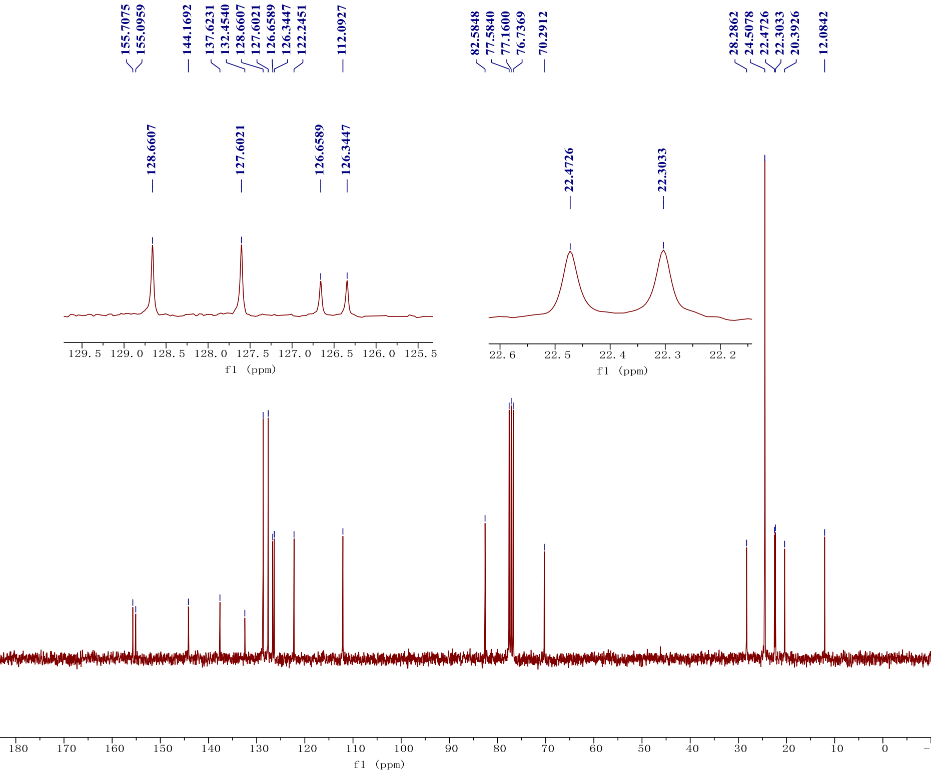

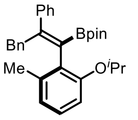

5i, ^{1}H NMR
($CDCl_3$, 300 MHz)

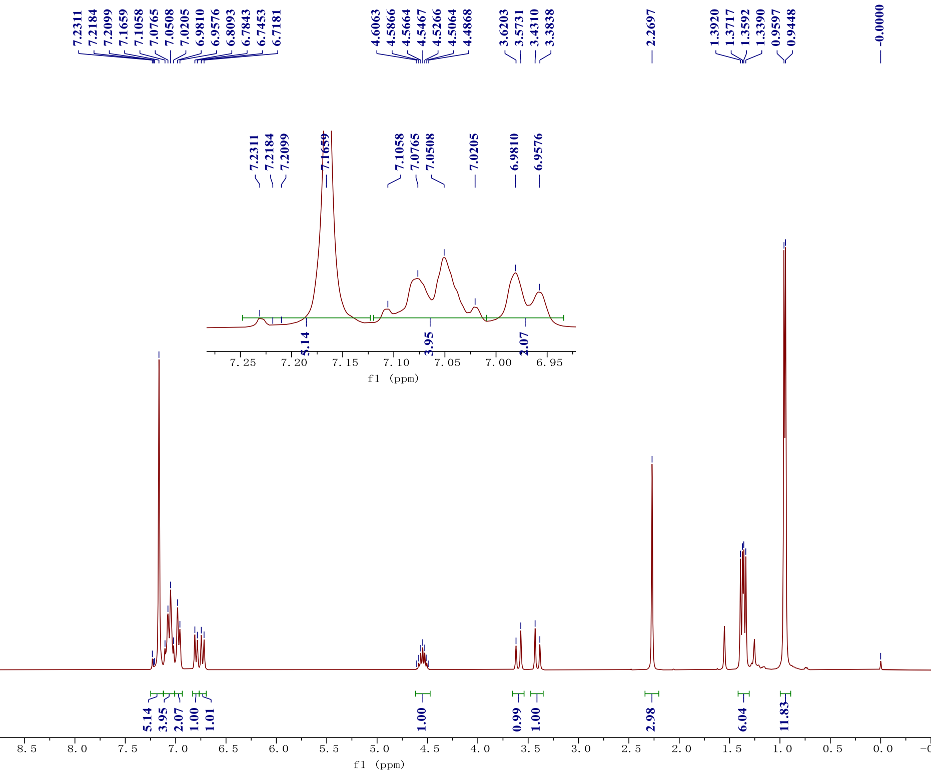

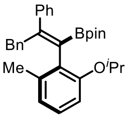

5i, ^{13}C NMR
($CDCl_3$, 75 MHz)

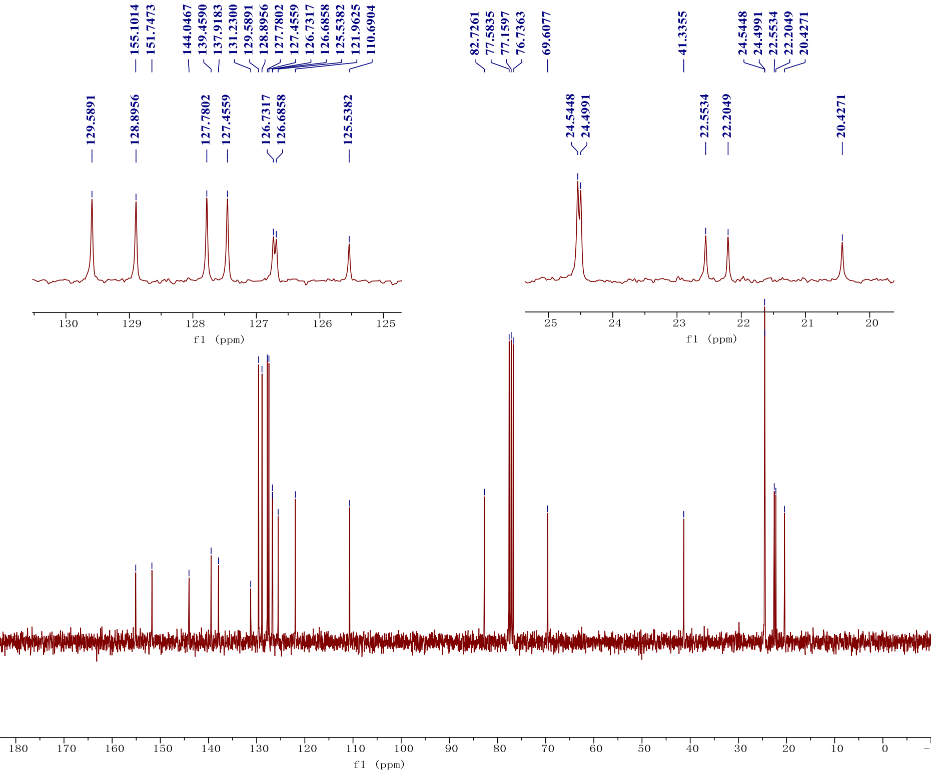

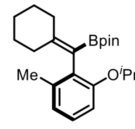

5j, ^{1}H NMR
($CDCl_3$, 300 MHz)

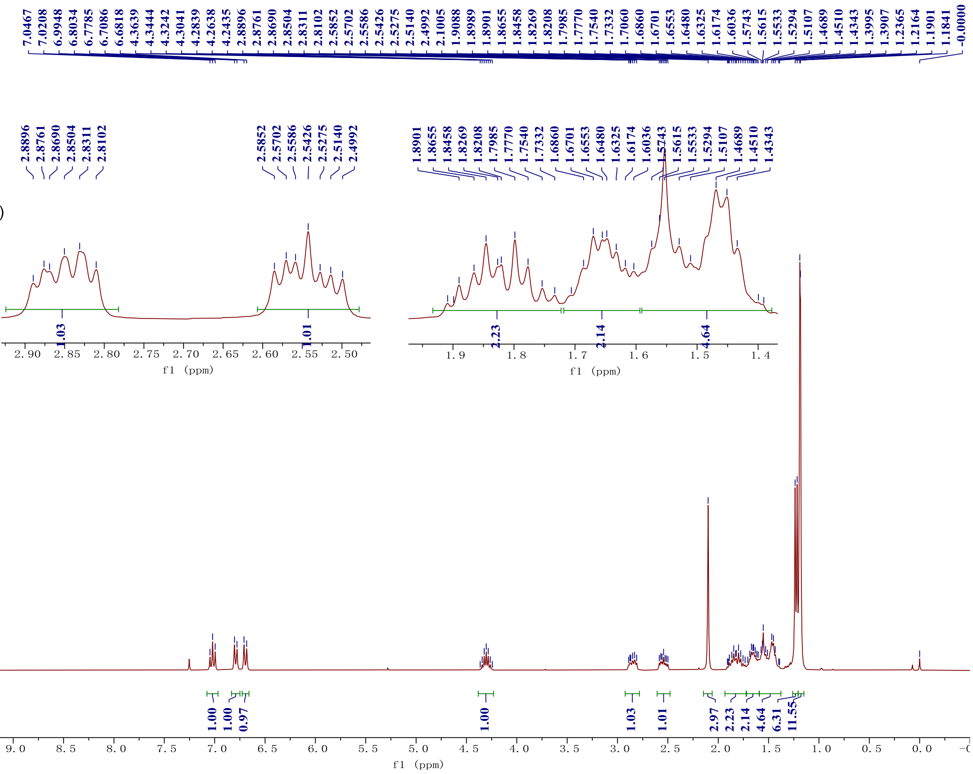

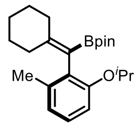

5j, ^{13}C NMR
($CDCl_3$, 75 MHz)

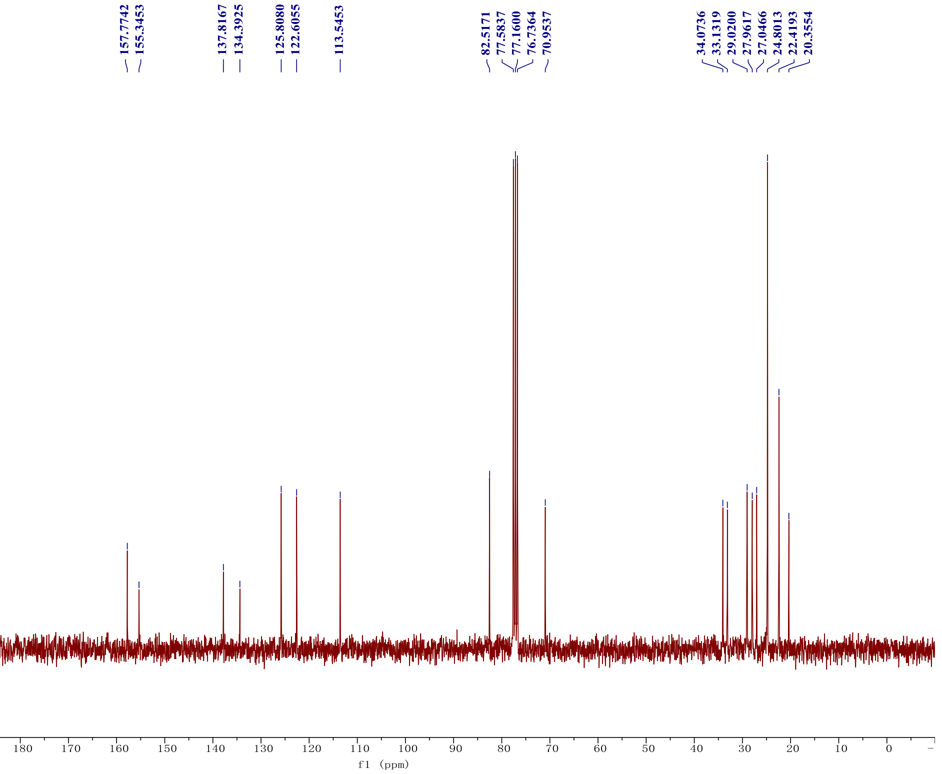

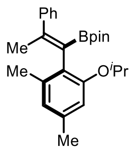

5k, ^{1}H NMR
($CDCl_3$, 300 MHz)

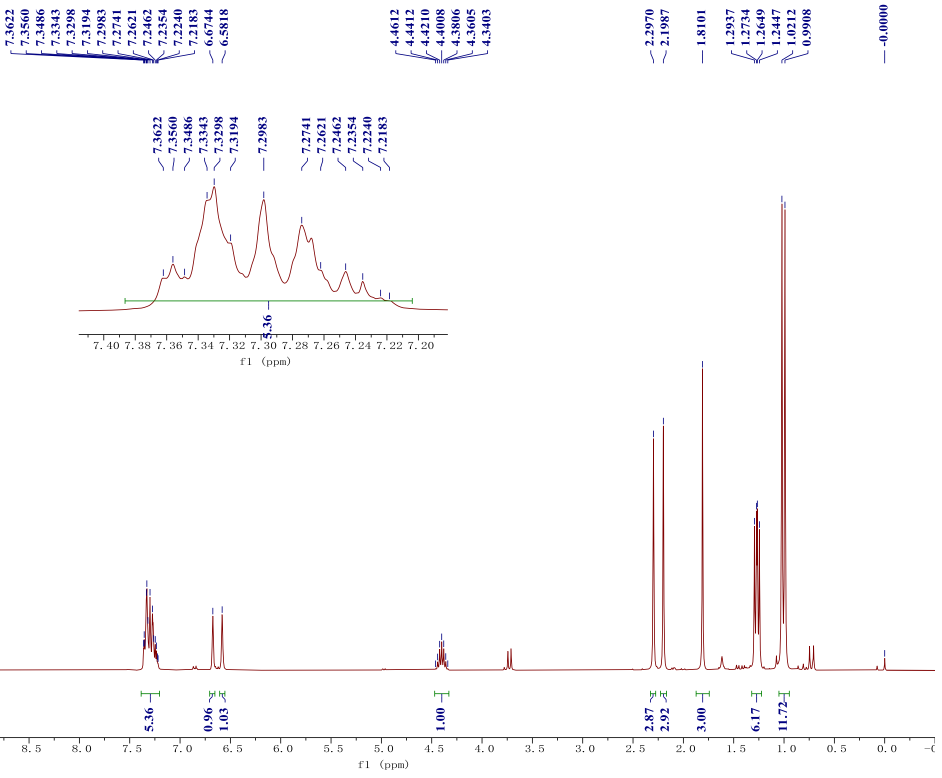

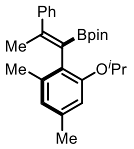

5k, ^{13}C NMR
($CDCl_3$, 75 MHz)

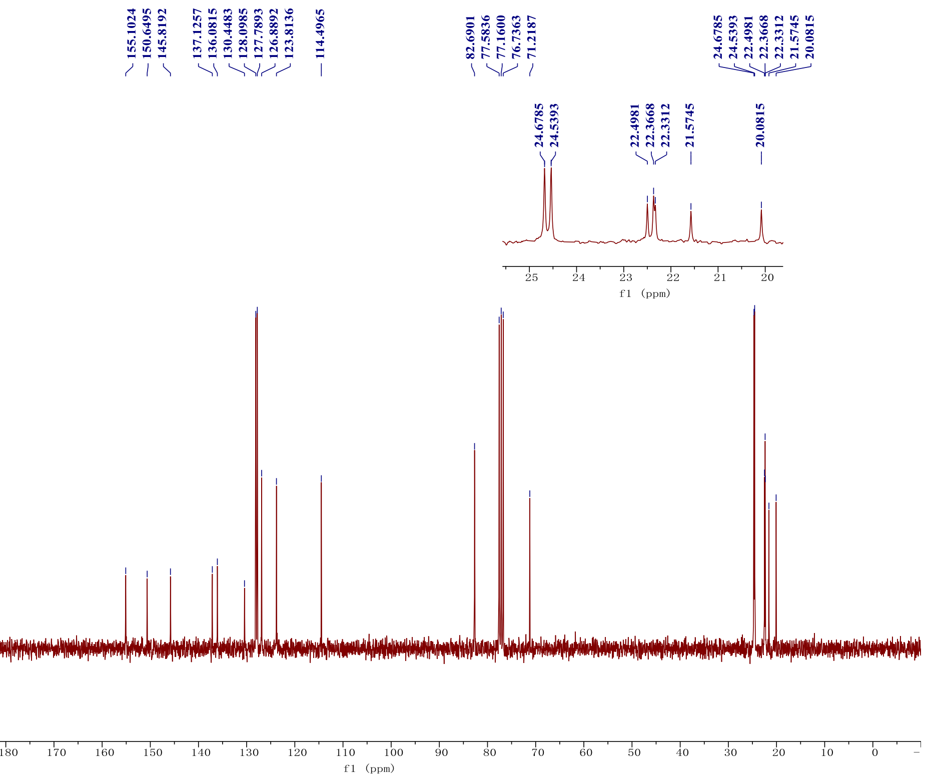

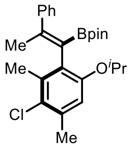

5l, ^{1}H NMR
($CDCl_3$, 300 MHz)

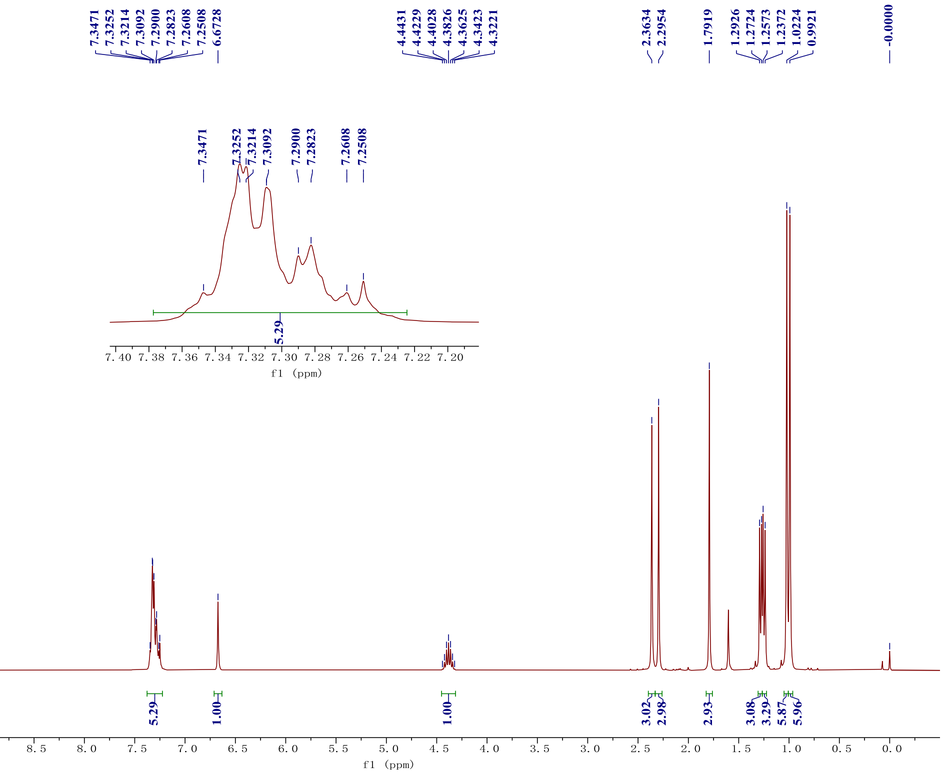

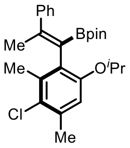

5l, ^{13}C NMR
($CDCl_3$, 75 MHz)

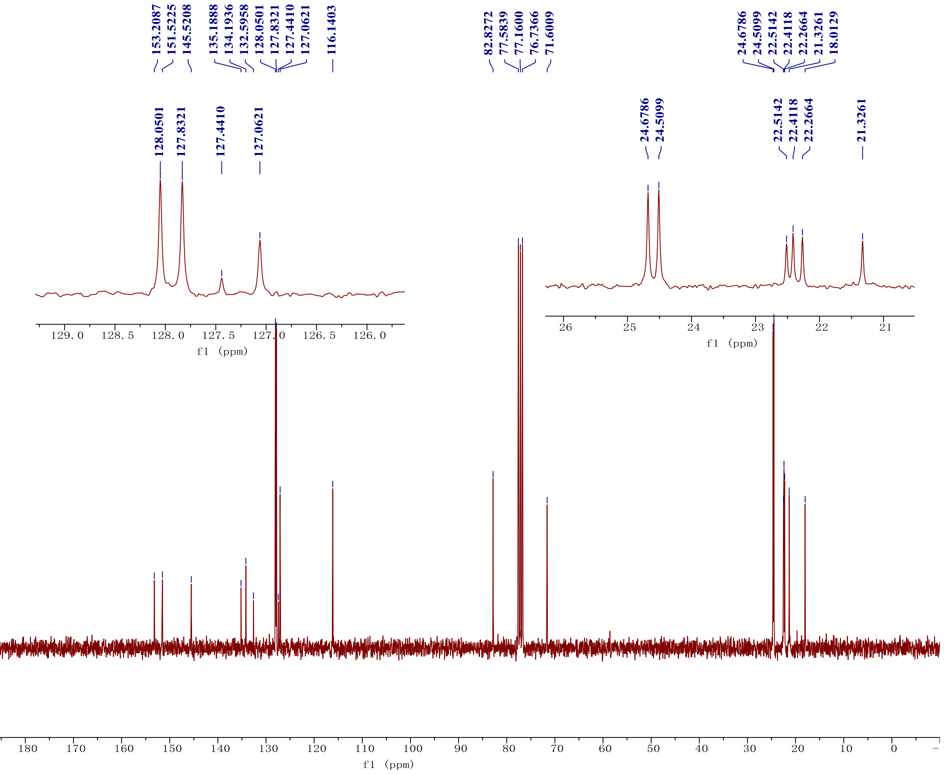

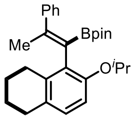

5m, ^{1}H NMR
($CDCl_3$, 300 MHz)

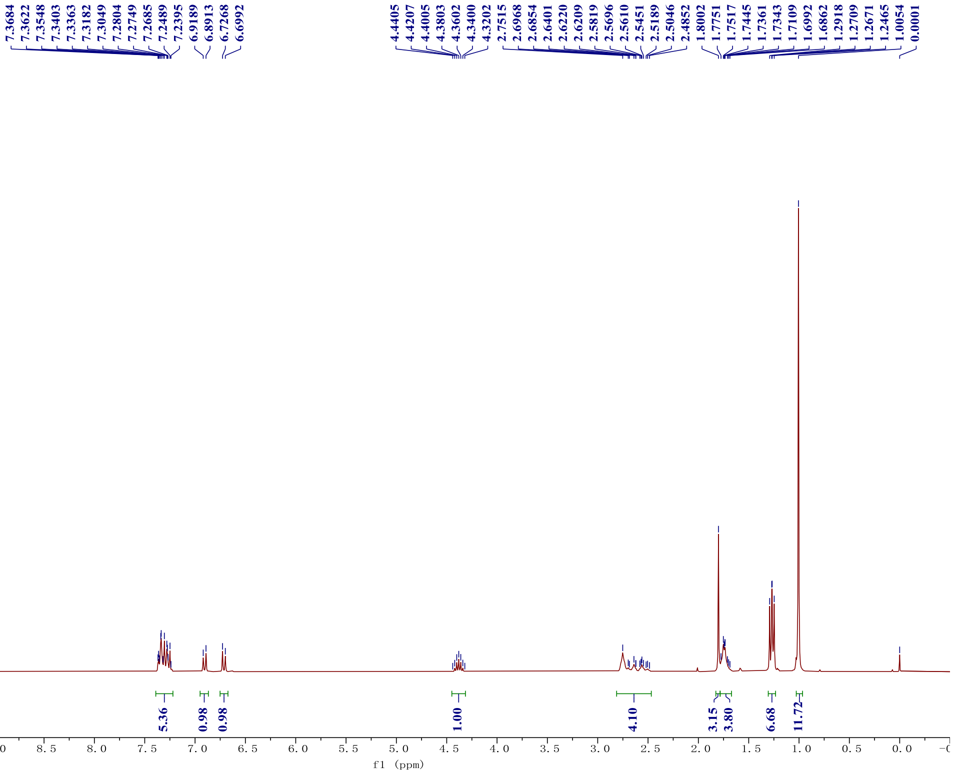

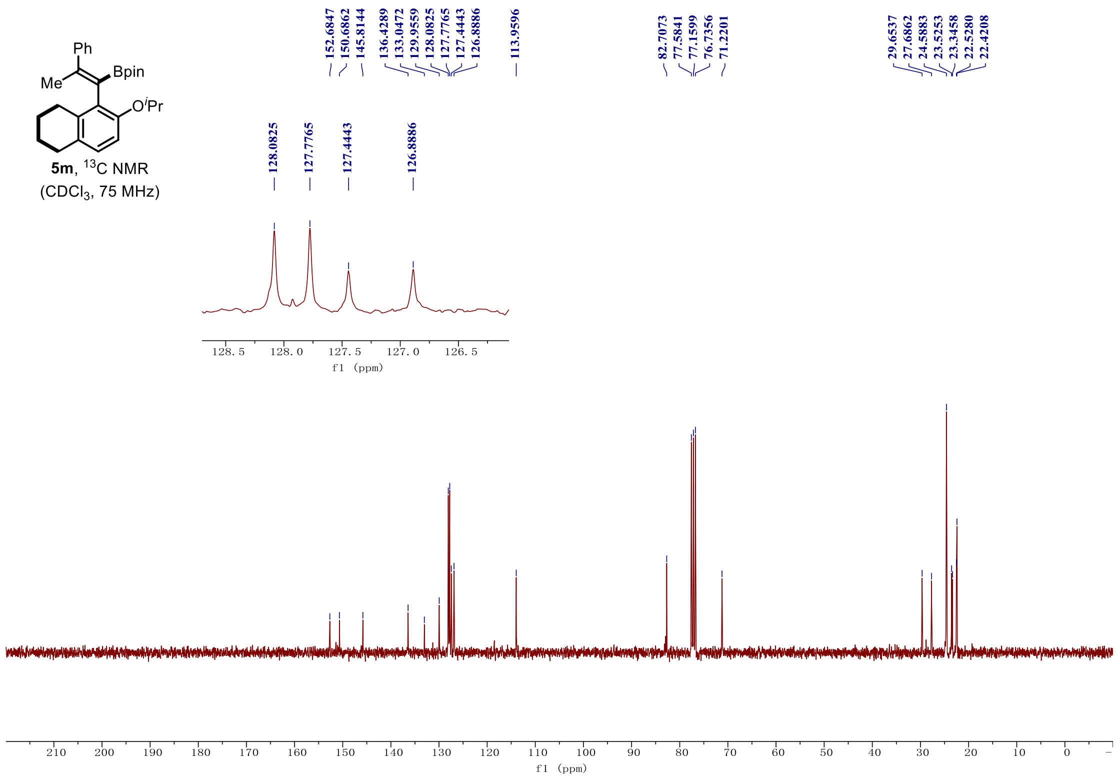

5m, ^{13}C NMR
(CDCl$_3$, 75 MHz)

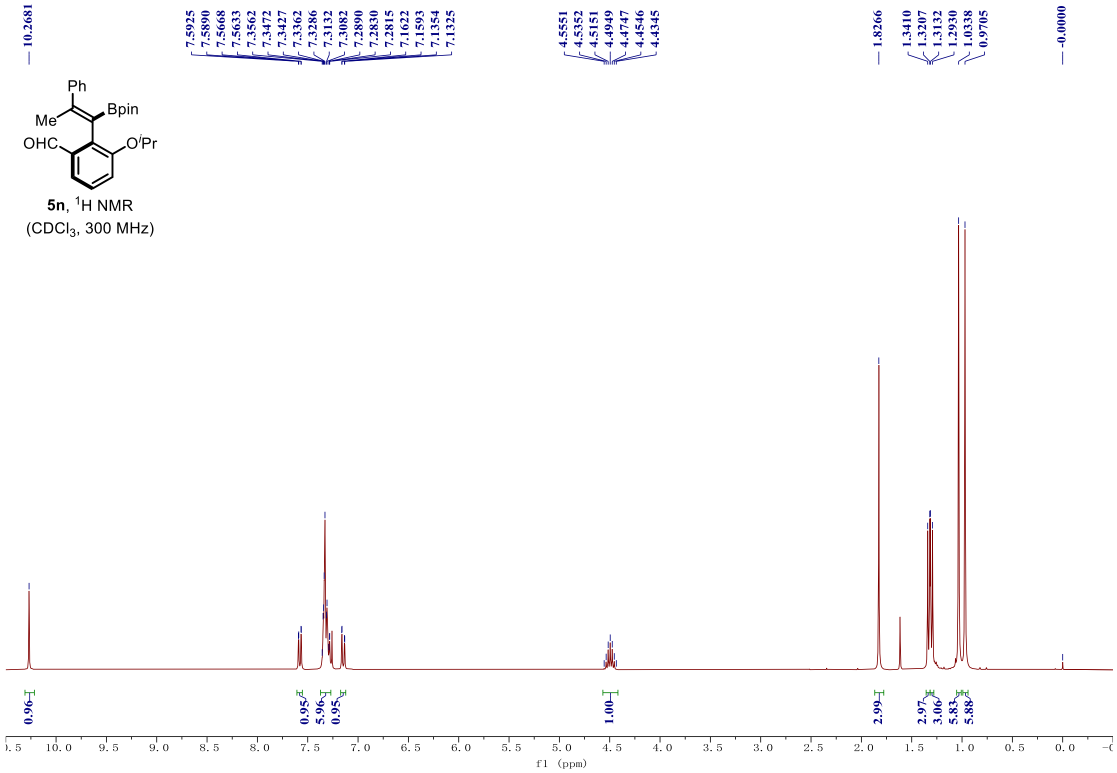

5n, ^{1}H NMR
(CDCl$_3$, 300 MHz)

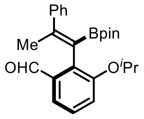

5n, ^{13}C NMR
($CDCl_3$, 75 MHz)

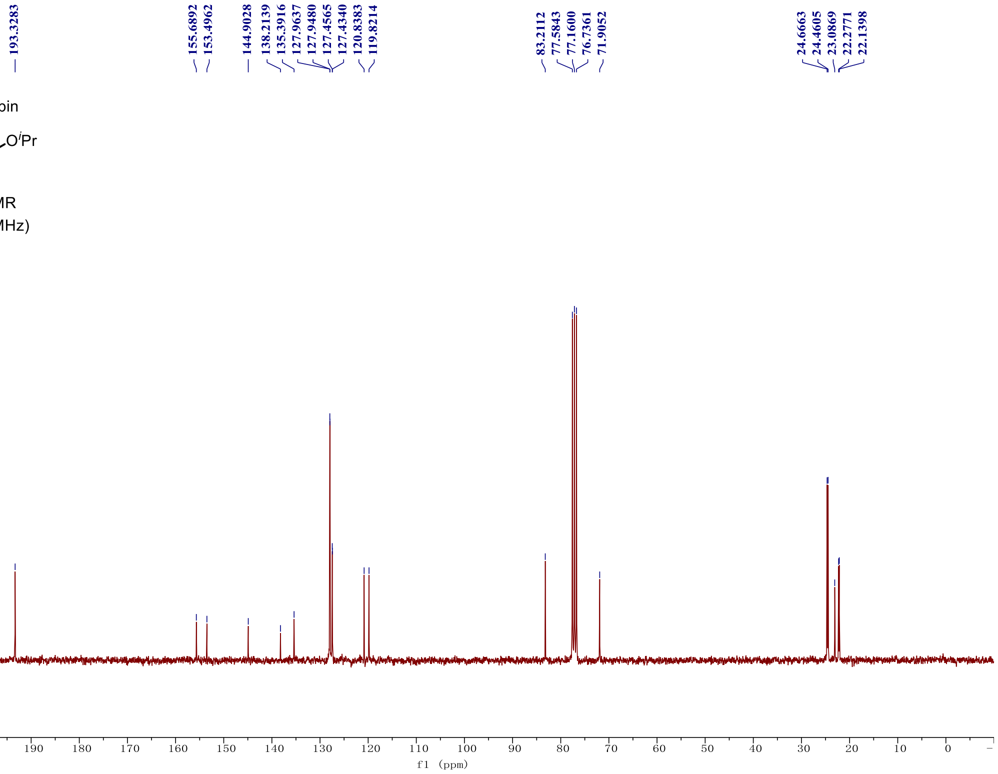

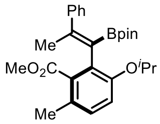

5o, ^{1}H NMR
($CDCl_3$, 300 MHz)

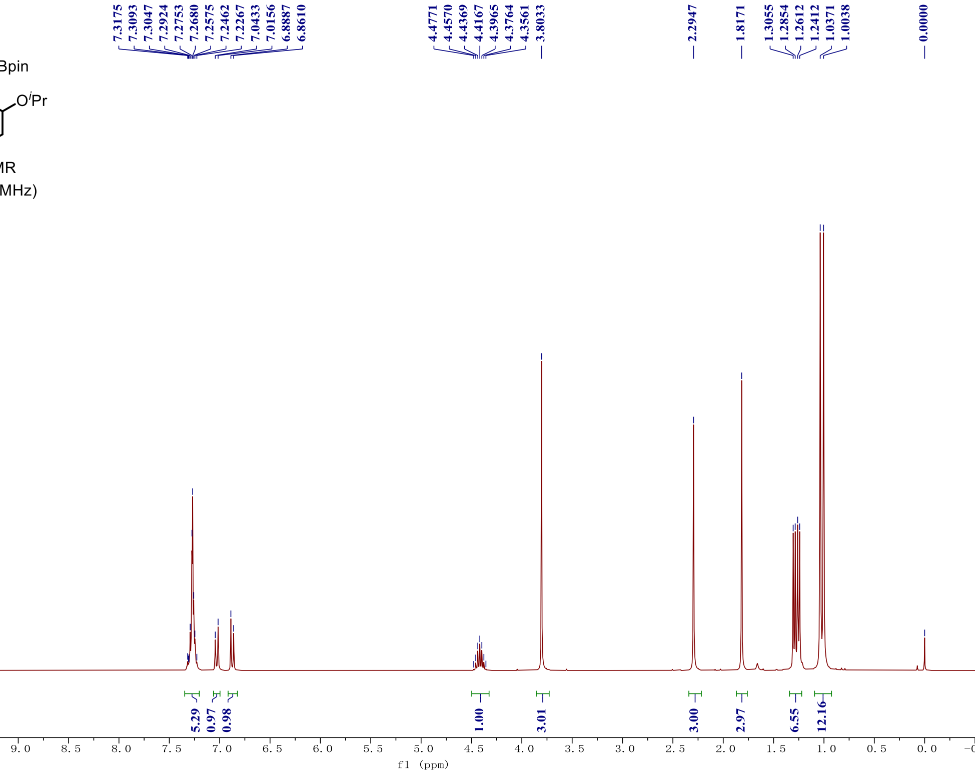

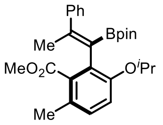

5o, ^{13}C NMR
($CDCl_3$, 75 MHz)

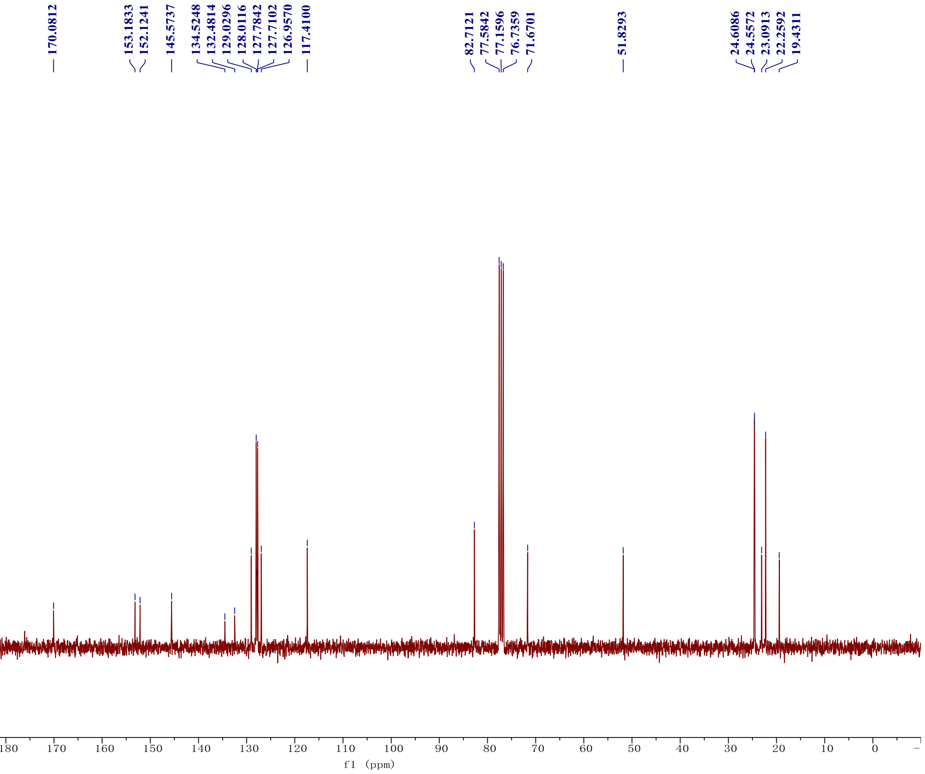

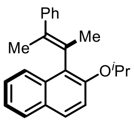

7a, ^{1}H NMR
($CDCl_3$, 300 MHz)

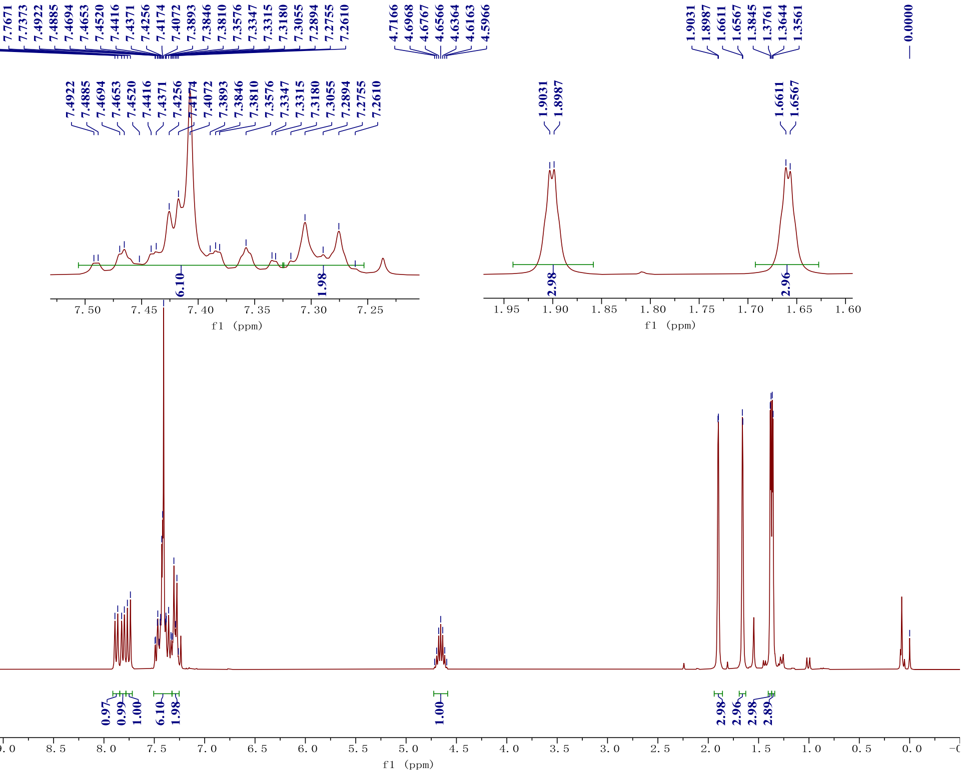

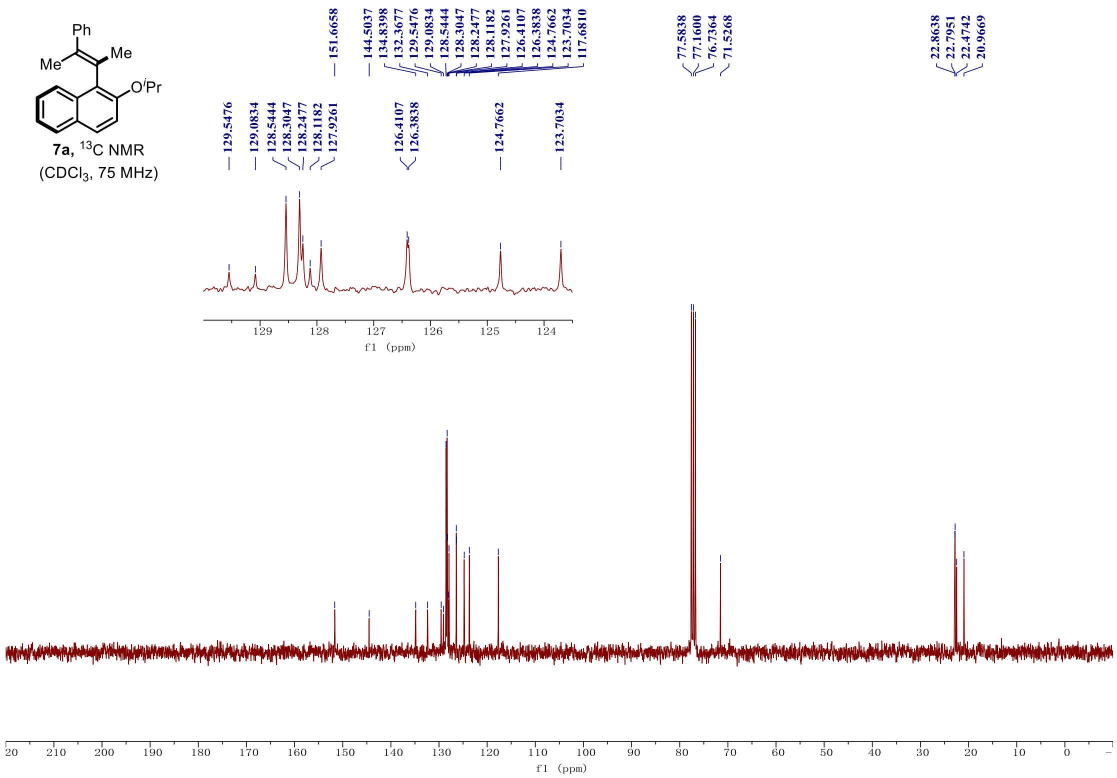

7a, ^{13}C NMR
($CDCl_3$, 75 MHz)

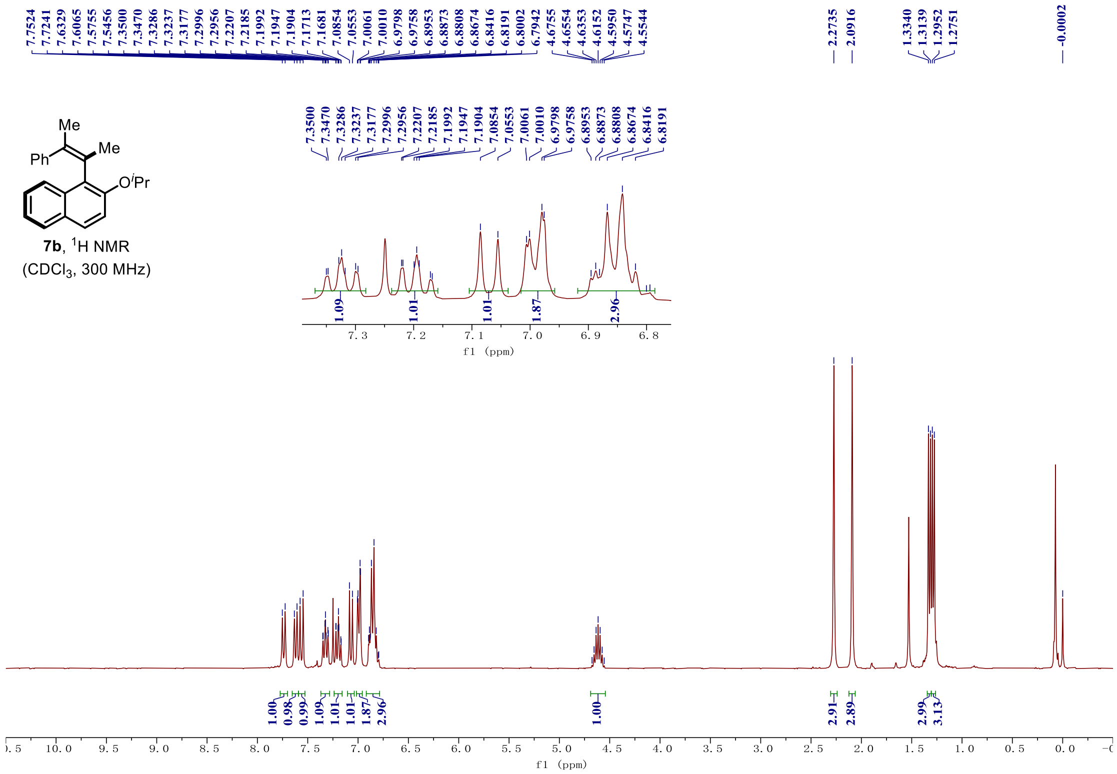

7b, ^{1}H NMR
($CDCl_3$, 300 MHz)

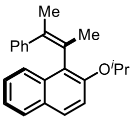

7b, ^{13}C NMR
($CDCl_3$, 75 MHz)

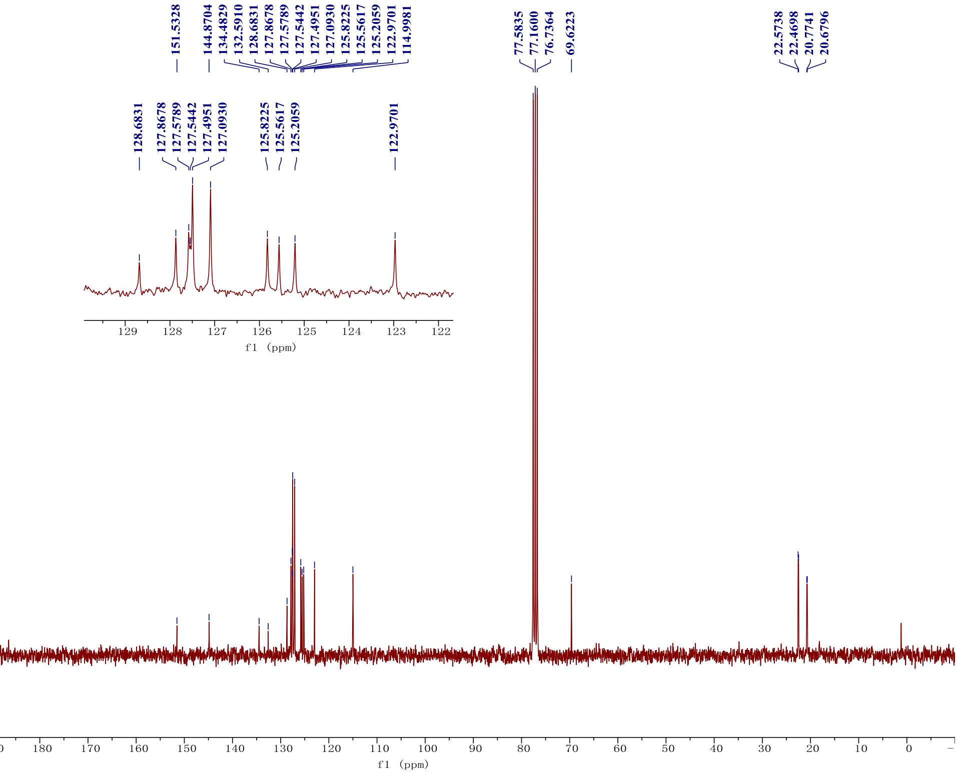

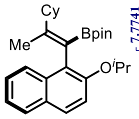

7c, ^{1}H NMR
($CDCl_3$, 300 MHz)

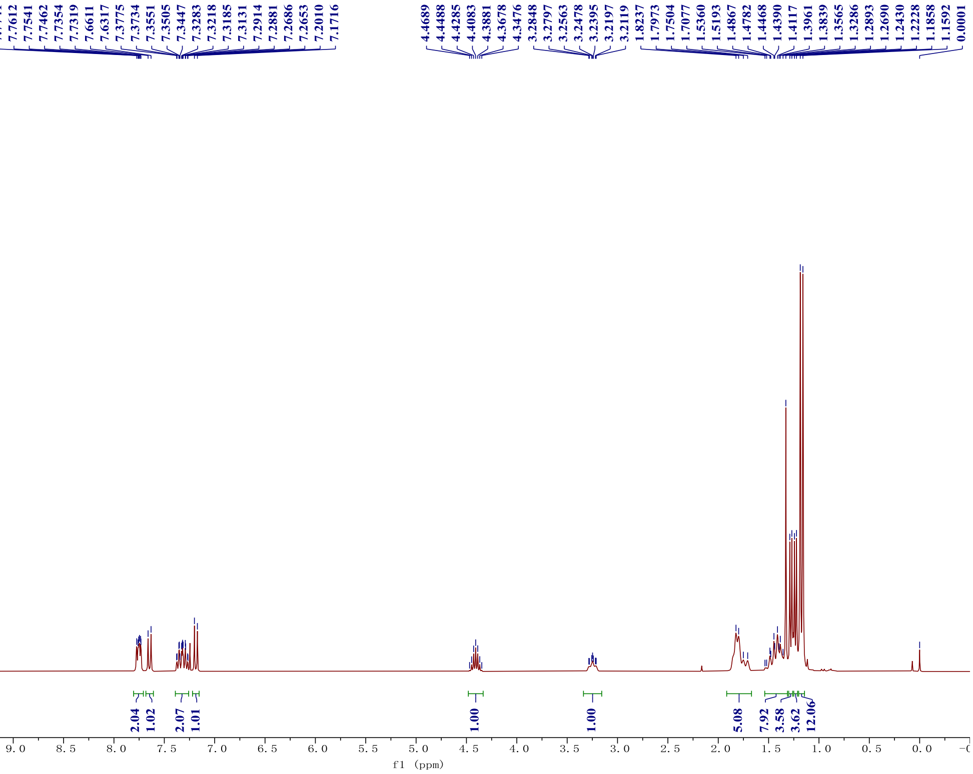

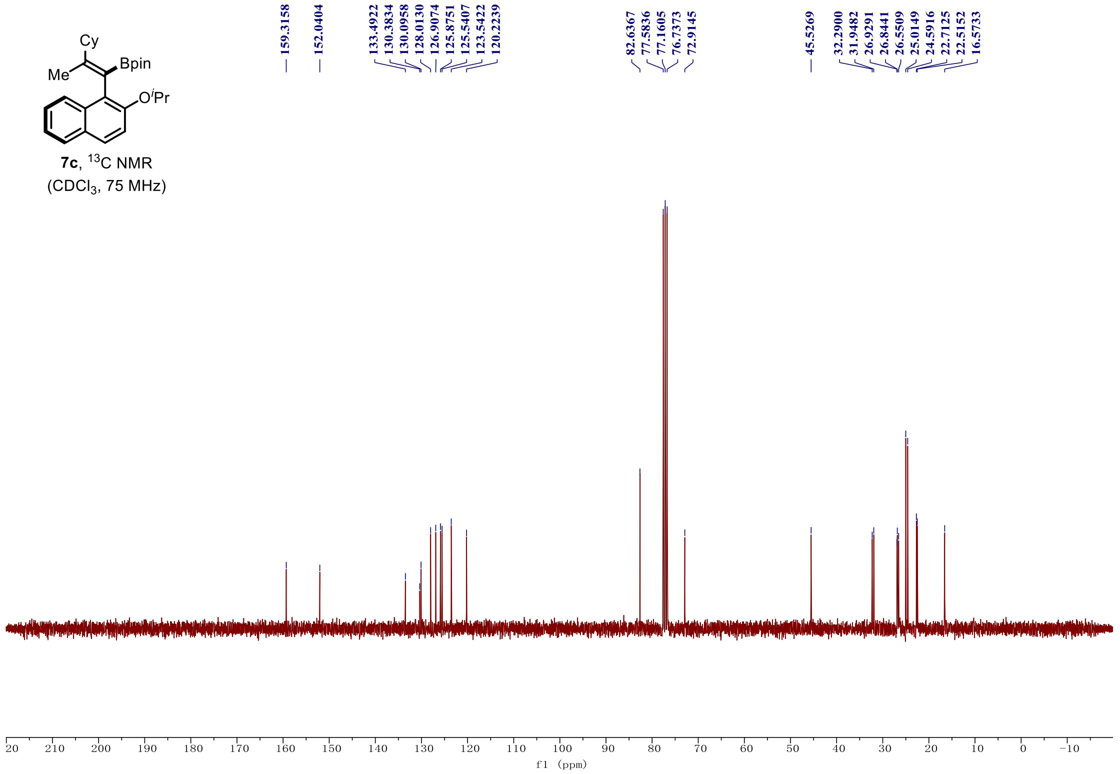

7c, ^{13}C NMR (CDCl$_3$, 75 MHz)

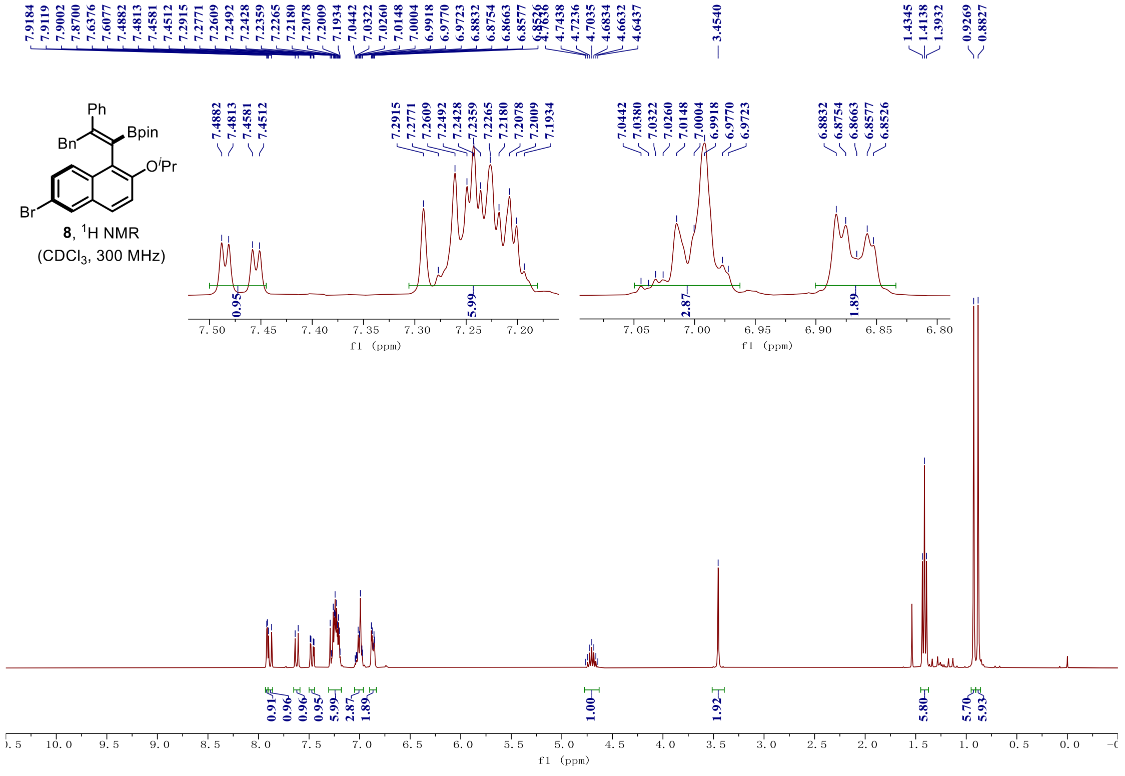

8, ^{1}H NMR (CDCl$_3$, 300 MHz)

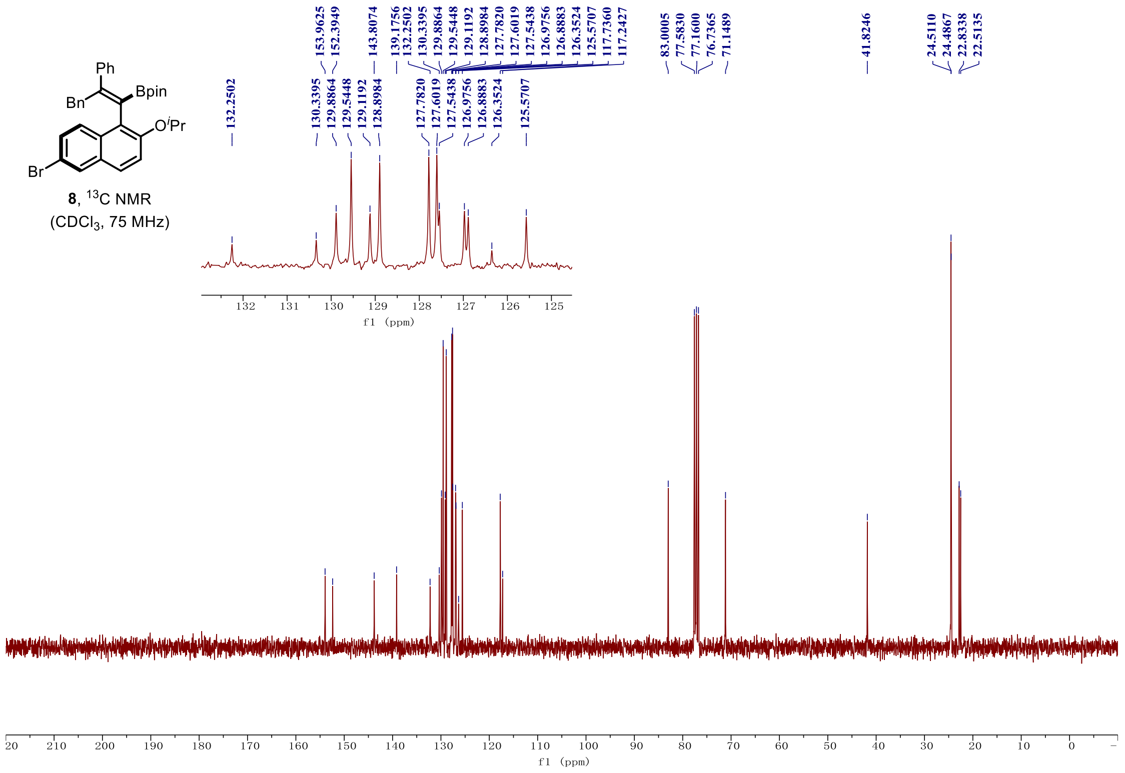

8, ^{13}C NMR
($CDCl_3$, 75 MHz)

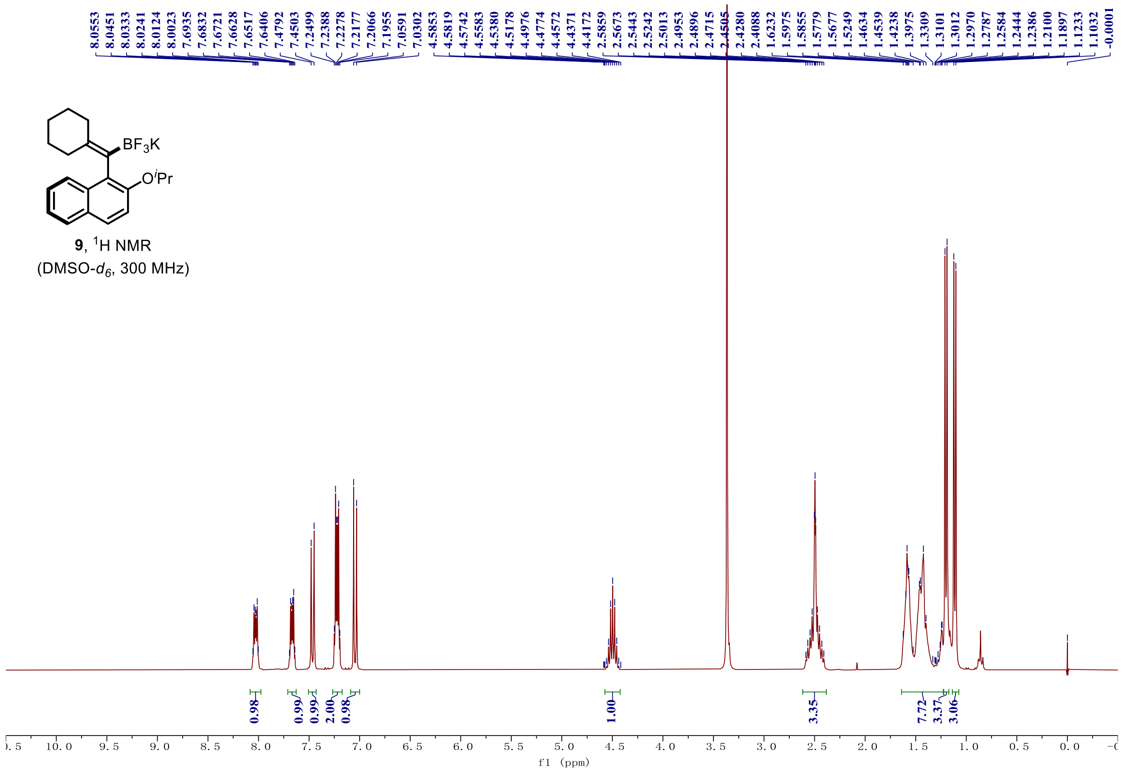

9, ^{1}H NMR
(DMSO-d_6, 300 MHz)

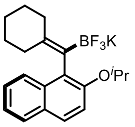

9, ^{13}C NMR
(DMSO-d_6, 75 MHz)

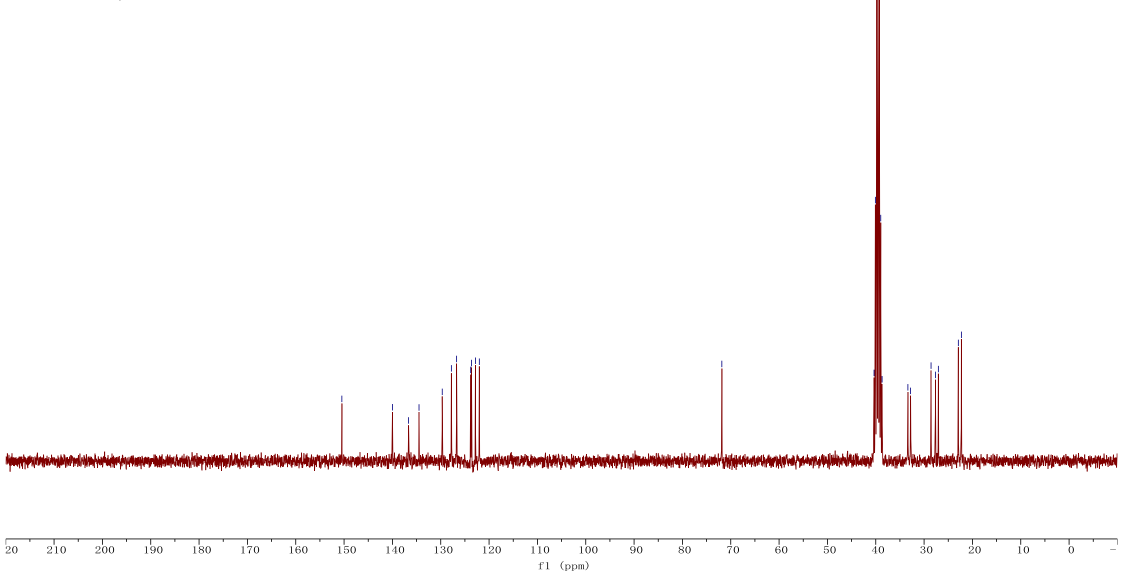

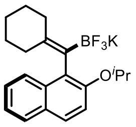

9, ^{19}F NMR
(DMSO-d_6, 282 MHz)

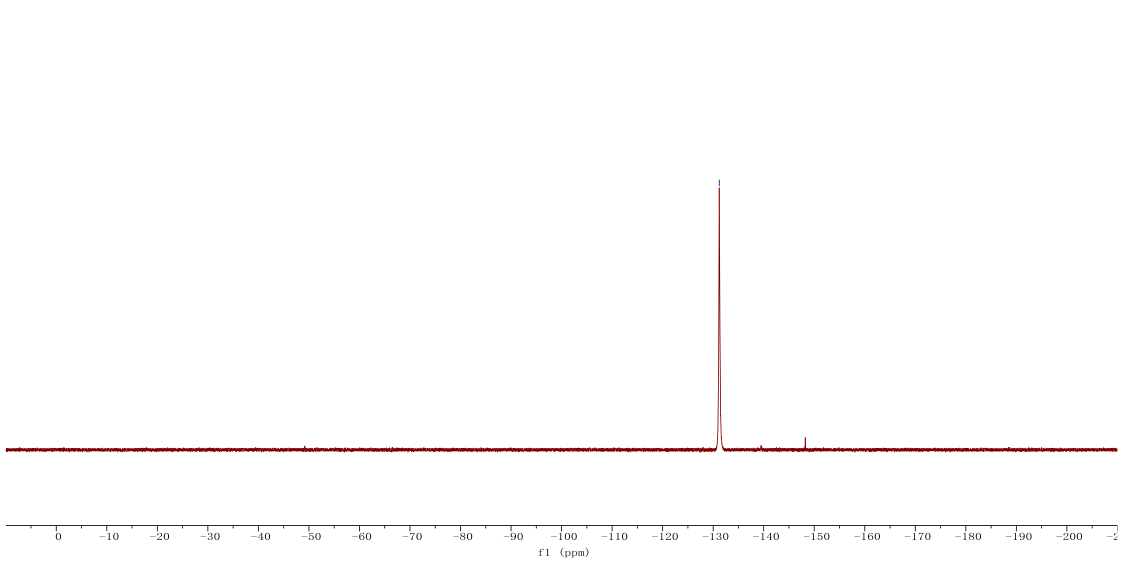

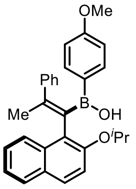

10, ^{1}H NMR
($CDCl_3$, 300 MHz)

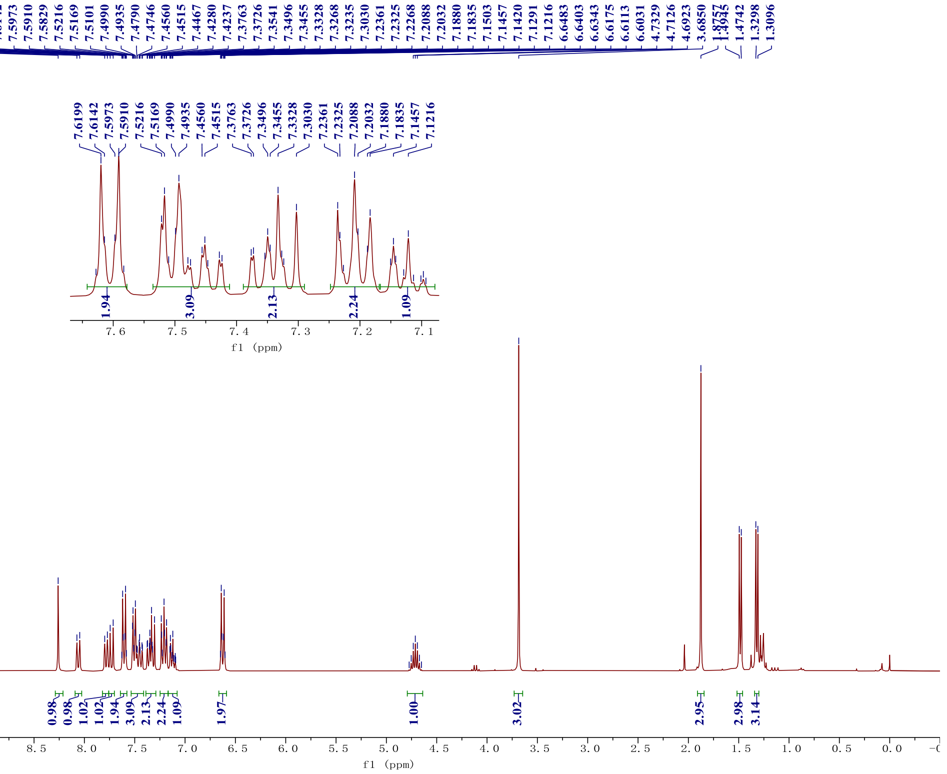

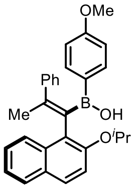

10, ^{13}C NMR
($CDCl_3$, 75 MHz)

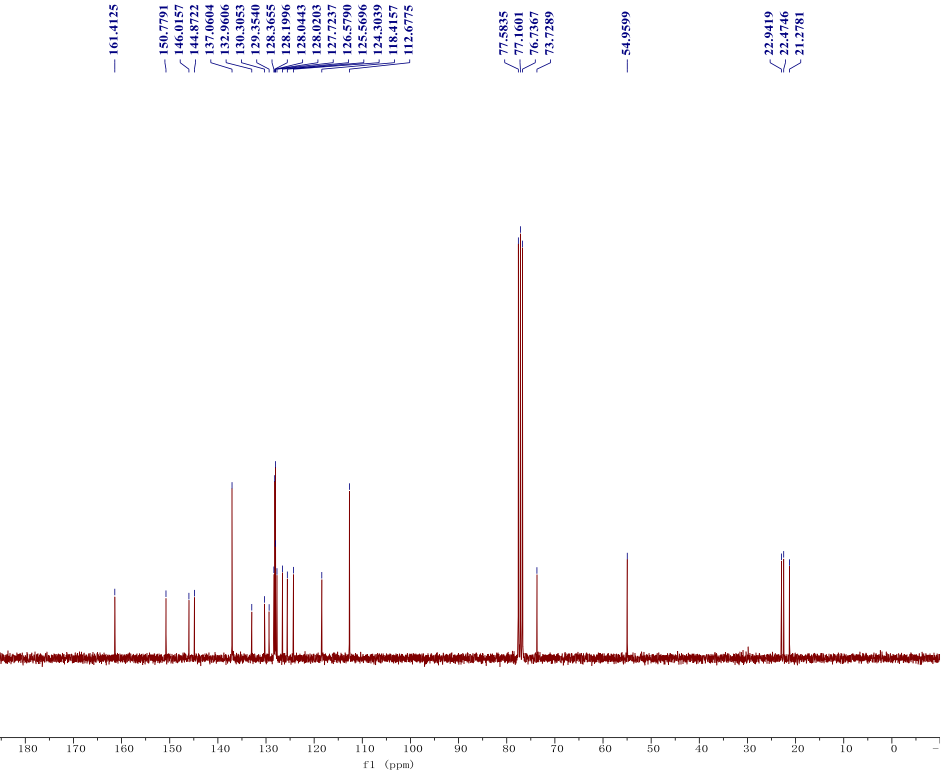

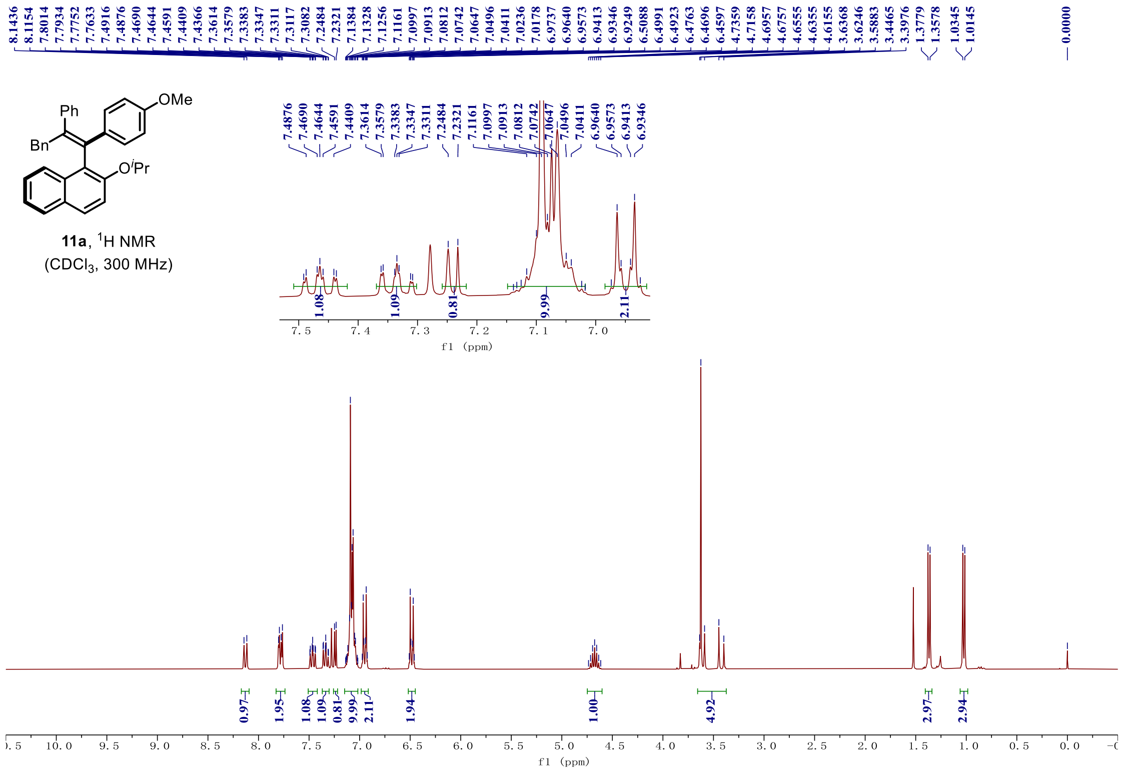

11a, ¹H NMR
($CDCl_3$, 300 MHz)

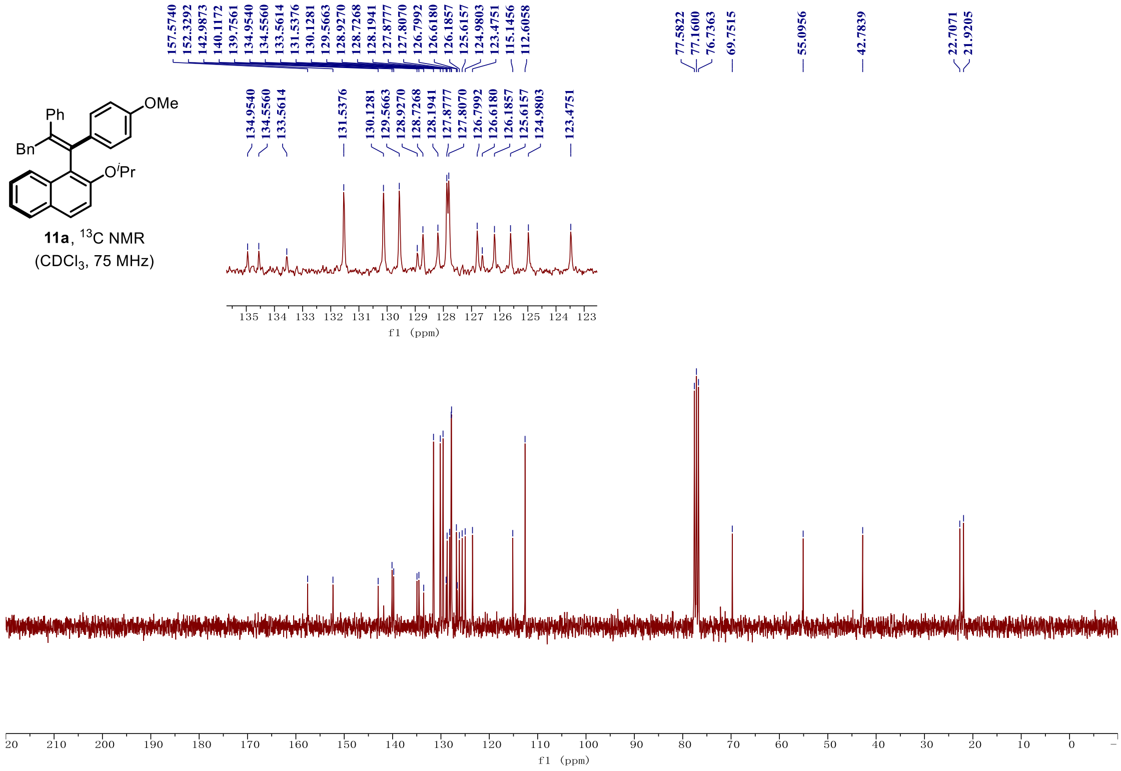

11a, ¹³C NMR
($CDCl_3$, 75 MHz)

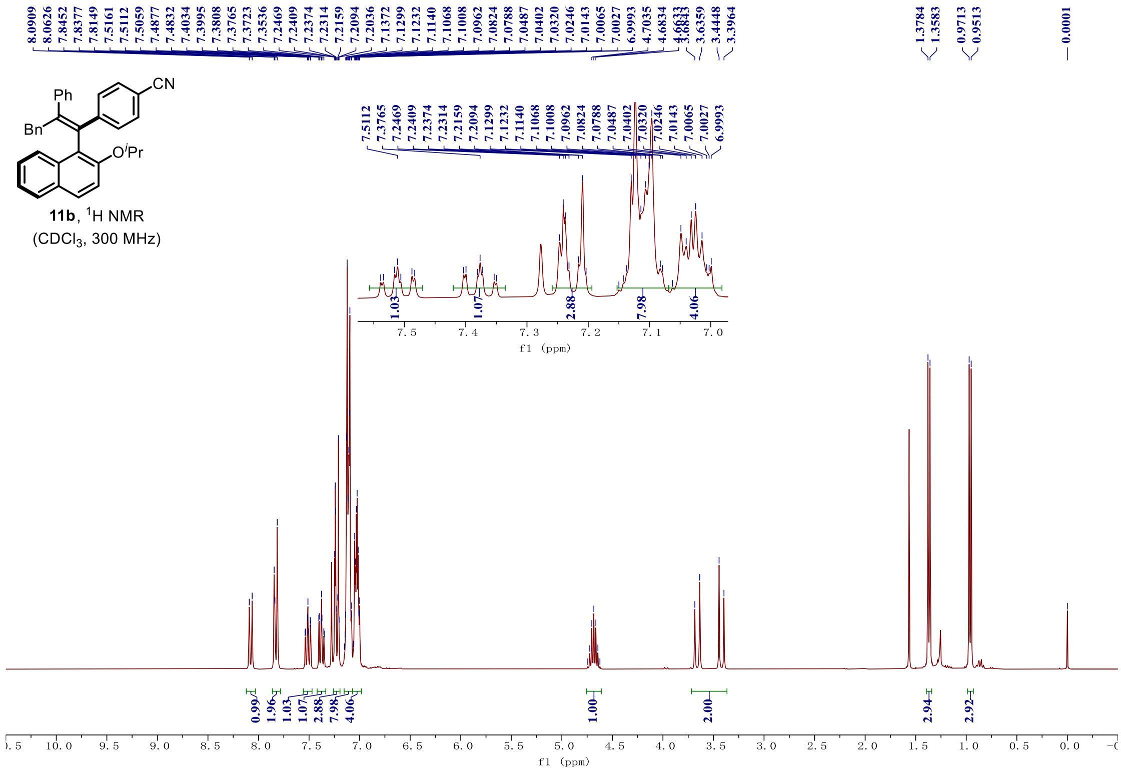
11b, ^{1}H NMR
(CDCl$_3$, 300 MHz)
f1 (ppm)

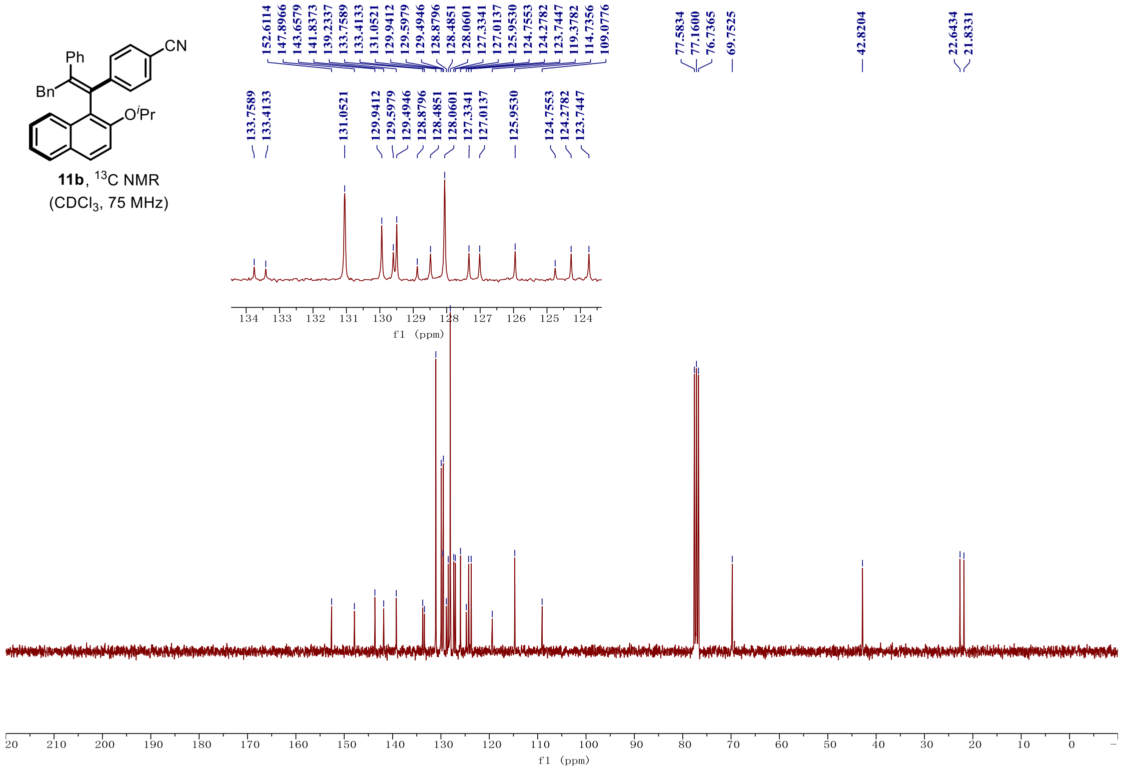
11b, ^{13}C NMR
(CDCl$_3$, 75 MHz)
f1 (ppm)

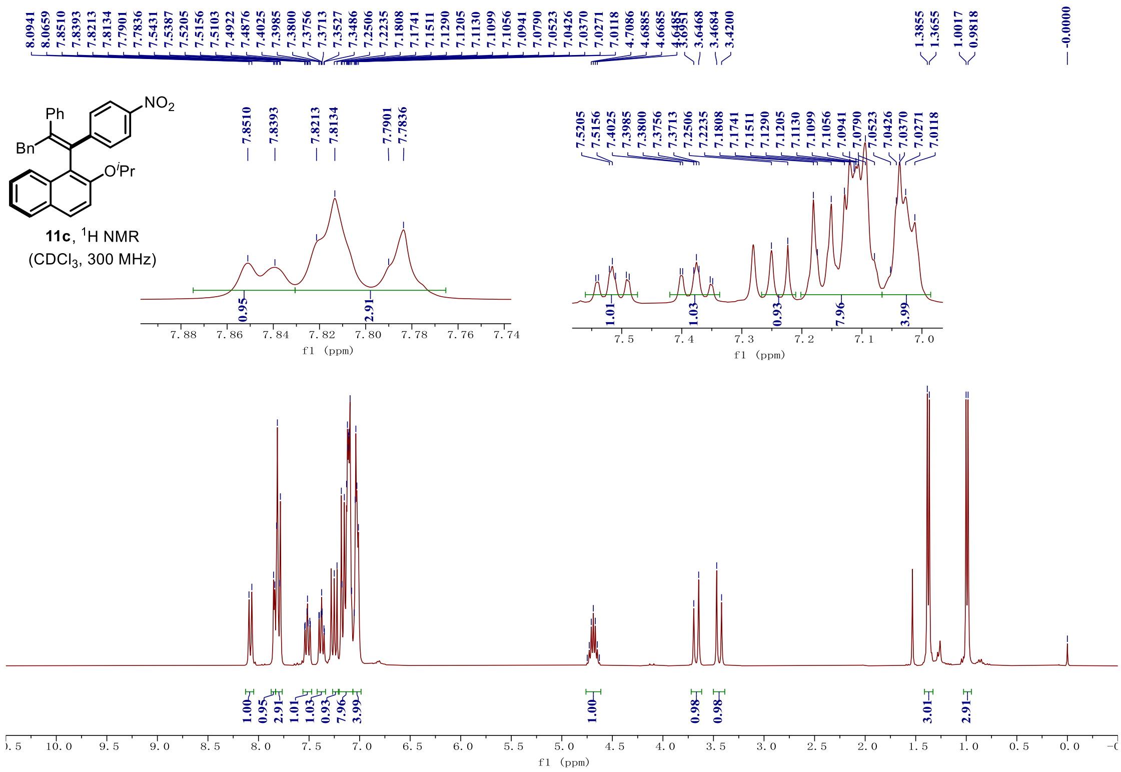
8.0941 8.0659 7.8510 7.8393 7.8213 7.8134 7.7901 7.7836 7.5431 7.5387 7.5205 7.5156 7.5103 7.4922 7.4876 7.4025 7.3985 7.3800 7.3756 7.3713 7.3527 7.3486 7.2506 7.2235 7.1808 7.1741 7.1511 7.1290 7.1205 7.1130 7.1099 7.1056 7.0941 7.0790 7.0523 7.0426 7.0370 7.0271 7.0118 4.7086 4.6885 4.6685 3.6951 3.6468 3.4684 3.4200 1.3855 1.3655 1.0017 0.9818 -0.0000
NO2
Ph
Bn
OⁱPr
11c, 1H NMR
(CDCl3, 300 MHz)
f1 (ppm)

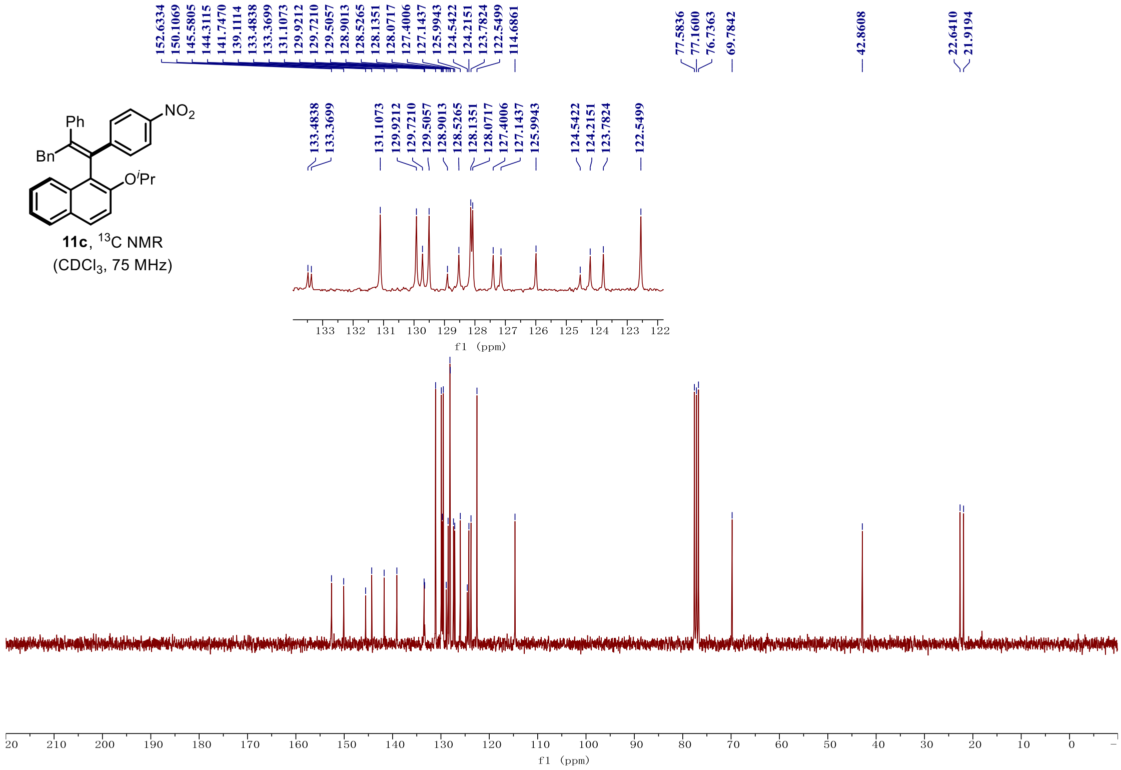
152.6334 150.1069 145.5805 144.3115 141.7470 139.1114 133.4838 133.3699 131.1073 129.9212 129.7210 129.5057 128.9013 128.5265 128.1351 128.0717 127.4006 127.1437 125.9943 124.5422 124.2151 123.7824 122.5499 114.6861 77.5836 77.1600 76.7363 69.7842 42.8608 22.6410 21.9194
NO2
Ph
Bn
OⁱPr
11c, 13C NMR
(CDCl3, 75 MHz)
f1 (ppm)

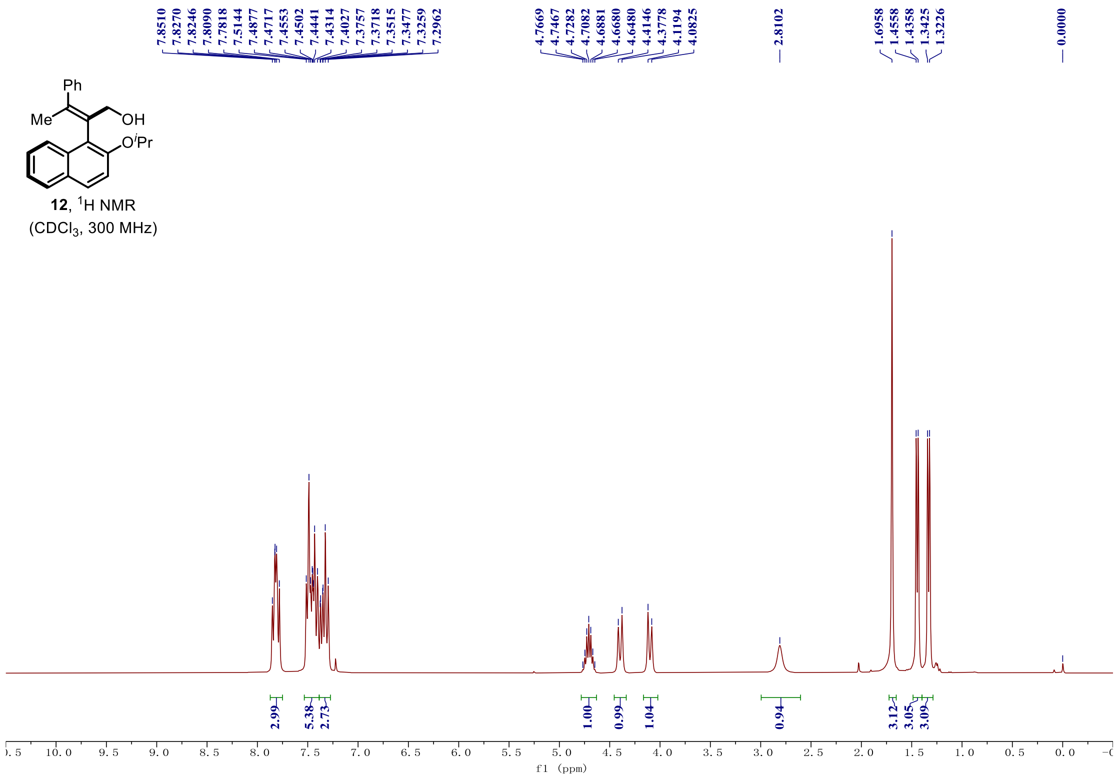

12, ^{1}H NMR
($CDCl_3$, 300 MHz)

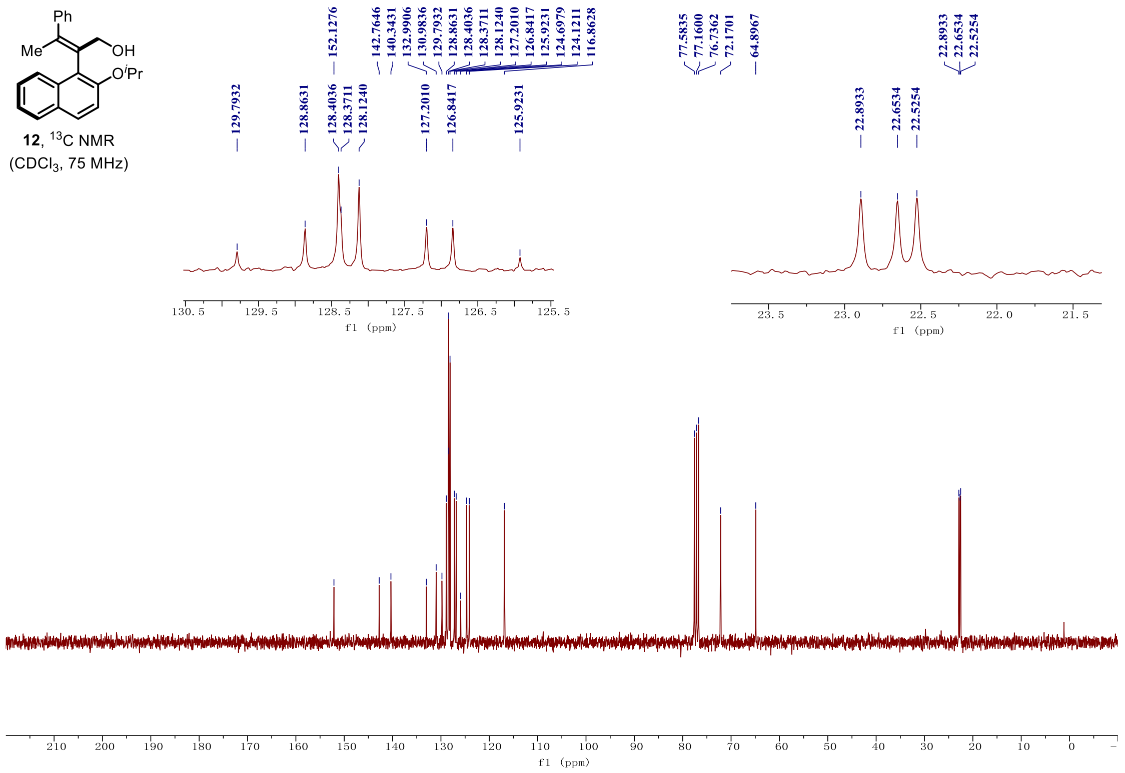

12, ^{13}C NMR
($CDCl_3$, 75 MHz)

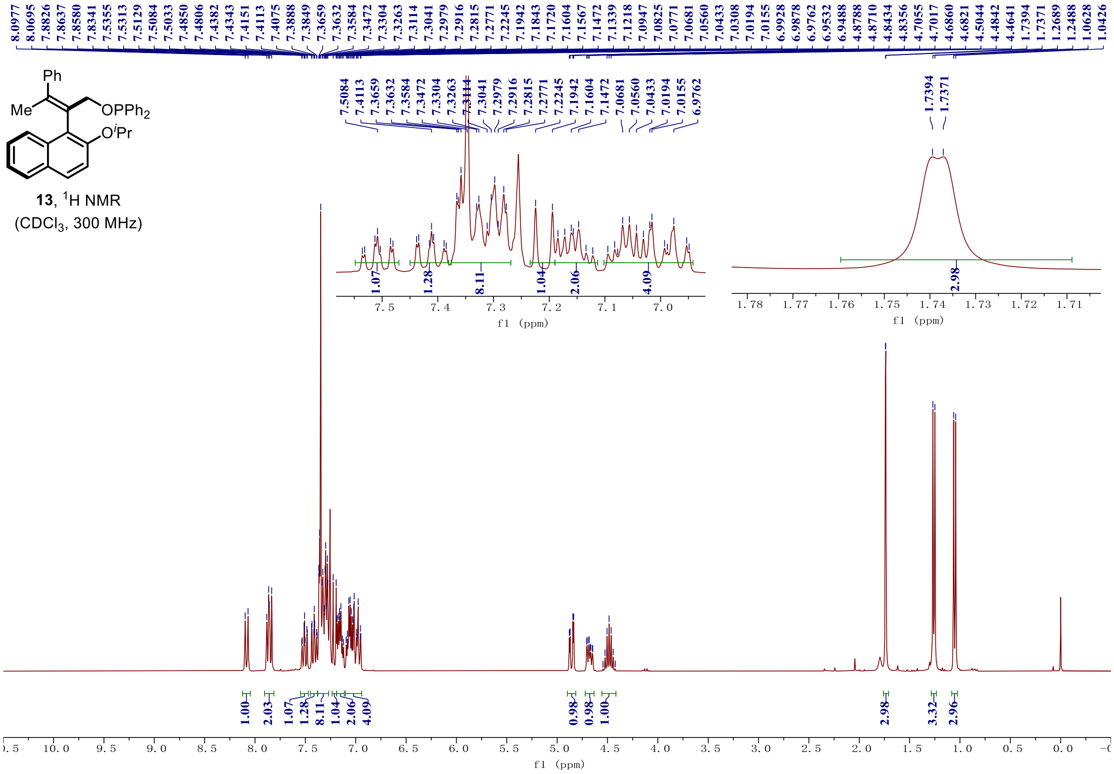

13, ^{1}H NMR (CDCl$_3$, 300 MHz)

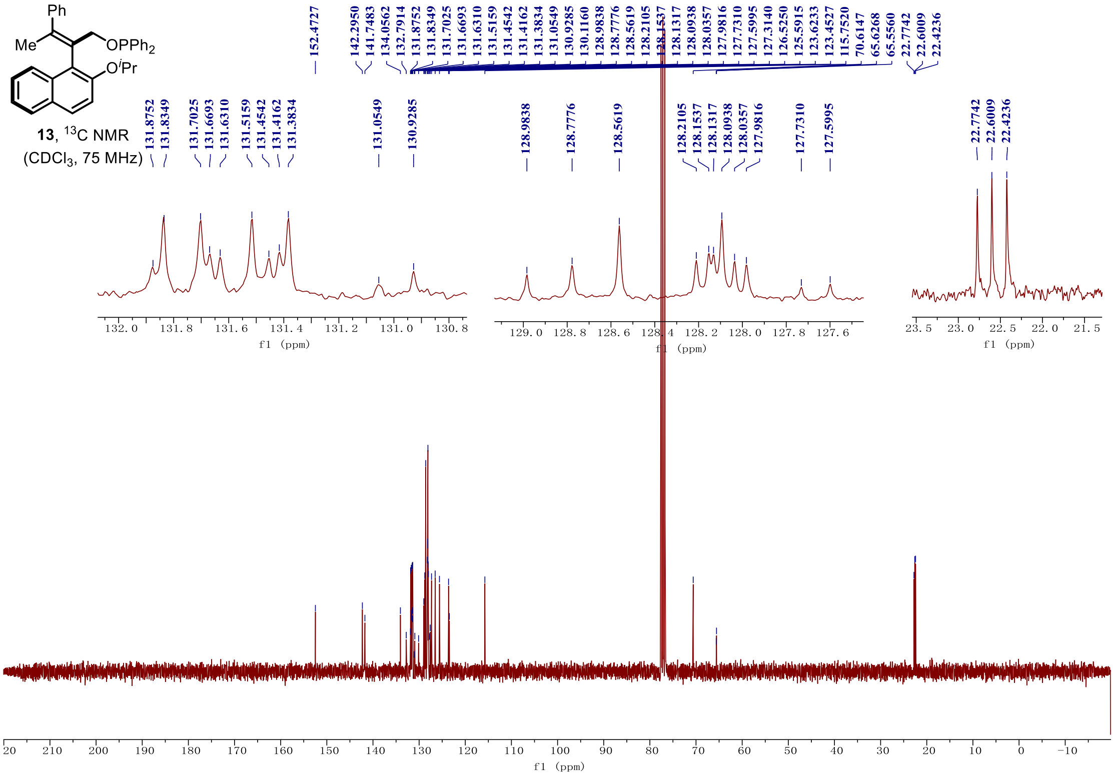

13, ^{13}C NMR (CDCl$_3$, 75 MHz)

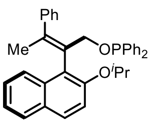

13, ^{31}P NMR
($CDCl_3$, 121 MHz)

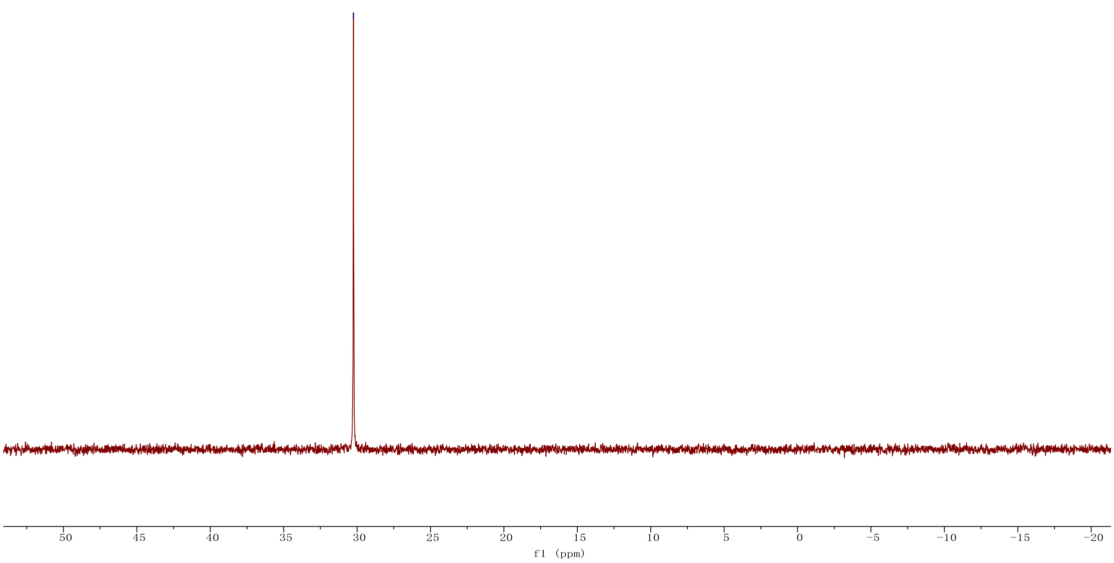

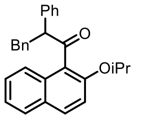

14, ^{1}H NMR
($CDCl_3$, 300 MHz)

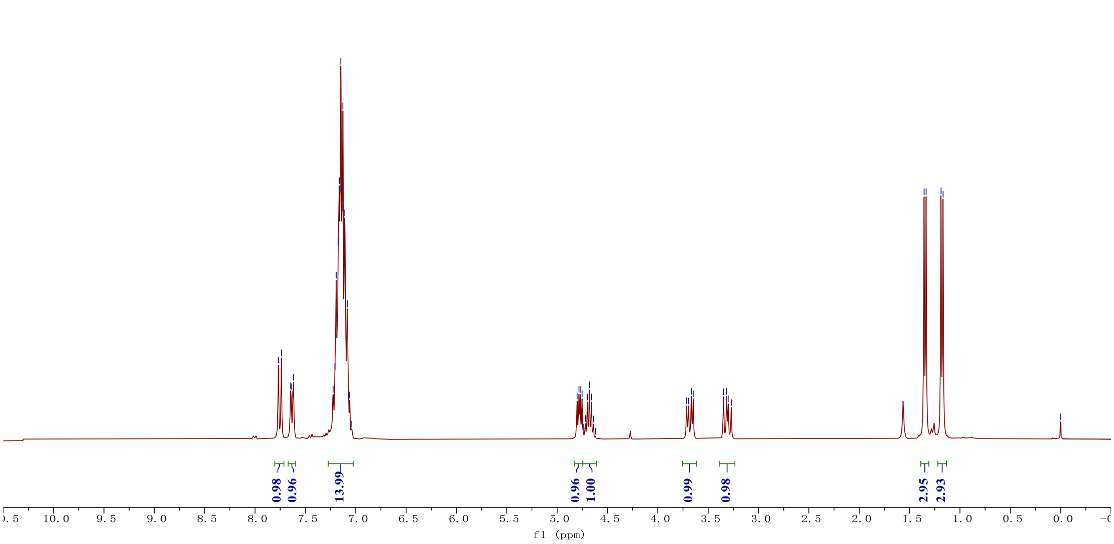

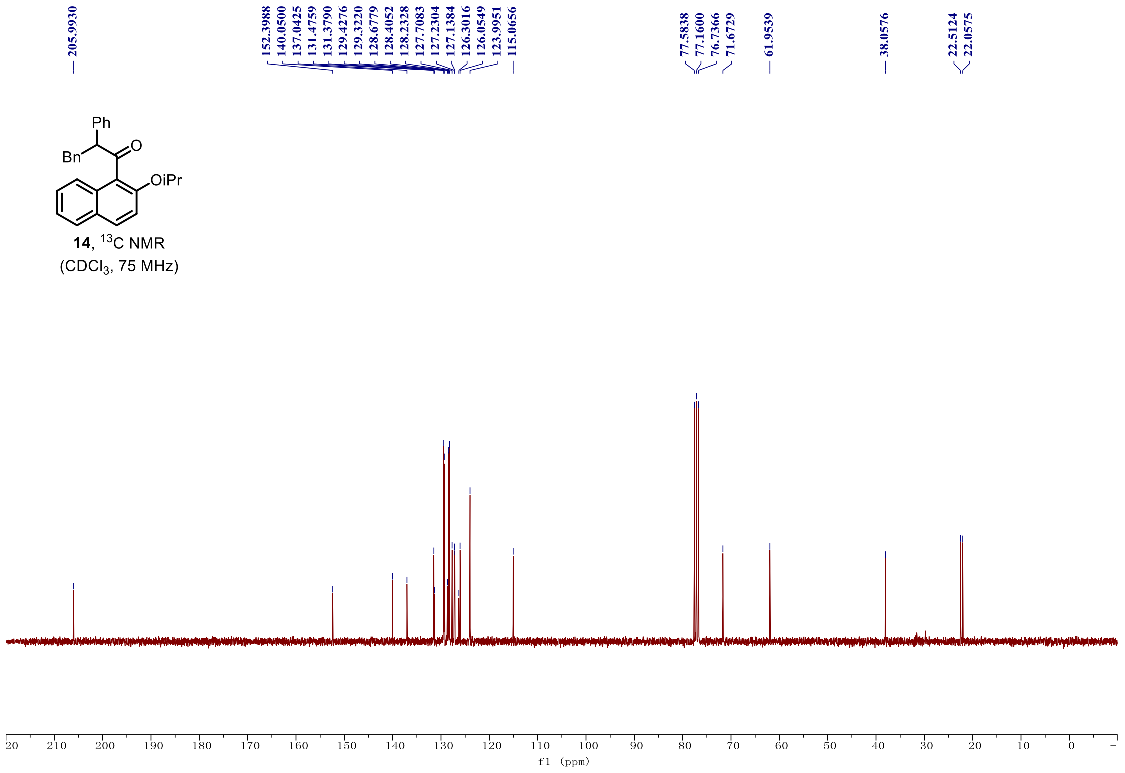

205.9930
152.3988
140.0500
137.0425
131.4759
131.3790
129.4276
129.3220
128.6779
128.4052
128.2328
127.7083
127.2304
127.1384
126.3016
126.0549
123.9951
115.0656
77.5838
77.1600
76.7366
71.6729
61.9539
38.0576
22.5124
22.0575
Ph
Bn
O
OiPr
14, ^{13}C NMR
($CDCl_3$, 75 MHz)
f1 (ppm)

12. References

1. X.-L. Min, X.-L. Zhang, W.-B. Yi, Y. He, *Nat. Commun*. **2022**, *13*, 373.
2. S.-Q. Jia, Z.-L. Chen, N. Zhang, Y. Tan, Y.-D. Liu, J. Deng, H.-L. Yan, *J. Am. Chem. Soc.* **2018**, *140*, 7056.
3. N. Sakiyama, K. Noguchi, K. Tanaka, *Angew. Chem., Int. Ed.* **2012**, *51*, 5976.
4. H.-J. Lee, H. Kim, J.-I. Yoshida, D.-P. Kim, *Chem. Commun.* **2018**, *54*, 547.
5. A. Mazzanti, M. Boffa, E. Marotta, M. Mancinelli, *J. Org. Chem.* **2019**, *84*, 12253.
6. Q.-Z. Li, Z.-H. Li, J.-C. Kang, T.-M. Ding, S.-Y. Zhang, *Chem. Catal.* **2022**, *2*, 3185.
7. F.-T. Sheng, S.-C. Wang, J.-Q. Zhou, C.-P. Chen, Y. Wang, S.-L. Zhu, *ACS Catal.* **2023**, *13*, 3841.
8. N.-D. Patel, J.-D. Sieber, S. Tcyrulnikov, B.-J. Simmons, D. Rivalti, K. Duvvuri, Y.-D. Zhang, D.-H. Gao, K.-R. Fandrick, N. Haddad, K.-S. Lao, H.-P.-R. Mangunuru, S. Biswas, B. Qu, N. Grinberg, S. Pennino, H. Lee, J.-H. Song, B.-F. Gupton, N.-K. Garg, M.-C. Kozlowski, C.-H. Senanayake, *ACS Catal*. **2018**, *8*, 10190.
9. M. Shimizu, C. Nakamaki, K. Shimono, M. Schelper, T. Kurahashi, T. Hiyama, *J. Am. Chem. Soc.* **2005**, *127*, 12506.
10. T. Hata, H. Kitagawa, H. Masai, T. Kurahashi, M. Shimizu, T. Hiyama, *Angew. Chem., Int. Ed.* **2001**, *40*, 790.
11. X. Liang, T. D. James, J. Zhao, *Tetrahedron* **2008**, *64*, 1309.
12. V. Bagutski, A. Ros, V. K. Aggarwal, *Tetrahedron* **2009**, *65*, 9956.
13. S.-S. Xun, H. Wang, C.-B. Yu, S.-M. Lu, Y.-G. Zhou, *Org. Lett.* **2023**, *25*, 7540.
14. S. Miyamura, M. Araki, T. Suzuki, J. Yamaguchi, K. Itami, *Angew. Chem., Int. Ed.* **2015**, *54*, 846.
15. S. P. Thomas, R. M. French, V. Jheengut, V. K. Aggarwal, *Chem. Rec.* **2009**, *9*, 24.
16. H.-X. Jiang, D.-D. Han, R.-P. Song, Q. Shi, X.-F. He, W.-Q. Kou, Q. Zhao, Y.-D. Shao, D.-J. Cheng, *Adv. Synth. Catal.* **2023**, *365*, 1398.
